# Novel Double-Modified Colchicine Derivatives Bearing 1,2,3-Triazole: Design, Synthesis, and Biological Activity Evaluation

**DOI:** 10.1021/acsomega.1c03948

**Published:** 2021-09-28

**Authors:** Julia Krzywik, Anna Nasulewicz-Goldeman, Witold Mozga, Joanna Wietrzyk, Adam Huczyński

**Affiliations:** † Department of Medical Chemistry, Faculty of Chemistry, Adam Mickiewicz University, Uniwersytetu Poznańskiego 8, 61-614 Poznań, Poland; ‡ TriMen Chemicals, Piłsudskiego 141, 92-318 Łódź, Poland; § Hirszfeld Institute of Immunology and Experimental Therapy, Polish Academy of Sciences, Rudolfa Weigla 12, 53-114 Wrocław, Poland

**Keywords:** anticancer agents, antiproliferative activity, colchicine azide, colchicine triazole, click chemistry

## Abstract

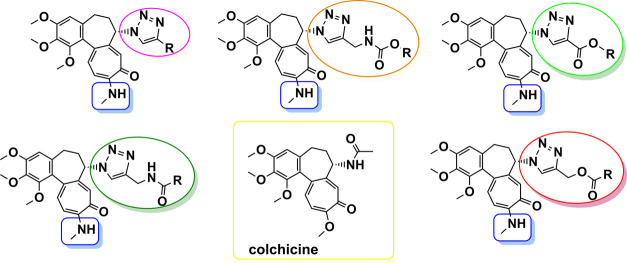

A series of 1,4-disubstituted 1,2,3-triazoles having 10-demethoxy-10-*N*-methylaminocolchicine core were designed and synthesized *via* the Cu(I)-catalyzed “click” reaction and screened for their *in vitro* cytotoxicity against four cancer cell lines (A549, MCF-7, LoVo, LoVo/DX) and one noncancerous cell line (BALB/3T3). Indexes of resistance (RI) and selectivity (SI) were also determined to assess the potential of the analogues to break drug resistance of the LoVo/DX cells and to verify their selectivity toward killing cancer cells over normal cells. The compounds with an ester or amide moiety in the fourth position of 1,2,3-triazole of 10-*N*-methylaminocolchicine turned out to have the greatest therapeutic potential (low IC_50_ values and favorable SI values), much better than that of unmodified colchicine or doxorubicin and cisplatin. Thus, they make a valuable clue for the further search for a drug having a colchicine scaffold.

## 1 Introduction

Colchicine **1** is a known active alkaloid that has been used for the treatment of acute gout, Behcet’s disease, or familial Mediterranean fever since ancient times. This compound occurs in the environment and is isolated mainly from *Colchicum autumnale* and *Gloriosa superba*.^1−4^ Due to its antimitotic properties, its skeleton has been the subject of incessant interest of researchers involved in the search for compounds with anticancer activity. It binds to tubulin, the protein that is the basic structural unit of microtubules building the mitotic spindle, causes changes in its structure, and stops the formation of microtubules. As a result, the cell cycle is arrested and apoptosis is induced.^5−7^

Colchicine has not been used in cancer chemotherapy as yet due to its relatively high toxicity and adverse side effects. Colchicine toxicity has been divided into three stages: gastrointestinal phase, the multiorgan failure phase, and the recovery phase, with a risk of sepsis. The side effects of colchicine generally concern the gastrointestinal tract. They are manifested by abdominal pain, nausea, vomiting, or diarrhea. Initial leukocytosis may lead to bone marrow depression which, in combination with gastrointestinal hemorrhages, may result in life-threatening anemia. Subsequent symptoms include electrolyte disturbances (*e.g.*, hypocalcemia), hematological disturbances (*e.g.*, thrombocytopenia), respiratory failure, arrhythmias, muscle weakness, and kidney and liver damage. All of these symptoms lead to multiple organ failure, sepsis, and ultimately can cause death. However, it should be noted that the occurrence of severe symptoms of poisoning, *e.g.*, bone marrow failure, paralysis, nerve inflammation or myopathy, and peripheral neuropathy, is very rare and is associated with the earlier occurrence of kidney or liver disorders in the patient. Colchicine’s toxicity is an extension of its ability to disrupt the microtubule network. The cells have a problem with the proper assembly of proteins, their morphology is changed, their mobility is reduced, mitosis is inhibited, and the mechanisms of endocytosis and exocytosis do not work properly. The culmination of these mechanisms in many different cells leads to multiorgan dysfunction.^1,8−12^

However, the research on the development of a colchicine analogue that would have at least nonworsening antiproliferative activity and would be devoid of at least some of the side effects of the unchanged compound has been ongoing for years. It would take too much space to list scientific publications describing new colchicine derivatives with a significantly increased selectivity of action. As an example, we can provide the analogues previously described by us: 7-*N*-(2-chlorobenzyl)-10-methylaminocolchcine or 7-*N*-(6-chlorohexyl)carbamate of 10-methylaminocolchicine for which the calculated selectivity coefficients were around 90 for human colon adenocarcinoma cell lines.^13,14^ A confirmation of the continuous interest in the properties of colchicine and its derivatives can be, for instance, the number of continually submitted patent applications, to protect, among others, the use of compounds as anticancer agents in the treatment or inhibition of cancer growth (WO2016059650, WO2019149884, or WO2021089715)^15^ and information about research in the ClinicalTrials.gov database^16^ (identifier: NCT04264260, NCT01935700, or NCT04823897).

**Figure 1:**
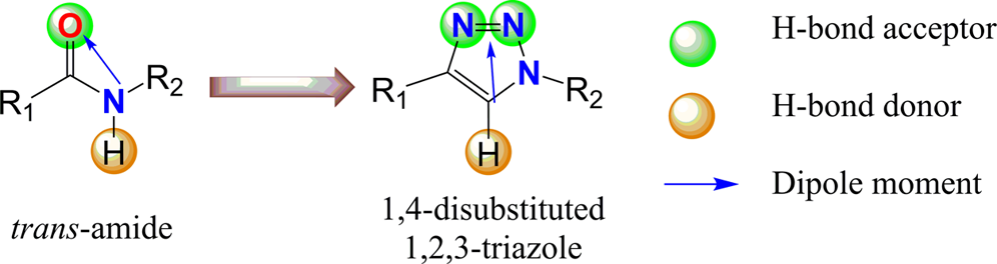
Structural features of disubstituted *trans*-amides and 1,2,3-triazoles as bioisosteres.

Colchicine can be modified in each of the three rings of which it consists: a trimethoxyphenyl ring A, a saturated seven-membered ring B, and a tropolone ring C. Nevertheless, the broadest possibility of modification is provided by the amine group at C7 position. The replacement of the acetamide located in the unmodified colchicine on carbon C7 with various bioisosteres may allow obtaining new compounds with improved biological, physicochemical, or pharmacokinetic properties. Many classes of amide bond surrogates are known, including carbamate, thioamide, 1,2,3-triazole, tetrazole, urea, sulfonamide, or phosphonamidate ones, and many reviews have been devoted to them.^17−19^ Nevertheless, the most popular peptidomimetic bioisosteres are 1,2,3-triazoles. Among them, 1,5-disubstituted triazoles are good isosteres of *cis*-amides, while 1,4-disubstituted triazoles mimic the most common *trans*-amides (Figure 1). The structural and electronic properties of triazoles allow them to well mimic the amide bond. They are better hydrogen bonds acceptors (HBA) and donors (HBD). In addition, the high dipole moment gives them also the possibility of dipole–dipole interactions and the aromatic ring is capable of π-stacking interactions. Thus, these nitrogen-containing heterocycles can easily interact *via* different pathways with biological/molecular targets, such as proteins, receptors, or enzymes, which play important roles in the organisms. On the other hand, 1,2,3-triazoles are stable under oxidative and reductive conditions and hydrolysis, which makes this moiety more resistant to metabolism in living cells, compared to amides.^18,20−24^

Many literature reports have shown that replacement of the amide bond with 1,2,3-triazole can improve the properties of chemical compounds with proven biological activity. For example, two analogues of cyclotetrapeptide *cyclo*-[Pro-Tyr-Pro-Val], in which one of the peptide bonds was replaced with a triazole ring, showed three times higher activity as tyrosinase inhibitors compared to the unmodified peptide.^25^ In pantothenamides, the triazole isoster in the place of the amide moiety allowed not only to prevent degradation of the compounds but also enhanced their antiplasmatic effect,^26^ while in phenacetin conjugates, in which the discussed moiety change allowed reduction of toxicity and at the same time led to improvement of the anti-inflammatory, antinociceptive, and antipyretic effects of unmodified phenacetin.^27^ It should also be mentioned that the compounds containing the 1,2,3-triazole skeleton in their structure exhibit a wide spectrum of biological properties such as antimicrobial,^28,29^ anticancer,^30,31^ antiviral,^32,33^ anti-inflammatory,^34^ antitubercular,^35,36^ or anti-Alzheimer’s disease.^37^

## 2 Results and Discussion

### 2.1 Chemistry

**Figure 2:**
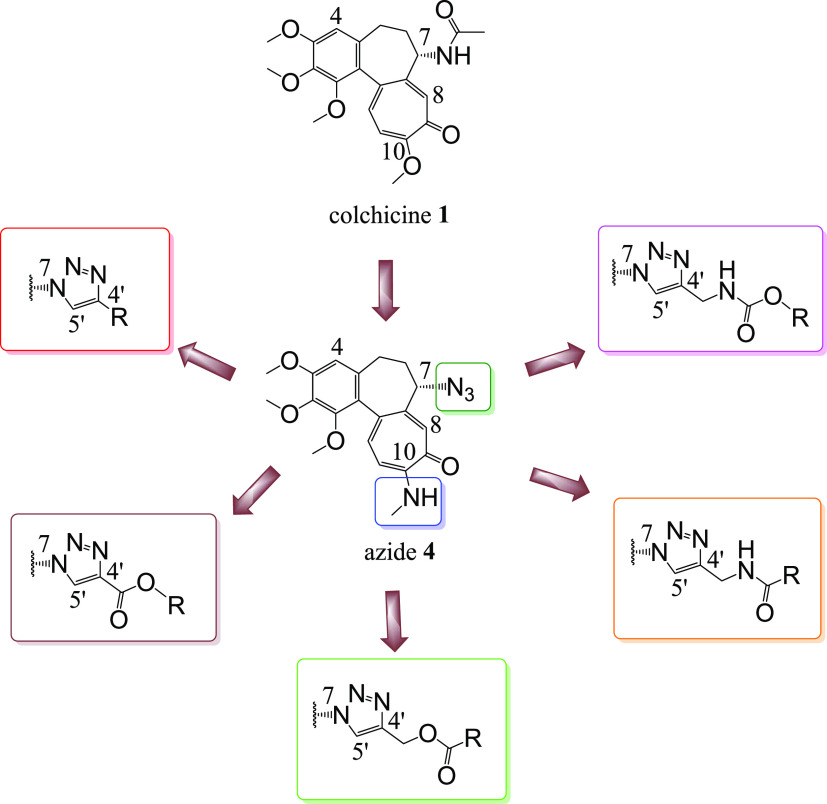
Structural modifications carried out on colchicine **1** to give 7-triazoles of 10-*N*-methylaminocolchicine.

**Scheme 1:**
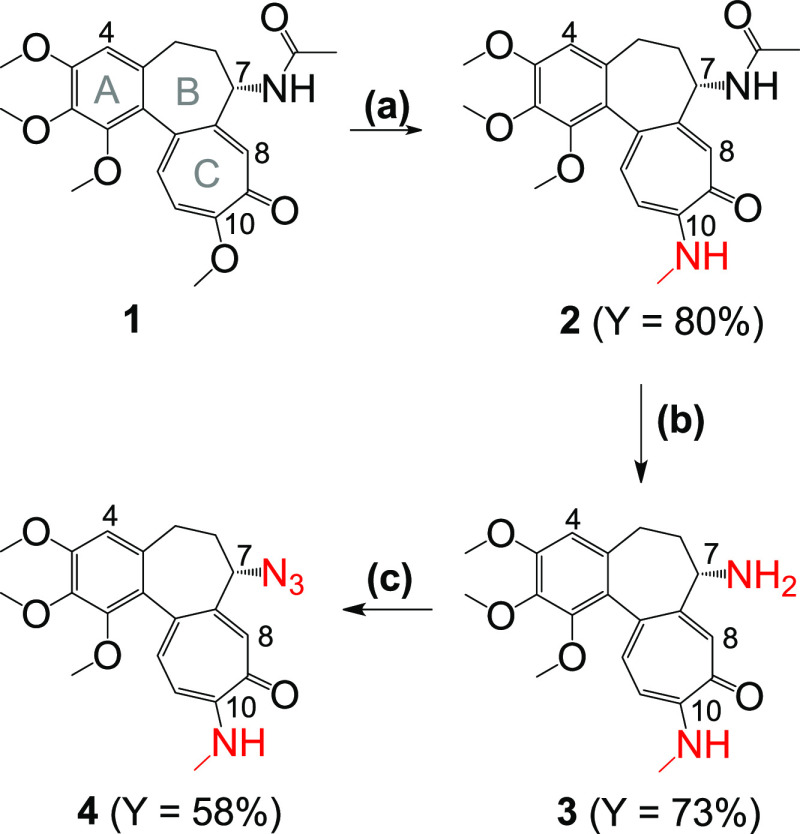
Synthesis of Azide **4** Reagents and conditions: (a) NH_2_CH_3_/EtOH, reflux; (b) 2 M HCl, reflux; (c) imidazole-1-sulfonyl azide hydrochloride, K_2_CO_3_, CuSO_4_, MeOH, 0 °C to room temperature (RT).

**Scheme 2:**
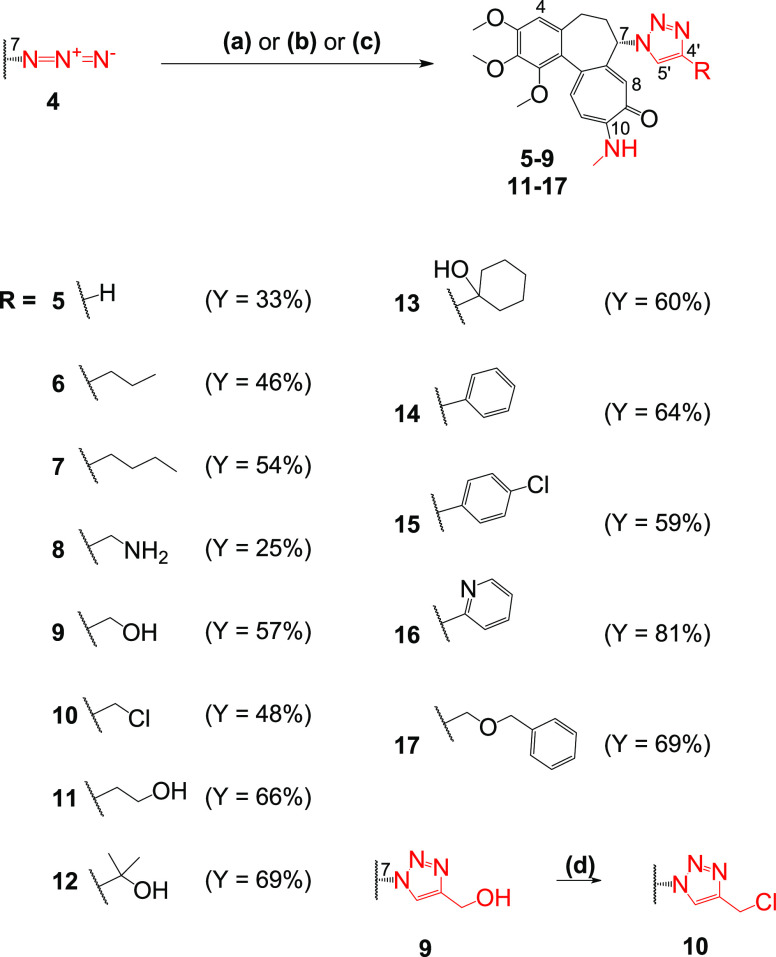
Synthesis of Colchicine Derivatives (**5**–**17**) Reagents and conditions: (a) trimethylsilylacetylene, CuSO_4_, sodium ascorbate, MeOH/H_2_O 10/1 v/v, 55 °C for **5**; (b) respective alkyne, CuI, *i*-Pr_2_NEt, MeOH, 55 °C for **6** and **7**; (c) respective alkyne, CuSO_4_, sodium ascorbate, MeOH/H_2_O 10/1 v/v, 55 °C for **8** and **9** and **11**–**17**; (d) CH_3_SO_2_Cl, Et_3_N, dichloromethane (DCM), 0 °C to RT for **10**.

**Scheme 3:**
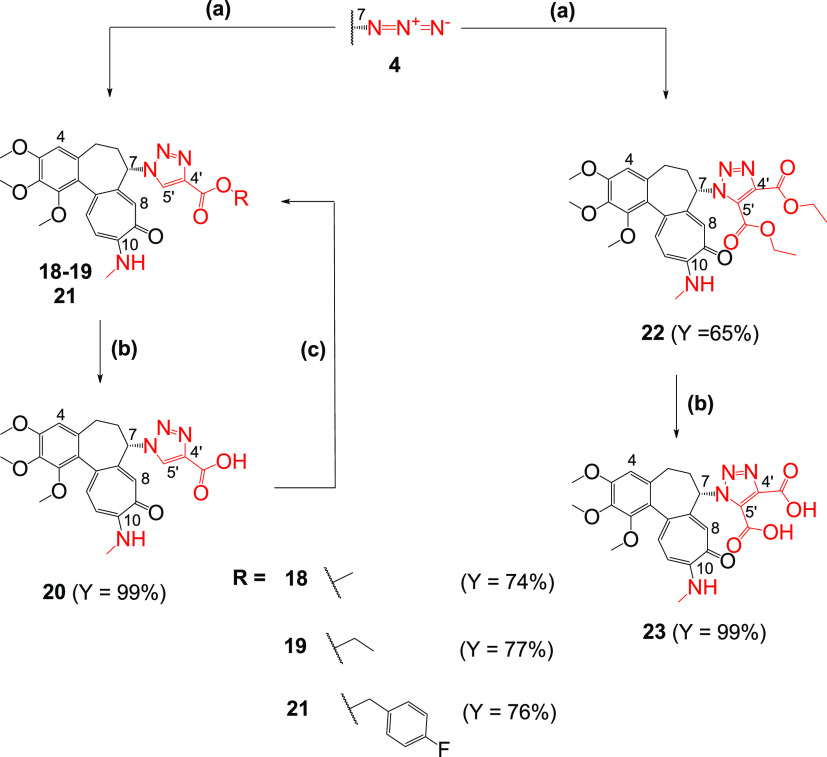
Synthesis of Colchicine Derivatives (**18**–**23**) Reagents and conditions: (a) methyl propiolate for **18**/ethyl propiolate for **19**/diethyl acetylenedicarboxylate for **22**, CuSO_4_, sodium ascorbate, MeOH/H_2_O 10/1 v/v, 55 °C; (b) 1 M NaOH, EtOH, RT; (c) *p*-fluorobenzyl bromide, K_2_CO_3_, dimethylformamide (DMF), RT for **21**.

**Scheme 4:**
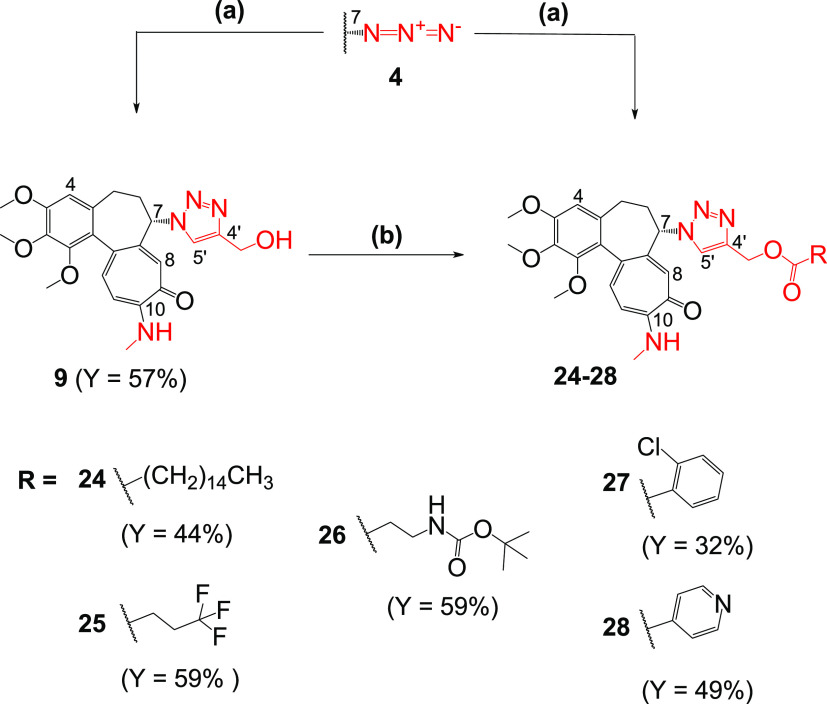
Synthesis of Colchicine Derivatives (**9** and **24**–**28**) Reagents and conditions: (a) propargyl alcohol or respective ester of propargyl alcohol, CuSO_4_, sodium ascorbate, MeOH/H_2_O 10/1 v/v, 55 °C for **9**, **26**, and **27**; (b) respective carboxylic acid, 1-(3-dimethylaminopropyl)-3-ethylcarbodiimide hydrochloride (EDCI), 4-(dimethylamino)pyridine (DMAP), DCM, RT for **24**, **25**, and **28**.

**Scheme 5:**
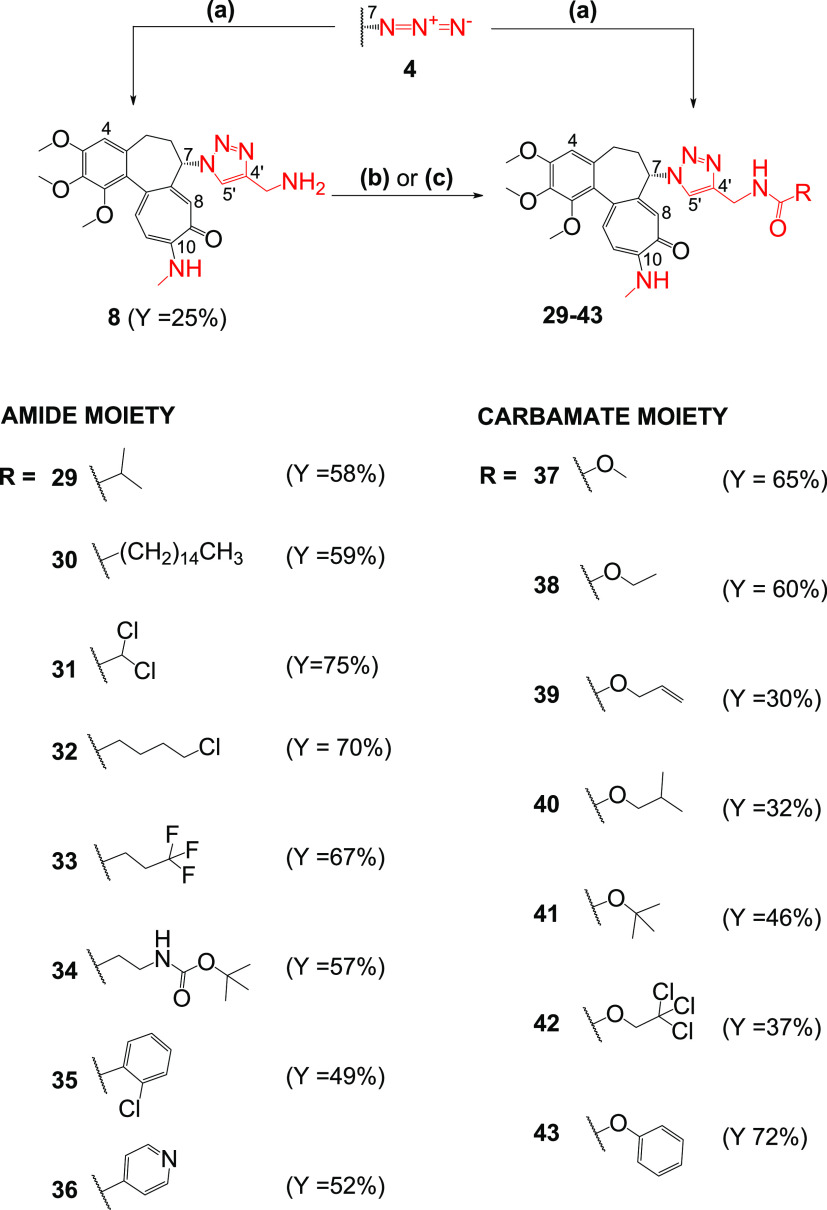
Synthesis of Colchicine Derivatives (**8** and **29**–**43**) Reagents and conditions: (a) propargylamine or respective amide/carbamate of propargylamine, CuSO_4_, sodium ascorbate, MeOH/H_2_O 10/1 v/v, 55 °C for **8**, **29**–**40**, and **43**; (b) di-*tert*-butyl dicarbonate, Et_3_N, DCM, RT for **41**; (c) 2,2,2-trichloroethyl chloroformate, Py, DCM, 0 °C to RT for **42**.

In view of the above, 1,2,3-triazoles represent a promising scaffold in the search for compounds with biological properties improved over those of the starting substances in which they could replace and mimic a certain moiety. Therefore, continuing our research on modifications of 10-*N*-methylaminocolchicine at position C7,^13,14,38,39^ and together with the reports on the cytotoxicity of colchicines with a triazole ring on C7 carbon (both those with simple substituents and more complex conjugates with ferrocenyl and ruthenocenyl),^40−43^ we synthesized 7-azido-10-*N*-methylaminocolchicine **4** and obtained a series of 39 derivatives (**5**–**43**) containing 1,2,3-triazole core. It should be noted that the double-modified derivatives of colchicine with a triazole ring have not been previously described. In addition, the colchicine analogues with a 1,2,3-triazole ring described so far did not contain such diverse moieties as those disclosed in this manuscript. On the fourth carbon of triazole, besides the alkyl chains substituted or not, cyclic or aromatic moieties, we also obtained fragments with ester bonds (derived from both propiolic acid and propargyl alcohol) as well as amide and urethane bonds (derived from modified propargylamine); see Figure 2 and Schemes 1–5.

A detailed description of the synthetic procedures is provided in Section 4. Scheme 1 shows the synthetic route to azide **4**. Compound **2** was obtained by the reaction with methylamine starting from colchicine **1**, and the amino group located on carbon C7 was subjected to deacetylation with an aqueous solution of HCl to give **3**.^38,44^ Derivative **3** was then treated with imidazole-1-sulfonyl azide hydrochloride in the presence of potassium carbonate and a catalytic amount of copper(II) sulfate, which allowed conversion of the primary amino group to an azide functionality,^45^ which was the starting material **4** for the synthesis of triazoles **5**–**43**. The synthesis of 7-azidocolchicine from 7-deacetylcolchicine with the use of a solution of trifluoromethanesulfonylazide, freshly prepared each time, is described in the literature.^46^ The use of a stable imidazole-1-sulfonyl azide in this work eliminated the stage of the diazotransfer reagent synthesis and provided a comparable yield of azide at carbon C7 of the colchicine derivative.

Copper(I)-catalyzed variant of the Huisgen 1,3-dipolar cycloaddition^46,47^ between 7-azido-10-*N*-methylaminocolchicine **4** and the corresponding alkynes (“click chemistry”) led to the formation of 39 1,2,3-triazoles (**5**–**43**) of the structures shown in Schemes 2–5. Most of the 1,4-disubstituted triazoles were obtained in the MeOH/H_2_O solvent mixture with the addition of catalytic amounts of copper(II) sulfate and sodium ascorbate. However, the preparation of two of the designed colchicine derivatives (**6** and **7**) required replacement of this catalytic system for copper(I) iodide and *N*,*N*-diisopropylethylamine.

First, azide **4** was reacted with commercially available alkynes to easily obtain 13 structurally diverse triazoles (**5**–**17**); see Scheme 2.

The derivative of propiolic acid (**20**) and three of its esters were also obtained (**18**, **19**, and **21**). To compare the properties of 1,4-disubstituted triazoles with those of 1,4,5-disubstituted triazoles, two derivatives, **22** and **23**, were designed and synthesized (Scheme 3) and their antiproliferative activities were also assessed.

The syntheses of five derivatives of 7-azido-10-*N*-methylaminocolchicine **4** and various propargyl esters were also performed (**24**–**28**, Scheme 4). Different acids were selected, containing both aliphatic and aromatic chains, to see if the connection of the appropriate carboxylic acid to the triazole obtained from propargyl alcohol could improve the biological activity of such derivatives.

Following this line of thinking, the simple structures of 1,4-disubstituted triazoles were also extended to include propargylamine derivatives (Scheme 5). Eight amides (**29**–**36**) and seven carbamates (**37**–**43**) were designed, prepared, and then their ability to inhibit cell proliferation was assessed. In both series of derivatives, various structures of side chains were selected to be able to draw initial relationships between the structure and biological activity (structure–activity relationship (SAR)) on the basis of the conducted study.

### 2.2 Characterization of Compounds

All compounds were purified by column flash chromatography on silica gel. The triazole compounds were characterized by liquid chromatography-mass spectrometry (LC-MS), ^1^H NMR, and ^13^C NMR, and the results are shown in Section 4 and the Supporting Information.

The electrospray ionization (ESI) mass spectrometry confirmed the structure of the synthesized compounds on the basis of the presence of the *m*/*z* signals assigned to the corresponding pseudomolecular ions ([M + H^+^] and/or [M – H^–^]) and/or complexes with alkali metal ions ([M + Na^+^]) and/or characteristic response to the presence of formic acid in the mobile phase ([M + HCOO^–^]). In the spectra of some compounds, also pseudomolecular ions corresponding to doubled masses were observed ([2M + H^+^] or [2M + Na^+^]). Details on individual signals can be found in the compound characterization in Section 4, and the original ESI-MS spectra are included in the Supporting Information.

In the NMR spectra, changes in the chemical shifts of certain atoms in the new derivatives appeared, relative to their positions in the spectra of the starting compounds **1**–**3** (Table S1, the labeling of the atoms is the same as in Figure 2 and Schemes 1–5). The chemical shift of H7 proton was visible at 4.53–4.73 ppm in amides **1** and **2**, at 3.72–3.75 ppm in the spectrum of compound **3**, and at 4.28–4.34 ppm in that of azide **4**. The chemical shift of C7 carbon observed at 52.7–54.0 ppm in the spectra of starting compounds **1**–**3** appeared at 63.6 ppm and that of **4** after introducing the azide moiety to 7-deacetyl-10-*N*-methylaminocolchicine. The formation of triazole changed the positions of chemical shifts of H7 and H8 protons and C7 carbon in the derivatives **5**–**43** compared to their positions in the spectra of **1**–**4**. The chemical shift assigned to H7 proton in the spectra of compounds **1**–**4** was found in the range 3.72–4.73 ppm, while in the spectra of compounds **5**–**43**, it was in the range of 5.35–5.96 ppm. The chemical shift of proton H8 in the spectra of **1**–**4** appeared at approximately 7.58 ppm, while in the spectra of triazoles **5**–**43**, it appeared at about 6.30 ppm. The chemical shift of C7 carbon in the spectra of colchicine derivatives **5**–**43** can be found at approximately 63.0 ppm, similar to that in the spectrum of azide **4**. The appearance of a characteristic singlet of aromatic protons H5′ in the region 7.40–8.27 ppm in the ^1^H NMR spectra and the signal assigned to carbons C5′ in the range 120.2–124.9 ppm in the ^13^C NMR spectra confirmed the formation of the triazole ring. The presence of esters, amides, or carbamates in derivatives **18**–**28**, **29**–**36**, and **37**–**43** was confirmed by the appearance of characteristic signals of a carbonyl group and proton of these moieties (Table S1 and NMR spectra).

Further evidence of triazole formation is the absence of the three characteristic bands at approximately 2100, 2120, and 3300 cm^–1^ in the Fourier transform infrared (FT-IR) spectra. The first one, located near 2100 cm^–1^, is assigned to the *v*(N_3_) stretching vibrations and is observed in the FT-IR spectrum of azide **4**. The two bands at 2120 and 3300 cm^–1^ are attributed to the *v*(C=C) and *v*(=C–H) stretching vibrations of alkynes. These bands are not present in the FT-IR spectra of compounds **5**–**43** after triazole ring formation. The exemplary FT-IR spectra of compounds **2**–**4**, **9** as well as propargyl alcohol are compared in Figures S126 and S127.

### 2.3 *In Silico* Calculations of the Physicochemical Properties (Drug-Likeness Filters)

Advances in medical chemistry have provided tools to predict the physicochemical and druglike properties of drug candidates, which allowed avoiding slow and costly *in vivo* testing in the early stages of research.^48^ Using the Molinspiration database, we determined the physicochemical properties of a series of 39 derivatives of colchicine with a triazole ring (**5**–**43**) and of the starting compounds (**1**–**4**).^49^ We used two methods, the Lipinski and Veber rules, to study the bioavailability of the compounds presented in this work.^50,51^ These rules help to identify the molecules that may have problems with diffusion through lipid barriers or solubility in aqueous body fluids. The Lipinski’s rules are: molecular weight (MW) **≤**500 Da, octanol/water partition coefficient (clog *P*) **≤**5, number of hydrogen-bond donors (NHD) **≤**5, and number of hydrogen-bond acceptors (NHA) **≤**10. The Veber’s rules are: number of rotatable bonds (NBR) **≤**10 and topological polar surface area (TPSA) **≤**140 Å^2^. The properties are tabulated in Table S2.

From the 43 compounds studied, more than half had molecular weight higher than 500 Da, which means they may have difficulty crossing cell membranes. However, the fact that they do not satisfy the molecular weight rule alone does not unequivocally classify them as poorly available after oral administration. In turn, all compounds except four (**15**, **24**, **27**, and **30**) were characterized by clog* P* values lower than 5. Therefore, they should exhibit good membrane permeability and elimination by metabolism, but not the best solubility in aqueous medium and gastric tolerance.^52−54^ All of the studied compounds had from 1–3 hydrogen-bond donors and 6–13 hydrogen-bond acceptors. Thus, compounds **22**, **23**, **26**, **34**, and **36**–**43** do not meet the Lipinski rule concerning NHA that should be ≤10. Taking into account all four descriptors together (MW, clog *P*, NHD, NHA), the number of violations (NV) of the Lipinski rule was determined (Table S2). So, 15 compounds presented here with NV ≥ 1 are considered to be marginal for further development and 28 molecules with NV ≤ 1 theoretically will not have problems with oral bioavailability.

For 36 of the obtained derivatives, the number of rotatable bonds was ≤10, so they met the first Veber’s rule, which means that these molecules have reduced flexibility so any possible conformational changes upon binding to the molecular target would be insignificant. Additionally, all of the molecules except only three (**23**, **26**, and **34**) had polar surface area ≤140 Å^2^, which theoretically implies good biological membrane permeability and good oral availability.^51^

Medicinal chemistry tools used here allowed a rough assessment of the physicochemical profile of drug candidates. However, it should be remembered that the *in silico* predicted properties will not determine the ultimate biological properties of the compound. In addition, the rules of drug-likeness may not be generally applicable to all classes of compounds or therapeutic targets and each route of drug administration has different restrictions and barriers. Furthermore, the constantly developing field of drug formulation provides a growing number of new solutions that allow avoiding the limitations of the bioavailability of active substances. Thus, *in silico* studies can help in drug discovery but do not replace pharmacokinetics and other *in vivo* tests.

### 2.4 *In Vitro* Determination of Drug-Induced Inhibition of Human Cancer Cell Lines Growth

The newly designed and prepared doubly modified colchicine derivatives containing triazole ring (**5**–**43**) were evaluated for their ability to inhibit cell proliferation *in vitro*. Four tumor cell lines with varying degrees of aggressiveness and resistance to cytostatics (A549, MCF-7, LoVo, LoVo/DX) and one noncancerous cell line (BALB/3T3) were used. For comparison, the starting compounds (**1**–**4**) as well as doxorubicin and cisplatin were also tested. Detailed information concerning biological assay can be found in Section 4. The results are collected in Table 1.

**Table 1:** Antiproliferative Activity (IC_50_) (nM) and the Calculated Values of the Selectivity (SI) and Resistance (RI) Index of Tested Compounds

	A549		MCF-7		LoVo		LoVo/DX			BALB/3T3
compd	IC_50_ (nM)	SI	IC_50_ (nM)	SI	IC_50_ (nM)	SI	IC_50_ (nM)	RI	SI	IC_50_ (nM)
1	35.0 ± 8.0	1.3	17.0 ± 1.3	2.6	20.0 ± 4.7	2.2	1200 ± 440	60	<0.1	44.0 ± 7.0
2	4.4 ± 0.6	1.4	3.5 ± 0.7	1.7	5.4 ± 0.7	1.1	230 ± 17	43	<0.1	6.0 ± 2.3
3	12.0 ± 2.3	1.3	8.1 ± 1.3	2.0	8.9 ± 1.4	1.8	55.0 ± 13.0	6.2	0.3	16.0 ± 2.1
4	1.4 ± 0.5	1.5	1.5 ± 0.3	1.4	1.1 ± 0.5	1.9	1.7 ± 0.6	1.5	1.2	2.1 ± 1.2
5	6.1 ± 1.1	1.8	5.6 ± 1.0	2.0	5.6 ± 0.6	2.0	55.0 ± 20.0	9.8	0.2	11.0 ± 2.2
6	15.0 ± 3.7	1.6	11.0 ± 1.2	2.2	13.0 ± 1.9	1.8	85.0 ± 21.0	6.5	0.3	24.0 ± 9.3
7	6.4 ± 3.9	1.5	5.4 ± 3.7	1.8	3.1 ± 1.3	3.2	23.0 ± 11.0	7.4	0.4	9.9 ± 3.7
8	70.0 ± 23.0	5.7	34.0 ± 16.0	11.8	140 ± 56	2.9	12000 ± 2400	86	<0.1	400 ± 37
9	12.0 ± 2.1	1.2	6.8 ± 1.0	2.1	11.0 ± 5.6	1.3	1500 ± 210	140	<0.1	14.0 ± 2.1
10	13.0 ± 3.3	1.0	8.6 ± 3.2	1.5	12.0 ± 2.4	1.1	95.0 ± 24.0	7.9	0.1	13.0 ± 3.0
11	14.0 ± 2.0	1.3	13.0 ± 1.6	1.4	14.0 ± 6.5	1.3	1700 ± 290	120	<0.1	18.0 ± 7.1
12	37.0 ± 4.2	1.4	13.0 ± 3.4	3.8	19.0 ± 3.9	2.6	2400 ± 550	130	<0.1	50.0 ± 4.5
13	94.0 ± 30.0	1.1	88.0 ± 50.0	1.1	65.0 ± 33.0	1.5	1700 ± 160	26	0.1	100 ± 33
14	13.0 ± 2.6	1.5	10.0 ± 1.8	2.0	10.0 ± 0.8	2.0	27.0 ± 7.6	2.7	0.7	20.0 ± 7.6
15	39.0 ± 2.7	2.4	35.0 ± 14.0	2.6	47.0 ± 22.0	2.0	150 ± 35	3.2	0.6	92.0 ± 20.0
16	35.0 ± 10.0	1.2	28.0 ± 12.0	1.5	27.0 ± 14.0	1.5	210 ± 65	7.8	0.2	41.0 ± 4.0
17	6.9 ± 5.3	1.6	6.1 ± 4.8	1.8	5.7 ± 4.9	1.9	84.0 ± 42.0	15	0.1	11.0 ± 7.8
18	13.0 ± 2.3	1.5	12.0 ± 2.7	1.7	12.0 ± 4.3	1.7	280 ± 77	23	0.1	20.0 ± 13.0
19	17.0 ± 0.8	1.4	11.0 ± 3.7	2.2	13.0 ± 3.5	1.8	220 ± 23	17	0.1	24.0 ± 11.0
20	1300 ± 330	1.6	1200 ± 390	1.8	1200 ± 350	1.8	3400 ± 890	2.8	0.6	2100 ± 750
21	21.0 ± 7.7	1.2	21.0 ± 8.0	1.2	21.0 ± 6.4	1.2	530 ± 110	25	0.1	25.0 ± 5.6
22	72.0 ± 27.0	1.5	93.0 ± 43.0	1.2	97.0 ± 24.0	1.1	580 ± 120	6.0	0.2	110 ±± 15
23	1000 ± 240	1.2	990 ± 82	1.2	860 ± 190	1.4	2900 ± 390	3.4	0.4	1200 ± 14
24	16.0 ± 5.2	3.4	11.0 ± 0.7	4.9	19.0 ± 7.0	2.8	3300 ± 280	170	<0.1	54.0 ± 20.0
25	1.3 ± 0.2	5.2	1.1 ± 0.4	6.1	2.2 ± 1.2	3.0	350 ± 56	160	<0.1	6.7 ± 2.4
26	2.4 ± 0.7	2.7	1.1 ± 0.1	5.8	2.9 ± 1.2	2.2	700 ± 120	240	<0.1	6.4 ± 1.4
27	2.9 ± 0.6	3.0	2.4 ± 0.8	3.6	4.7 ± 1.7	1.9	89.0 ± 18.0	19	0.1	8.7 ± 2.0
28	9.5 ± 2.9	1.3	3.9 ± 1.1	3.1	7.9 ± 3.3	1.5	1000 ± 94	130	<0.1	12.0 ± 1.1
29	12.0 ± 2.4	4.0	12.0 ± 3.2	4.0	14.0 ± 2.3	3.4	2700 ± 600	190	<0.1	48.0 ± 14.0
30	16.0 ± 7.3	18.1	9.3 ± 2.4	31.2	300 ± 94	1.0	7300 ± 1100	24	<0.1	290 ± 49
31	3.3 ± 1.1	3.0	3.0 ± 1.5	3.3	3.1 ± 1.1	3.2	830 ± 220	270	<0.1	9.9 ± 3.0
32	4.4 ± 1.3	5.0	2.7 ± 1.1	8.1	5.0 ± 0.4	4.4	2800 ± 200	560	<0.1	22.0 ± 5.6
33	11.0 ± 0.1	2.5	9.7 ± 3.4	2.8	11.0 ± 5.5	2.5	3000 ± 800	270	<0.1	27.0 ± 8.7
34	26.0 ± 11.0	10.4	10.0 ± 2.1	27.0	33.0 ± 10.0	8.2	17000 ± 1600	510	<0.1	270 ± 67
35	5.7 ± 2.0	2.1	3.8 ± 0.4	3.2	6.3 ± 3.1	1.9	1000 ± 97	160	<0.1	12.0 ± 2.9
36	22.0 ± 7.4	3.4	12.0 ± 1.4	6.2	13.0 ± 3.4	5.7	9100 ± 790	700	<0.1	74.0 ± 22.0
37	7.2 ± 0.7	1.9	4.7 ± 0.5	3.0	8.5 ± 2.3	1.6	1500 ± 240	180	<0.1	14.0 ± 2.4
38	4.8 ± 0.7	2.5	4.2 ± 0.7	2.9	7.7 ± 2.9	1.6	840 ± 95	110	<0.1	12.0 ± 1.6
39	3.3 ± 0.5	2.8	2.6 ± 0.5	3.5	4.3 ± 0.6	2.1	440 ± 64	100	<0.1	9.1 ± 2.6
40	1.2 ± 0.1	3.3	1.2 ± 0.3	3.3	1.7 ± 0.2	2.3	310 ± 61	180	<0.1	3.9 ± 0.2
41	5.4 ± 1.5	2.0	3.2 ± 0.6	3.4	5.1 ± 0.3	2.2	570 ± 120	110	<0.1	11.0 ± 0.8
42	1.9 ± 2.9	1.4	2.5 ± 1.8	1.1	3.8 ± 4.2	0.7	470 ± 300	120	<0.1	2.7 ± 2.4
43	4.1 ± 0.6	2.7	2.8 ± 1.2	3.9	4.7 ± 1.1	2.3	920 ± 150	200	<0.1	11.0 ± 0.4
doxorubicin	120 ± 100	0.1	82 ± 31	0.1	52 ± 13	0.2	5800 ± 2200	110	<0.1	12.0 ± 2.3
cisplatin	3500 ± 630	0.9	4400 ± 1200	0.8	4100 ± 860	0.8	3000 ± 800	0.7	1.1	3300 ± 970

To assess the ability of each compound studied to preferential killing of cancer cells than normal ones (BALB/3T3), the selectivity index (SI) was calculated as the ratio of IC_50_ value for the normal cell line BALB/3T3 to the IC_50_ value for a respective cancer cell line.^55^ Selectivity index is an important parameter in the assessment of activities of new chemotherapeutic agents as it characterizes their therapeutic potential. The favorable SI values should be at least 2 (Table 1).

Resistance indexes (RI) were calculated (ratio of IC_50_ value for LoVo/DX cell line to IC_50_ value for LoVo cell line) for evaluation of the ability of the tested compounds to break the drug resistance of the LoVo/DX line.^56^ The RI values obtained for the studied derivatives are shown in Table 1. According to the RI value, the cells can be classified as: drug-sensitive (RI value in the range 0–2), moderate drug-sensitive (RI value in the range 2–10), and strong drug resistance (RI value above 10).^56^

As we reported earlier^13,14,38,39^ and as confirmed by the results presented here, replacement of an −OMe group with an −NHMe group at position C10 increases the cytotoxicity of native colchicine **1**. In addition, the majority of the 10-*N*-methylaminocolchicines containing the triazole ring showed antiproliferative activity in the nanomolar values against three out of four tumor cell lines tested (Table 1). Except for a few derivatives (e.g., compounds **20** and **23** with a free carboxyl group attached to triazole or compound **13** with hydroxycyclohexyl fragment), the IC_50_ values obtained for the remaining ones (for A549, MCF-7, and LoVo cells) were lower than those observed for the well-known and used chemotherapeutic agents, doxorubicin and cisplatin.

Of the 40 new colchicine derivatives (azide **4** and triazoles **5**–**43**), 32 were more toxic against A549 line than unmodified colchicine **1** and 18 of them were characterized by IC_50_ values lower than 10 nM. Moreover, nine compounds inhibited the proliferation of A549 cells to a greater extent than 10-*N*-methylaminocolchicine **2**, with IC_50_ = 4.4 nM. The most active were azide **4** (IC_50_ = 1.4 nM), hydroxymethyltriazole with trifluorobutanoic acid ester **25** (IC_50_ = 1.3 nM), and two aminomethyltriazole—with isobutyl carbamate **40** and with trichloroethyl carbamate **42** (IC_50_ = 1.2–1.9 nM).

As for the MCF7 line, 32 new derivatives were more cytotoxic than **1** and 22 were characterized by IC_50_ < 10 nM. Eleven compounds were more active than the second starting compound **2**. The most toxic against MCF7 were: azide **4** (IC_50_ = 1.5 nM), two hydroxymethyltriazole—with trifluorobutanoic acid ester **25** and with *tert*-butyloxy-β-alanyl ester **26** (IC_50_ = 1.1 nM), and aminomethyltriazole with isobutyl carbamate **40** (IC_50_ = 1.2 nM).

Against LoVo cells, 30 analogues were more active than the parent colchicine **1**, of which 18 had IC_50_ values lower than 10 nM. In turn, 12 were more toxic than 10-*N*-methylaminocolchicine **2** (IC_50_ = 5.4 nM). Two compounds showed IC_50_ values lower than 2 nM, azide **4** (IC_50_ = 1.1 nM), and the already mentioned aminomethyltriazole with isobutyl carbamate **40** (IC_50_ = 1.7 nM).

The data presented in Table 1 show that unmodified colchicine **1** and the majority of the colchicine derivatives **2**–**43** less effectively inhibited the proliferation of the doxorubicin-resistant subline LoVo/DX than the sensitive LoVo cell line. Twenty-five novel derivatives were more active than **1**, and eight of them were characterized by IC_50_ lower than 100 nM. Eleven new compounds were more cytotoxic against LoVo/DX than amide **2**. Compound **4** had the greatest effect on this resistant cancer cell line (IC_50_ = 1.7 nM).

The IC_50_ value is defined as the concentration of a compound at which 50% growth inhibition is observed. The IC_50_ values shown are mean ± standard deviation (SD). Human lung carcinoma (A549), human breast adenocarcinoma (MCF-7), human colon adenocarcinoma (LoVo), doxorubicin-resistant subline (LoVo/DX), and normal murine embryonic fibroblasts (BALB/3T3).

The results of antiproliferative tests, depending on the diversity of substituents attached to the fourth carbon of triazole of 10-demethoxy-10-*N*-methylaminocolchicine and the cell line tested, are discussed below. Preliminary relationships between the structure and biological activity (SAR) of the new 1,4-disubstituted triazoles containing the colchicine core are also presented.

The most toxic compound against all cells used, both cancerous and normal, was azide **4** (IC_50_ = 1.1–2.1 nM). Its high toxicity is reflected in the low selectivity coefficients of this compound (SI < 2, Table 1). Moreover, its RI of 1.5 is also noteworthy, which means that azide **4** is able to break the drug resistance of the LoVo/DX line. Nevertheless, it is only the starting compound that was used for the synthesis of 39 triazoles. In the obtained series of triazoles, we managed to design the compounds that showed favorable selectivity indexes (SI > 2) in relation to healthy cells. Therefore, in contrast to C7-azide **4**, they have therapeutic potential, which confirms the validity of the study undertaken.

Among the derivatives obtained by the “click chemistry” from 7-azido-10-*N*-methylaminocolchicine **4** and simple, commercially available alkynes (**5**–**17**), the lowest IC_50_ values against A549, MCF-7, and LoVo cells (IC_50_ = 3.1–6.4 nM, Table 1) were observed for analogues **5** (with unsubstituted triazole on C7 carbon) and **7** (with pentyltriazole on C7 carbon). In relation to the LoVo/DX line, the most active were **7** (pentyltriazole) and **14** (phenyltriazole) showing IC_50_ < 30 nM. From triazoles **5**–**17** only aminomethyltriazole **8** showed selectivity toward three out of four cancer cell lines tested, SI > 2 (Table 1, SI = 5.7, 11.8, and 2.9 for A549, MCF-7, and LoVo cell line, respectively). In addition, the IC_50_ values characterizing compound **8** against three tumor cell lines ranged from 30 to 140 nM, making this structure a promising starting point in the search for new anticancer drugs containing the triazole ring.

Derivatives **18**–**23** were synthesized to compare the properties of triazoles substituted only in the fourth position of triazole ring with those of triazoles substituted both in the fourth and fifth positions. For three out of four tested tumor cells, monoesters **18**, **19**, and **21** were 3–5 times more active than diester **22** (IC_50_ in the range 10–25 nM *versus* IC_50_ in the range 70–100 nM). Moreover, *p*-fluorobenzyl ester **21** showed slightly lower antiproliferative activity than methyl and ethyl esters **18** and **19**. The compounds containing free carboxyl groups, **20** and **23**, were characterized by significantly higher IC_50_ values than those of the compounds with esters **18**, **19**, and **21**, and **22** (Table 1). Thus, these results indicate that the monosubstituted triazoles attached to colchicine have a greater effect on cell viability than the doubly substituted triazoles. Unfortunately, only compound **19** (from **18**–**23**) exhibited favorable selectivity coefficients against MCF-7 cells (SI ≥ 2, Table 1).

Another series of compounds obtained were the propargyl ester derivatives **24**–**28**. All of these derivatives were characterized by IC_50_ below 20 nM against A549, MCF-7, and LoVo cells (Table 1). The compound with outstanding activity against the drug-resistant LoVo/DX line was the triazole containing *o*-chlorobenzoic acid ester, **27** (IC_50_ < 100 nM). In addition, most of these derivatives (**24**–**27**) showed good selectivity indexes for two out of the four cell lines tested (SI = 2.7–6.1, Table 1), which means that cancer cells are more susceptible to these compounds than normal ones. Compound **28** showed slightly lower selectivity, SI > 2 only for MCF-7 line. It may be a result of the presence of a nitrogen atom in the benzene ring, and therefore, lower lipophilicity of **28** compared to derivatives **24**–**27**, or some interactions in which this atom can participate in the living cell. The results for 10-*N*-methylaminocolchicine analogues with a triazole ring at position C7 containing a fragment of propargyl ester, **24**–**27**, are noteworthy (low IC_50_ values, SI > 2), and these compounds should be subject to extended and more detailed research determining their anticancer potential.

The next series of analogues designed are triazoles with propargyl amides, **29**–**36**. The most active compounds were **31** (with dichloroacetamide), **32** (with chloropentanamide), and **35** (*o-*chlorobenzamide) characterized with IC_50_ in the range 2.7–6.3 nM for A549, MCF-7, and LoVo cells and IC_50_ ≤ 1000 nM for LoVo/DX line. The remaining amides were also characterized by good antiproliferative properties against three tumor lines (IC_50_ < 35 nM, except the compound **30** against the LoVo cell line, Table 1). The results for these analogues (**29**–**36**) seem very promising because most of the amides showed SI > 2 for three of the tested cell lines (except **30** and **35** for LoVo cells, Table 1). The highest selectivity against cancer cells was obtained for palmitamide **30** (SI = 18.1 for A549 and SI = 31.2 for MCF-7) and amide of Boc-β-alanine **34** (SI = 10.4, 27.0, and 8.2 for A549, MCF-7, and LoVo cell line, respectively). High SI values were also observed for chloropentanamide **32** (SI = 5.0, 8.1, and 4.4) or isonicotamide **36** (SI = 3.4, 6.3, and 5.7) and relatively good—for isobutyramide **29** (SI = 4.0, 4.0, and 3.4) and dichloroacetamide **31** (SI = 3.0, 3.3, and 3.2). So, 10-*N*-methylaminocolchicine derivatives with a heterocyclic triazole ring derived from azide and propargylamine amides are very good starting points for further work aimed at finding an anticancer compound that would be active at a low concentration and would be relatively harmless toward healthy cells in the organism.

Analysis of the biological activity of 7-amides of 10-*N*-methylaminocolchicine described earlier^38^ with 7-triazoles containing a fragment derived from various propargylamine amides characterized above, it can be concluded that most of the values of IC_50_ against A549, MCF7, and LoVo cells for isobutyramide, 4,4,4-trifluorobutyramide, or isonicotamide are comparable (IC_50_ in the range 7.0–22.0 nM) and against LoVo/DX cells, 7-amides were more cytotoxic than the corresponding amides attached to colchicine *via* triazole ring (IC_50_ = 170–950 nM *versus* 2700–9100 nM). In turn, looking at the selectivity of action toward three out four tested cell lines (characterized by SI coefficient), 7-triazole aminomethyl isobutyramide and isonicotamide turned out to be slightly more selective than the corresponding 7-amides (SI = 3.4–6.2 *versus* 1.5–4.8, depending on the cells). 4,4,4-Trifluorobutyramide, on the other hand, has a greater therapeutic potential than the corresponding triazole derivative (SI = 5.6–7.8 *versus* 2.5–2.8). The palmitic acid derivative deserves special attention, the compound with the amide directly on C7 carbon of 10-*N*-methylaminocolchicine does not show selectivity toward A549 and MCF7 cells (SI = 0.9 and 1.2, IC_50_ = 460–620 nM), while that with this amide connected through a triazole ring shows a good selectivity with SI = 18.1 and 31.2 (IC_50_ = 9.3–16.0 nM). The inverse correlation is observed in relation to LoVo cells, 7-palmitamide is characterized by SI = 8.8 (IC_50_ = 62 nM), and this amide linked through a triazole is devoid of selectivity (SI = 1.0, IC_50_ = 300 nM). Therefore, there is no simple relationship between the activity of the compounds with an amide bond on C7 carbon and the same amides located in the chain attached to the C4′ carbon of the triazole at position C7 of 10-*N*-methylaminocolchicine. The introduction of a triazole ring to some derivatives worsens the activity, and to some others improves it. The results also depend on the type of cell line tested.

The last series of compounds analyzed are the propargyl carbamate derivatives **37**–**43**. The most active compound was **40** (with aminomethyltriazoleisobutylcarbamate) with IC_50_ in the range 1.2–1.7 nM for A549, MCF-7, and LoVo cells and IC_50_ = 310 nM for LoVo/DX cell line. The remaining carbamates also efficiently inhibited the proliferation of three tumor lines with IC_50_ = 1.9–8.5 nM (Table 1). The low IC_50_ values are supported with a favorable selectivity index (SI), which is greater than 2 for compounds **39**, **40**, and **43** against A549, MCF-7, and LoVo cells (SI in the range 2.1–3.9, Table 1). These results indicate that the attachment of a carbamate moiety may also improve the biological properties of 1,4-disubstituted triazoles having a 10-*N*-methylaminocolchicine core and should be taken into account in the planning of new structures.

A comparison of the cytotoxicity of 7-carbamates of 10-*N*-methylaminocolchicine^14^ and urethanes attached to the core of the modified colchicine through the aminomethyltriazole described above shows that most of the compounds without a triazole ring (for example, with an ethyl, allyl, 2,2,2-trichloroethyl substituent) show higher activity (IC_50_ = 0.2–11.6 nM *versus* 1.9–840 nM toward all cell lines tested). The inverse relationship is seen for the phenyl substituent, IC_50_ = 2.8–4.7 nM for the compounds with a triazole ring and IC_50_ = 9.1–35.0 nM for those without a triazole ring (toward A549, MCF-7, and LoVo cells). If, on the other hand, the selectivity of action is considered (for ethyl, allyl, 2,2,2-trichloroethyl, phenyl substituent), 7-urethanes directly attached to C7 carbon turn out to be more promising candidates for further development, the insertion of a triazole ring worsened the selectivity of the corresponding carbamates (SI in the range 1.5–21.6 for the compound without a triazole ring and SI in the range 0.7–3.9 for the compounds with a triazole ring).

The data presented in Table 1 show that the studied compounds inhibited the proliferation of the doxorubicin-resistant subline LoVo/DX less effectively than that of the sensitive LoVo cell line. However, as many as 10 of the new colchicine derivatives (**5**–**7**, **10**, **14**–**16**, **20**, **22**, and **23**) were able to break this resistance, RI < 10. It means that replacement of the amide moiety at position C7 by a triazole ring could lead to compounds that will solve one of the main problems of using colchicine in chemotherapy and multidrug resistance (RI values for the starting amides **1** and **2** are 60 and 43, respectively).

The resistance coefficients (RI) determined for the 7-modified 10-*N*-methylaminocolchicine derivatives obtained by us^13,14,38,39^ indicate an interesting relationship. The majority of the compounds containing amide, urethane, urea bond, *etc.* in position C7 were unable to break the drug resistance of the LoVo/DX line (RI > 10); the exception were 9 out of 14 compounds obtained by reductive amination reaction between 7-amino-10-*N*-methylaminocolchicine and the corresponding aldehyde (RI in the range 1.1–9.3).^13^ Together with the results presented in this study, the calculated coefficients of resistance RI indicate that to find compounds based on the 10-*N*-methylaminocolchicine skeleton and capable of breaking drug resistance of the LoVo/DX line, attention should be paid to those that do not have bonds hydrolyzable in the organism. To confirm the emerging thesis, extensive research on a number of more structurally diverse derivatives is necessary.

## 3 Conclusions

In conclusion, we have designed, synthesized, and characterized a series of new doubly modified colchicines with structurally diverse chains on C4′ carbon of triazole. We also assessed their *in vitro* antiproliferative activity against drug-sensitive and drug-resistant tumor lines as well as against normal cells. Among the presented derivatives, the most interesting seem to be compounds **30** and **34** with high SI values and low IC_50_ values. Important compounds, in terms of biological properties, are also compound **8** (aminomethyltriazole) and its amides (**29**, **31**, **33**, **35**, **36**) and carbamates (**37**–**41**, **43**) along with esters of hydroxymethyltriazole (**24**–**27**). The results clearly indicate that the appropriate selection of the side chain on C4′ carbon of 7-triazole-10-*N*-methylaminocolchicine allows obtaining derivatives with increased therapeutic potential compared to that of unmodified colchicine **1** or doxorubicin and cisplatin. The variety of substituents of the mentioned moieties on the triazole ring should be expanded, and for the most promising, *ex vivo* and *in vivo* tests should be performed to confirm their effectiveness. Compounds **30** and **34**, along with the previously selected 7-modified 10-*N*-methylaminocolchicines with promising therapeutic potential,^13,14,38,39^ provide valuable information for the further design of compounds based on the colchicine skeleton that could help in the development of a new drug effective in cancer chemotherapy.

## 4 Experimental Section

### 4.1 General

All solvents, substrates, and reagents were obtained from TriMen Chemicals (Poland) or Sigma-Aldrich and were used without further purification. Spectral grade solvents were stored over 3 Å molecular sieves for several days. Thin-layer chromatography (TLC) analysis was performed using aluminum-backed plates (200 μm thickness, F-254 indicator) from SiliCycle, Inc., and spots were visualized by UV light. Products were purified by flash chromatography using high-purity grade silica gel (pore size 60 Å, 230–400 mesh particle size) from SiliCycle, Inc. Solvents were removed using a rotary evaporator.

### 4.2 Spectroscopic Measurements

NMR spectra were recorded on Bruker Avance DRX 500 (^1^H NMR at 500 MHz and ^13^C NMR at 126 MHz) magnetic resonance spectrometers. ^1^H NMR spectra are reported in chemical shifts downfield from tetramethylsilane (TMS) using the respective residual solvent peak as internal standard (CDCl_3_ δ 7.26 ppm, (CD_3_)_2_SO δ 2.50 ppm). ^1^H NMR spectra are reported as follows: chemical shift (δ, ppm), multiplicity (s = singlet, d = doublet, t = triplet, q = quartet, dd = doublet of doublets, dt = doublet of triplets, dq = doublet of quartets, m = multiplet), coupling constant (*J*) in hertz, and integration. ^13^C NMR spectra are reported in chemical shifts downfield from TMS using the respective residual solvent peak as internal standard (CDCl_3_ δ 77.2 ppm, (CD_3_)_2_SO δ 39.5 ppm).

Electrospray ionization (ESI) mass spectra were obtained on a Waters Alliance 2695 separation module with a PDA 2996 UV detector and a Waters Micromass ZQ 2000 mass detector equipped with a Kinetex Biphenyl 50 × 2.1 mm^2^, 2.6 μm column eluted with 0.3 mL/min flow of 3–100% gradient (over 6 min) of acetonitrile in water (mobile phases contained an addition of 0.04% of formic acid).

Infrared spectra in the mid-infrared region were recorded in KBr tablets on an IFS 113v FT-IR spectrophotometer (Bruker) equipped with a DTGS detector. The resolution of the spectra was 2 cm^–1^, NSS = 64. The Happ–Genzel apodization function was used.

### 4.3 Synthesis

All reactions were performed on a 70 mg (≈0.2 mmol) scale of the starting colchicine derivative, numbered as indicated in the schemes (Schemes 1–5) and in the detailed preparative descriptions below.

Synthesis of 10-*N*-methylaminocolchicine **2** and *N*-deacetyl-10-methylamino-10-demethoxycolchicine **3** was performed according to the previously published procedure.^38,44^

#### 4.3.1 Synthesis of **2**

To a solution of **1** (1.0 equiv) in EtOH, methylamine (solution, 33% in EtOH, 10.0 equiv) was added. The mixture was stirred at reflux for 24 h and then concentrated under reduced pressure to dryness. The residue was purified using column flash chromatography (silica gel; DCM/MeOH) and next lyophilized from dioxane to give the pure product **2** as a yellow solid with a yield of 80%.

ESI-MS for C_22_H_26_N_2_O_5_ (*m*/*z*): [M + H]^+^ 399, [M + Na]^+^ 421, [2M + H]^+^ 797, [2M + Na]^+^ 819, [M – H]^−^ 397, [M + HCOO^–^]^−^ 443.

^1^H NMR (500 MHz, CDCl_3_) δ 8.70 (d, *J* = 6.4 Hz, 1H), 7.58 (s, 1H), 7.46 (d, *J* = 11.1 Hz, 1H), 7.28–7.25 (m, 1H), 6.58 (d, *J* = 11.3 Hz, 1H), 6.52 (s, 1H), 4.73–4.64 (m, 1H), 3.93 (s, 3H), 3.88 (s, 3H), 3.61 (s, 3H), 3.08 (d, *J* = 5.4 Hz, 3H), 2.47–2.43 (m, 1H), 2.37–2.31 (m, 1H), 2.29–2.22 (m, 1H), 2.02–1.96 (m, 1H), 1.94 (s, 3H).

^13^C NMR (126 MHz, CDCl_3_) δ 175.1, 170.2, 155.2, 152.9, 151.6, 151.1, 141.5, 139.4, 134.7, 130.4, 126.9, 122.8, 108.3, 107.2, 61.5, 61.4, 56.2, 52.7, 37.1, 30.1, 29.5, 22.7.

#### 4.3.2 Synthesis of **3**

To a solution of compound **2** (1.0 equiv) in dioxane, 2 M HCl (10.0 equiv) was added and the mixture was stirred at reflux. The reaction progress was monitored by LC-MS. Then, the reaction mixture was neutralized with 4 M NaOH to pH ∼ 10 and extracted four times with EtOAc. The organic layers were combined, washed with brine, dried over Na_2_SO_4_, filtered, and evaporated under reduced pressure. The residue was purified using column flash chromatography (silica gel; DCM/MeOH) and next lyophilized from dioxane to give the pure product **3** as a yellow solid with a yield of 73%.

ESI-MS for C_20_H_24_N_2_O_4_ (*m*/*z*): [M + H]^+^ 357, [M + Na]^+^ 379, [2M + Na]^+^ 735.

^1^H NMR (500 MHz, CDCl_3_) δ 7.61 (s, 1H), 7.33 (d, *J* = 11.1 Hz, 1H), 7.23–7.21 (m, 1H), 6.50 (s, 1H), 6.50 (d, *J* = 11.4 Hz, 1H), 3.87 (s, 3H), 3.87 (s, 3H), 3.75–3.72 (m, 1H), 3.59 (s, 3H), 3.05 (d, *J* = 5.5 Hz, 3H), 2.41–2.37 (m, 1H), 2.33–2.31 (m, 2H), 2.25 (s, 2H), 1.71–1.61 (m, 1H).

^13^C NMR (126 MHz, CDCl_3_) δ 175.6, 155.0, 153.2, 152.8, 150.6, 141.1, 138.7, 135.4, 129.7, 126.6, 123.7, 107.3, 106.9, 61.2, 60.8, 56.1, 54.0, 40.9, 30.7, 29.5.

#### 4.3.3 Synthesis of **4**

To a solution of compound **3** (1.0 eqiuv) in MeOH, K_2_CO_3_ (2.0 equiv) and CuSO_4_·5H_2_O (0.02 equiv) were added, and then the mixture was placed in a water bath. To this mixture, imidazole-1-sulfonyl azide hydrochloride (1.3 equiv) was added portionwise, the reaction was stirred at RT, and its progress was monitored by LC-MS. Then, the reaction mixture was diluted with H_2_O and extracted two times with EtOAc. The organic layers were combined, washed with 10% citric acid and brine, and dried over Na_2_SO_4_. The residue was purified using column flash chromatography (silica gel; EtOAc/hexanes) and next lyophilized from dioxane to give the pure product **4** as a yellow solid with a yield of 58%.

ESI-MS for C_20_H_22_N_4_O_4_ (*m*/*z*): [M + H]^+^ 383, [M + Na]^+^ 405, [2M + Na]^+^ 787.

^1^H NMR (500 MHz, CDCl_3_) δ 7.54 (s, 1H), 7.37–7.32 (m, 2H), 6.53–6.49 (m, 2H), 4.34–4.28 (m, 1H), 3.89 (s, 3H), 3.88 (s, 3H), 3.61 (s, 3H), 3.07 (d, *J* = 5.4 Hz, 3H), 2.50–2.32 (m, 3H), 1.96–1.83 (m, 1H).

^13^C NMR (126 MHz, CDCl_3_) δ 175.5, 155.4, 153.1, 150.7, 147.0, 141.4, 139.1, 134.4, 128.5, 126.2, 124.3, 107.4, 107.2, 63.6, 61.3, 60.9, 56.1, 37.8, 30.0, 29.6.

#### 4.3.4 General Procedure for the Synthesis of Colchicine Derivatives **5**, **8**, **9**, **11**–**19**, and **22**

To a solution of compound **4** (1.0 eqiuv) in MeOH/H_2_O (10/1, v/v) solvent mixture, corresponding alkynes (1.1 eqiuv), CuSO_4_·5H_2_O (0.1 eqiuv), and sodium ascorbate (0.2 eqiuv) were added. The reaction was heated at 55 °C, and its progress was monitored by LC-MS. Then, the reaction mixture was diluted with EtOAc; washed with 5% NaHCO_3_, 0.2 M ethylenediaminetetraacetic acid disodium salt (EDTA-Na_2_), and brine; and dried over Na_2_SO_4_. The residue was purified using column flash chromatography (silica gel; EtOAc/hexanes or EtOAc/MeOH, depending on the substituent) and next lyophilized from dioxane to give the respective compound.

##### 4.3.4.1 Compound **5**

Alkyne: trimethylsilylacetylene.

Yellow solid, yield 33%.

ESI-MS for C_22_H_24_N_4_O_4_ (*m*/*z*): [M + H]^+^ 409, [M + Na]^+^ 431, [2M + H]^+^ 817, [M – H]^−^ 407.

^1^H NMR (500 MHz, CDCl_3_) δ 7.95–7.60 (m, 2H), 7.43 (d, *J* = 11.2 Hz, 1H), 7.30 (s, 1H), 6.59 (s, 1H), 6.54 (d, *J* = 11.2 Hz, 1H), 6.32 (s, 1H), 5.48 (d, *J* = 9.2 Hz, 1H), 3.92 (s, 3H), 3.89 (s, 3H), 3.70 (s, 3H), 3.05 (d, *J* = 5.2 Hz, 3H), 2.90–2.78 (m, 1H), 2.73–2.64 (m, 1H), 2.61–2.52 (m, 2H).

^13^C NMR (126 MHz, CDCl_3_) δ 175.1, 155.5, 153.3, 150.9, 146.8, 141.6, 139.3, 133.9, 128.0, 126.3, 123.3, 107.5, 107.3, 63.1, 61.3, 61.1, 56.1, 36.2, 29.9, 29.5.

##### 4.3.4.2 Compound **8**

Yellow solid, yield 25%.

Alkyne: propargylamine.

ESI-MS for C_23_H_27_N_5_O_4_ (*m*/*z*): [M + H]^+^ 438, [M + Na]^+^ 460, [2M + H]^+^ 875, [2M + Na]^+^ 897, [M – H]^−^ 436, [M + HCOO^–^]^−^ 482.

^1^H NMR (500 MHz, CDCl_3_) δ 8.27 (s, 1H), 7.61 (d, *J* = 4.7 Hz, 1H), 7.39 (d, *J* = 11.3 Hz, 1H), 6.60–6.47 (m, 4H), 6.21 (s, 1H), 5.40 (dd, *J* = 12.3, 4.4 Hz, 1H), 4.22 (s, 2H), 3.87 (s, 3H), 3.85 (s, 3H), 3.64 (s, 3H), 2.97 (d, *J* = 5.1 Hz, 3H), 2.82–2.75 (m, 1H), 2.62–2.53 (m, 1H), 2.51–2.45 (s, 2H).

^13^C NMR (151 MHz, CDCl_3_) δ 174.5, 155.7, 153.4, 150.9, 147.0, 143.1, 141.6, 139.8, 134.1, 128.3, 126.1, 124.9, 122.9, 108.4, 107.4, 63.2, 61.3, 61.2, 56.2, 36.2, 29.9, 29.7, 29.5.

##### 4.3.4.3 Compound **9**

Alkyne: propargyl alcohol.

Yellow solid, yield 57%.

ESI-MS for C_23_H_26_N_4_O_5_ (*m*/*z*): [M + H]^+^ 439, [M + Na]^+^ 461, [2M + H]^+^ 877, [2M + Na]^+^ 899, [M – H]^−^ 437, [M + HCOO^–^]^−^ 483.

^1^H NMR (500 MHz, CDCl_3_) δ 7.70 (s, 1H), 7.43 (d, *J* = 11.2 Hz, 1H), 7.34 (d, *J* = 5.3 Hz, 1H), 6.58 (s, 1H), 6.54 (d, *J* = 11.4 Hz, 1H), 6.31 (s, 1H), 5.43 (dd, *J* = 12.4, 5.0 Hz, 1H), 4.73 (s, 2H), 3.91 (s, 3H), 3.88 (s, 3H), 3.67 (s, 3H), 3.04 (d, *J* = 5.4 Hz, 3H), 2.84–2.76 (m, 1H), 2.68–2.64 (m, 1H), 2.56–2.50 (m, 2H).

^13^C NMR (126 MHz, CDCl_3_) δ 174.8, 155.5, 153.4, 150.9, 148.5, 147.1, 141.6, 139.5, 134.0, 128.2, 126.2, 123.1, 122.6, 107.9, 107.3, 63.1, 61.3, 61.1, 56.5, 56.1, 36.1, 29.9, 29.5.

##### 4.3.4.4 Compound **11**

Alkyne: 3-butin-1-ol.

Yellow solid, yield 66%.

ESI-MS for C_24_H_28_N_4_O_5_ (*m*/*z*): [M + H]^+^ 453, [M + Na]^+^ 475, [2M + H]^+^ 906, [2M + Na]^+^ 928, [M – H]^−^ 451, [M + HCOO^–^]^−^ 497.

^1^H NMR (500 MHz, CDCl_3_) δ 7.52 (s, 1H), 7.42 (d, *J* = 11.2 Hz, 1H), 7.34–7.29 (m, 1H), 6.57 (s, 1H), 6.54 (d, *J* = 11.3 Hz, 1H), 6.35 (s, 1H), 5.40 (d, *J* = 8.4 Hz, 1H), 3.91 (s, 3H), 3.88 (s, 3H), 3.68 (s, 3H), 3.57–3.35 (m, 2H), 3.04 (d, *J* = 5.4 Hz, 3H), 2.91 (s, 2H), 2.85–2.77 (m, 1H), 2.68–2.60 (m, 1H), 2.57–2.48 (m, 2H).

^13^C NMR (126 MHz, CDCl_3_) δ 174.9, 155.5, 153.3, 150.9, 147.2, 141.6, 139.5, 134.0, 128.2, 126.2, 123.2, 107.8, 107.3, 63.0, 61.3, 61.3, 61.1, 56.1, 36.1, 29.9, 29.5, 29.0.

##### 4.3.4.5 Compound **12**

Alkyne: 2-methyl-3-butin-2-ol.

Yellow solid, yield 69%.

ESI-MS for C_25_H_30_N_4_O_5_ (*m*/*z*): [M + H]^+^ 467, [M + Na]^+^ 489, [2M + H]^+^ 933, [2M + Na]^+^ 955, [M – H]^−^ 465, [M + HCOO^–^]^−^ 511.

^1^H NMR (500 MHz, CDCl_3_) δ 7.59 (s, 1H), 7.42 (d, *J* = 11.2 Hz, 1H), 7.31 (d, *J* = 5.4 Hz, 1H), 6.57 (s, 1H), 6.54 (d, *J* = 11.3 Hz, 1H), 6.37 (s, 1H), 5.40 (dd, *J* = 12.2, 4.8 Hz, 1H), 3.91 (s, 3H), 3.88 (s, 3H), 3.66 (s, 3H), 3.04 (d, *J* = 5.4 Hz, 3H), 2.84–2.76 (m, 1H), 2.67–2.62 (m, 1H), 2.56–2.48 (m, 2H), 1.60 (d, *J* = 5.8 Hz, 6H).

^13^C NMR (126 MHz, CDCl_3_) δ 174.9, 155.4, 153.3, 150.9, 147.1, 141.6, 139.4, 134.0, 128.2, 126.2, 123.3, 120.2, 107.8, 107.3, 68.5, 63.1, 61.3, 61.1, 56.1, 36.1, 30.4, 30.3, 29.9, 29.5.

##### 4.3.4.6 Compound **13**

Alkyne: 1-ethynyl-1-cyclohexanol.

Yellow solid, yield 60%.

ESI-MS for C_28_H_34_N_4_O_5_ (*m*/*z*): [M + H]^+^ 507, [M + Na]^+^ 529, [2M + H]^+^ 1013, [2M + Na]^+^ 1035, [M – H]^−^ 505, [M + HCOO^–^]^−^ 551.

^1^H NMR (500 MHz, CDCl_3_) δ 7.56 (s, 1H), 7.42 (d, *J* = 11.2 Hz, 1H), 7.31 (d, *J* = 5.2 Hz, 1H), 6.57 (s, 1H), 6.54 (d, *J* = 11.4 Hz, 1H), 6.37 (s, 1H), 5.40 (dd, *J* = 12.2, 5.0 Hz, 1H), 3.91 (s, 3H), 3.88 (s, 3H), 3.68 (s, 3H), 3.04 (d, *J* = 5.4 Hz, 3H), 2.83–2.78 (m, 1H), 2.68–2.61 (m, 1H), 2.59–2.48 (m, 2H), 2.00–1.92 (m, 2H), 1.90–1.82 (m, 2H), 1.75–1.68 (m, 2H), 1.61–1.47 (m, 3H), 1.35–1.28 (m, 1H).

^13^C NMR (126 MHz, CDCl_3_) δ 174.9, 156.1, 155.4, 153.3, 150.9, 147.1, 141.6, 139.4, 134.0, 128.1, 126.2, 123.3, 120.6, 107.8, 107.3, 69.6, 63.1, 61.3, 61.1, 56.1, 38.1, 38.0, 36.1, 29.9, 29.5, 25.4, 22.0, 21.9.

##### 4.3.4.7 Compound **14**

Alkyne: phenylacetylene.

Yellow solid, yield 64%.

ESI-MS for C_28_H_28_N_4_O_4_ (*m*/*z*): [M + H]^+^ 485, [2M + H]^+^ 969, [M – H]^−^ 483, [M + HCOO^–^]^−^ 529.

^1^H NMR (500 MHz, CDCl_3_) δ 7.84–7.80 (m, 3H), 7.44 (d, *J* = 11.2 Hz, 1H), 7.39 (t, *J* = 7.6 Hz, 2H), 7.33–7.28 (m, 2H), 6.60 (s, 1H), 6.54 (d, *J* = 11.3 Hz, 1H), 6.51 (s, 1H), 5.48 (dd, *J* = 12.2, 5.1 Hz, 1H), 3.95 (s, 3H), 3.91 (s, 3H), 3.74 (s, 3H), 3.06 (d, *J* = 5.4 Hz, 3H), 2.91–2.86 (m, 1H), 2.77–2.68 (m, 1H), 2.66–2.53 (m, 2H).

^13^C NMR (126 MHz, CDCl_3_) δ 175.1, 155.4, 153.4, 150.9, 147.9, 146.8, 141.6, 139.3, 134.0, 130.5, 128.8, 128.2, 127.9, 126.3, 125.8, 123.4, 120.2, 107.4, 107.3, 63.2, 61.3, 61.1, 56.2, 36.2, 30.0, 29.5.

##### 4.3.4.8 Compound **15**

Alkyne: 4-chlorophenylacetylene.

Yellow solid, yield 59%.

ESI-MS for C_28_H_27_ClN_4_O_4_ (*m*/*z*): [M + H]^+^ 519/521, [M + Na]^+^ 541/543, [2M + H]^+^ 1038/1040, [M – H]^−^ 517/519.

^1^H NMR (500 MHz, CDCl_3_) δ 7.83 (s, 1H), 7.73 (d, *J* = 8.6 Hz, 2H), 7.44 (d, *J* = 11.2 Hz, 1H), 7.37–7.32 (m, 2H), 7.30 (d, *J* = 5.5 Hz, 1H), 6.59 (s, 1H), 6.54 (d, *J* = 11.4 Hz, 1H), 6.46 (s, 1H), 5.45 (dd, *J* = 12.3, 5.2 Hz, 1H), 3.93 (s, 3H), 3.89 (s, 3H), 3.73 (s, 3H), 3.04 (d, *J* = 5.4 Hz, 3H), 2.90–2.81 (m, 1H), 2.72–2.65 (m, 1H), 2.61–2.55 (m, 2H).

^13^C NMR (126 MHz, CDCl_3_) δ 175.0, 155.5, 153.4, 150.9, 146.8, 146.7, 141.6, 139.4, 133.9, 133.9, 129.0, 129.0, 127.9, 127.1, 126.2, 123.3, 120.4, 107.5, 107.4, 63.3, 61.3, 61.1, 56.1, 36.2, 29.9, 29.5.

##### 4.3.4.9 Compound **16**

Alkyne: 2-ethynylpyridine.

Yellow solid, yield 81%.

ESI-MS for C_27_H_27_N_5_O_4_ (*m*/*z*): [M + H]^+^ 486, [M + Na]^+^ 508, [2M + H]^+^ 971, [M – H]^−^ 484.

^1^H NMR (500 MHz, CDCl_3_) δ 8.53 (dd, *J* = 4.8, 0.6 Hz, 1H), 8.24 (s, 1H), 8.18 (d, *J* = 7.9 Hz, 1H), 7.76 (td, *J* = 7.8, 1.7 Hz, 1H), 7.44 (d, *J* = 11.2 Hz, 1H), 7.29 (d, *J* = 5.0 Hz, 1H), 7.20 (ddd, *J* = 7.5, 4.9, 1.0 Hz, 1H), 6.59 (s, 1H), 6.52 (d, *J* = 11.4 Hz, 1H), 6.44 (s, 1H), 5.49 (dd, *J* = 12.2, 5.3 Hz, 1H), 3.93 (s, 3H), 3.89 (s, 3H), 3.68 (s, 3H), 3.04 (d, *J* = 5.4 Hz, 3H), 2.90–2.85 (m, 1H), 2.72–2.68 (m, 1H), 2.66–2.55 (m, 2H).

^13^C NMR (126 MHz, CDCl_3_) δ 175.0, 155.4, 153.3, 151.0, 150.1, 149.3, 148.3, 146.8, 141.6, 139.3, 137.0, 133.9, 127.9, 126.3, 123.4, 123.0, 122.7, 120.5, 107.5, 107.4, 63.3, 61.3, 61.1, 56.1, 36.0, 29.9, 29.5.

##### 4.3.4.10 Compound **17**

Alkyne: benzyl propargyl ether.

Yellow solid, yield 69%.

ESI-MS for C_30_H_32_N_4_O_5_ (*m*/*z*): [M + H]^+^ 529, [M + Na]^+^ 551, [2M + H]^+^ 1057, [M – H]^−^ 527.

^1^H NMR (500 MHz, CDCl_3_) δ 7.61 (s, 1H), 7.43 (d, *J* = 11.2 Hz, 1H), 7.36–7.24 (m, 6H), 6.58 (s, 1H), 6.53 (d, *J* = 11.3 Hz, 1H), 6.39 (s, 1H), 5.43 (dd, *J* = 12.5, 5.2 Hz, 1H), 4.71 (d, *J* = 2.1 Hz, 2H), 4.58 (s, 2H), 3.93 (s, 3H), 3.89 (s, 3H), 3.70 (s, 3H), 3.05 (d, *J* = 5.4 Hz, 3H), 2.86–2.76 (m, 1H), 2.70–2.63 (m, 1H), 2.61–2.52 (m, 2H).

^13^C NMR (126 MHz, CDCl_3_) δ 175.0, 155.4, 153.3, 150.9, 146.9, 145.5, 141.6, 139.3, 137.8, 133.9, 128.4, 128.0, 127.9, 127.8, 126.3, 123.4, 123.1, 107.5, 107.3, 63.9, 63.1, 61.3, 61.1, 56.1, 36.2, 29.9, 29.5, 29.4.

##### 4.3.4.11 Compound **18**

Alkyne: methyl propiolate.

Yellow solid, yield 74%.

ESI-MS for C_24_H_26_N_4_O_6_ (*m*/*z*): [M + H]^+^ 467, [M + Na]^+^ 489, [2M + H]^+^ 933, [M – H]^−^ 465.

^1^H NMR (500 MHz, CDCl_3_) δ 8.20 (s, 1H), 7.43 (d, *J* = 11.2 Hz, 1H), 7.32 (d, *J* = 5.3 Hz, 1H), 6.58 (s, 1H), 6.54 (d, *J* = 11.3 Hz, 1H), 6.26 (s, 1H), 5.50 (dd, *J* = 12.5, 4.9 Hz, 1H), 3.92 (s, 3H), 3.91 (s, 3H), 3.88 (s, 3H), 3.70 (s, 3H), 3.05 (d, *J* = 5.4 Hz, 3H), 2.81–2.76 (m, 1H), 2.71–2.67 (m, 1H), 2.57–2.53 (m, 2H).

^13^C NMR (126 MHz, CDCl_3_) δ 174.9, 161.1, 155.5, 153.4, 150.9, 146.0, 141.7, 140.0, 139.5, 133.6, 128.2, 127.7, 126.1, 123.0, 107.5, 107.4, 63.5, 61.2, 61.1, 56.1, 52.2, 36.2, 29.8, 29.5.

##### 4.3.4.12 Compound **19**

Alkyne: ethyl propiolate.

Yellow solid, yield 77%.

ESI-MS for C_25_H_28_N_4_O_6_ (*m*/*z*): [M + H]^+^ 481, [M + Na]^+^ 503, [2M + H]^+^ 961, [2M + Na]^+^ 983, [M – H]^−^ 479, [M + HCOO^–^]^−^ 525.

^1^H NMR (500 MHz, CDCl_3_) δ 8.18 (s, 1H), 7.44 (d, *J* = 11.2 Hz, 1H), 7.35–7.27 (m, 1H), 6.59 (s, 1H), 6.55 (d, *J* = 11.3 Hz, 1H), 6.29 (s, 1H), 5.51 (dd, *J* = 12.6, 5.0 Hz, 1H), 4.42 (q, *J* = 7.1 Hz, 2H), 3.93 (s, 3H), 3.90 (s, 3H), 3.72 (s, 3H), 3.07 (d, *J* = 5.4 Hz, 3H), 2.85–2.78 (m, 1H), 2.74–2.69 (m, 1H), 2.62–2.55 (m, 2H), 1.40 (t, *J* = 7.1 Hz, 3H).

^13^C NMR (126 MHz, CDCl_3_) δ 174.9, 160.7, 155.5, 153.4, 150.9, 146.0, 141.7, 140.4, 139.5, 133.6, 128.1, 127.8, 126.1, 123.0, 107.5, 107.3, 63.4, 61.4, 61.3, 61.1, 56.2, 36.2, 29.8, 29.5, 14.4.

##### 4.3.4.13 Compound **22**

Alkyne: diethyl acetylenedicarboxylate.

Yellow solid, yield 65%.

ESI-MS for C_28_H_32_N_4_O_8_ (*m*/*z*): [M + H]^+^ 553, [M + Na]^+^ 575, [2M + H]^+^ 1105, [M – H]^−^ 551.

^1^H NMR (500 MHz, CDCl_3_) δ 7.45 (d, *J* = 11.2 Hz, 1H), 7.28 (s, 1H), 6.58 (s, 1H), 6.51 (d, *J* = 11.3 Hz, 1H), 6.23 (s, 1H), 5.65 (dd, *J* = 12.2, 5.6 Hz, 1H), 4.38 (qd, *J* = 7.1, 2.2 Hz, 2H), 4.07 (q, *J* = 7.1 Hz, 2H), 3.92 (s, 3H), 3.89 (s, 3H), 3.79 (s, 3H), 3.27–3.20 (m, 1H), 3.04 (d, *J* = 5.4 Hz, 3H), 2.73–2.48 (m, 3H), 1.36 (t, *J* = 7.1 Hz, 3H), 1.07 (t, *J* = 7.1 Hz, 3H).

^13^C NMR (126 MHz, CDCl_3_) δ 174.9, 160.2, 158.4, 155.2, 153.3, 151.0, 146.7, 141.7, 140.6, 139.4, 134.0, 130.4, 128.3, 126.1, 123.0, 107.3, 107.3, 63.4, 62.4, 61.8, 61.1, 61.0, 56.1, 35.7, 30.0, 29.5, 14.2, 13.5.

#### 4.3.5 General Procedure for the Synthesis of Colchicine Derivatives **6** and **7**

To a solution of compound **4** (1.0 eqiuv) in MeOH, the corresponding alkynes (excess), CuI (2.0 eqiuv), and *i*-Pr_2_NEt (2.0 eqiuv) were added. The reaction was heated at 55 °C and its progress was monitored by LC-MS. Then, the reaction mixture was diluted with EtOAc; washed with 5% NaHCO_3_, 0.2 M EDTA-Na_2_, and brine; and dried over Na_2_SO_4_. The residue was purified using column flash chromatography (silica gel; EtOAc/hexanes) and next lyophilized from dioxane to give the respective compound.

##### 4.3.5.1 Compound **6**

Alkyne: 1-pentyne.

Yellow solid, yield 46%.

ESI-MS for C_25_H_30_N_4_O_4_ (*m*/*z*): [M + H]^+^ 451, [M + Na]^+^ 473, [2M + H]^+^ 901, [M – H]^−^ 449, [M + HCOO^–^]^−^ 495.

^1^H NMR (500 MHz, CDCl_3_) δ 7.46–7.27 (m, 3H), 6.58 (s, 1H), 6.54 (d, *J* = 10.9 Hz, 1H), 6.41 (s, 1H), 5.40 (d, *J* = 10.1 Hz, 1H), 3.93 (s, 3H), 3.89 (s, 3H), 3.88–3.79 (m, 2H), 3.71 (s, 3H), 3.06 (d, *J* = 4.0 Hz, 3H), 2.87–2.78 (m, 1H), 2.73–2.66 (m, 2H), 2.58–2.48 (s, 2H), 1.69 (s, 2H), 0.95 (t, *J* = 6.5 Hz, 3H).

^13^C NMR (126 MHz, CDCl_3_) δ 153.3, 150.9, 147.1, 141.6, 139.2, 134.0, 128.0, 126.3, 123.5, 107.4, 107.3, 72.8, 63.0, 61.3, 61.1, 56.1, 36.1, 30.0, 29.7, 29.5, 27.9, 22.6, 13.8.

##### 4.3.5.2 Compound **7**

Alkyne: 1-hexyne.

Yellow solid, yield 54%.

ESI-MS for C_26_H_32_N_4_O_4_ (*m*/*z*): [M + H]^+^ 465, [2M + H]^+^ 929, [2M + Na]^+^ 951, [M – H]^−^ 463, [M + HCOO^–^]^−^ 509.

^1^H NMR (500 MHz, CDCl_3_) δ 7.42 (d, *J* = 11.2 Hz, 1H), 7.33 (s, 1H), 7.32–7.26 (m, 1H), 6.58 (s, 1H), 6.53 (d, *J* = 11.3 Hz, 1H), 6.42 (s, 1H), 5.47–5.30 (m, 1H), 3.93 (s, 3H), 3.89 (s, 3H), 3.71 (s, 3H), 3.06 (d, *J* = 5.3 Hz, 3H), 2.84–2.79 (m, 1H), 2.72–2.68 (m, 2H), 2.67–2.64 (m, 1H), 2.56–2.53 (m, 2H), 1.70–1.58 (m, 2H), 1.37 (dd, *J* = 14.7, 7.3 Hz, 2H), 0.91 (t, *J* = 7.3 Hz, 3H).

^13^C NMR (126 MHz, CDCl_3_) δ 153.3, 150.9, 147.2, 141.6, 139.2, 134.0, 128.0, 126.3, 123.6, 107.4, 107.3, 62.9, 61.3, 61.1, 56.1, 36.1, 31.4, 30.0, 29.5, 25.5, 22.4, 13.9.

#### 4.3.6 Synthesis of **10**

To a solution of compound **9** (1.0 eqiuv) in DCM, Et_3_N (3.0 equiv) was added, and then the mixture was cooled in an ice bath. To this mixture, methanesulfonyl chloride (1.5 equiv) diluted with DCM was added dropwise and next the reaction was stirred at RT, and its progress was monitored by LC-MS. Then, the reaction mixture was diluted with EtOAc, washed with 5% NaHCO_3_ and brine, and dried over Na_2_SO_4_. The residue was purified using column flash chromatography (silica gel; EtOAc/hexanes) and next lyophilized from dioxane to give the pure product **10** as a yellow solid with a yield of 48%.

ESI-MS for C_23_H_25_ClN_4_O_4_ (*m*/*z*): [M + H]^+^ 457/159, [2M + H]^+^ 913/915, [M – H]^−^ 455.

^1^H NMR (500 MHz, CDCl_3_) δ 7.65 (s, 1H), 7.45 (d, *J* = 11.2 Hz, 1H), 7.33–7.28 (m, 1H), 6.65–6.50 (m, 2H), 6.37 (s, 1H), 5.43 (dd, *J* = 12.3, 5.0 Hz, 1H), 4.80–4.68 (m, 2H), 3.94 (s, 3H), 3.91 (s, 3H), 3.73 (s, 3H), 3.08 (d, *J* = 5.3 Hz, 3H), 2.86–2.81 (m, 1H), 2.71–2.66 (m, 1H), 2.64–2.54 (m, 2H).

^13^C NMR (126 MHz, CDCl_3_) δ 155.4, 153.4, 150.9, 146.6, 141.6, 139.4, 133.9, 127.9, 126.2, 123.3, 107.5, 107.3, 63.3, 61.3, 61.1, 56.2, 53.5, 36.3, 36.1, 29.9, 29.7, 29.5.

#### 4.3.7 General Procedure for the Synthesis of Colchicine Derivatives **20** and **23**

To a solution of compound **19** or **22** (1.0 equiv) in EtOH, 1 M NaOH (1.5 equiv for **19** or 3.0 equiv for **22**) was added, and the mixture was stirred at RT. The reaction progress was monitored by LC-MS. Then, the reaction mixture was diluted with DCM and acidified to pH ∼ 2–3 with 2 M HCl. The water layer was washed with DCM again. The organic layers were combined and dried over Na_2_SO_4_, filtered, and evaporated under reduced pressure. The residue was next lyophilized from dioxane to give the respective compound.

##### 4.3.7.1 Compound **20**

Yellow solid, yield 99%.

ESI-MS for C_23_H_24_N_4_O_6_ (*m*/*z*): [M + H]^+^ 453, [M + Na]^+^ 475, [2M + H]^+^ 905, [2M + Na]^+^ 927, [M – H]^−^ 451.

^1^H NMR (500 MHz, CDCl_3_) δ 8.55 (s, 1H), 7.60–7.52 (m, 2H), 6.77–6.69 (m, 2H), 6.59 (s, 1H), 5.59 (dd, *J* = 12.4, 5.4 Hz, 1H), 3.91 (s, 3H), 3.88 (s, 3H), 3.69 (s, 3H), 3.07 (d, *J* = 5.3 Hz, 3H), 2.95–2.84 (m, 1H), 2.69–2.60 (m, 1H), 2.59–2.50 (m, 2H).

^13^C NMR (126 MHz, CDCl_3_) δ 173.5, 162.6, 155.9, 153.6, 150.8, 147.4, 141.6, 140.8, 140.7, 133.9, 129.8, 128.8, 125.7, 122.6, 110.3, 107.5, 63.3, 61.3, 61.2, 56.2, 36.5, 29.8, 29.6.

##### 4.3.7.2 Compound **23**

Yellow solid, yield 99%.

ESI-MS for C_24_H_24_N_4_O_8_ (*m*/*z*): [M + H]^+^ 497, [2M + H]^+^ 993, [M – H]^−^ 495.

^1^H NMR (500 MHz, (CD_3_)_2_SO) δ 7.97 (s, 1H), 7.24 (d, *J* = 11.5 Hz, 1H), 6.63–6.53 (m, 2H), 5.96 (dd, *J* = 12.5, 6.0 Hz, 1H), 5.72 (s, 11H), 3.61 (s, 3H), 3.57 (s, 3H), 3.45 (s, 3H), 2.87 (dd, *J* = 12.4, 7.4 Hz, 1H), 2.74 (d, *J* = 7.4 Hz, 3H), 2.56–252 (m, 1H), 2.18–2.09 (m, 2H).

#### 4.3.8 Synthesis of **21**

To a solution of compound **20** (1.0 eqiuv) in DMF, K_2_CO_3_ (2.0 equiv) was added. To this mixture, 4-fluorobenzyl bromide (1.0 equiv) diluted with DMF was added dropwise. The reaction progress was monitored by LC-MS. Then, the reaction mixture was diluted with H_2_O and washed twice with EtOAc. The organic layers were combined washed with brine and dried over Na_2_SO_4_. The residue was purified using column flash chromatography (silica gel; EtOAc/hexanes) and next lyophilized from dioxane to give the pure product **21** as a yellow solid with a yield of 76%.

ESI-MS for C_30_H_29_N_4_O_6_ (*m*/*z*): [M + H]^+^ 561, [2M + H]^+^ 1121, [M – H]^−^ 559, [M + HCOO^–^]^−^ 605.

^1^H NMR (500 MHz, CDCl_3_) δ 8.17 (s, 1H), 7.48–7.39 (m, 3H), 7.31 (d, *J* = 5.4 Hz, 1H), 7.04 (t, *J* = 8.7 Hz, 2H), 6.58 (s, 1H), 6.54 (d, *J* = 11.3 Hz, 1H), 6.27 (s, 1H), 5.49 (dd, *J* = 12.6, 5.0 Hz, 1H), 5.39–5.29 (m, 2H), 3.92 (s, 3H), 3.89 (s, 3H), 3.69 (s, 3H), 3.06 (d, *J* = 5.4 Hz, 3H), 2.83–2.76 (m, 1H), 2.70–2.63 (m, 1H), 2.59–2.55 (m, 2H).

^13^C NMR (126 MHz, CDCl_3_) δ 174.9, 163.8, 161.8, 160.4, 155.5, 153.5, 150.9, 146.0, 141.7, 139.9, 139.5, 133.6, 130.8, 130.8, 128.3, 127.7, 126.1, 123.0, 115.6, 115.5, 107.5, 107.3, 66.2, 63.5, 61.2, 61.1, 56.1, 36.2, 29.8, 29.5.

#### 4.3.9 General Procedure for the Synthesis of Colchicine Derivatives **24**, **25**, and **28**

To a solution of compound **9** (1.0 equiv) in DCM/DMF (10/1, v/v) solvent mixture, the corresponding carboxylic acid (1.1 equiv), 1-(3-dimethylaminopropyl)-3-ethylcarbodiimide hydrochloride (EDCI, 1.1 equiv), and a catalytic amount of 4-(dimethylamino)pyridine (DMAP) were added. The reaction progress was monitored by LC-MS. Then, the reaction mixture was diluted with EtOAc; washed with H_2_O, 1 M K_2_CO_3_, and brine; and dried over Na_2_SO_4_. The residue was purified using column flash chromatography (silica gel; EtOAc/hexanes for **24** and **25** or EtOAc/MeOH for **28**) and next lyophilized from dioxane to give the respective compound.

##### 4.3.9.1 Compound **24**

Carboxylic acid: palmitic acid.

Yellow solid, yield 44%.

ESI-MS for C_39_H_56_N_4_O_6_ (*m*/*z*): [M + H]^+^ 677, [M + Na]^+^ 699, [M – H]^−^ 675, [M + HCOO^–^]^−^ 721.

^1^H NMR (500 MHz, CDCl_3_) δ 7.66 (s, 1H), 7.42 (d, *J* = 11.1 Hz, 1H), 7.30 (s, 1H), 6.58–6.53 (m, 2H), 6.37 (s, 1H), 5.40 (d, *J* = 9.1 Hz, 1H), 5.20 (s, 2H), 3.92 (s, 3H), 3.89 (s, 3H), 3.71 (s, 3H), 3.05 (d, *J* = 3.5 Hz, 3H), 2.85–2.82 (m, 1H), 2.68–2.65 (m, 1H), 2.56–2.53 (m, 2H), 2.29 (t, *J* = 7.4 Hz, 2H), 1.58–1.55 (m, 2H), 1.25–1.20 (m, 23H), 0.85 (t, *J* = 6.8 Hz, 4H).

^13^C NMR (126 MHz, CDCl_3_) δ 173.8, 153.4, 150.9, 146.6, 141.6, 139.3, 133.9, 127.9, 126.2, 123.4, 107.5, 107.3, 63.2, 61.2, 61.1, 57.6, 56.1, 36.1, 34.1, 31.9, 29.9, 29.7, 29.7, 29.6, 29.5, 29.5, 29.4, 29.3, 29.1, 24.8, 22.7, 14.1.

##### 4.3.9.2 Compound **25**

Carboxylic acid: 4,4,4-trifluorobutyric acid.

Yellow solid, yield 59%.

ESI-MS for C_27_H_29_N_4_O_6_ (*m*/*z*): [M + H]^+^ 563, [M + Na]^+^ 585, [2M + H]^+^ 1125, [2M + Na]^+^ 1147, [M – H]^−^ 561.

^1^H NMR (500 MHz, CDCl_3_) δ 7.64 (s, 1H), 7.42 (d, *J* = 11.2 Hz, 1H), 7.30 (d, *J* = 4.9 Hz, 1H), 6.57 (s, 1H), 6.54 (d, *J* = 11.4 Hz, 1H), 6.33 (s, 1H), 5.40 (dd, *J* = 12.5, 4.8 Hz, 1H), 5.29–5.19 (m, 2H), 3.91 (s, 3H), 3.88 (s, 3H), 3.70 (s, 3H), 3.05 (d, *J* = 5.3 Hz, 3H), 2.87–2.78 (m, 1H), 2.67 (d, *J* = 6.9 Hz, 1H), 2.60–2.53 (m, 4H), 2.47–2.36 (m, 2H).

^13^C NMR (151 MHz, CDCl_3_) δ 175.0, 170.8, 155.4, 153.4, 150.9, 146.6, 141.6, 139.3, 133.8, 127.9, 127.3, 126.1, 125.5, 124.6, 123.2, 107.5, 107.3, 63.2, 61.2, 61.1, 58.2, 56.1, 36.1, 29.8, 29.5, 29.1, 27.0.

##### 4.3.9.3 Compound **28**

Carboxylic acid: isonicotinic acid.

Yellow solid, yield 49%.

ESI-MS for C_29_H_29_N_5_O_6_ (*m*/*z*): [M + H]^+^ 544, [M + Na]^+^ 566, [2M + H]^+^ 1087, [2M + Na]^+^ 1109, [M – H]^−^ 542, [M + HCOO^–^]^−^ 588.

^1^H NMR (500 MHz, CDCl_3_) δ 7.89 (s, 2H), 7.73 (s, 1H), 7.42 (d, *J* = 11.2 Hz, 1H), 7.30 (d, *J* = 5.2 Hz, 1H), 6.56 (s, 1H), 6.53 (d, *J* = 11.3 Hz, 1H), 6.38 (s, 1H), 5.53–5.45 (m, 2H), 5.41 (dd, *J* = 12.5, 4.9 Hz, 1H), 3.91 (s, 3H), 3.87 (s, 3H), 3.71 (s, 3H), 3.05 (d, *J* = 5.4 Hz, 3H), 2.88–2.82 (m, 1H), 2.69–2.65 (m, 1H), 2.60–2.53 (m, 2H).

^13^C NMR (126 MHz, CDCl_3_) δ 175.0, 165.1, 155.5, 153.4, 150.9, 150.4, 146.6, 142.2, 141.6, 139.4, 133.9, 127.9, 126.1, 124.9, 123.3, 107.5, 107.3, 63.3, 61.2, 61.1, 58.8, 56.1, 36.1, 29.9, 29.5.

#### 4.3.10 General Procedure for the Synthesis of Colchicine Derivatives **26** and **27**

Compounds **26** and **27** were prepared in a two-step procedure. The first step involved the synthesis of propargyl ester, and the second step was the reaction of the obtained ester with the azide **4**.

*Synthesis of Ester*: To a solution of *tert*-butyloxy-β-alanine (1.3 equiv) for **26** or 2-chlorobenzoic acid (1.3 equiv) for **27** in DCM, propargyl alcohol (1.3 equiv), 1-(3-dimethylaminopropyl)-3-ethylcarbodiimide hydrochloride (EDCI, 1.3 equiv), and a catalytic amount of 4-(dimethylamino)pyridine (DMAP) were added. The reaction progress was monitored by LC-MS. Then, the reaction mixture was diluted with Et_2_O and washed with H_2_O, 2 M HCl, 1 M K_2_CO_3_, and brine. Et_2_O was evaporated under reduced pressure to give the respective ester, which was used in the next step (without further purification).

*Synthesis of 1,2,3-Triazole*: To a solution of compound **4** (1.0 eqiuv) in MeOH/H_2_O (10/1, v/v) solvent mixture, corresponding to the previously prepared propargyl ester (1.1 eqiuv), CuSO_4_·5H_2_O (0.1 eqiuv) and sodium ascorbate (0.2 eqiuv) were added. The reaction was heated at 55 °C, and its progress was monitored by LC-MS. Then, the reaction mixture was diluted with EtOAc; washed with 5% NaHCO_3_, 0.2 M EDTA-Na_2_, and brine; and dried over Na_2_SO_4_. The residue was purified using column flash chromatography (silica gel; EtOAc/hexanes) and next lyophilized from dioxane to give the respective compound.

##### 4.3.10.1 Compound **26**

Yellow solid, yield 59%.

ESI-MS for C_31_H_39_N_5_O_8_ (*m*/*z*): [M + H]^+^ 610, [M + Na]^+^ 632, [M – H]^−^ 608, [M + HCOO^–^]^−^ 654.

^1^H NMR (500 MHz, CDCl_3_) δ 7.65 (s, 1H), 7.41 (d, *J* = 11.2 Hz, 1H), 7.32–7.27 (m, 1H), 6.56 (s, 1H), 6.52 (d, *J* = 11.3 Hz, 1H), 6.31 (s, 1H), 5.40 (dd, *J* = 12.4, 5.0 Hz, 1H), 5.22 (s, 3H), 3.90 (s, 3H), 3.87 (s, 3H), 3.69 (s, 3H), 3.37–3.33 (m, 2H), 3.04 (d, *J* = 5.4 Hz, 3H), 2.86–2.76 (m, 1H), 2.68–2.62 (m, 1H), 2.58–2.48 (m, 4H), 1.36 (s, 9H).

^13^C NMR (126 MHz, CDCl_3_) δ 175.0, 172.3, 155.9, 155.4, 153.4, 150.9, 146.6, 142.7, 141.6, 139.3, 133.9, 127.9, 126.2, 124.5, 123.3, 107.5, 107.3, 79.2, 72.8, 63.2, 61.2, 61.1, 57.8, 56.1, 36.1, 34.5, 29.9, 29.5, 28.4.

##### 4.3.10.2 Compound **27**

Yellow solid, yield 32%.

ESI-MS for C_30_H_29_ClN_4_O_6_ (*m*/*z*): [M + H]^+^ 577/579, [2M + H]^+^ 1153/1155, [M – H]^−^ 575/577, [M + HCOO^–^]^−^ 621/622.

^1^H NMR (500 MHz, CDCl_3_) δ 7.83 (d, *J* = 7.1 Hz, 1H), 7.75 (s, 1H), 7.46–7.37 (m, 3H), 7.31–7.27 (m, 2H), 6.58 (s, 1H), 6.54 (d, *J* = 11.3 Hz, 1H), 6.39 (s, 1H), 5.52–5.45 (m, 2H), 5.42 (dd, *J* = 12.5, 4.9 Hz, 1H), 3.93 (s, 3H), 3.89 (s, 3H), 3.72 (s, 3H), 3.06 (d, *J* = 5.3 Hz, 3H), 2.91–2.81 (m, 1H), 2.69–2.65 (m, 1H), 2.58–2.54 (m, 2H).

^13^C NMR (126 MHz, CDCl_3_) δ 175.0, 165.4, 155.4, 153.4, 150.9, 146.7, 142.7, 141.6, 139.3, 133.9, 133.9, 132.8, 131.8, 131.1, 129.4, 127.9, 126.7, 126.2, 124.8, 123.4, 107.5, 107.3, 63.3, 61.3, 61.1, 58.7, 56.1, 36.1, 29.9, 29.5.

#### 4.3.11 General Procedure for the Synthesis of Colchicine Derivatives **29**–**36**

Compounds **29**–**36** were prepared in a two-step procedure. The first step involved the synthesis of the amide of propargylamine, and the second step was the reaction of the obtained amide with the azide **4**.

*Synthesis of Amide for **29**, **32**, and **35***: To a solution of propargylamine (1.3 eqiuv) and Et_3_N (2.0 equiv) in DCM, a solution of appropriate acid chloride (1.3 eqiuv) (isobutyryl chloride for **29**, 5-chloropentanoyl chloride for **32**, 2-chlorobenzoyl chloride for **35**) in DCM was added slowly. The reaction progress was monitored by LC-MS. Then, the reaction mixture was diluted with Et_2_O and washed with H_2_O, 2 M HCl, 1 M K_2_CO_3_, and brine. Et_2_O was evaporated under reduced pressure to give the respective amide, which was used in the next step (without further purification).

*Synthesis of Amide for **30**, **31**, **33**, **34**, and **36***: To a solution of the appropriate carboxylic acid (1.3 eqiuv) (palmitic acid for **30**, dichloroacetic acid for **31**, 4,4,4-trifluorobutyric acid for **33**, *tert*-butyloxy-β-alanine for **34**, isonicotinic acid for **36**) in DCM, propargylamine (1.3 equiv), 1-(3-dimethylaminopropyl)-3-ethylcarbodiimide hydrochloride (EDCI, 1.3 equiv), and a catalytic amount of 4-(dimethylamino)pyridine (DMAP) were added. The reaction progress was monitored by LC-MS. Then, the reaction mixture was diluted with Et_2_O and washed with H_2_O, 2 M HCl, 1 M K_2_CO_3_, and brine. Et_2_O was evaporated under reduced pressure to give the respective amide, which was used in the next step (without further purification).

*Synthesis of 1,2,3-Triazole*: To a solution of compound **4** (1.0 eqiuv) in MeOH/H_2_O (10/1, v/v) solvent mixture, corresponding to the previously prepared amide (1.1 eqiuv), CuSO_4_·5H_2_O (0.1 eqiuv) and sodium ascorbate (0.2 eqiuv) were added. The reaction was heated at 55 °C, and its progress was monitored by LC-MS. Then, the reaction mixture was diluted with EtOAc and washed with 5% NaHCO_3_, 0.2 M EDTA-Na_2_, brine, and dried over Na_2_SO_4_. The residue was purified using column flash chromatography (silica gel; EtOAc/MeOH) and next lyophilized from dioxane to give the respective compound.

##### 4.3.11.1 Compound **29**

Yellow solid, yield 58%.

ESI-MS for C_27_H_33_N_5_O_5_ (*m*/*z*): [M + H]^+^ 508, [2M + H]^+^ 1015, [2M + Na]^+^ 1037, [M – H]^−^ 506, [M + HCOO^–^]^−^ 552.

^1^H NMR (500 MHz, CDCl_3_) δ 7.75–7.53 (m, 1H), 7.41 (d, *J* = 11.1 Hz, 1H), 7.32 (s, 1H), 6.72 (s, 1H), 6.60–6.50 (m, 2H), 6.31 (s, 1H), 5.37 (d, *J* = 8.9 Hz, 1H), 4.47 (s, 2H), 3.91 (s, 3H), 3.88 (s, 3H), 3.69 (s, 3H), 3.04 (s, 3H), 2.85–2.77 (s, 1H), 2.67–2.64 (m, 1H), 2.55–2.50 (m, 2H), 2.38–2.34 (s, 1H), 1.09 (t, *J* = 5.2 Hz, 6H).

^13^C NMR (126 MHz, CDCl_3_) δ 177.3, 174.9, 153.4, 150.9, 146.8, 141.6, 139.3, 133.9, 128.0, 126.2, 123.3, 107.6, 107.3, 63.3, 61.3, 61.1, 56.1, 36.0, 35.3, 29.9, 29.5, 19.5.

##### 4.3.11.2 Compound **30**

Yellow solid, yield 59%.

ESI-MS for C_39_H_57_N_5_O_5_ (*m*/*z*): [M + H]^+^ 676, [M + Na]^+^ 698, [M – H]^−^ 674, [M + HCOO^–^]^−^ 720.

^1^H NMR (500 MHz, CDCl_3_) δ 7.56 (s, 1H), 7.43 (d, *J* = 11.2 Hz, 1H), 7.35–7.32 (m, 1H), 6.70 (s, 1H), 6.61–6.50 (m, 2H), 6.34 (s, 1H), 5.36 (dd, *J* = 12.5, 5.1 Hz, 1H), 4.47 (d, *J* = 5.6 Hz, 2H), 3.91 (s, 3H), 3.88 (s, 3H), 3.69 (s, 3H), 3.06 (d, *J* = 5.4 Hz, 3H), 2.89–2.77 (m, 1H), 2.67–2.64 (m, 1H), 2.56–2.50 (m, 2H), 2.19–2.11 (m, 2H), 1.61–1.53 (m, 2H), 1.25–1.15 (m, 23H), 0.85 (t, *J* = 6.9 Hz, 4H).

^13^C NMR (126 MHz, CDCl_3_) δ 174.8, 173.5, 155.4, 153.4, 150.9, 147.0, 145.0, 141.6, 139.4, 133.9, 128.1, 126.1, 123.2, 123.1, 107.7, 107.3, 63.1, 61.2, 61.1, 56.1, 36.5, 36.0, 34.9, 31.9, 29.9, 29.7, 29.7, 29.5, 29.4, 29.3, 25.6, 22.7, 14.1.

##### 4.3.11.3 Compound **31**

Yellow solid, yield 75%.

ESI-MS for C_25_H_27_Cl_2_N_5_O_5_ (*m*/*z*): [M + H]^+^ 548/550, [2M + H]^+^ 1095/1097, [M – H]^−^ 546/548, [M + HCOO^–^]^−^ 592/594.

^1^H NMR (500 MHz, CDCl_3_) δ 8.22 (s, 1H), 7.70–7.62 (m, 1H), 7.43 (d, *J* = 11.2 Hz, 2H), 6.59–6.54 (m, 2H), 6.29 (s, 1H), 6.02 (s, 1H), 5.38 (d, *J* = 8.7 Hz, 1H), 4.56 (s, 2H), 3.91 (s, 3H), 3.88 (s, 3H), 3.69 (s, 3H), 3.05 (d, *J* = 2.7 Hz, 3H), 2.86–2.82 (m, 1H), 2.68–2.64 (m, 1H), 2.53 (s, 2H).

^13^C NMR (126 MHz, CDCl_3_) δ 174.9, 164.5, 153.4, 150.9, 146.8, 141.6, 139.5, 133.9, 128.1, 126.1, 123.1, 107.9, 107.4, 66.4, 63.4, 61.3, 61.1, 56.2, 36.0, 35.7, 29.9, 29.7, 29.5.

##### 4.3.11.4 Compound **32**

Yellow solid, yield 70%.

ESI-MS for C_28_H_34_ClN_5_O_5_ (*m*/*z*): [M + H]^+^ 556/558, [2M + H]^+^ 1112/1114, [M – H]^−^ 554/556, [M + HCOO^–^]^−^ 600/602.

^1^H NMR (500 MHz, CDCl_3_) δ 7.61 (s, 1H), 7.42 (d, *J* = 11.2 Hz, 1H), 7.32 (s, 1H), 7.09 (s, 1H), 6.59–6.51 (m, 2H), 6.31 (s, 1H), 5.35 (d, *J* = 9.2 Hz, 1H), 4.46 (s, 2H), 3.91 (s, 3H), 3.88 (s, 3H), 3.69 (s, 3H), 3.46 (s, 2H), 3.05 (d, *J* = 4.7 Hz, 3H), 2.88–2.79 (m, 1H), 2.67–2.63 (m, 1H), 2.53 (s, 2H), 2.20 (s, 2H), 1.72 (s, 4H).

^13^C NMR (126 MHz, CDCl_3_) δ175.0, 172.8, 153.4, 150.8, 146.9, 141.6, 139.4, 133.9, 128.0, 126.1, 123.2, 107.6, 107.3, 63.3, 61.3, 61.1, 56.1, 44.7, 36.0, 35.3, 34.9, 32.0, 29.7, 29.5, 22.9.

##### 4.3.11.5 Compound **33**

Yellow solid, yield 67%.

ESI-MS for C_27_H_30_F_3_N_5_O_5_ (*m*/*z*): [M + H]^+^ 562, [2M + H]^+^ 1123, [2M + Na]^+^ 1145, [M – H]^−^ 560, [M + HCOO^–^]^−^ 606.

^1^H NMR (500 MHz, CDCl_3_) δ 7.67 (s, 1H), 7.58 (s, 1H), 7.48–7.41 (m, 1H), 7.38–7.35 (m, 1H), 6.61–6.50 (m, 2H), 6.29 (s, 1H), 5.35 (dd, *J* = 12.3, 4.6 Hz, 1H), 4.47 (d, *J* = 3.3 Hz, 2H), 3.91 (s, 3H), 3.88 (s, 3H), 3.68 (s, 3H), 3.05 (d, *J* = 5.3 Hz, 3H), 2.86–2.79 (m, 1H), 2.68–2.63 (m, 1H), 2.54–2.50 (m, 2H), 2.46–2.38 (m, 4H).

^13^C NMR (126 MHz, CDCl_3_) δ 174.8, 170.2, 155.5, 153.4, 150.8, 147.0, 141.6, 139.5, 133.9, 128.1, 128.0, 126.1, 125.8, 123.4, 123.1, 107.9, 107.3, 63.2, 61.2, 61.0, 56.1, 35.9, 34.9, 29.8, 29.5, 29.4, 28.4.

##### 4.3.11.6 Compound **34**

Yellow solid, yield 57%.

ESI-MS for C_31_H_40_N_6_O_7_ (*m*/*z*): [M + H]^+^ 609, [M + Na]^+^ 631, [M – H]^−^ 607, [M + HCOO^–^]^−^ 653.

^1^H NMR (500 MHz, CDCl_3_) δ 7.69 (s, 1H), 7.43 (d, *J* = 11.2 Hz, 1H), 7.36 (s, 1H), 7.07 (s, 1H), 6.58–6.53 (m, 2H), 6.32 (s, 1H), 5.52 (s, 1H), 5.39 (d, *J* = 9.3 Hz, 1H), 4.48 (s, 2H), 3.92 (s, 3H), 3.89 (s, 3H), 3.69 (s, 3H), 3.35 (s, 2H), 3.06 (d, *J* = 4.1 Hz, 3H), 2.86–2.52 (m, 1H), 2.69–2.64 (m, 1H), 2.54 (s, 2H), 2.40 (s, 2H), 1.36 (s, 9H).

^13^C NMR (126 MHz, CDCl_3_) δ 171.9, 156.2, 153.4, 150.9, 146.8, 141.6, 139.4, 133.9, 128.1, 126.1, 123.2, 107.7, 107.3, 79.0, 72.8, 63.3, 61.3, 61.1, 56.1, 36.7, 36.1, 29.9, 29.5, 28.4.

##### 4.3.11.7 Compound **35**

Yellow solid, yield 49%.

ESI-MS for C_30_H_30_ClN_5_O_5_ (*m*/*z*): [M + H]^+^ 576/578, [2M + H]^+^ 1151/1153, [M – H]^−^ 574/576, [M + HCOO^–^]^−^ 620/622.

^1^H NMR (500 MHz, CDCl_3_) δ 7.69 (s, 1H), 7.57 (dd, *J* = 7.5, 1.5 Hz, 1H), 7.43 (d, *J* = 11.2 Hz, 1H), 7.35–7.27 (m, 4H), 7.17–7.14 (m, 1H), 6.58 (s, 1H), 6.53 (d, *J* = 11.3 Hz, 1H), 6.33 (s, 1H), 5.40 (dd, *J* = 12.5, 4.9 Hz, 1H), 4.72 (d, *J* = 5.7 Hz, 2H), 3.92 (s, 3H), 3.89 (s, 3H), 3.69 (s, 3H), 3.05 (d, *J* = 5.4 Hz, 3H), 2.85–2.80 (m, 1H), 2.69–2.65 (m, 1H), 2.60–2.52 (m, 2H).

^13^C NMR (126 MHz, CDCl_3_) δ 175.0, 166.9, 155.4, 153.4, 150.9, 146.9, 144.5, 141.6, 139.3, 134.9, 133.9, 131.3, 130.9, 130.2, 129.9, 128.0, 127.0, 126.2, 123.3, 123.2, 107.5, 107.3, 63.2, 61.3, 61.1, 56.1, 36.0, 35.6, 29.9, 29.5.

##### 4.3.11.8 Compound **36**

Yellow solid, yield 52%.

ESI-MS for C_29_H_30_N_6_O_5_ (*m*/*z*): [M + H]^+^ 543, [2M + H]^+^ 1085, [M – H]^−^ 541, [M + HCOO^–^]^−^ 587.

^1^H NMR (500 MHz, CDCl_3_) δ 8.65–8.56 (m, 3H), 7.72–7.66 (m, 3H), 7.46–7.40 (m, 2H), 6.61–6.50 (m, 2H), 6.34 (s, 1H), 5.35 (dd, *J* = 12.4, 5.0 Hz, 1H), 4.72–4.61 (m, 2H), 3.91 (s, 3H), 3.87 (s, 3H), 3.70 (s, 3H), 3.06 (d, *J* = 5.4 Hz, 3H), 2.88–2.84 (m, 1H), 2.67–2.64 (m, 1H), 2.57–2.47 (m, 2H).

^13^C NMR (126 MHz, CDCl_3_) δ 174.8, 165.7, 155.6, 153.4, 150.8, 150.3, 146.9, 144.7, 141.6, 141.2, 139.6, 133.9, 128.1, 126.0, 123.7, 123.1, 121.4, 107.9, 107.3, 63.3, 61.2, 61.1, 56.1, 36.0, 35.3, 29.9, 29.5.

#### 4.3.12 General Procedure for the Synthesis of Colchicine Derivatives **37**–**40 and 43**

Compounds **37**–**40** and **43** were prepared in a two-step procedure. The first step involved the synthesis of the carbamate of propargylamine, and the second step was the reaction of the obtained carbamate with the azide **4**.

*Synthesis of Carbamate*: To a solution of propargylamine (1.3 equiv) in DCM, Py (2.0 equiv) was added and then the mixture was cooled in an ice bath. To the reaction mixture, an appropriate chloroformate (1.3 equiv) (methyl chloroformate for **37**, ethyl chloroformate for **38**, allyl chloroformate for **39**, isobutyl chloroformate for **40**, phenyl chloroformate for **43**) diluted with DCM was added dropwise. Next, the ice bath was removed and reaction was continued at RT. The reaction progress was monitored by LC-MS. Then, the reaction mixture was diluted with Et_2_O and washed with H_2_O, 2 M HCl, 1 M K_2_CO_3_, and brine. Et_2_O was evaporated under reduced pressure to give the respective carbamate, which was used in the next step (without further purification).

*Synthesis of 1,2,3-Triazole*: To a solution of compound **4** (1.0 eqiuv) in MeOH/H_2_O (10/1, v/v) solvent mixture, corresponding to the previously prepared carbamate (1.1 eqiuv), CuSO_4_·5H_2_O (0.1 eqiuv) and sodium ascorbate (0.2 eqiuv) were added. The reaction was heated at 55 °C, and its progress was monitored by LC-MS. Then, the reaction mixture was diluted with EtOAc and washed with 5% NaHCO_3_, 0.2 M EDTA-Na_2_, brine, and dried over Na_2_SO_4_. The residue was purified using column flash chromatography (silica gel; EtOAc/MeOH) and next lyophilized from dioxane to give the respective compound.

##### 4.3.12.1 Compound **37**

Yellow solid, yield 65%.

ESI-MS for C_25_H_29_N_5_O_6_ (*m*/*z*): [M + H]^+^ 496, [M + Na]^+^ 518, [2M + H]^+^ 991, [2M + Na]^+^ 1013, [M – H]^−^ 494, [M + HCOO^–^]^−^ 540.

^1^H NMR (500 MHz, CDCl_3_) δ 7.56 (s, 1H), 7.41 (d, *J* = 11.2 Hz, 1H), 7.32–7.29 (m, 1H), 6.56 (s, 1H), 6.52 (d, *J* = 11.3 Hz, 1H), 6.33 (s, 1H), 5.64 (s, 1H), 5.41–5.33 (m, 1H), 4.42 (d, *J* = 5.7 Hz, 2H), 3.91 (s, 3H), 3.88 (s, 3H), 3.69 (s, 3H), 3.61 (s, 3H), 3.04 (d, *J* = 5.3 Hz, 3H), 2.84–2.78 (m, 1H), 2.66–2.60 (m, 1H), 2.58–2.45 (m, 2H).

^13^C NMR (126 MHz, CDCl_3_) δ 175.0, 157.2, 155.4, 153.3, 150.9, 146.9, 145.3, 141.6, 139.3, 133.9, 127.9, 126.2, 123.3, 122.8, 107.5, 107.3, 63.1, 61.2, 61.1, 56.1, 52.2, 36.5, 36.0, 29.9, 29.5.

##### 4.3.12.2 Compound **38**

Yellow solid, yield 60%.

ESI-MS for C_26_H_31_N_5_O_6_ (*m*/*z*): [M + H]^+^ 510, [M + Na]^+^ 532, [2M + H]^+^ 1019, [2M + Na]^+^ 1041, [M – H]^−^ 508, [M + HCOO^–^]^−^ 554.

^1^H NMR (500 MHz, CDCl_3_) δ 7.56 (s, 1H), 7.42 (d, *J* = 11.2 Hz, 1H), 7.32–7.28 (m, 1H), 6.57 (s, 1H), 6.53 (d, *J* = 11.3 Hz, 1H), 6.35 (s, 1H), 5.42–5.36 (m, 2H), 4.44 (d, *J* = 5.9 Hz, 2H), 4.12–4.04 (m, 2H), 3.92 (s, 3H), 3.89 (s, 3H), 3.70 (s, 3H), 3.06 (d, *J* = 5.4 Hz, 3H), 2.85–2.80 (m, 1H), 2.68–2.65 (m, 1H), 2.59–2.51 (m, 2H), 1.22–1.16 (m, 3H).

^13^C NMR (126 MHz, CDCl_3_) δ 175.0, 156.7, 155.4, 153.3, 150.9, 146.9, 145.3, 141.6, 139.3, 133.9, 127.9, 126.2, 123.4, 122.7, 107.5, 107.3, 63.1, 61.3, 61.1, 56.1, 36.5, 36.1, 29.9, 29.7, 29.5, 14.6.

##### 4.3.12.3 Compound **39**

Yellow solid, yield 30%.

ESI-MS for C_27_H_31_N_5_O_6_ (*m*/*z*): [M + H]^+^ 522, [M + Na]^+^ 544, [2M + H]^+^ 1043, [2M + Na]^+^ 1065, [M – H]^−^ 520, [M + HCOO^–^]^−^ 566.

^1^H NMR (500 MHz, CDCl_3_) δ 7.57 (s, 1H), 7.43 (d, *J* = 11.2 Hz, 1H), 7.32–7.28 (m, 1H), 6.58 (s, 1H), 6.54 (d, *J* = 11.3 Hz, 1H), 6.36 (s, 1H), 5.93–5.83 (m, 1H), 5.48 (s, 1H), 5.39 (dd, *J* = 12.6, 4.5 Hz, 1H), 5.29 (s, 1H), 5.17 (dd, *J* = 10.5, 1.1 Hz, 1H), 4.54 (d, *J* = 5.2 Hz, 2H), 4.46 (d, *J* = 5.9 Hz, 2H), 3.93 (s, 3H), 3.90 (s, 3H), 3.71 (s, 3H), 3.07 (d, *J* = 5.4 Hz, 3H), 2.89–2.80 (m, 1H), 2.69–2.65 (m, 1H), 2.57–2.53 (m, 2H).

^13^C NMR (126 MHz, CDCl_3_) δ 175.0, 156.3, 155.4, 153.4, 150.9, 146.9, 145.1, 141.6, 139.3, 133.9, 132.8, 127.9, 126.2, 123.4, 122.8, 117.7, 107.5, 107.3, 65.7, 63.2, 61.3, 61.1, 56.2, 36.6, 36.1, 29.9, 29.5.

##### 4.3.12.4 Compound **40**

Yellow solid, yield 32%.

ESI-MS for C_28_H_35_N_5_O_6_ (*m*/*z*): [M + H]^+^ 538, [M + Na]^+^ 560, [2M + H]^+^ 1075, [2M + Na]^+^ 1097, [M – H]^−^ 536, [M + HCOO^–^]^−^ 582.

^1^H NMR (500 MHz, CDCl_3_) δ 7.56 (s, 1H), 7.43 (d, *J* = 11.2 Hz, 1H), 7.32–7.28 (m, 1H), 6.58 (s, 1H), 6.54 (d, *J* = 11.3 Hz, 1H), 6.37 (s, 1H), 5.39 (d, *J* = 7.6 Hz, 2H), 4.45 (d, *J* = 5.8 Hz, 2H), 3.93 (s, 3H), 3.90 (s, 3H), 3.81 (d, *J* = 6.5 Hz, 2H), 3.71 (s, 3H), 3.07 (d, *J* = 5.4 Hz, 3H), 2.86–2.81 (m, 1H), 2.69–2.64 (m, 1H), 2.57–2.53 (m, 2H), 1.90–1.84 (m, 1H), 0.88 (d, *J* = 6.7 Hz, 6H).

^13^C NMR (126 MHz, CDCl_3_) δ 175.0, 156.9, 155.4, 153.4, 150.9, 146.9, 141.6, 139.3, 133.9, 127.9, 126.2, 123.4, 122.7, 107.5, 107.3, 71.2, 63.1, 61.3, 61.1, 56.2, 36.5, 36.1, 29.9, 29.7, 29.5, 28.0, 19.1.

##### 4.3.12.5 Compound **43**

Yellow solid, yield 72%.

ESI-MS for C_30_H_31_N_5_O_6_ (*m*/*z*): [M + H]^+^ 558, [2M + H]^+^ 1115, [M – H]^−^ 556, [M + HCOO^–^]^−^ 602.

^1^H NMR (500 MHz, CDCl_3_) δ 7.62 (s, 1H), 7.43 (d, *J* = 11.2 Hz, 1H), 7.36–7.27 (m, 3H), 7.18–7.14 (m, 1H), 7.12–7.06 (m, 2H), 6.58 (s, 1H), 6.53 (d, *J* = 11.3 Hz, 1H), 6.36 (s, 1H), 6.11 (s, 1H), 5.38 (dd, *J* = 12.5, 4.8 Hz, 1H), 4.51 (d, *J* = 5.8 Hz, 2H), 3.92 (s, 3H), 3.89 (s, 3H), 3.70 (s, 3H), 3.05 (d, *J* = 5.4 Hz, 3H), 2.87–2.80 (m, 1H), 2.68–2.63 (m, 1H), 2.59–2.50 (m, 2H).

^13^C NMR (126 MHz, CDCl_3_) δ 175.0, 155.4, 154.9, 153.4, 151.0, 151.0, 146.9, 144.7, 141.6, 139.3, 133.9, 129.3, 128.0, 126.2, 125.3, 123.3, 123.2, 121.7, 107.5, 107.3, 63.2, 61.3, 61.1, 56.1, 36.7, 36.0, 29.9, 29.5.

#### 4.3.13 Synthesis of **41**

To a solution of compound **8** (1.0 equiv) in DCM, Et_3_N (3.0 equiv) and Boc_2_O (1.1 equiv) were added. The reaction progress was monitored by LC-MS. Then, the reaction mixture was diluted with EtOAc; washed with H_2_O, 5% NaHCO_3_, and brine; and dried over Na_2_SO_4_. The residue was purified using column flash chromatography (silica gel; EtOAc/hexanes) and next lyophilized from dioxane to give the pure product **41** as a yellow solid with a yield of 46%.

ESI-MS for C_28_H_35_N_5_O_6_ (*m*/*z*): [M + H]^+^ 538, [M + Na]^+^ 560, [2M + H]^+^ 1075, [M – H]^−^ 536, [M + HCOO^–^]^−^ 582.

^1^H NMR (500 MHz, CDCl_3_) δ 7.55 (s, 1H), 7.44 (d, *J* = 11.2 Hz, 1H), 7.33–7.27 (m, 1H), 6.58 (s, 1H), 6.54 (d, *J* = 11.3 Hz, 1H), 6.36 (s, 1H), 5.40 (d, *J* = 8.3 Hz, 1H), 5.22 (s, 1H), 4.41 (d, *J* = 5.7 Hz, 2H), 3.93 (s, 3H), 3.90 (s, 3H), 3.71 (s, 3H), 3.07 (d, *J* = 5.4 Hz, 3H), 2.85–2.80 (m, 1H), 2.70–2.65 (m, 1H), 2.58–2.53 (m, 2H), 1.41 (s, 9H).

^13^C NMR (126 MHz, CDCl_3_) δ 175.0, 155.9, 155.4, 153.4, 150.9, 146.9, 145.6, 141.6, 139.3, 133.9, 128.0, 126.2, 123.4, 122.6, 107.5, 107.3, 63.1, 61.3, 61.1, 56.1, 36.2, 36.1, 29.9, 29.5, 28.4.

#### 4.3.14 Synthesis of **42**

To a solution of **8** (1.0 equiv) in DCM, Py (3.0 equiv) was added and then the mixture was cooled in an ice bath. To the reaction mixture, 2,2,2-trichloroethyl chloroformate (1.1 equiv) diluted with DCM was added dropwise. Next, the ice bath was removed and the reaction was continued at RT. The reaction progress was monitored by LC-MS. Then, the reaction mixture was diluted with EtOAc; washed with H_2_O, 5% NaHCO_3_, and brine; and dried over Na_2_SO_4_. The residue was purified using column flash chromatography (silica gel; DCM/MeOH) and next lyophilized from dioxane to give the pure product **42** as a yellow solid with a yield of 37%.

ESI-MS for C_26_H_28_Cl_3_N_5_O_6_ (*m*/*z*): [M + H]^+^ 612/614, [M – H]^−^ 610, [M + HCOO^–^]^−^ 656/658.

^1^H NMR (500 MHz, CDCl_3_) δ 7.59 (s, 1H), 7.44 (d, *J* = 11.2 Hz, 1H), 7.30 (d, *J* = 5.4 Hz, 1H), 6.59 (s, 1H), 6.55 (d, *J* = 11.3 Hz, 1H), 6.35 (s, 1H), 5.81–5.76 (m, 1H), 5.41 (dd, *J* = 12.6, 5.0 Hz, 1H), 4.77–4.67 (m, 2H), 4.53 (d, *J* = 5.8 Hz, 2H), 3.94 (s, 3H), 3.91 (s, 3H), 3.70 (s, 3H), 3.08 (d, *J* = 5.4 Hz, 3H), 2.87–2.79 (m, 1H), 2.70–2.66 (m, 1H), 2.58–2.54 (m, 2H).

^13^C NMR (126 MHz, CDCl_3_) δ 175.0, 155.4, 154.7, 153.4, 150.9, 146.8, 144.5, 141.6, 139.4, 133.9, 128.0, 126.2, 123.3, 122.8, 107.5, 107.3, 74.6, 63.2, 61.3, 61.1, 56.2, 36.8, 36.1, 29.9, 29.7, 29.5.

### 4.4 *In Vitro* Antiproliferative Activity

#### 4.4.1 Cell Lines and Culturing Conditions

Four human cancer cell lines and one murine normal cell line were used to evaluate antiproliferative activity of colchicine and its derivatives **1**–**43**: human lung carcinoma (A549), human breast adenocarcinoma (MCF7), human colon adenocarcinoma cell lines sensitive and resistant to doxorubicin (LoVo) and (LoVo/DX), respectively, and normal murine embryonic fibroblast cell line (BALB/3T3). The A549 and MCF7 cell lines were purchased from the European Collection of Authenticated Cell Cultures (ECACC, Salisbury, U.K., 86012804 and 86012803). The LoVo cell line was purchased from the American Type Culture Collection (CCL-229, ATCC, Manassas, VA), and LoVo/DX by courtesy of Prof. E. Borowski (Technical University of Gdańsk, Gdańsk, Poland). The BALB/3T3 cell line was purchased from the American Type Culture Collection (CCL-163, ATCC, Manassas, VA). All of the cell lines are maintained in the Institute of Immunology and Experimental Therapy (IIET), Wroclaw, Poland.

A549 cells were cultured in a mixture of OptiMEM and Roswell Park Memorial Institute (RPMI) 1640 (1:1) medium (IIET, Wroclaw, Poland), supplemented with 10% fetal bovine serum HyClone (GE Healthcare) and 2 mM l-glutamine (Sigma-Aldrich, Germany). MCF7 cells were cultured in a mixture of Eagle’s medium (IIET, Wroclaw, Poland), supplemented with 10% fetal bovine serum, 2 mM l-glutamine, and insulin (Sigma-Aldrich, Germany). LoVo and LoVo/DX cells were cultured in F12K medium (Corning), supplemented with 10% fetal bovine serum HyClone (GE Healthcare) and 0.1 μg/mL doxorubicin (Accord) for LoVo/DX. BALB/3T3 fibroblasts were cultured in Dulbecco’s medium (Gibco), supplemented with 10% fetal bovine serum (GE Healthcare) and 2 mM L-glutamine (Sigma-Aldrich, Germany). All culture media contained antibiotics: 100 U/mL penicillin (Polfa-Tarchomin, Poland) and 0.1 mg/mL streptomycin (Sigma-Aldrich, Germany). All cell lines were cultured during entire experiment in humid atmosphere at 37 °C and 5% CO_2_.

#### 4.4.2 Cell Viability Assays

Twenty-four hours before adding the tested compounds, all cells were seeded in 384-well plates (Greiner Bio-One, Austria) in appropriate media with (1 × 10^3^)–(2 × 10^3^) cells per well. All cell lines were exposed to each tested agent at different concentrations in the range 100–0.00001 μg/mL for 72 h. The cells were also exposed to the reference drug cisplatin (Teva Pharmaceuticals, Poland) and doxorubicin (Accord Healthcare Limited, U.K.). Additionally, all cell lines were exposed to dimethyl sulfoxide (DMSO) (solvent used for tested compounds) (POCh, Poland) at concentrations corresponding to those present in tested agents dilutions. After 72 h, sulforhodamine B (SRB) assay was performed.^57^

#### 4.4.3 SRB

After 72 h of incubation with the tested compounds, the cells were fixed *in situ* by gently adding cold 50% trichloroacetic acid (TCA) (POCh, Poland) and were incubated at room temperature for 1 h. Then, the wells were washed four times with water and air-dried. Next, 0.1% solution of sulforhodamine B (Sigma-Aldrich, Germany) in 1% acetic acid (POCh, Poland) was added to each well and the plates were incubated at room temperature for 0.5 h. After incubation time, unbound dye was removed by washing plates four times with 1% acetic acid, whereas stain bound to cells was solubilized with 10 mM Tris base (Sigma-Aldrich, Germany). Absorbance of each solution was read at Synergy H4 Hybrid Multi-Mode Microplate Reader (BioTek Instruments) at the 540 nm wavelength.

Results are presented as mean IC_50_ (concentration of the tested compound that inhibits cell proliferation by 50%) ± standard deviation. IC_50_ values were calculated in in Excel sheet using formulas adapted by Mateusz Psurski fromCheburator 0.4, Dmitry Nevozhay software (version 1.2.0 software by Dmitry Nevozhay, 2004–2014, http://www.cheburator.nevozhay.com, freely available) for each experiment.^58^ Compounds at each concentration were tested in triplicate in a single experiment, and each experiment was repeated at least three times independently.

### 4.5 *In Silico* Calculation of Physicochemical Properties

We used the Molinspiration online database (http://www.molinspiration.com, free of charge) to predict the physicochemical properties of all presented compounds.^49^

## Supporting Information Available

Characteristic chemical shifts in the ^1^H and ^13^C NMR spectra of colchicine **1**, starting compounds **2** and **3**, azide **4**, and 1,2,3-triazoles **5**–**43**; selected physicochemical properties (drug-likeness filters) of colchicine (**1**) and its derivatives (**2**–**43**); LC-MS chromatograms and mass spectra, ^1^H NMR and ^13^C NMR spectra of compounds **2**–**43**; and FT-IR spectra of compounds **2**–**4**, **9**, and propargyl alcohol (PDF)
