# Supplementary material for: Novel Double-Modified Colchicine Derivatives Bearing 1,2,3-Triazole: Design, Synthesis, and Biological Activity Evaluation
Source: ACS Omega. 2021 Sep 28;6(40):26583–600. doi: 10.1021/acsomega.1c03948 (PMC8515607; doi:10.1021/acsomega.1c03948)
Supplement: Supplementary file 1 — ao1c03948_si_001.pdf [file ao1c03948_si_001.pdf]

Novel double-modified colchicine derivatives bearing 1,2,3-triazole: design, synthesis and biological activity evaluation

Julia Krzywik [1,2], Anna Nasulewicz-Goldeman [3], Witold Mozga [2], Joanna Wietrzyk [3], Adam Huczyński [1,*]

[1] *Department of Medical Chemistry, Faculty of Chemistry, Adam Mickiewicz University, Uniwersytetu Poznańskiego 8, 61-614 Poznań, Poland; julia.krzywik@amu.edu.pl (J.K.), adhucz@amu.edu.pl (A.H.)*
[2] *TriMen Chemicals, Piłsudskiego 141, 92-318 Łódź, Poland; mozga@trimen.pl (W.M.)*
[3] *Hirszfeld Institute of Immunology and Experimental Therapy, Polish Academy of Sciences, Rudolfa Weigla 12, 53-114 Wrocław, Poland; anna.nasulewicz-goldeman@hirszfeld.pl(A.N.-G.), joanna.wietrzyk@hirszfeld.pl (J.W.)*

Supplementary Materials

Table S1. Characteristic chemical shifts in the ^{1}H and ^{13}C NMR spectra of colchicine **1**, starting compounds **2-3**, azide **4** and 1,2,3-triazoles **5-43**.

Table S2. Selected physicochemical properties (drug-likeness filters) of colchicine (**1**) and its derivatives (**2–43**).

LC-MS chromatograms and mass spectra, ^{1}H NMR and ^{13}C NMR spectra of compounds 2-43.

Figure S1. The LC-MS chromatogram and mass spectra of **2**.
Figure S2. The ^{1}H NMR spectrum of **2** in $CDCl_3$.
Figure S3. The ^{13}C NMR spectrum of **2** in $CDCl_3$.
Figure S4. The LC-MS chromatogram and mass spectra of **3**.
Figure S5. The ^{1}H NMR spectrum of **3** in $CDCl_3$.
Figure S6. The ^{13}C NMR spectrum of **3** in $CDCl_3$.
Figure S7. The LC-MS chromatogram and mass spectra of **4**.
Figure S8. The ^{1}H NMR spectrum of **4** in $CDCl_3$.

Figure S9. The ^{13}C NMR spectrum of **4** in $CDCl_3$.
Figure S10. The LC-MS chromatogram and mass spectra of **5**.
Figure S11. The ^{1}H NMR spectrum of **5** in $CDCl_3$.
Figure S12. The ^{13}C NMR spectrum of **5** in $CDCl_3$.
Figure S13. The LC-MS chromatogram and mass spectra of **6**.
Figure S14. The ^{1}H NMR spectrum of **6** in $CDCl_3$.
Figure S15. The ^{13}C NMR spectrum of **6** in $CDCl_3$.
Figure S16. The LC-MS chromatogram and mass spectra of **7**.
Figure S17. The ^{1}H NMR spectrum of **7** in $CDCl_3$.
Figure S18. The ^{13}C NMR spectrum of **7** in $CDCl_3$.
Figure S19. The LC-MS chromatogram and mass spectra of **8**.
Figure S20. The ^{1}H NMR spectrum of **8** in $CDCl_3$.
Figure S21. The ^{13}C NMR spectrum of **8** in $CDCl_3$.
Figure S22. The LC-MS chromatogram and mass spectra of **9**.
Figure S23. The ^{1}H NMR spectrum of **9** in $CDCl_3$.
Figure S24. The ^{13}C NMR spectrum of **9** in $CDCl_3$.
Figure S25. The LC-MS chromatogram and mass spectra of **10**.
Figure S26. The ^{1}H NMR spectrum of **10** in $CDCl_3$.
Figure S27. The ^{13}C NMR spectrum of **10** in $CDCl_3$.
Figure S28. The LC-MS chromatogram and mass spectra of **11**.
Figure S29. The ^{1}H NMR spectrum of **11** in $CDCl_3$.
Figure S30. The ^{13}C NMR spectrum of **11** in $CDCl_3$.
Figure S31. The LC-MS chromatogram and mass spectra of **12**.
Figure S32. The ^{1}H NMR spectrum of **12** in $CDCl_3$.
Figure S33. The ^{13}C NMR spectrum of **12** in $CDCl_3$.
Figure S34. The LC-MS chromatogram and mass spectra of **13**.
Figure S35. The ^{1}H NMR spectrum of **13** in $CDCl_3$.
Figure S36. The ^{13}C NMR spectrum of **13** in $CDCl_3$.
Figure S37. The LC-MS chromatogram and mass spectra of **14**.
Figure S38. The ^{1}H NMR spectrum of **14** in $CDCl_3$.
Figure S39. The ^{13}C NMR spectrum of **14** in $CDCl_3$.
Figure S40. The LC-MS chromatogram and mass spectra of **15**.
Figure S41. The ^{1}H NMR spectrum of **15** in $CDCl_3$.
Figure S42. The ^{13}C NMR spectrum of **15** in $CDCl_3$.
Figure S43. The LC-MS chromatogram and mass spectra of **16**.
Figure S44. The ^{1}H NMR spectrum **16** in $CDCl_3$.
Figure S45. The ^{13}C NMR spectrum of **16** in $CDCl_3$.
Figure S46. The LC-MS chromatogram and mass spectra of **17**.
Figure S47. The ^{1}H NMR spectrum of **17** in $CDCl_3$.
Figure S48. The ^{13}C NMR spectrum of **17** in $CDCl_3$.
Figure S49. The LC-MS chromatogram and mass spectra of **18**.
Figure S50. The ^{1}H NMR spectrum of **18** in $CDCl_3$.
Figure S51. The ^{13}C NMR spectrum of **18** in $CDCl_3$.
Figure S52. The LC-MS chromatogram and mass spectra of **19**.
Figure S53. The ^{1}H NMR spectrum of **19** in $CDCl_3$.
Figure S54. The ^{13}C NMR spectrum of **19** in $CDCl_3$.
Figure S55. The LC-MS chromatogram and mass spectra of **20**.
Figure S56. The ^{1}H NMR spectrum of **20** in $CDCl_3$.
Figure S57. The ^{13}C NMR spectrum of **20** in $CDCl_3$.
Figure S58. The LC-MS chromatogram and mass spectra of **21**.

Figure S59. The ^{1}H NMR spectrum of **21** in $CDCl_3$.
Figure S60. The ^{13}C NMR spectrum of **21** in $CDCl_3$.
Figure S61. The LC-MS chromatogram and mass spectra of **22**.
Figure S62. The ^{1}H NMR spectrum of **22** in $CDCl_3$.
Figure S63. The ^{13}C NMR spectrum of **22** in $CDCl_3$.
Figure S64. The LC-MS chromatogram and mass spectra of **23**.
Figure S65. The ^{1}H NMR spectrum of **23** in $(CD_3)_2SO$.
Figure S66. The LC-MS chromatogram and mass spectra of **24**.
Figure S67. The ^{1}H NMR spectrum of **24** in $CDCl_3$.
Figure S68. The ^{13}C NMR spectrum of **24** in $CDCl_3$.
Figure S69. The LC-MS chromatogram and mass spectra of **25**.
Figure S70. The ^{1}H NMR spectrum of **25** in $CDCl_3$.
Figure S71. The ^{13}C NMR spectrum of **25** in $CDCl_3$.
Figure S72. The LC-MS chromatogram and mass spectra of **26**.
Figure S73. The ^{1}H NMR spectrum of **26** in $CDCl_3$.
Figure S74. The ^{13}C NMR spectrum of **26** in $CDCl_3$.
Figure S75. The LC-MS chromatogram and mass spectra of **27**.
Figure S76. The ^{1}H NMR spectrum of **27** in $CDCl_3$.
Figure S77. The ^{13}C NMR spectrum of **27** in $CDCl_3$.
Figure S78. The LC-MS chromatogram and mass spectra of **28**.
Figure S79. The ^{1}H NMR spectrum of **28** in $CDCl_3$.
Figure S80. The ^{13}C NMR spectrum of **28** in $CDCl_3$.
Figure S81. The LC-MS chromatogram and mass spectra of **29**.
Figure S82. The ^{1}H NMR spectrum of **29** in $CDCl_3$.
Figure S83. The ^{13}C NMR spectrum of **29** in $CDCl_3$.
Figure S84. The LC-MS chromatogram and mass spectra of **30**.
Figure S85. The ^{1}H NMR spectrum of **30** in $CDCl_3$.
Figure S86. The ^{13}C NMR spectrum of **30** in $CDCl_3$.
Figure S87. The LC-MS chromatogram and mass spectra of **31**.
Figure S88. The ^{1}H NMR spectrum of **31** in $CDCl_3$.
Figure S89. The ^{13}C NMR spectrum of **31** in $CDCl_3$.
Figure S90. The LC-MS chromatogram and mass spectra of **32**.
Figure S91. The ^{1}H NMR spectrum of **32** in $CDCl_3$.
Figure S92. The ^{13}C NMR spectrum of **32** in $CDCl_3$.
Figure S93. The LC-MS chromatogram and mass spectra of **33**.
Figure S94. The ^{1}H NMR spectrum of **33** in $CDCl_3$.
Figure S95. The ^{13}C NMR spectrum of **33** in $CDCl_3$.
Figure S96. The LC-MS chromatogram and mass spectra of **34**.
Figure S97. The ^{1}H NMR spectrum of **34** in $CDCl_3$.
Figure S98. The ^{13}C NMR spectrum of **34** in $CDCl_3$.
Figure S99. The LC-MS chromatogram and mass spectra of **35**.
Figure S100. The ^{1}H NMR spectrum of **35** in $CDCl_3$.
Figure S101. The ^{13}C NMR spectrum of **35** in $CDCl_3$.
Figure S102. The LC-MS chromatogram and mass spectra of **36**.
Figure S103. The ^{1}H NMR spectrum of **36** in $CDCl_3$.
Figure S104. The ^{13}C NMR spectrum of **36** in $CDCl_3$.
Figure S105. The LC-MS chromatogram and mass spectra of **37**.
Figure S106. The ^{1}H NMR spectrum of **37** in $CDCl_3$.
Figure S107. The ^{13}C NMR spectrum of **37** in $CDCl_3$.
Figure S108. The LC-MS chromatogram and mass spectra of **38**.

Figure S109. The ^{1}H NMR spectrum of **38** in $CDCl_3$.
Figure S110. The ^{13}C NMR spectrum of **38** in $CDCl_3$.
Figure S111. The LC-MS chromatogram and mass spectra of **39**.
Figure S112. The ^{1}H NMR spectrum of **39** in $CDCl_3$.
Figure S113. The ^{13}C NMR spectrum of **39** in $CDCl_3$.
Figure S114. The LC-MS chromatogram and mass spectra of **40**.
Figure S115. The ^{1}H NMR spectrum of **40** in $CDCl_3$.
Figure S116. The ^{13}C NMR spectrum of **40** in $CDCl_3$.
Figure S117. The LC-MS chromatogram and mass spectra of **41**.
Figure S118. The ^{1}H NMR spectrum of **41** in $CDCl_3$.
Figure S119. The ^{13}C NMR spectrum of **41** in $CDCl_3$.
Figure S120. The LC-MS chromatogram and mass spectra of **42**.
Figure S121. The ^{1}H NMR spectrum of **42** in $CDCl_3$.
Figure S122. The ^{13}C NMR spectrum of **42** in $CDCl_3$.
Figure S123. The LC-MS chromatogram and mass spectra of **43**.
Figure S124. The ^{1}H NMR spectrum of **43** in $CDCl_3$.
Figure S125. The ^{13}C NMR spectrum of **43** in $CDCl_3$.

FT-IR spectra of compounds 2-4, 9 and propargyl alcohol.

Figure S126.FT-IR spectra of (—) **2**, (- - -) **3** and (- • -) **4** in the ranges of: 4000–400cm^{-1} and 2500-1000cm^{-1}.
Figure S127. FT-IR spectra of (- • -) **4,** (- - -) **propargyl alcohol** (—) **9**, and in the ranges of : 4000–400cm^{-1}.

Table S1. Characteristic chemical shifts in the ^{1}H and ^{13}C NMR spectra of colchicine **1**, starting compounds **2-3**, azide **4** and 1,2,3-triazoles **5-43**.

	Atoms of colchicine and its derivatives						
**Compound**	**H7** [ppm]	**H8** [ppm]	**H5'** [ppm]	**-NH-** in amide or carbamate moiety [ppm]	**C7** [ppm]	**C5'** and **C4'** [ppm]	**-C=O** in ester or amide or carbamate moiety [ppm]
**1-2**	4.53-4.73	7.58-7.62	-	8.58-8.70	52.7-52.8	-	170.2-170.3
**3**	3.72-3.75	7.60	-	-	54.0	-	-
**4**	4.28-4.34	7.54	-	-	63.6	-	-
**5-17**	5.40-5.49	6.21-6.46	7.40-8.27	-	62.9-63.3	120.2-122.9<br>143.1-146.0	-
**18-23**	5.49-5.96	6.23-6.27	8.17-8.20	-	63.3-63.5	122.6-123.0<br>140.0-140.7	158.4-161.8
**24-28**	5.40-5.42	6.31-6.39	7.64-7.75	-	63.2-63.3	124.8-124.9<br>142.2-142.8	165.1-172.3
**29-36**	5.35-5.39	6.28-6.34	7.56-7.70	6.70-8.20	63.2-63.4	123.2-123.4<br>144.5-145.0	164.5-175.0
**37-43**	5.38-5.48	6.33-6.38	7.55-7.62	5.22-6.10	63.1-63.2	122.6-123.2<br>144.5-146.0	154.7-157.2

The labelling of the atoms is the same as in Figure 2 and Schemes 1-5.

Table S2. Selected physicochemical properties (drug-likeness filters) of colchicine (**1**) and its derivatives (**2–43**).

Compound	Lipinski rule					Veber rule	
	MW ≤ 500	clog*P* ≤ 5	NHD ≤ 5	NHA ≤ 10	NV	NBR ≤ 10	TPSA ≤ 140
**1**	399.4	1.1	1	7	0	5	83.1
**2**	398.5	1.6	2	7	0	5	85.9
**3**	356.4	0.9	3	6	0	4	82.8
**4**	382.4	4.1	1	8	0	5	106.6
**5**	408.5	2.9	1	8	0	5	87.5
**6**	450.5	4.2	1	8	0	7	87.5
**7**	464.6	4.7	1	8	0	8	87.5
**8**	437.5	2.3	3	9	0	6	113.5
**9**	438.5	2.5	2	9	0	6	107.8
**10**	456.9	3.7	1	8	0	6	87.5
**11**	452.5	2.7	2	9	0	7	107.8
**12**	466.5	3.3	2	9	0	6	107.8
**13**	466.5	3.3	2	9	1	6	107.8
**14**	484.6	4.6	1	8	0	6	87.5
**15**	519.0	5.2	1	8	2	6	87.5
**16**	485.5	3.4	1	9	0	6	100.4
**17**	528.6	4.7	1	9	1	9	96.8
**18**	466.5	3.0	1	10	0	7	113.8
**19**	480.5	3.3	1	10	0	8	113.8
**20**	425.5	2.7	2	10	0	6	124.8
**21**	560.6	4.7	1	10	1	9	113.8
**22**	552.6	3.8	1	12	2	11	140.1
**23**	496.5	2.5	3	12	1	7	162.1
**24**	676.9	9.2	1	10	2	22	113.8
**25**	562.5	4.1	1	10	1	11	113.8
**26**	609.7	3.7	2	13	2	13	152.2
**27**	577.0	5.2	1	10	2	9	113.8
**28**	543.6	3.6	1	11	2	9	126.7
**29**	507.6	3.1	2	10	1	8	116.6
**30**	675.9	8.9	2	10	2	21	116.6
**31**	548.4	3.2	2	10	1	8	116.6
**32**	556.1	3.7	2	10	1	11	116.6
**33**	561.6	3.5	2	10	1	10	116.6
**34**	608.7	3.1	3	13	2	12	155.0
**35**	576.1	4.4	2	10	1	8	116.6
**36**	542.6	2.4	2	11	2	8	129.5
**37**	495.5	2.5	2	11	1	8	125.9
**38**	509.6	3.1	2	11	2	9	125.9
**39**	521.6	3.4	2	11	2	10	125.9
**40**	537.6	3.9	2	11	2	10	125.9
**41**	537.6	3.7	2	11	2	9	125.9

**42**	612.9	4.6	2	11	2	10	125.9
**43**	557.6	3.9	2	11	2	9	125.9

MW: Molecular weight; clog*P*: calculated octanol/water partition coefficient; NHD: number of hydrogen bonds donors (OH and NH groups); NHB: number of hydrogen bonds acceptors (O and N atoms); NV: number of violations of rule; NBR: number of rotatable bonds; TPSA: topological polar surface area.

LC-MS chromatograms and mass spectra, ^{1}H NMR and ^{13}C NMR spectra of compounds 2-43.

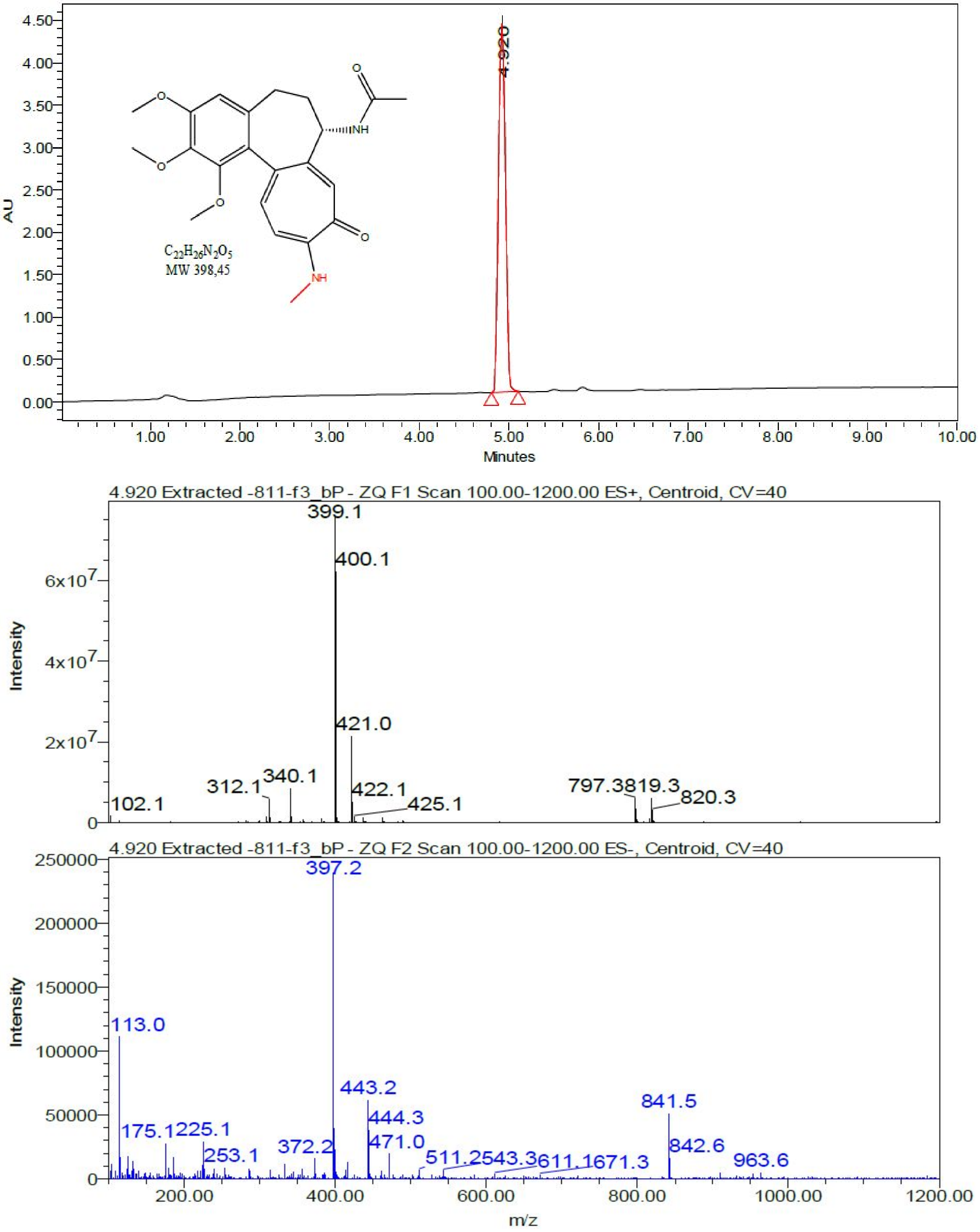

Figure S1. The LC-MS chromatogram and mass spectra of **2**.

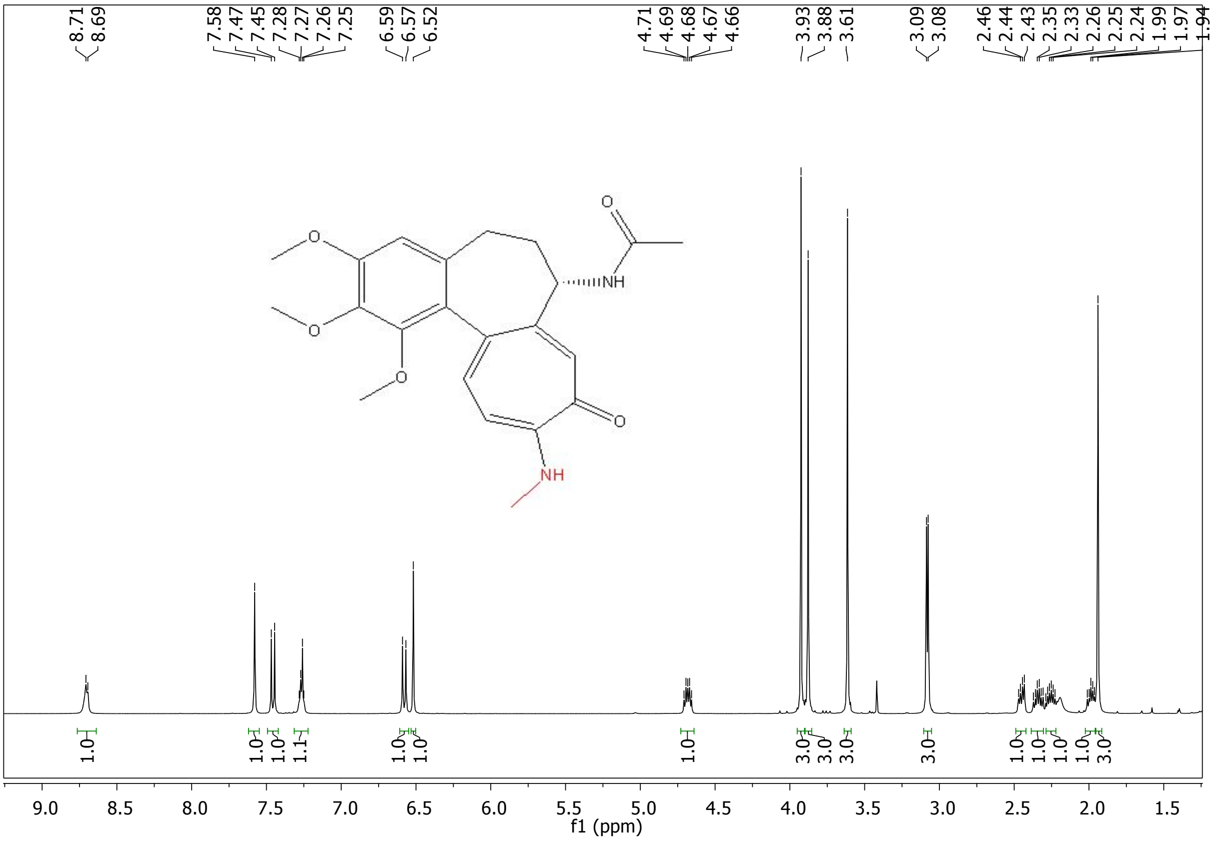

Figure S2. The ^{1}H NMR spectrum of **2** in $CDCl_3$.

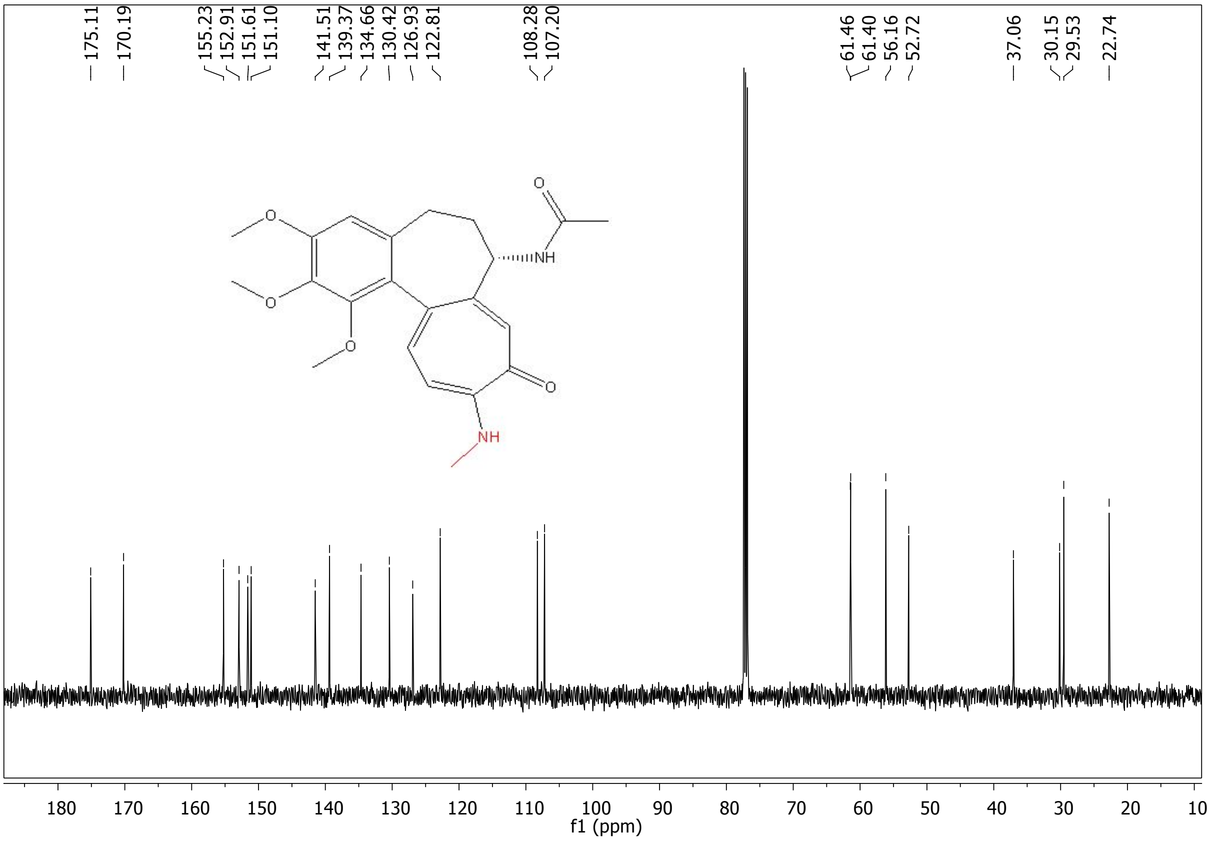

Figure S3. The ^{13}C NMR spectrum of **2** in $CDCl_3$.

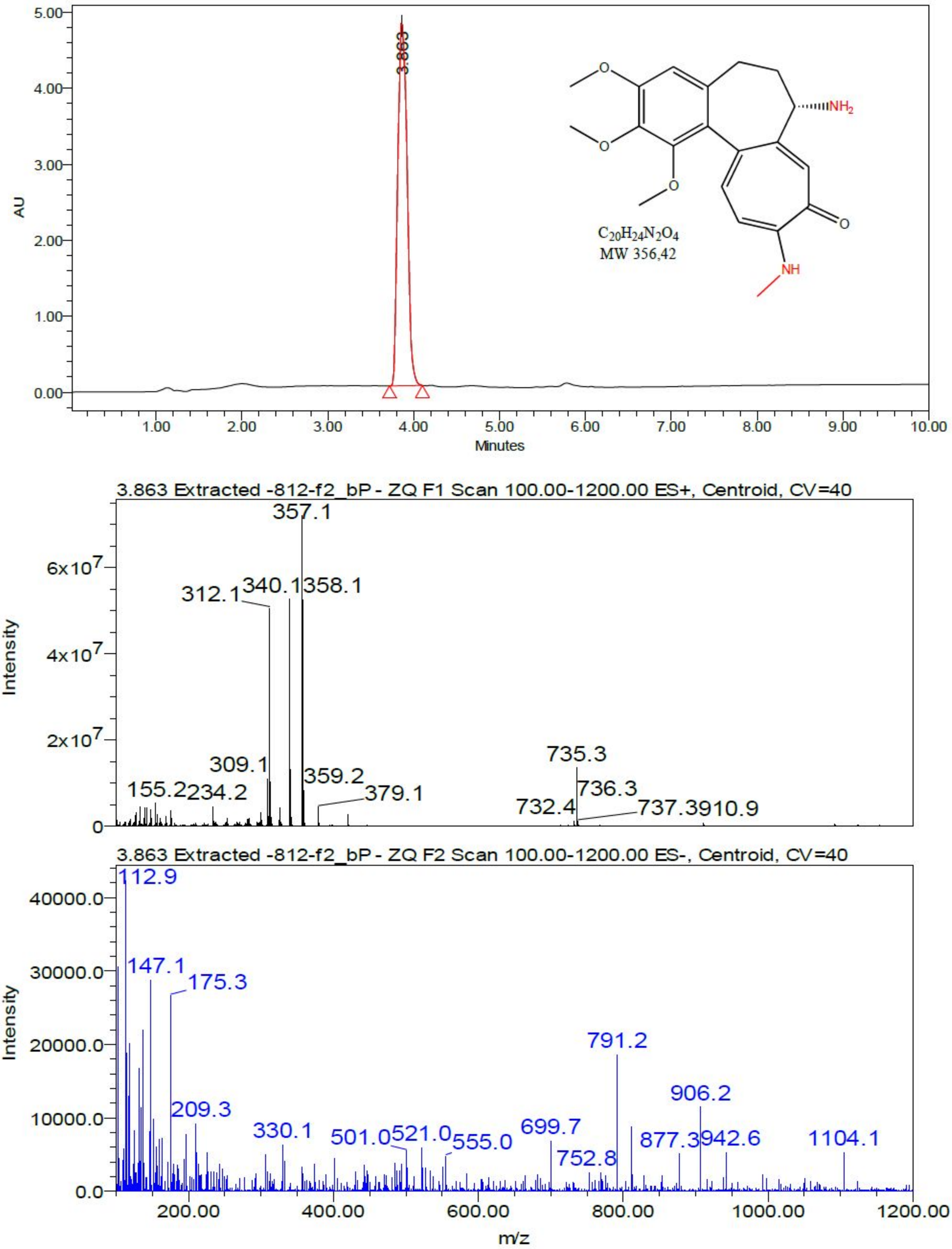

Figure S4. The LC-MS chromatogram and mass spectra of **3**.

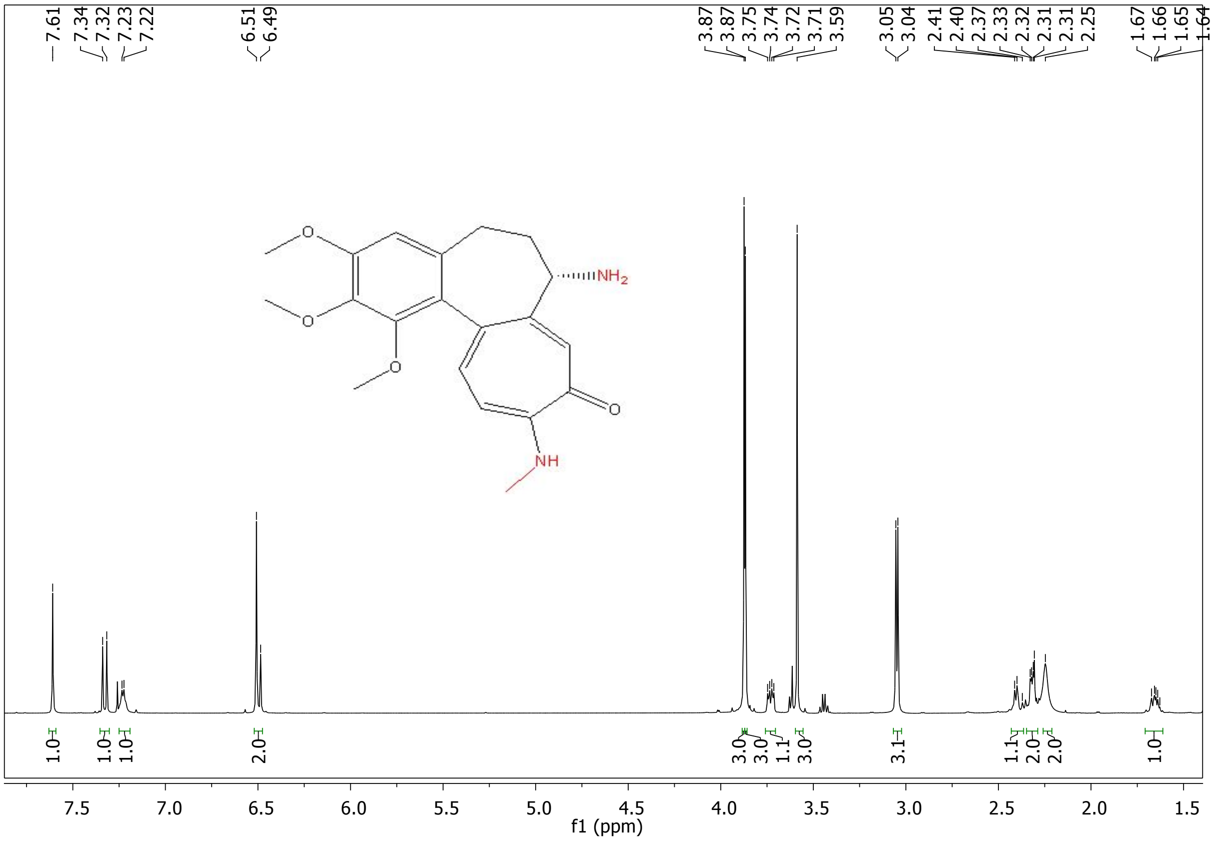

Figure S5. The ^{1}H NMR spectrum of **3** in $CDCl_3$.

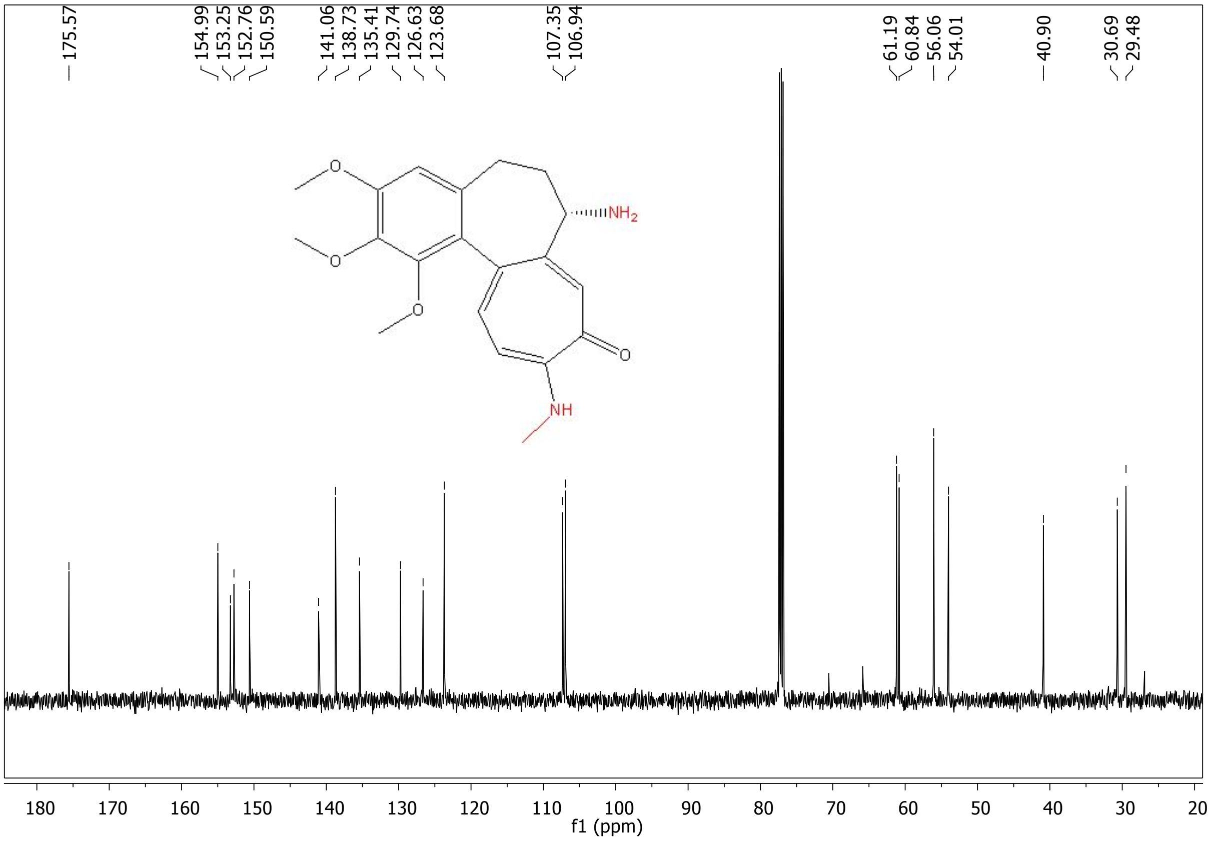

Figure S6. The ^{13}C NMR spectrum of **3** in $CDCl_3$.

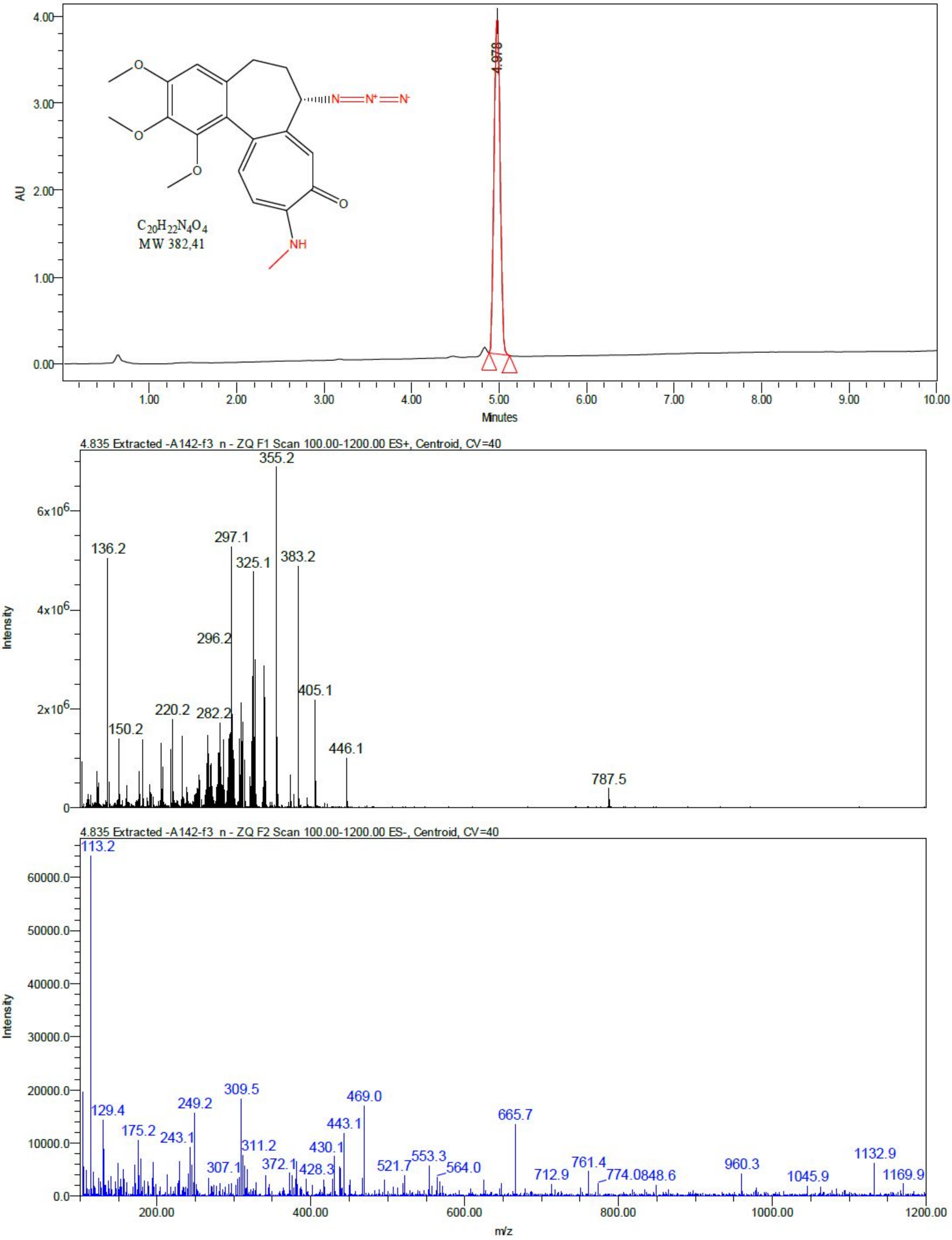

Figure S7. The LC-MS chromatogram and mass spectra of **4**.

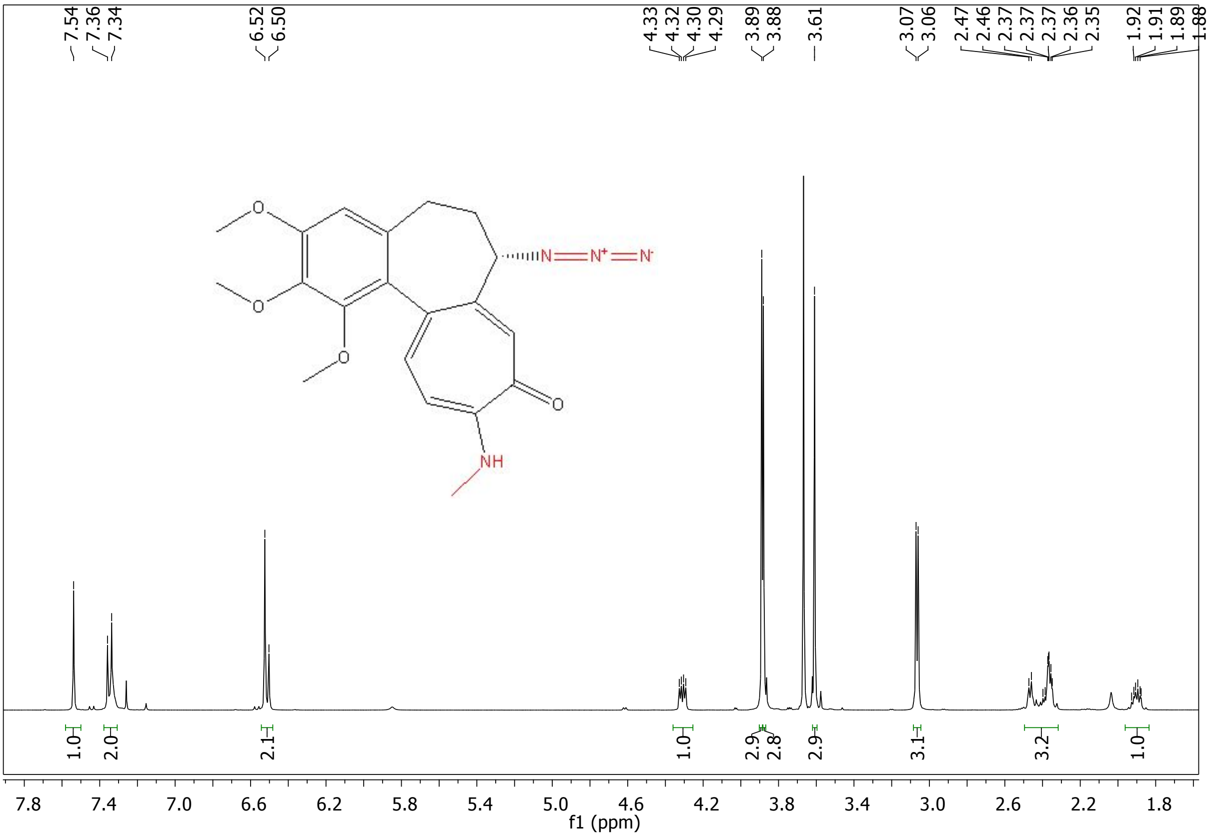

Figure S8. The ^{1}H NMR spectrum of **4** in $CDCl_3$.

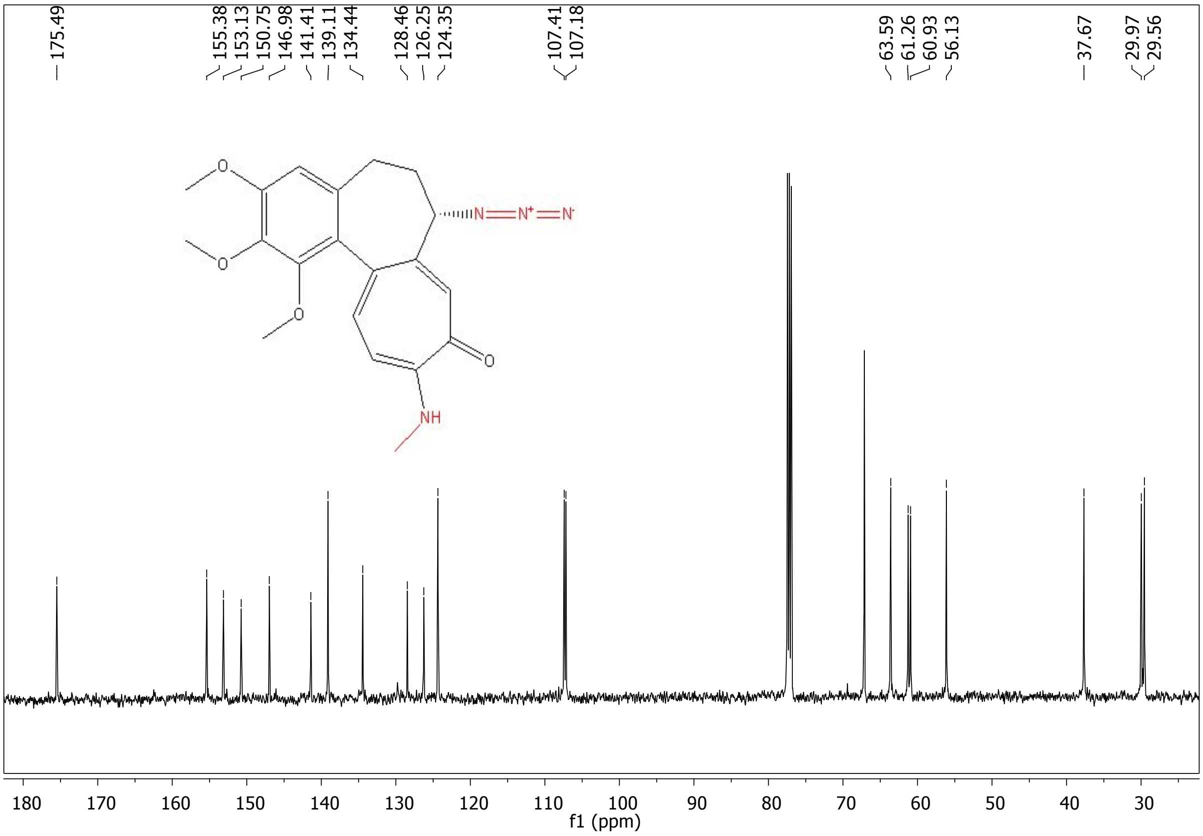

Figure S9. The ^{13}C NMR spectrum of **4** in $CDCl_3$.

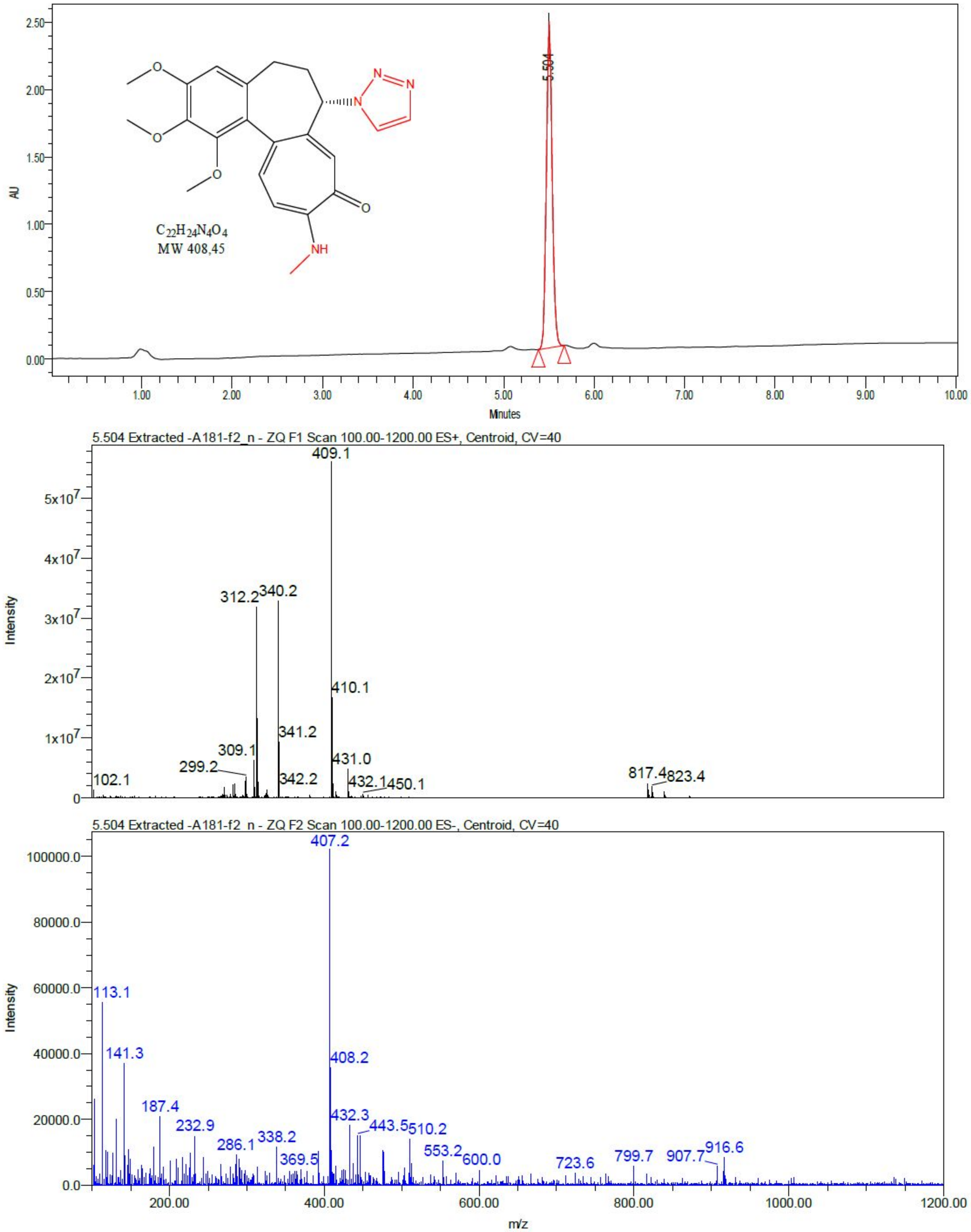

Figure S10. The LC-MS chromatogram and mass spectra of **5**.

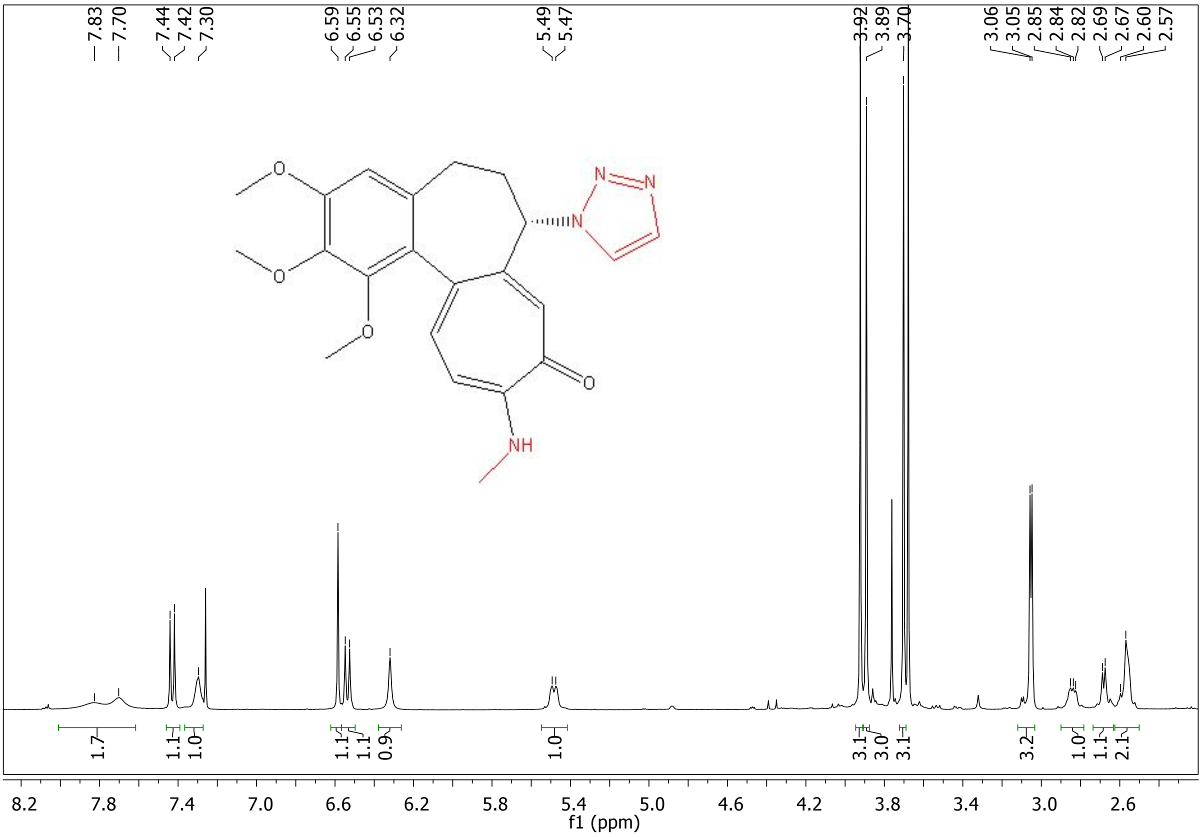

Figure S11. The ^{1}H NMR spectrum of **5** in $CDCl_3$.

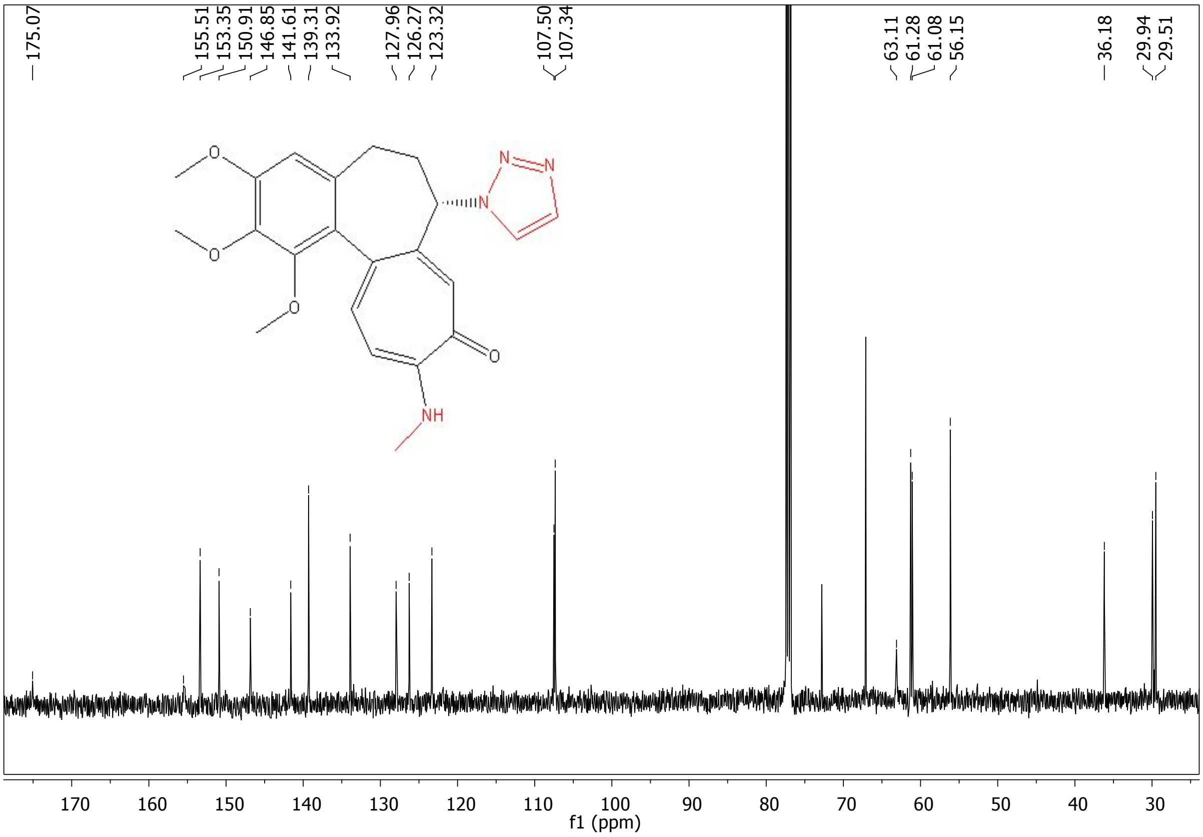

Figure S12. The ^{13}C NMR spectrum of **5** in $CDCl_3$.

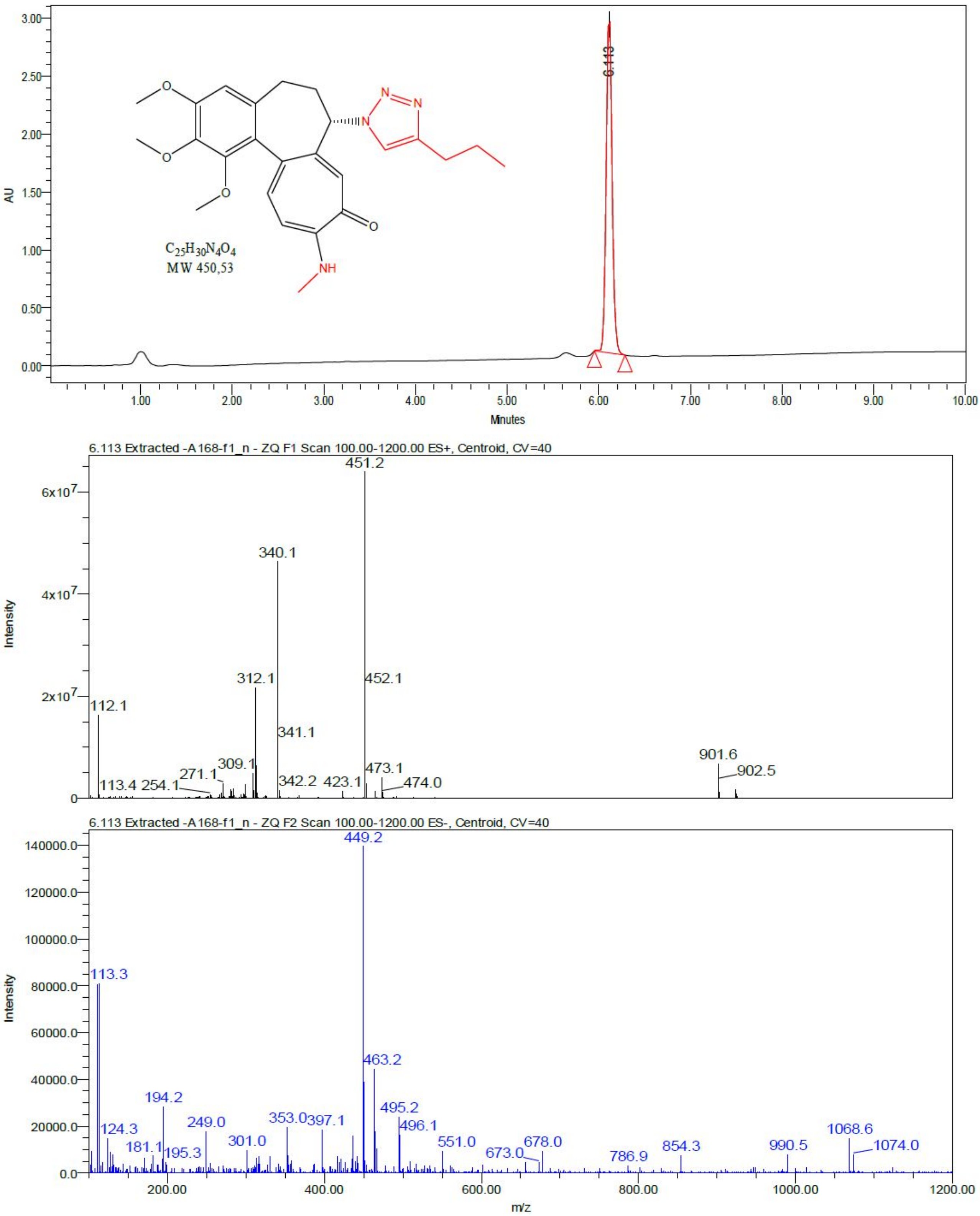

Figure S13. The LC-MS chromatogram and mass spectra of **6**.

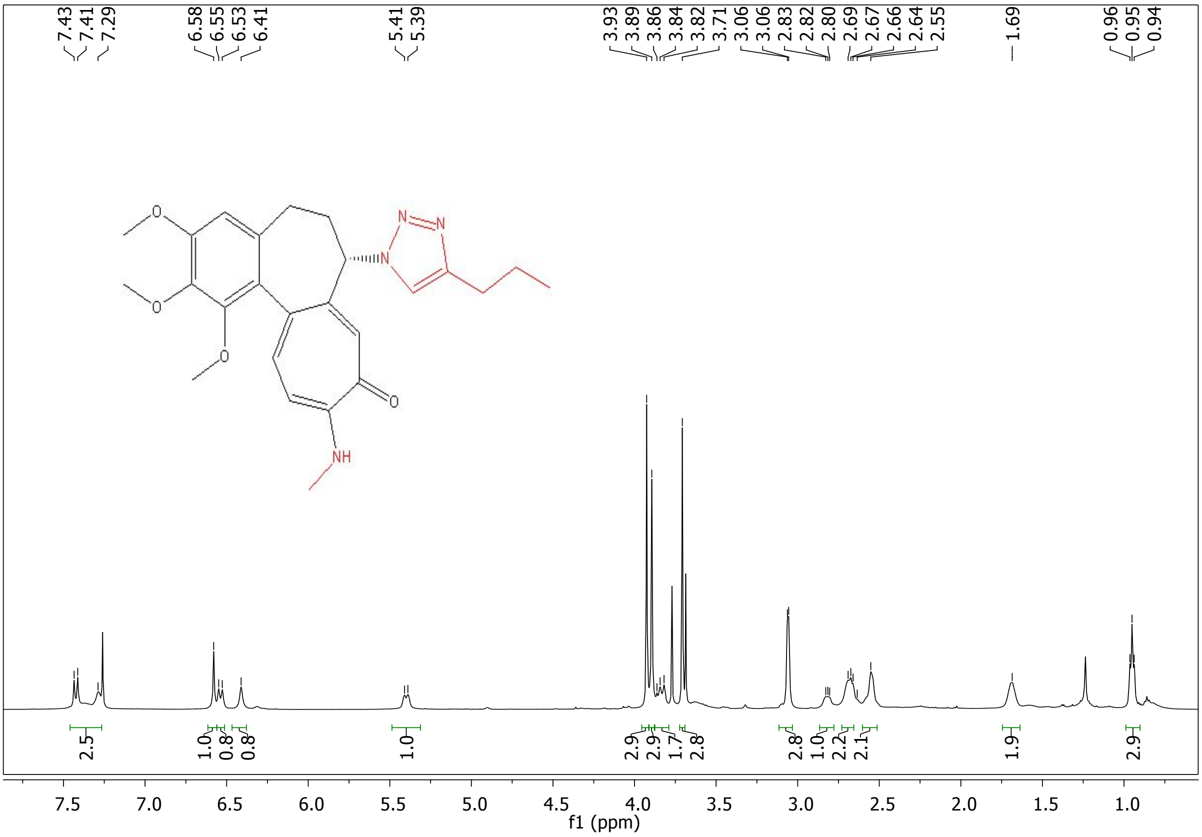

Figure S14. The ^{1}H NMR spectrum of **6** in $CDCl_3$.

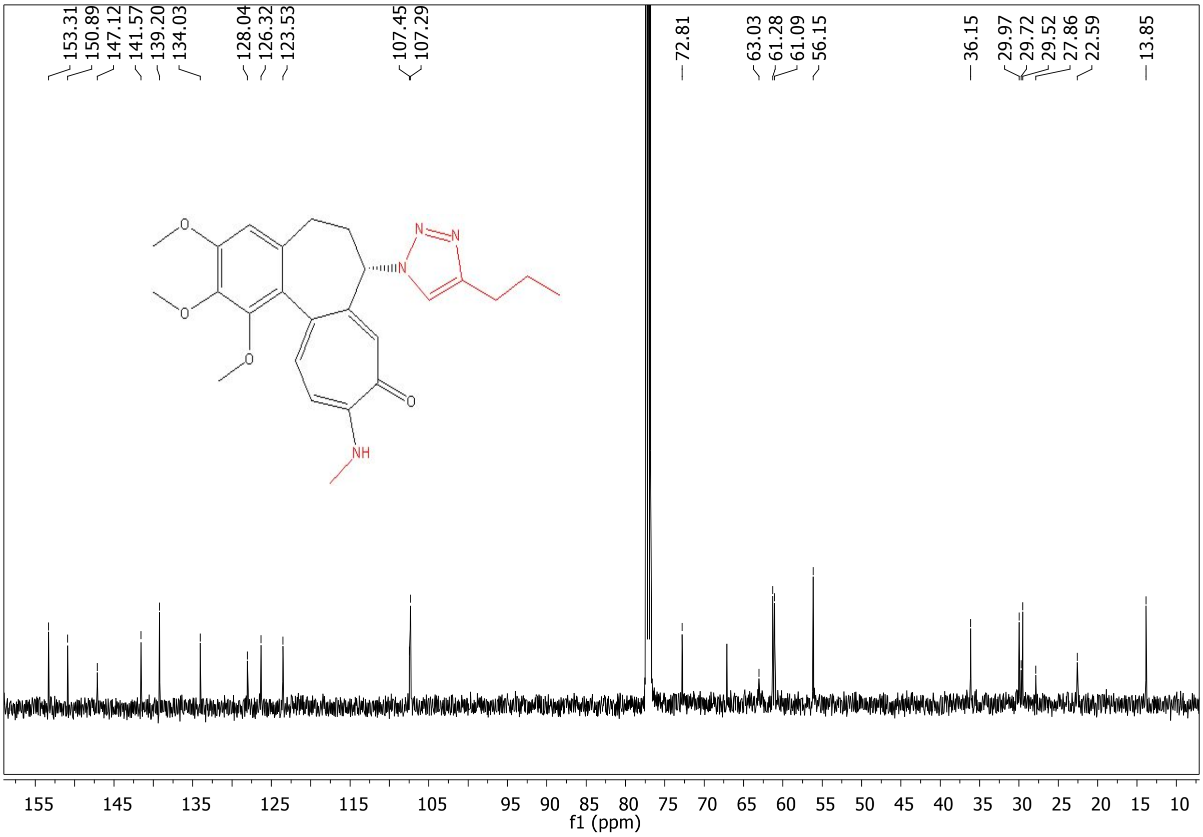

Figure S15. The ^{13}C NMR spectrum of **6** in $CDCl_3$.

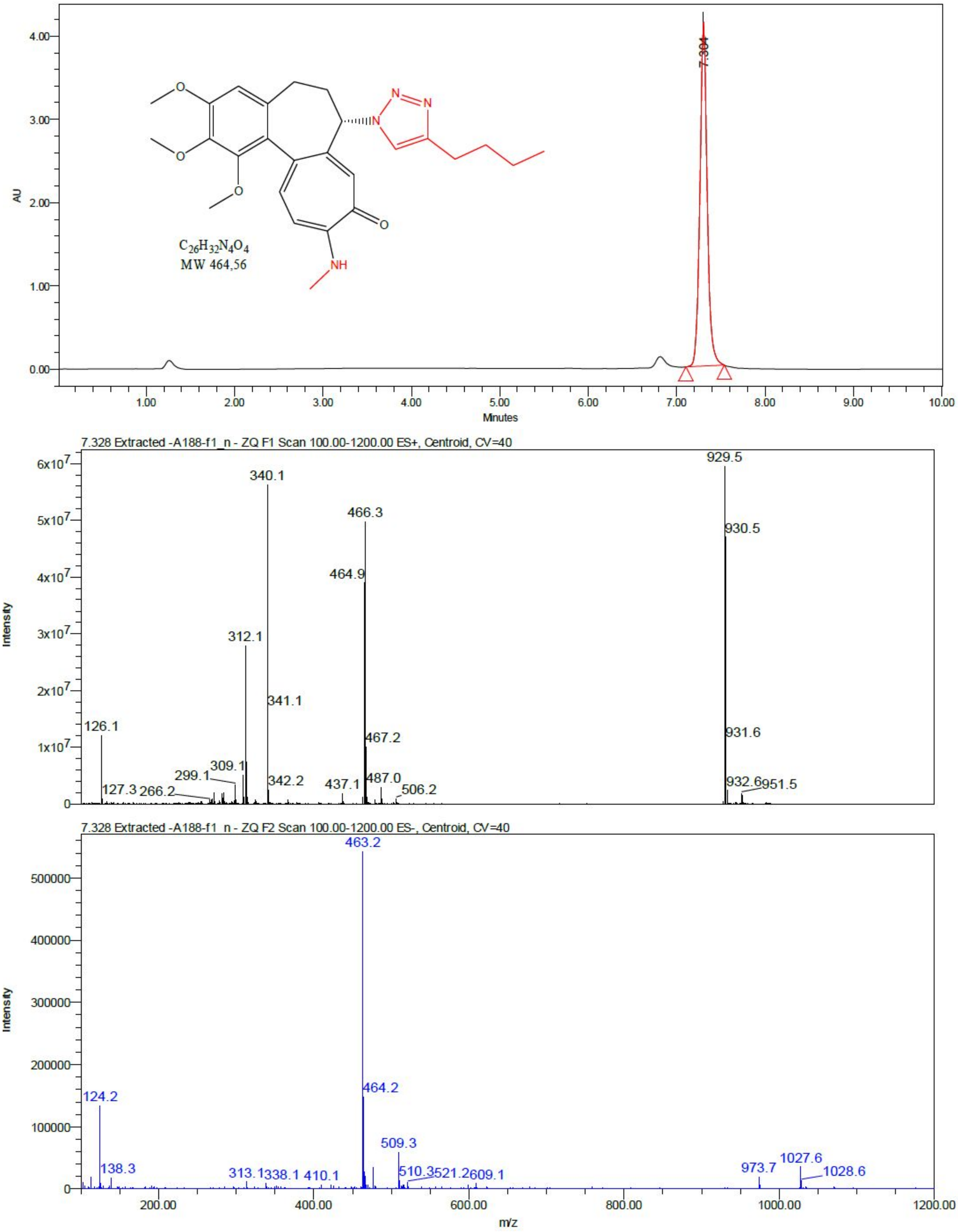

Figure S16. The LC-MS chromatogram and mass spectra of **7**.

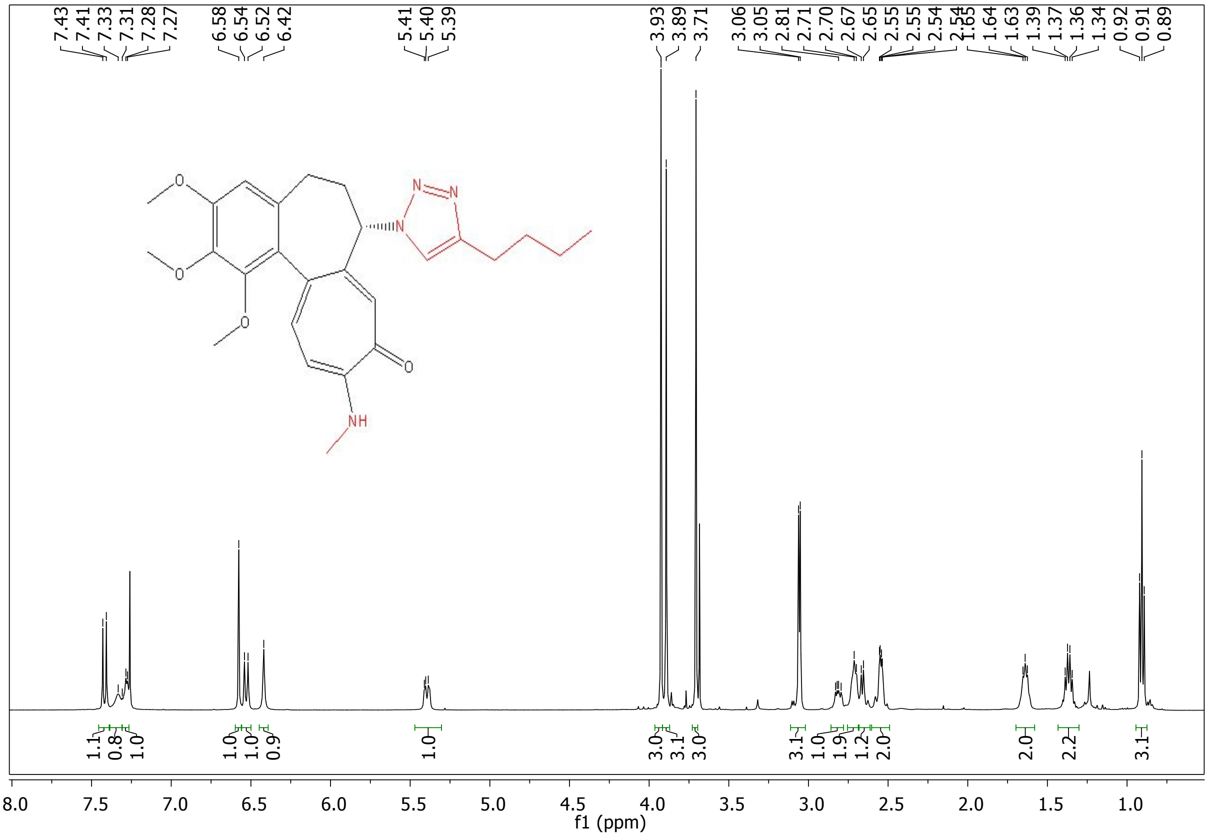

Figure S17. The ^{1}H NMR spectrum of **7** in $CDCl_3$.

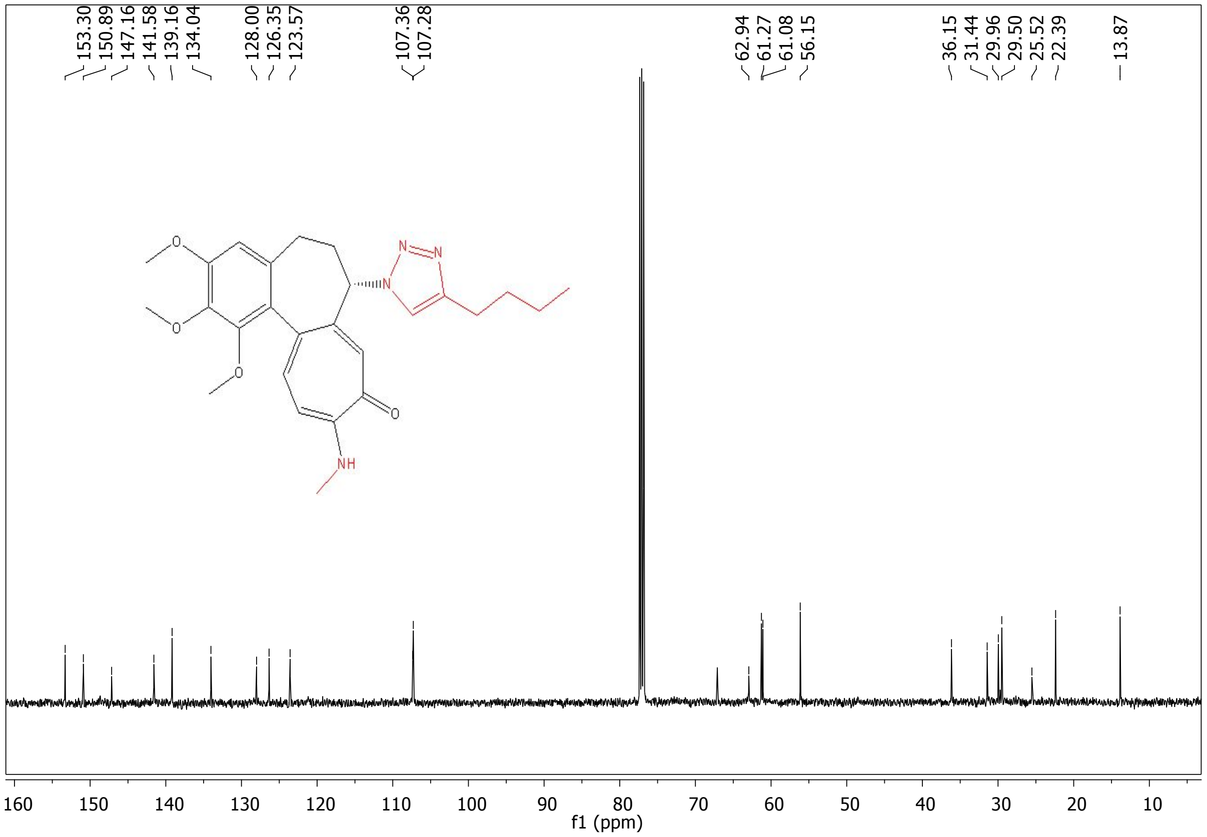

Figure S18. The ^{13}C NMR spectrum of **7** in $CDCl_3$.

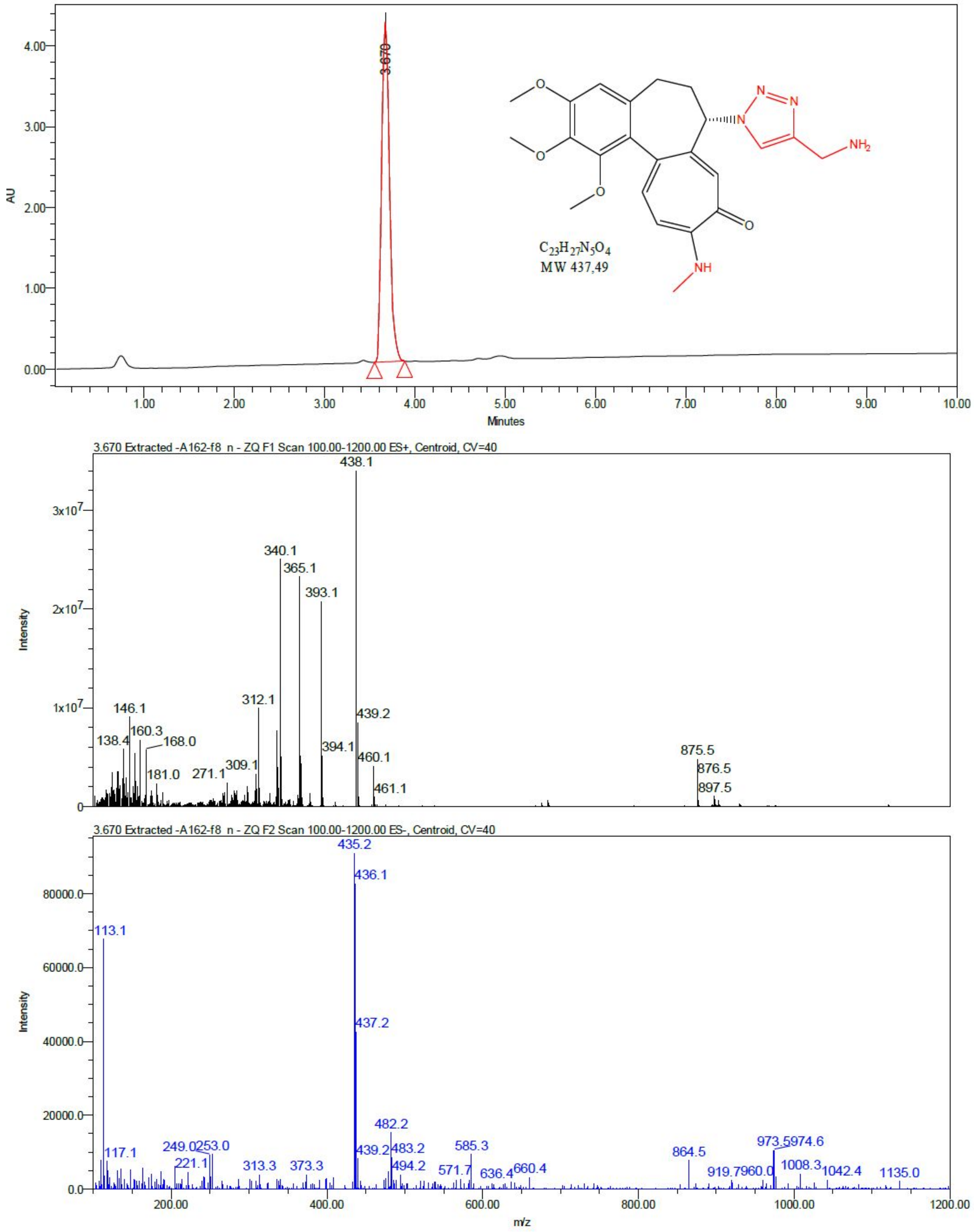

Figure S19. The LC-MS chromatogram and mass spectra of **8**.

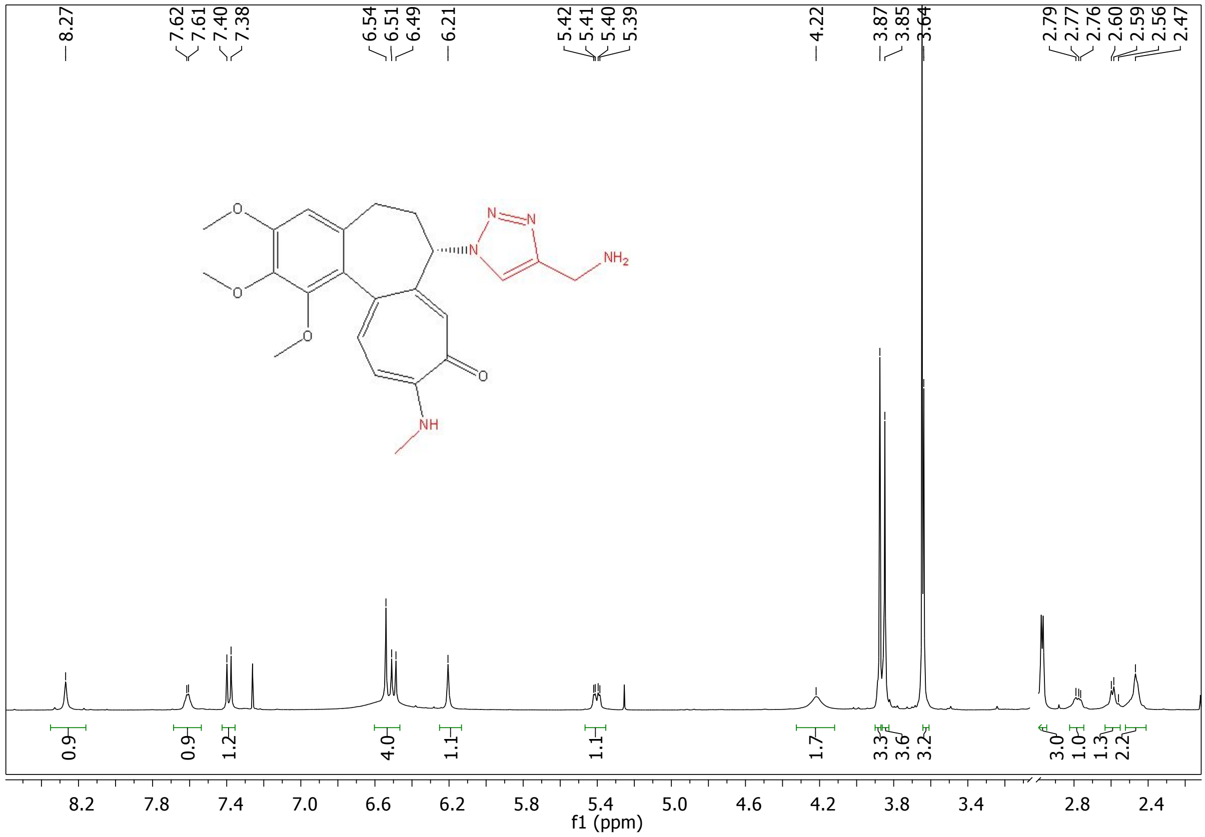

Figure S20. The ^{1}H NMR spectrum of **8** in $CDCl_3$.

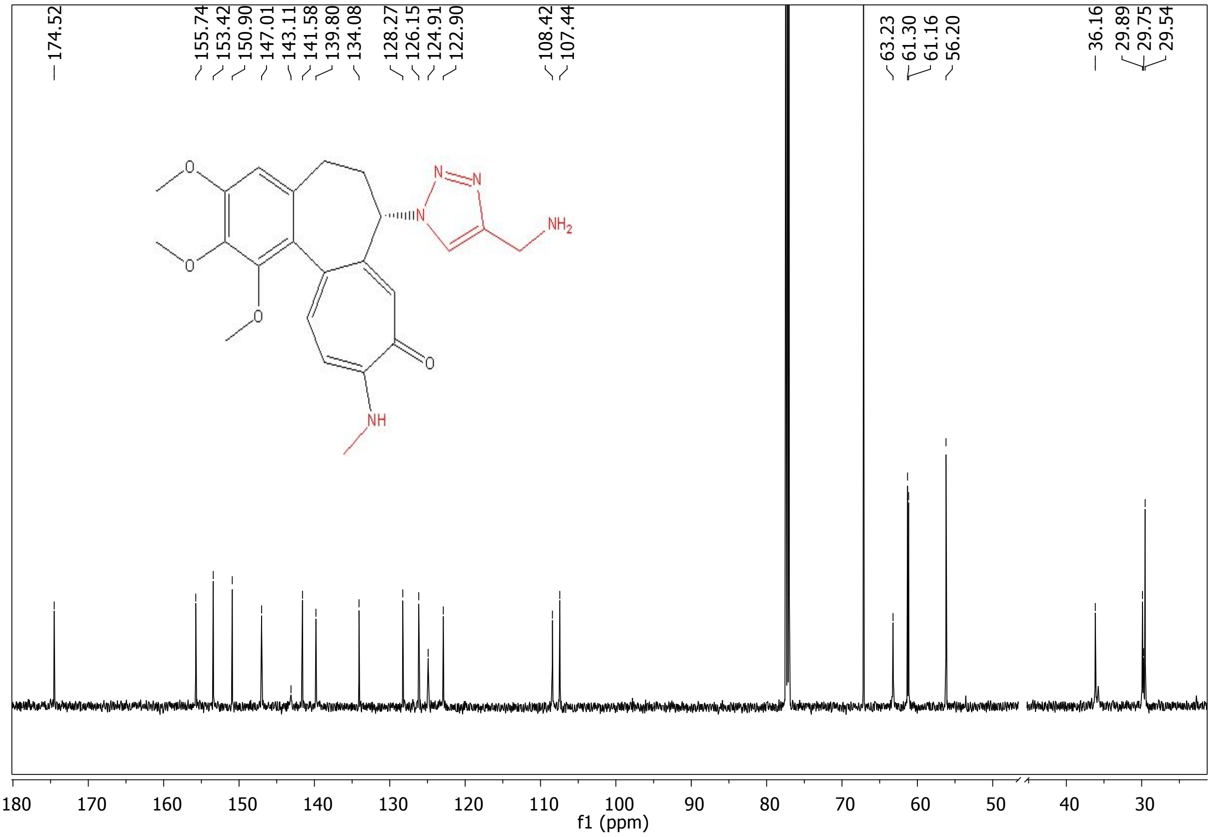

Figure S21. The ^{13}C NMR spectrum of **8** in $CDCl_3$.

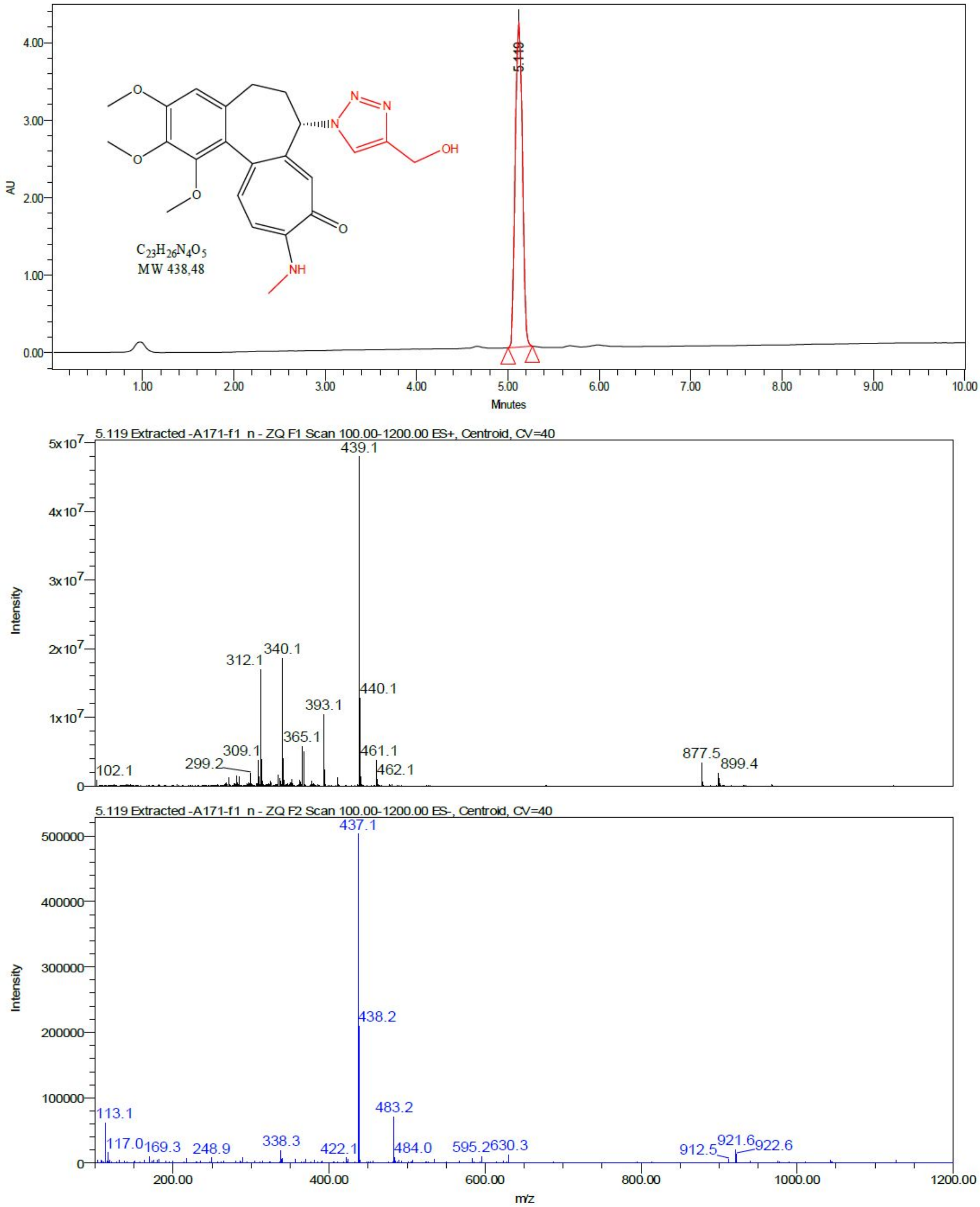

Figure S22. The LC-MS chromatogram and mass spectra of **9**.

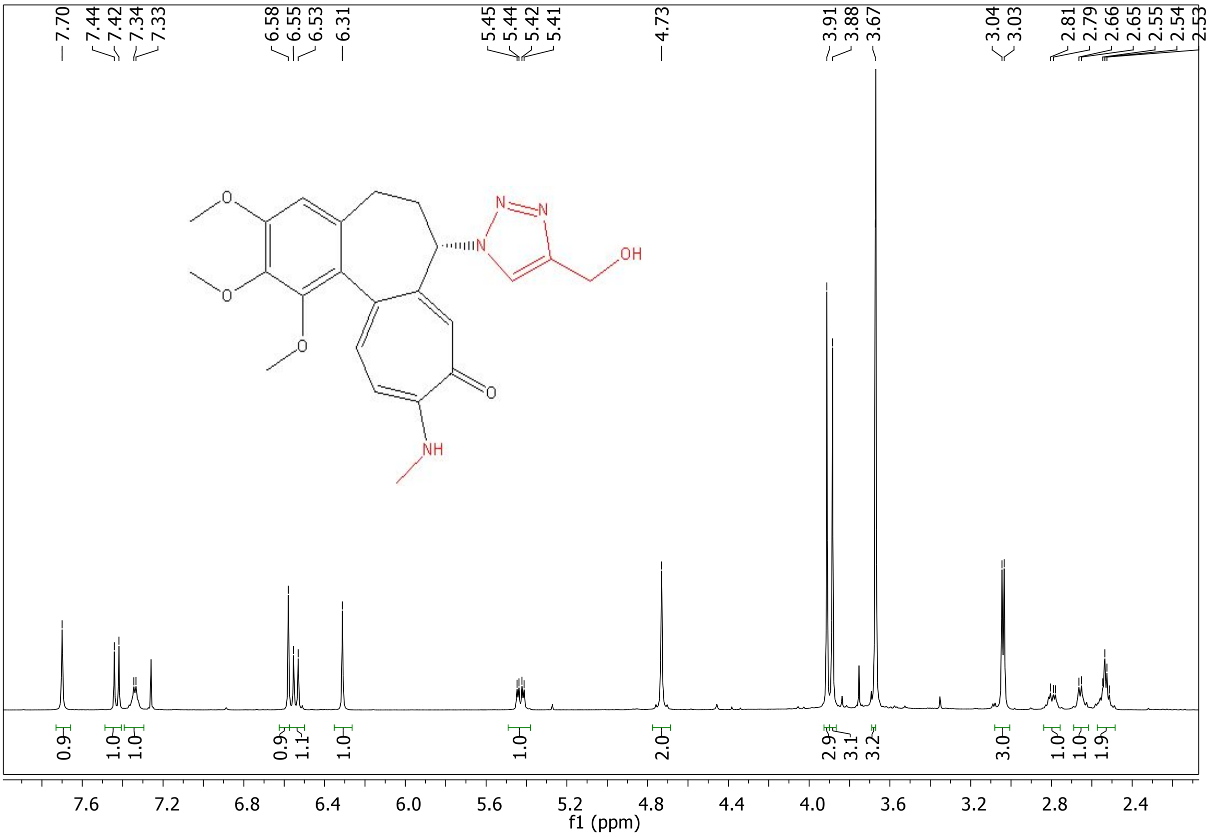

Figure S23. The ^{1}H NMR spectrum of **9** in $CDCl_3$.

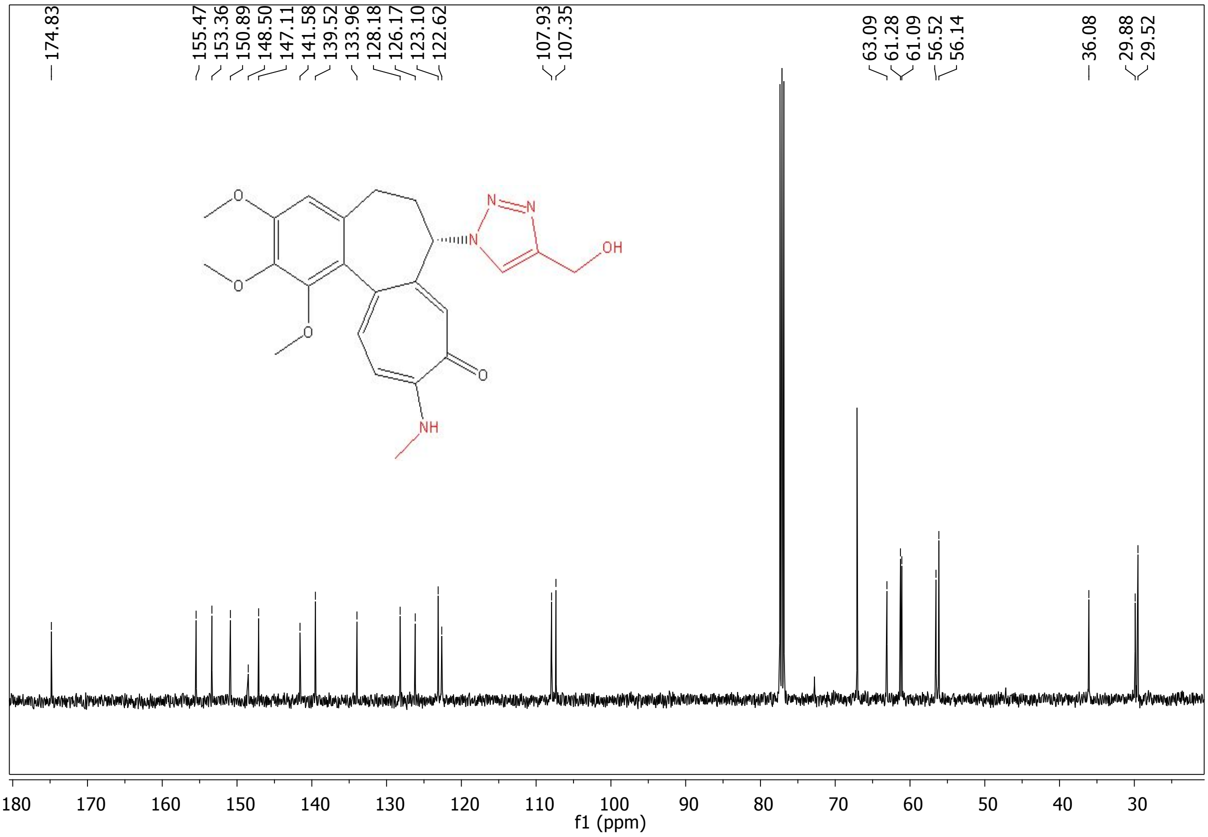

Figure S24. The ^{13}C NMR spectrum of **9** in $CDCl_3$.

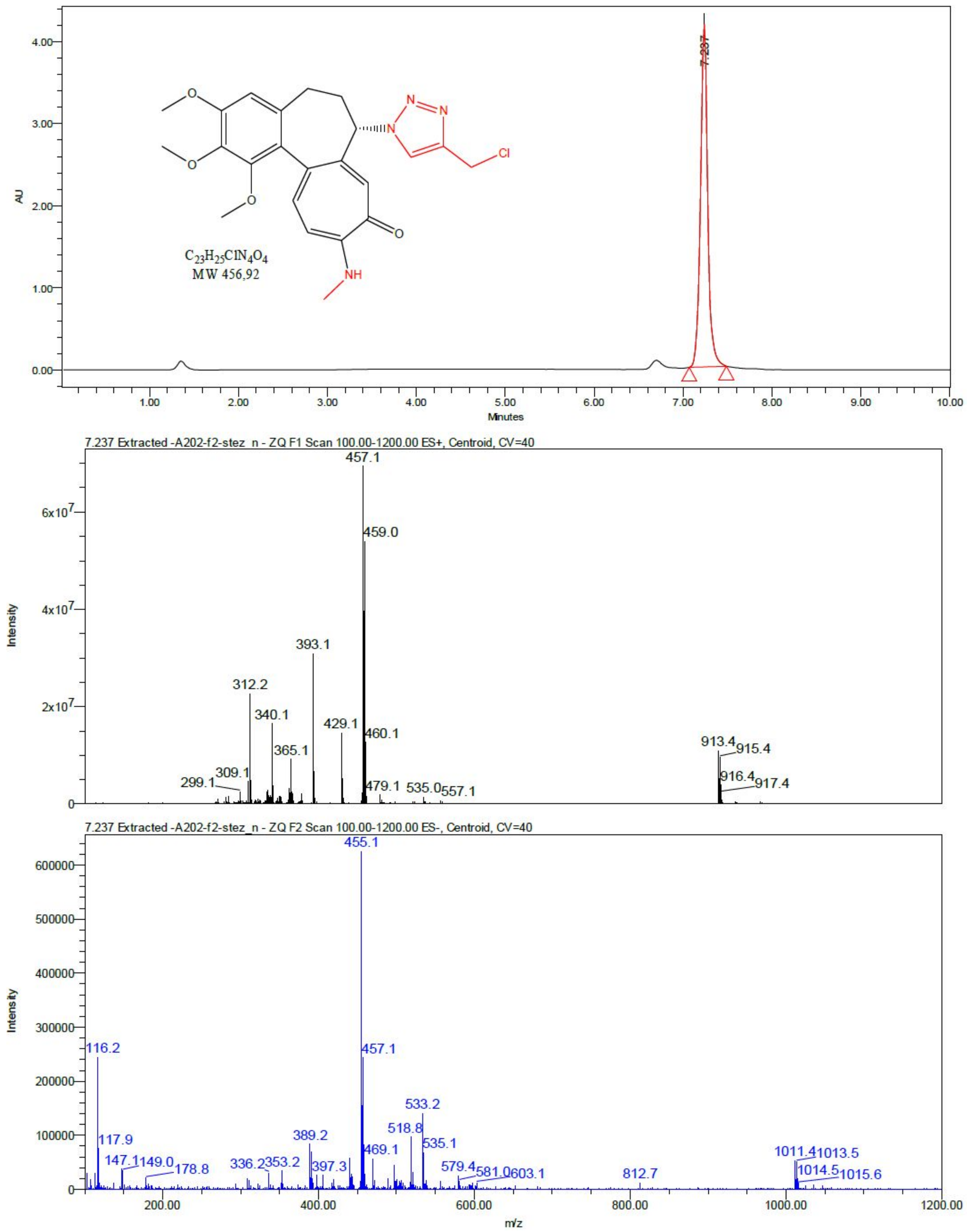

Figure S25. The LC-MS chromatogram and mass spectra of **10**.

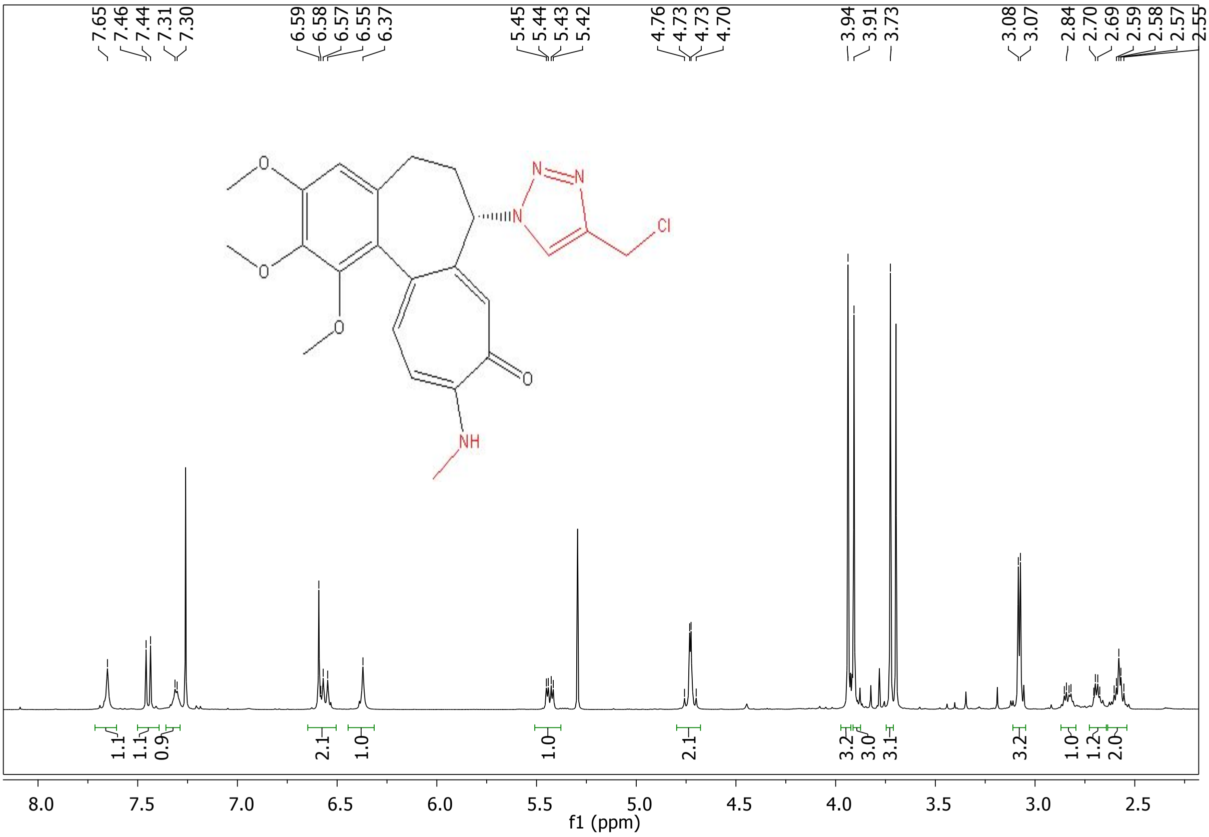

Figure S26. The ^{1}H NMR spectrum of **10** in $CDCl_3$.

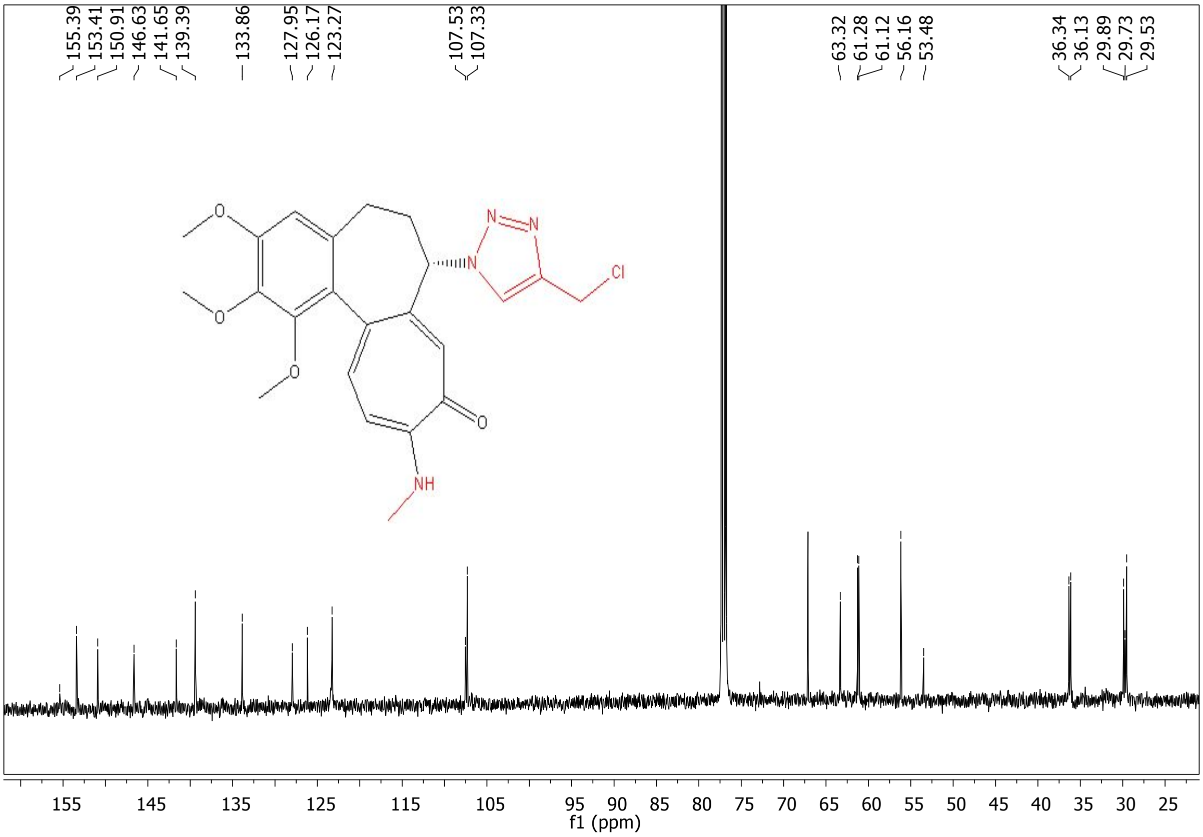

Figure S27. The ^{13}C NMR spectrum of **10** in $CDCl_3$.

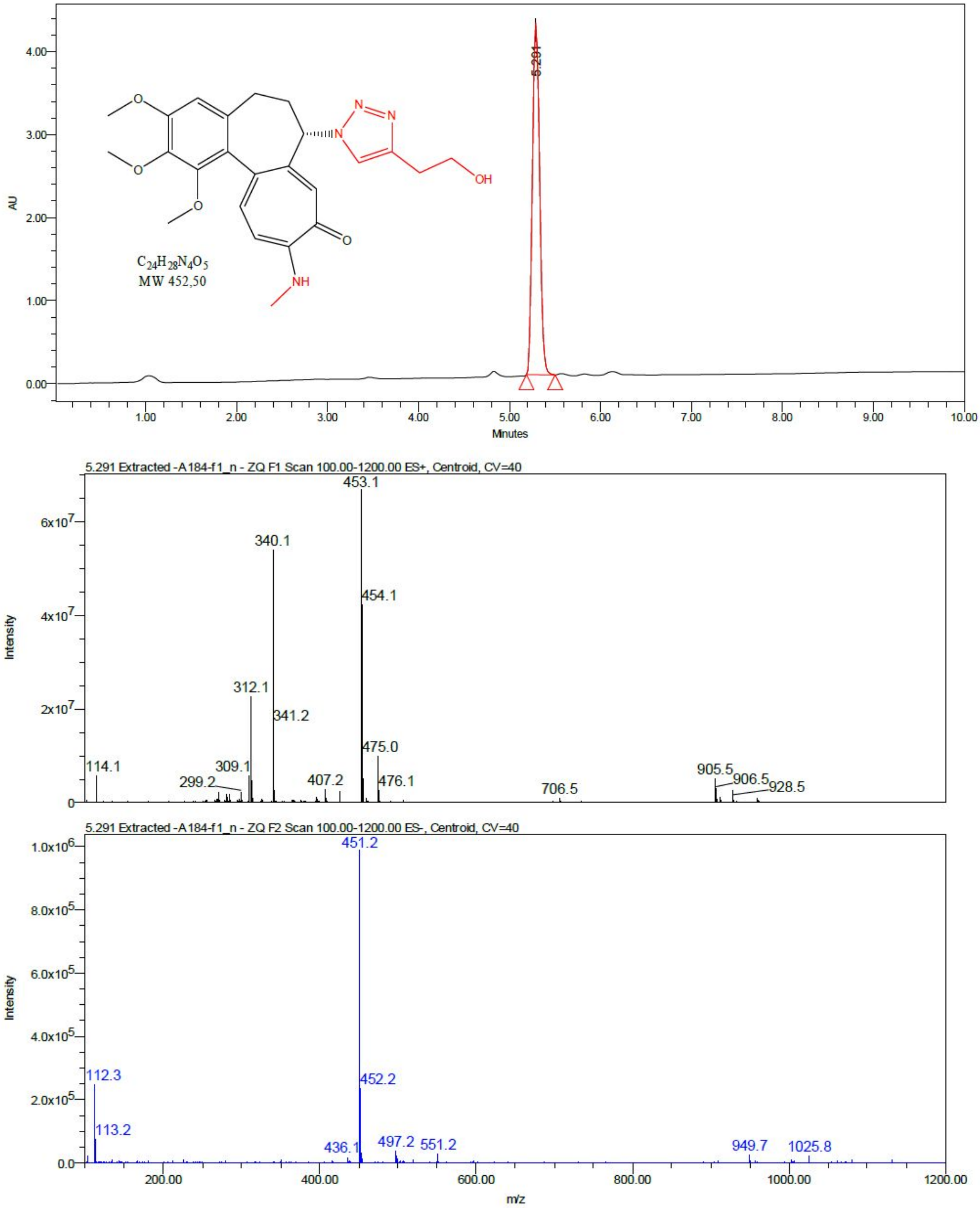

Figure S28. The LC-MS chromatogram and mass spectra of **11**.

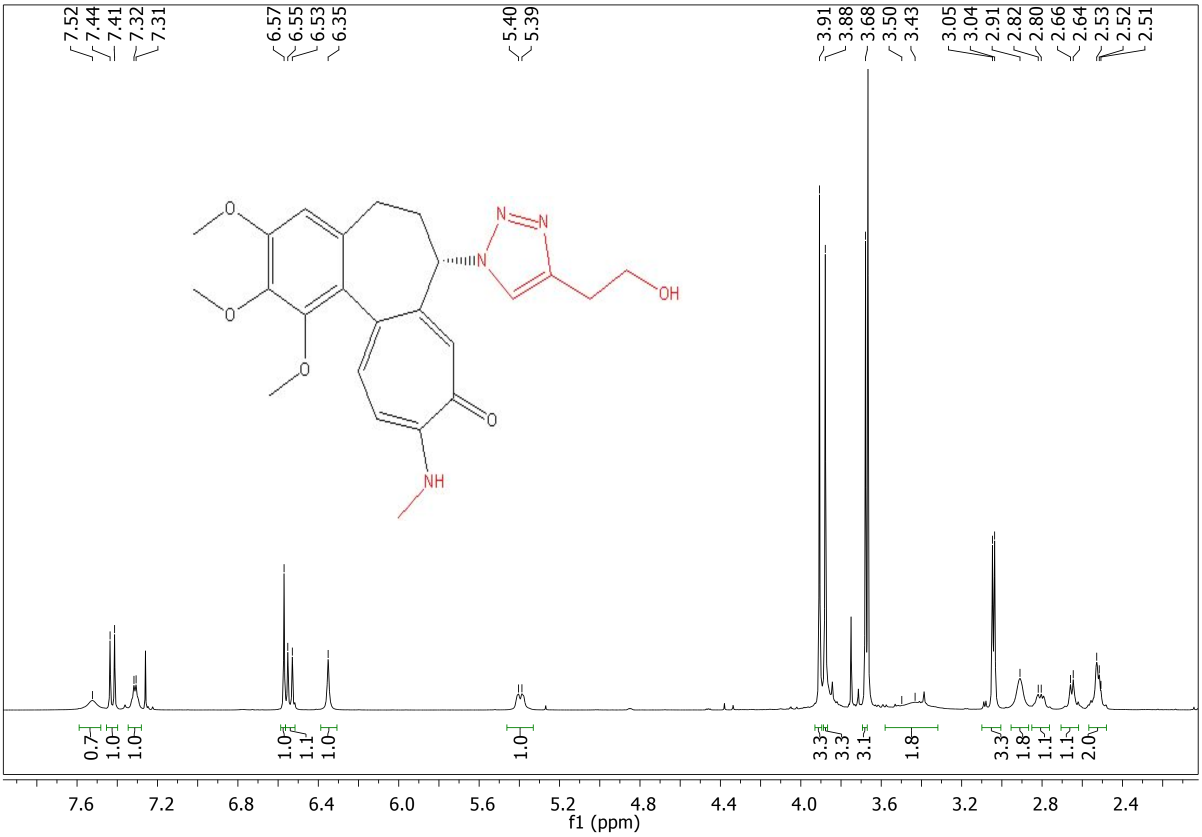

Figure S29. The ^{1}H NMR spectrum of **11** in $CDCl_3$.

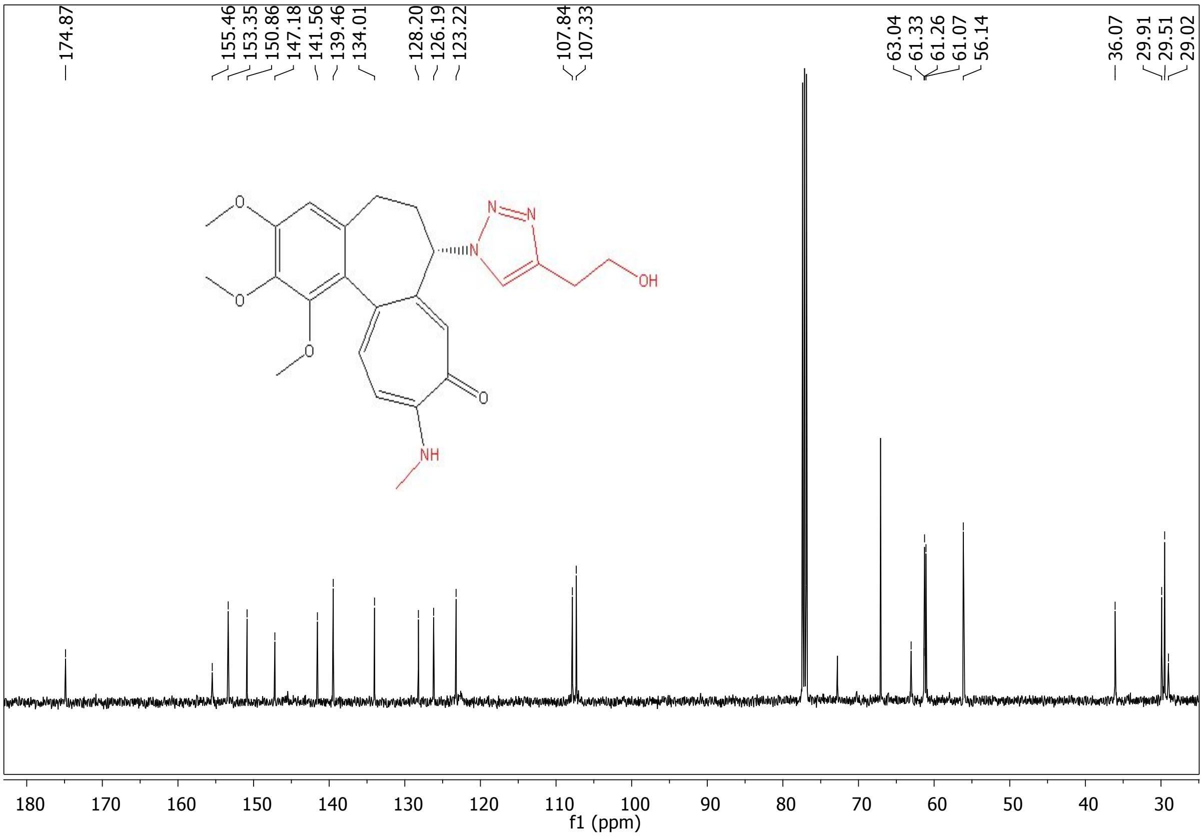

Figure S30. The ^{13}C NMR spectrum of **11** in $CDCl_3$.

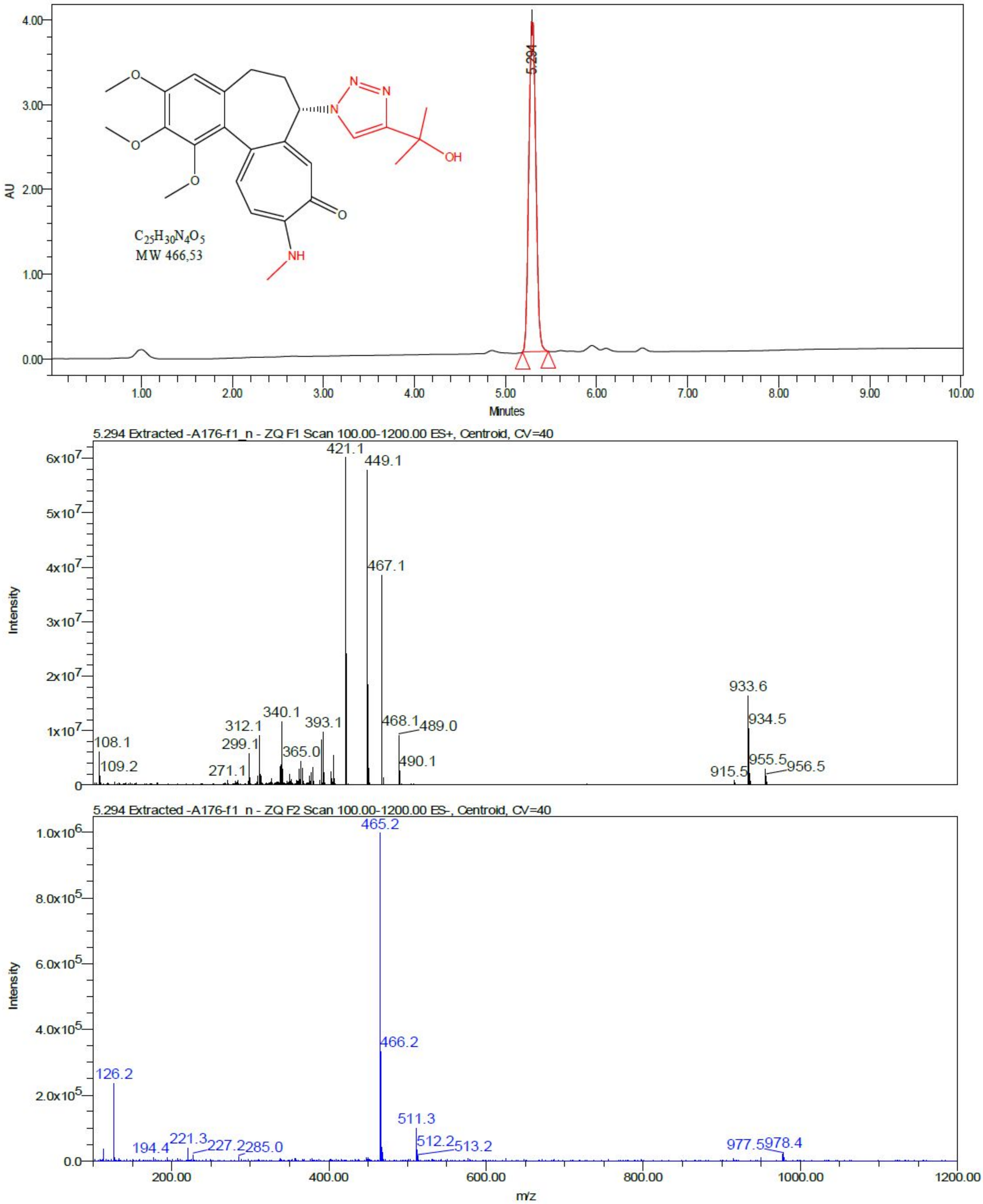

Figure S31. The LC-MS chromatogram and mass spectra of **12**.

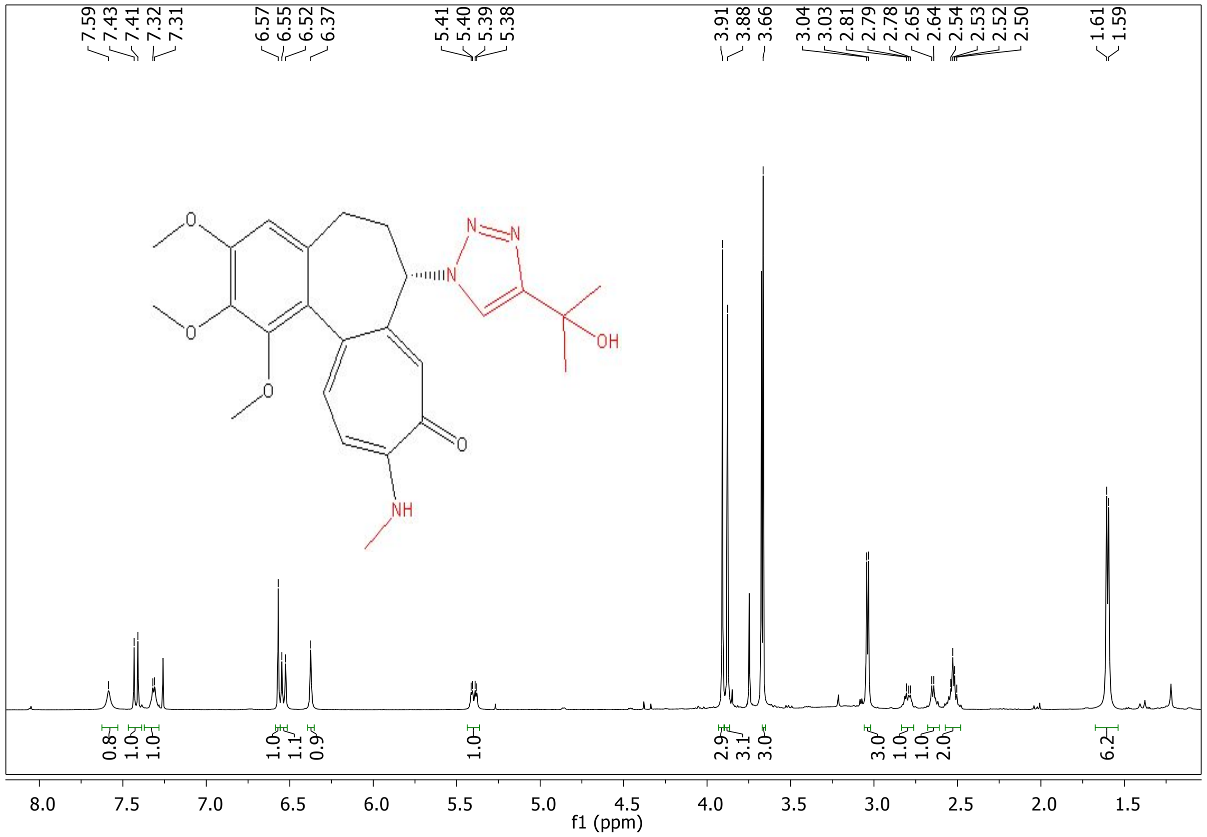

Figure S32. The ^{1}H NMR spectrum of **12** in $CDCl_3$.

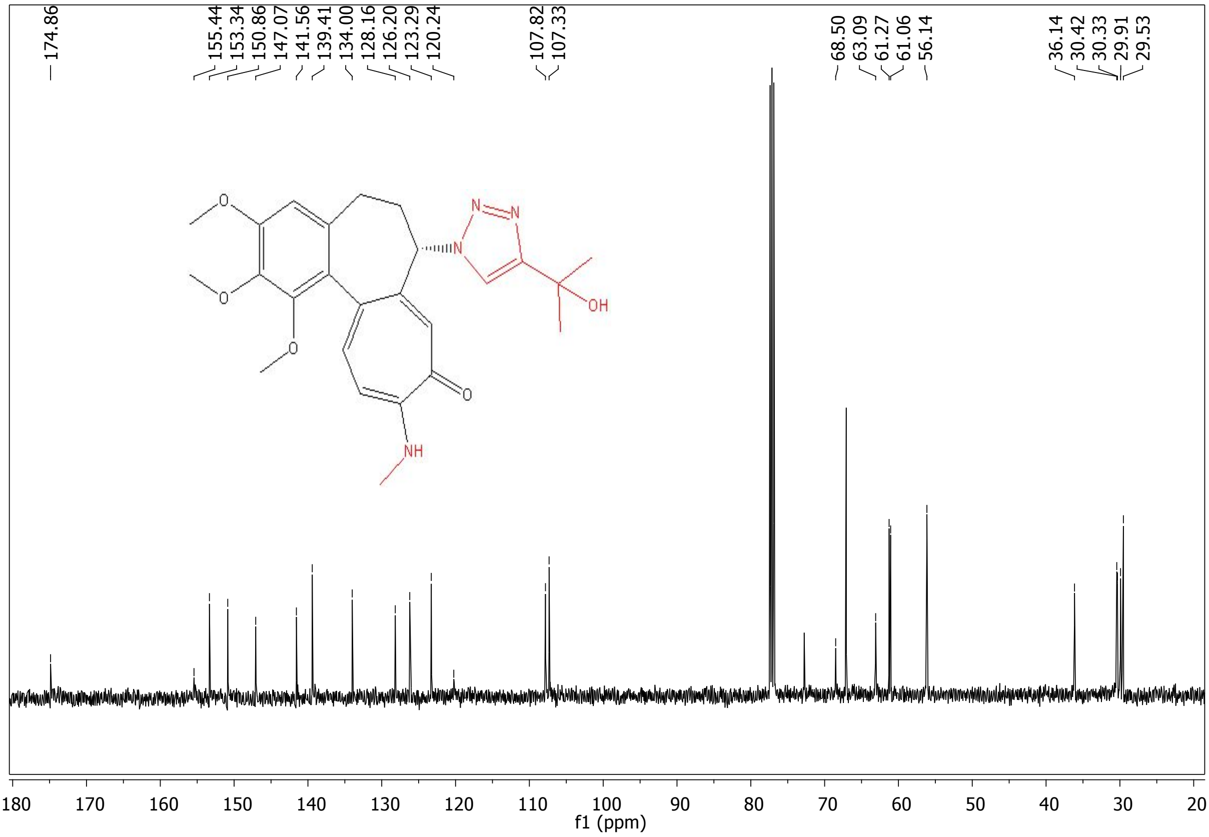

Figure S33. The ^{13}C NMR spectrum of **12** in $CDCl_3$.

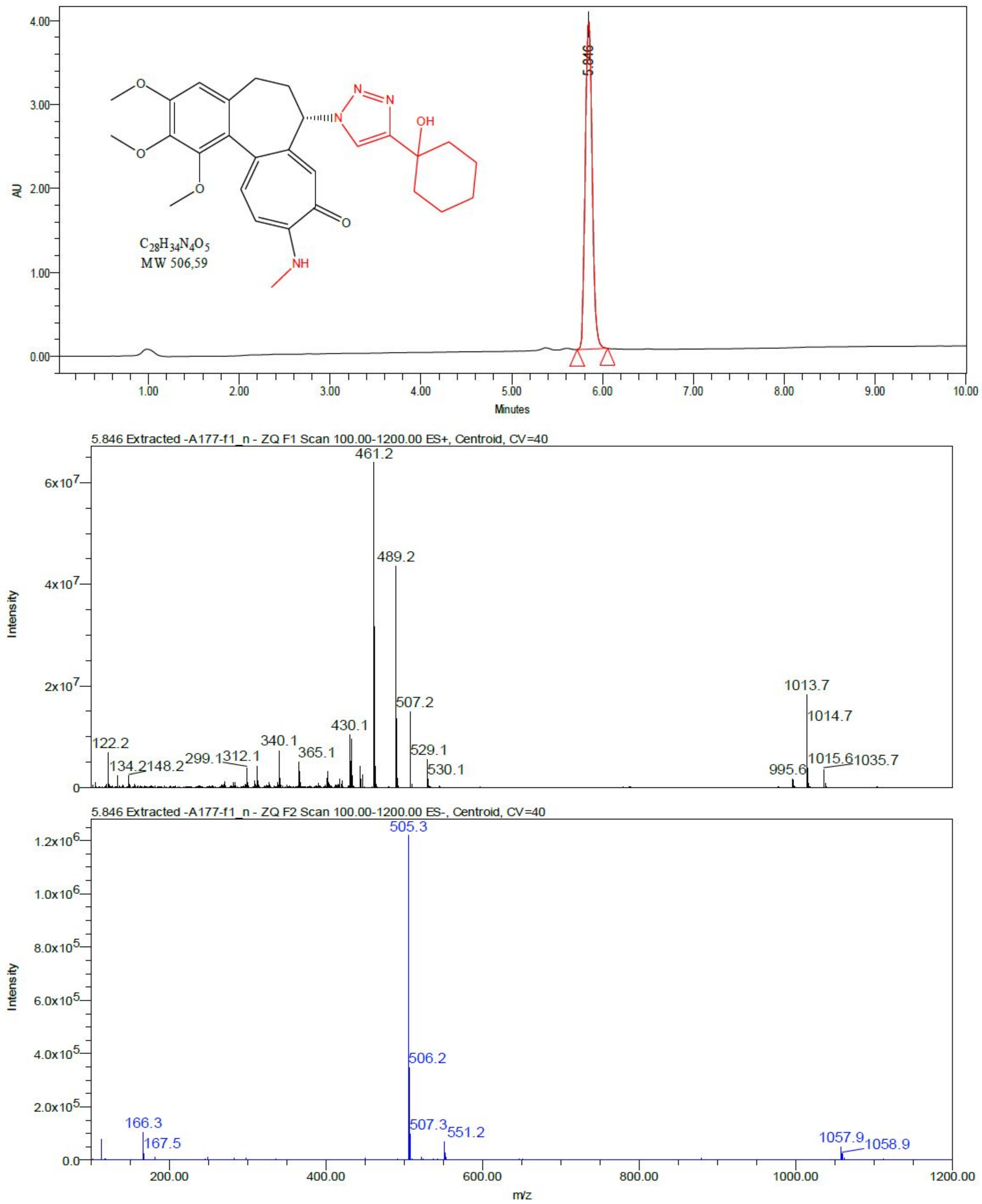

Figure S34. The LC-MS chromatogram and mass spectra of **13**.

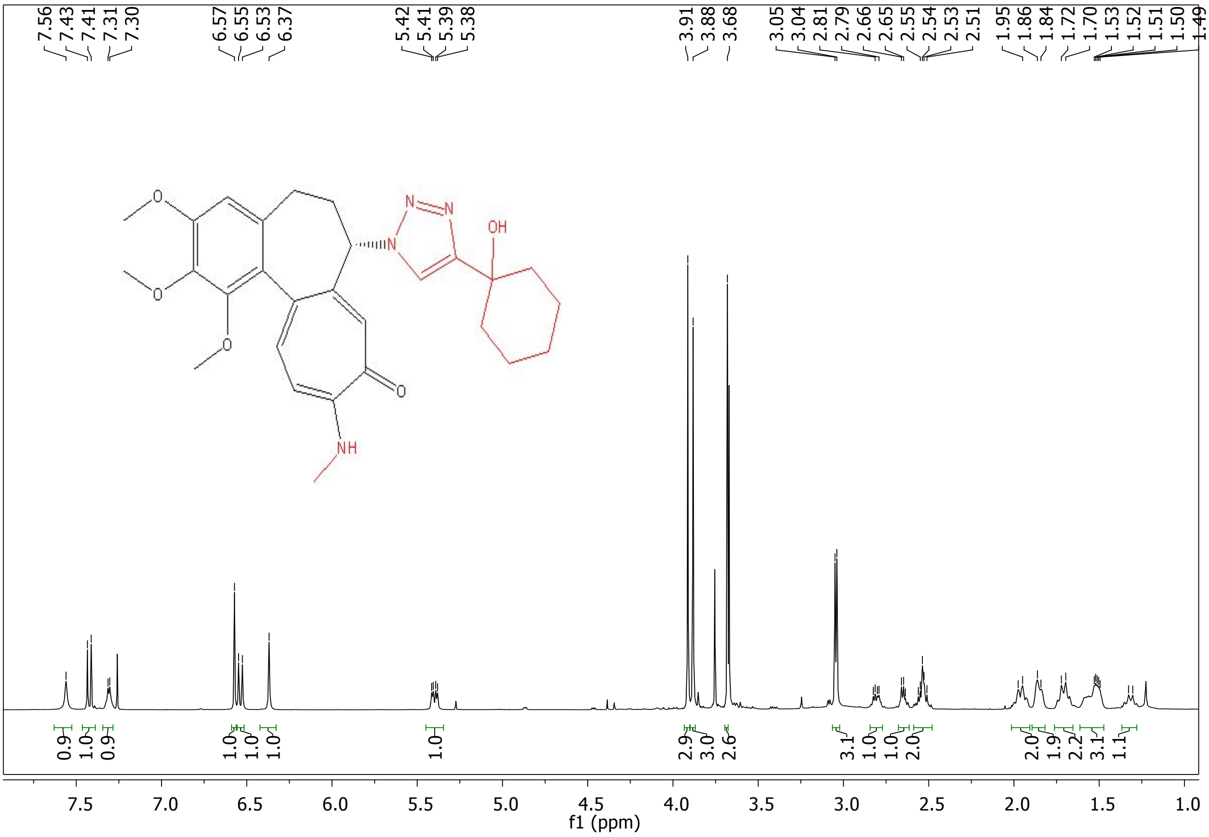

Figure S35. The ^{1}H NMR spectrum of **13** in $CDCl_3$.

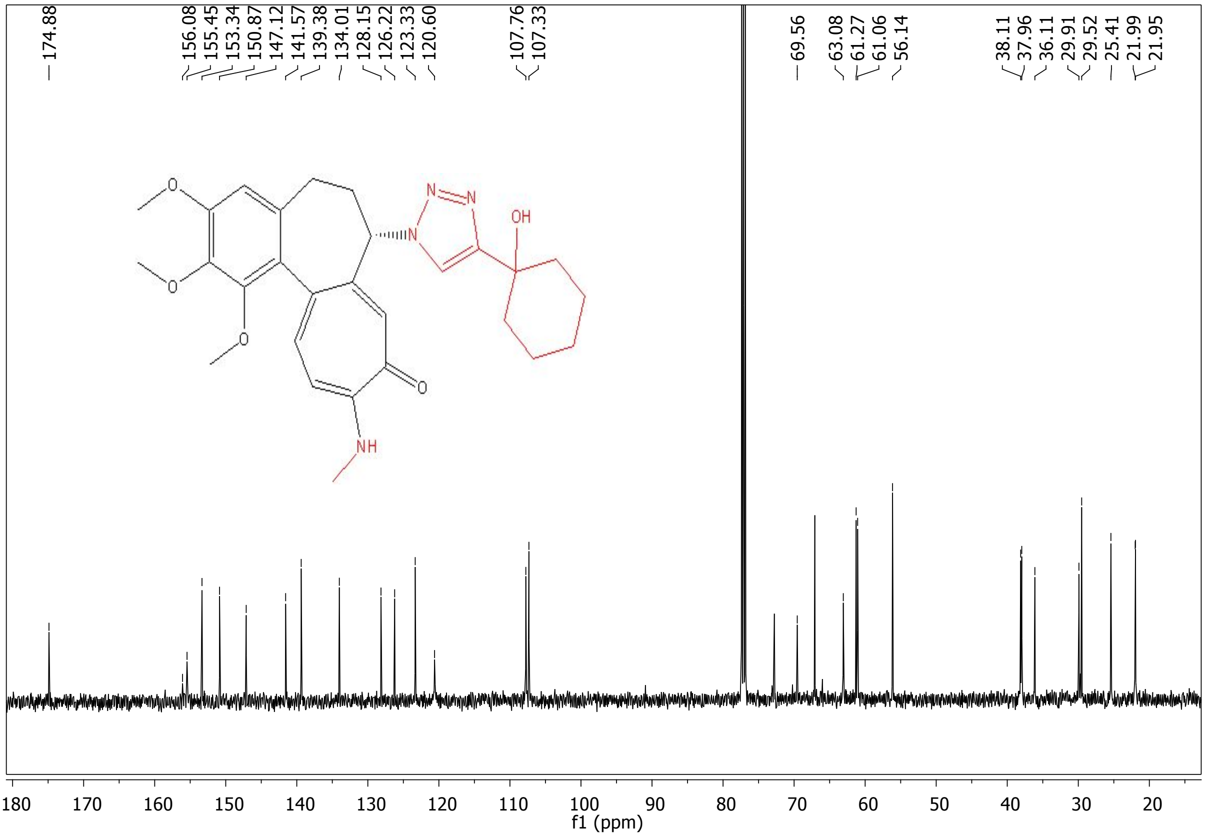

Figure S36. The ^{13}C NMR spectrum of **13** in $CDCl_3$.

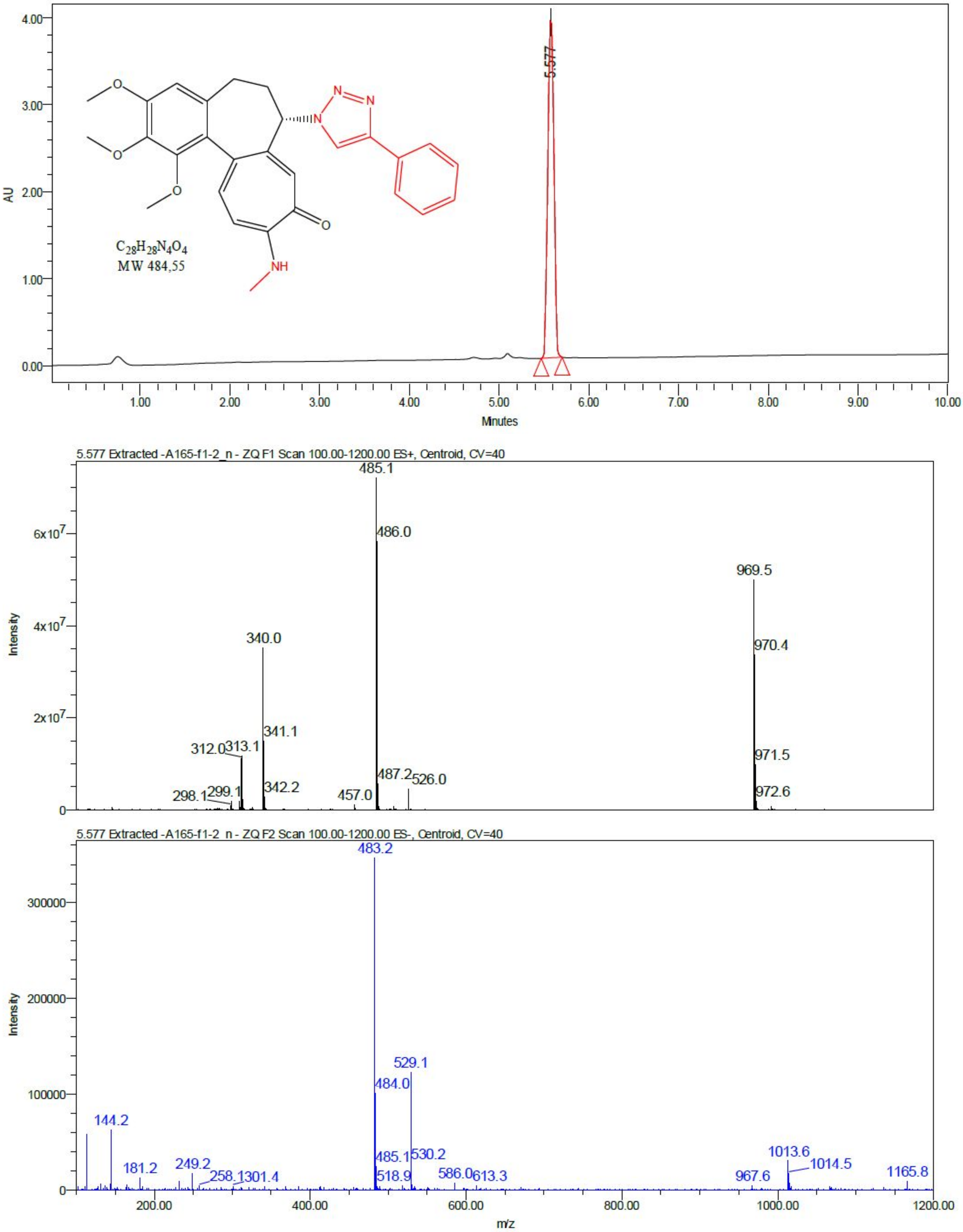

Figure S37. The LC-MS chromatogram and mass spectra of **14**.

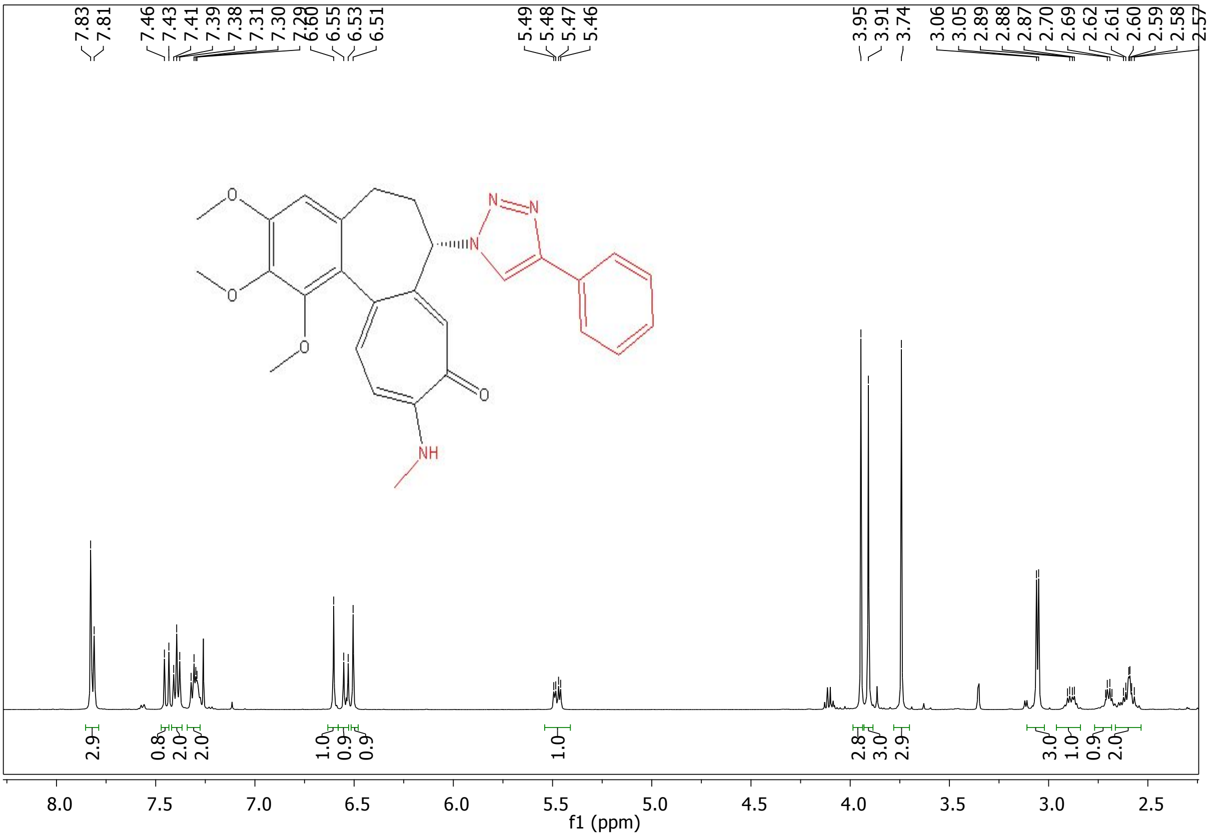

Figure S38. The ^{1}H NMR spectrum of **14** in $CDCl_3$.

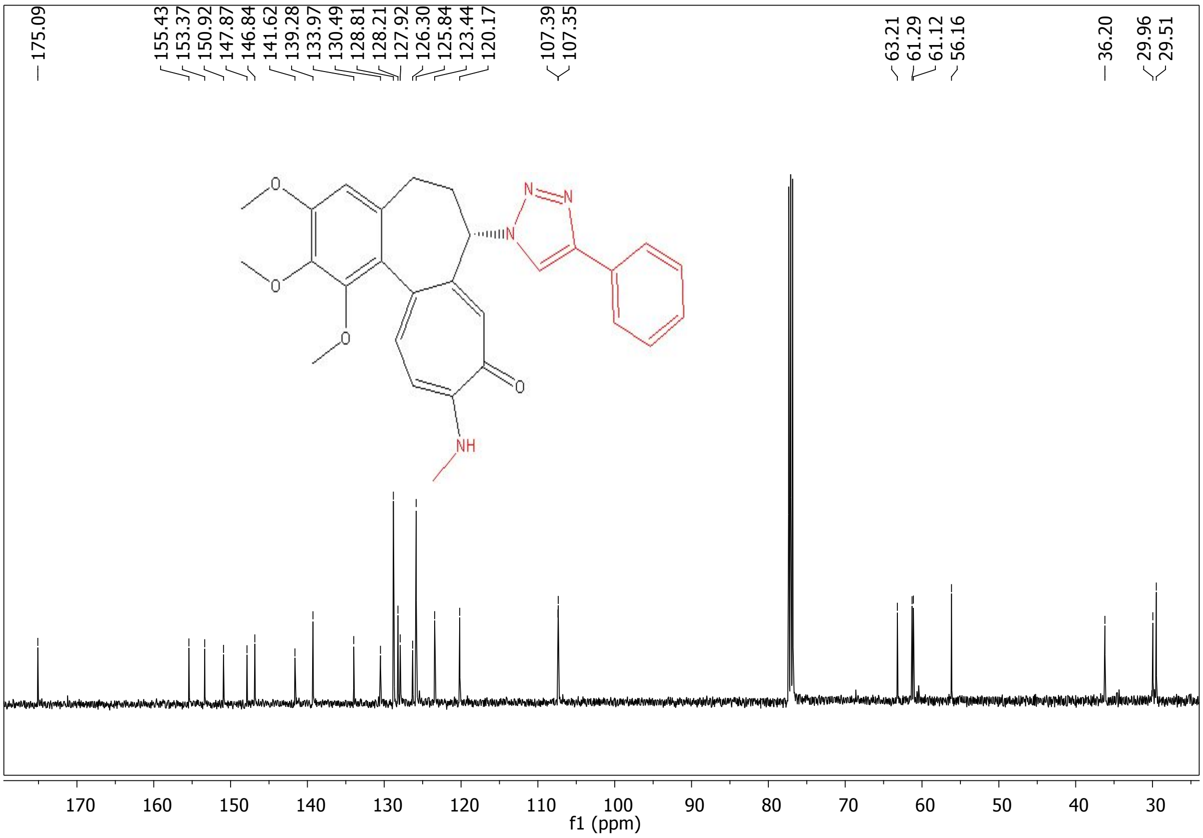

Figure S39. The ^{13}C NMR spectrum of **14** in $CDCl_3$.

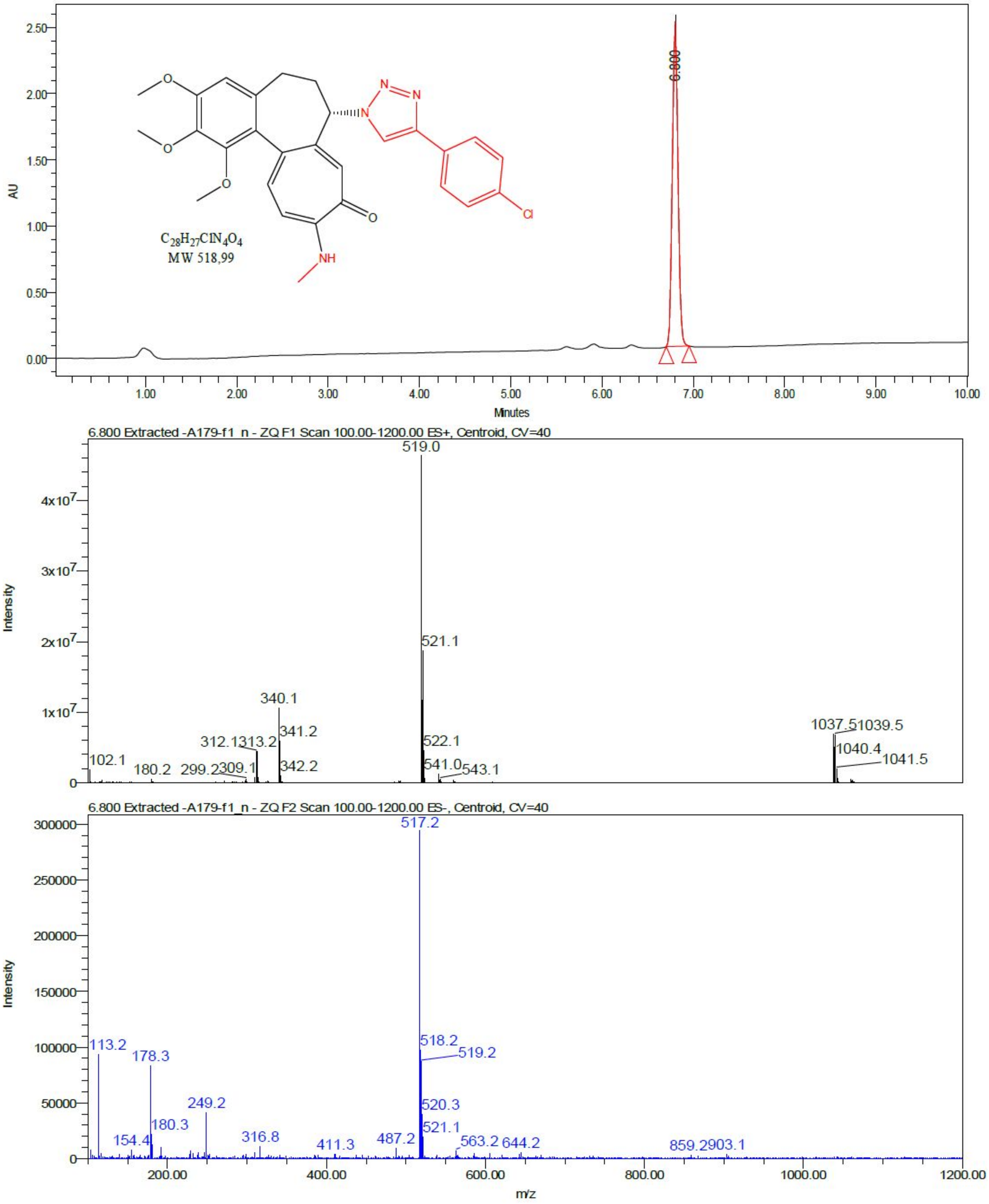

Figure S40. The LC-MS chromatogram and mass spectra of **15**.

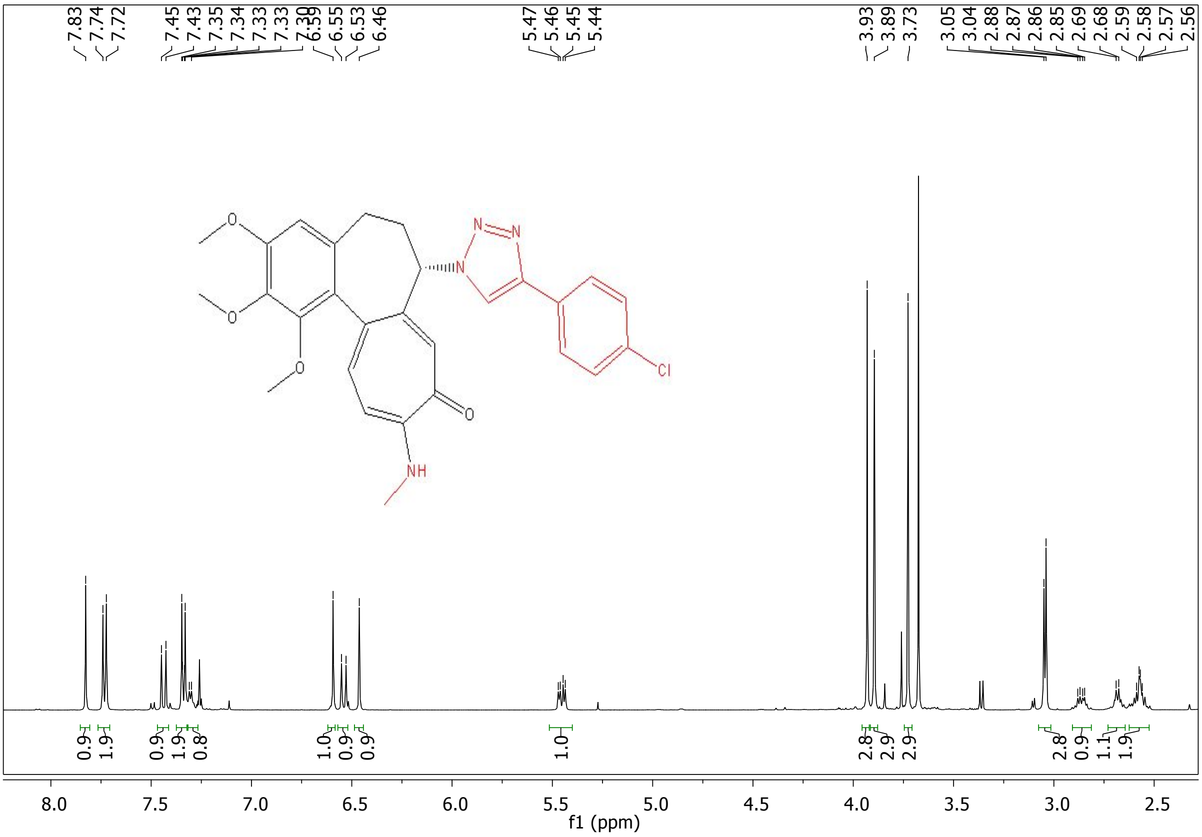

Figure S41. The ^{1}H NMR spectrum of **15** in $CDCl_3$.

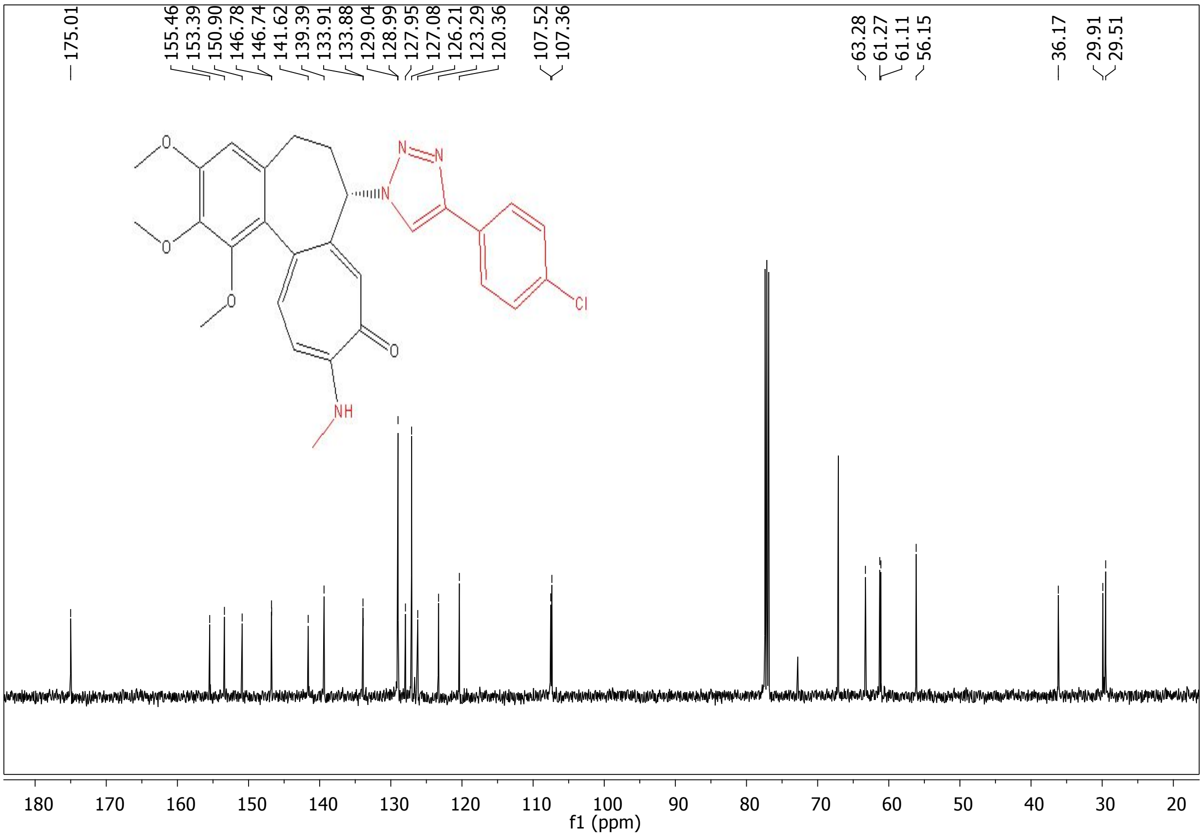

Figure S42. The ^{13}C NMR spectrum of **15** in $CDCl_3$.

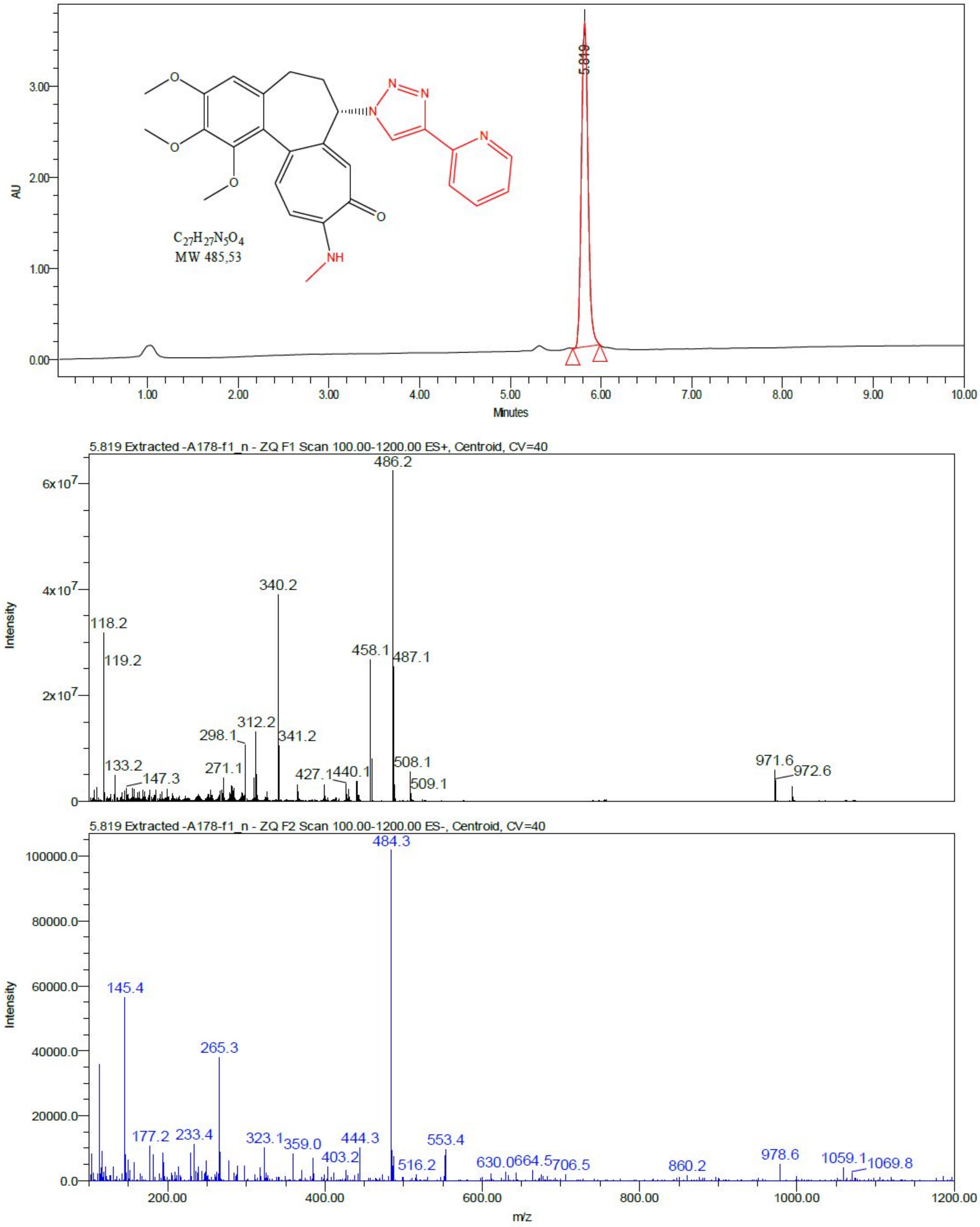

Figure S43. The LC-MS chromatogram and mass spectra of **16**.

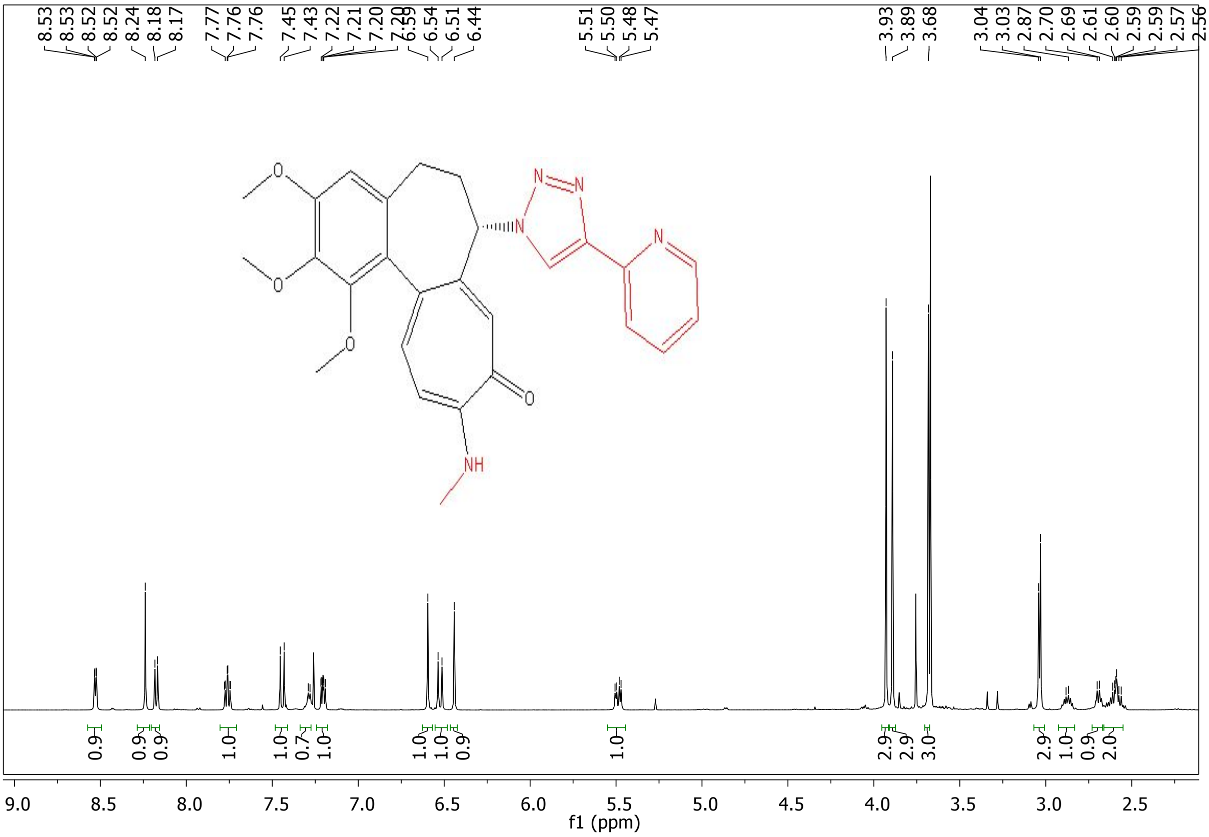

Figure S44. The ^{1}H NMR spectrum **16** in $CDCl_3$.

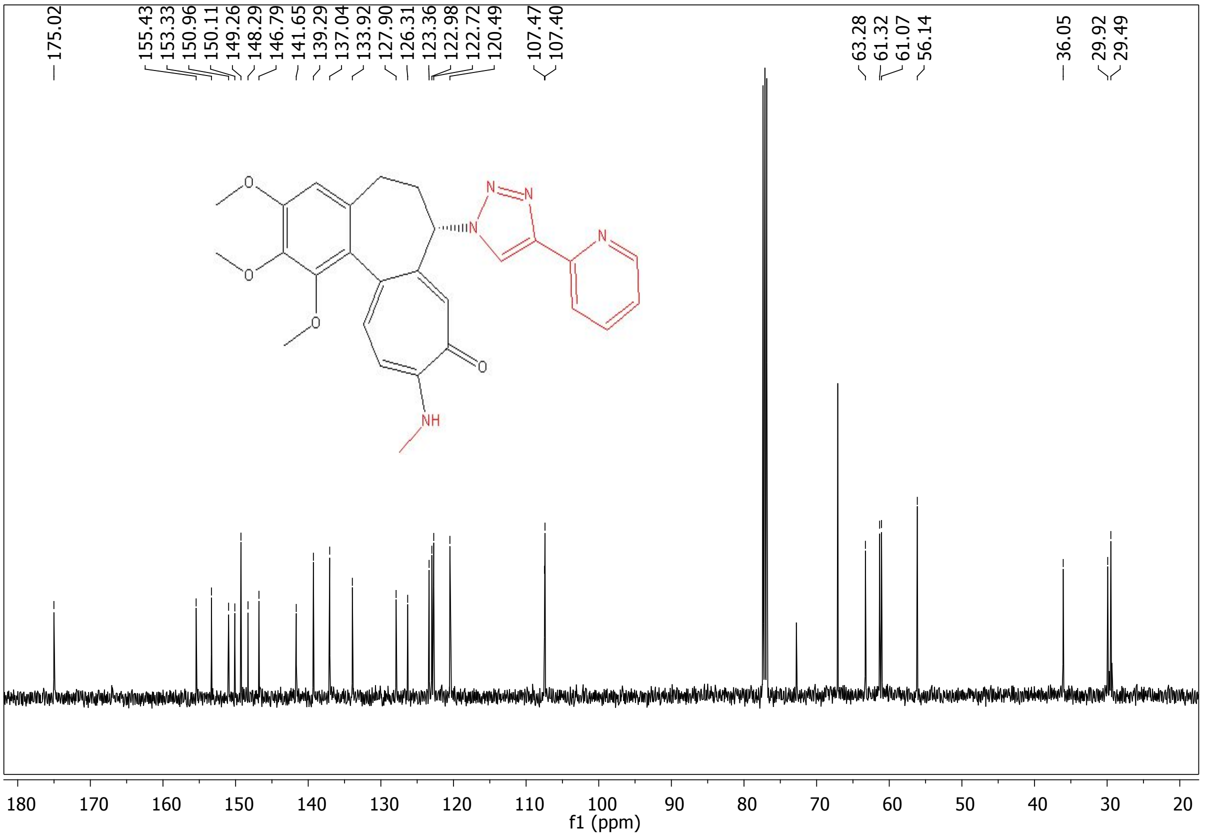

Figure S45. The ^{13}C NMR spectrum of **16** in $CDCl_3$.

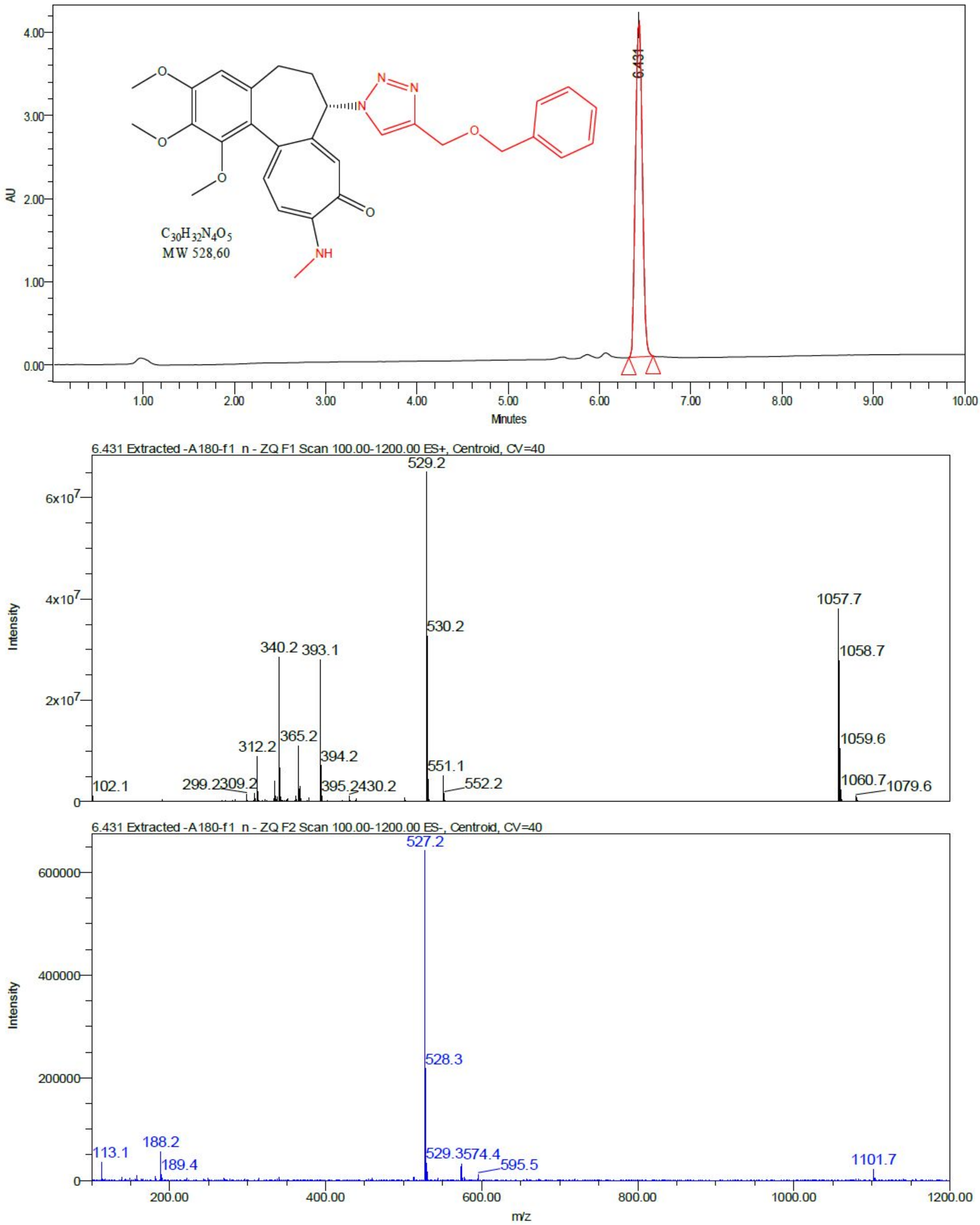

Figure S46. The LC-MS chromatogram and mass spectra of **17**.

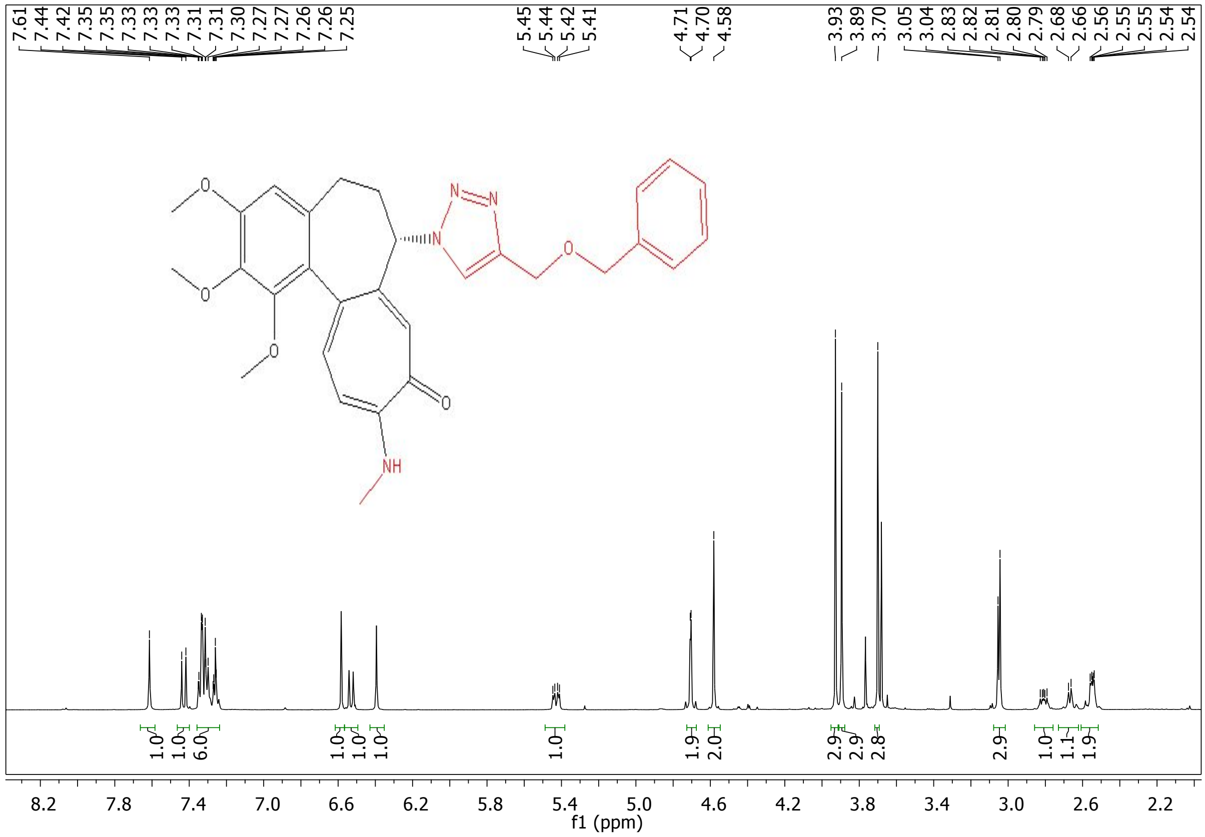

Figure S47. The ^{1}H NMR spectrum of **17** in $CDCl_3$.

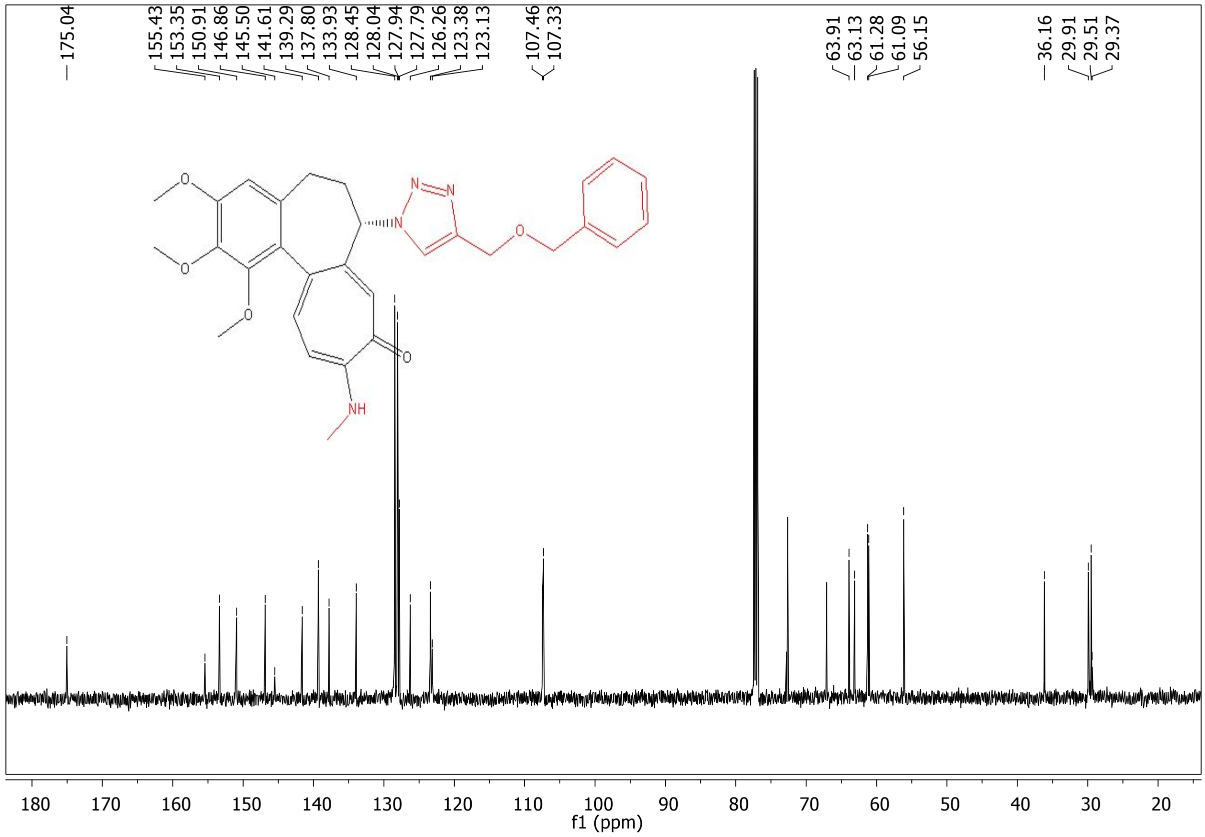

Figure S48. The ^{13}C NMR spectrum of **17** in $CDCl_3$.

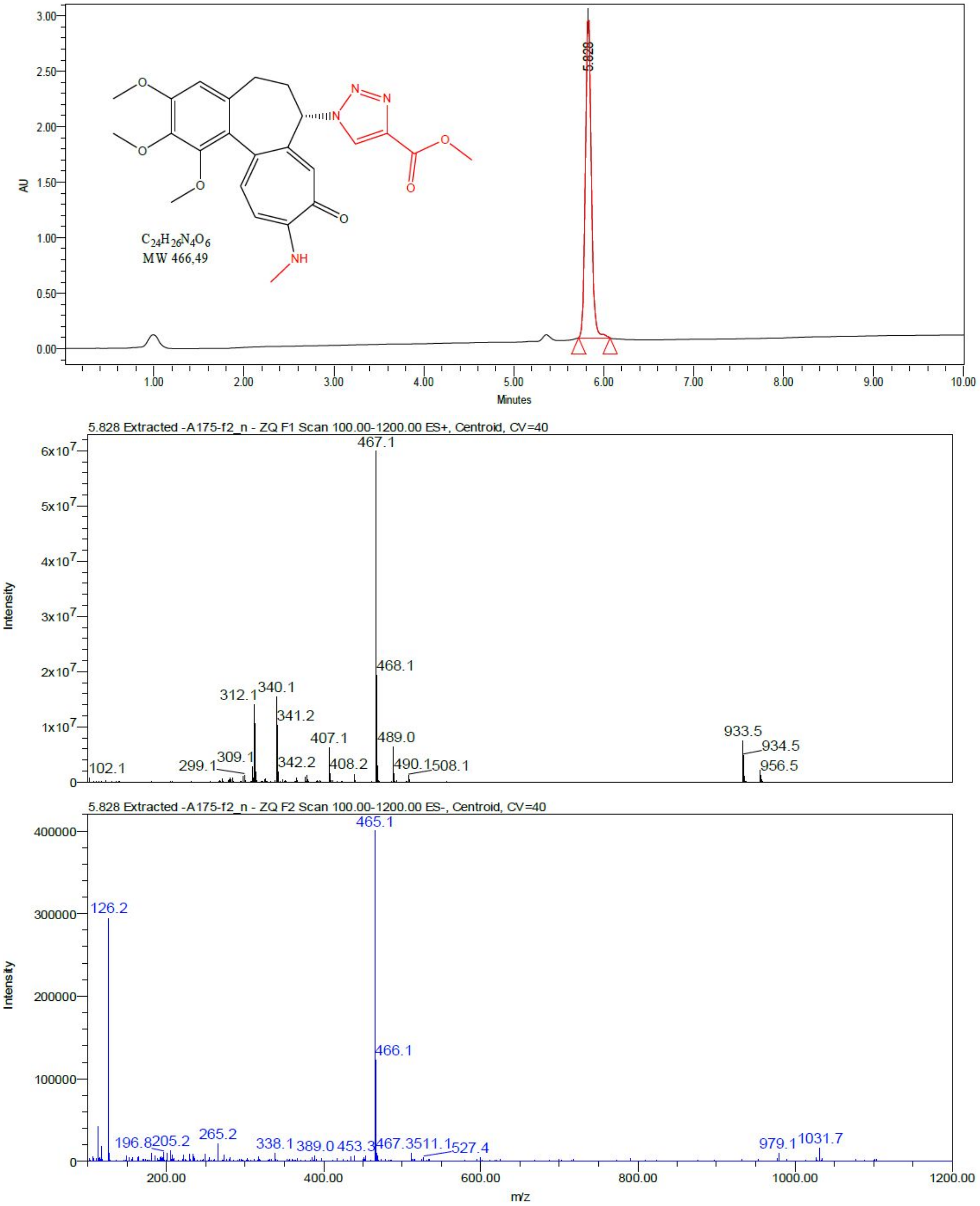

Figure S49. The LC-MS chromatogram and mass spectra of **18**.

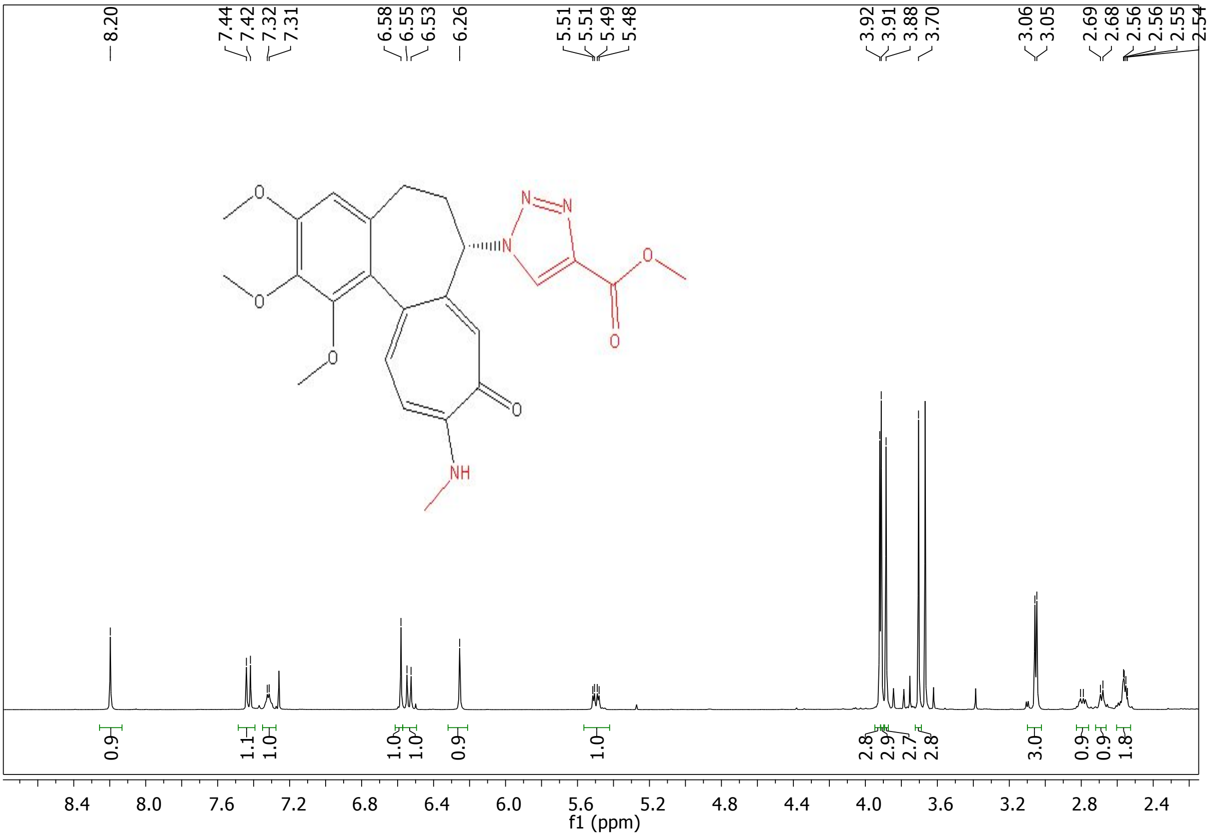

Figure S50. The ^{1}H NMR spectrum of **18** in $CDCl_3$.

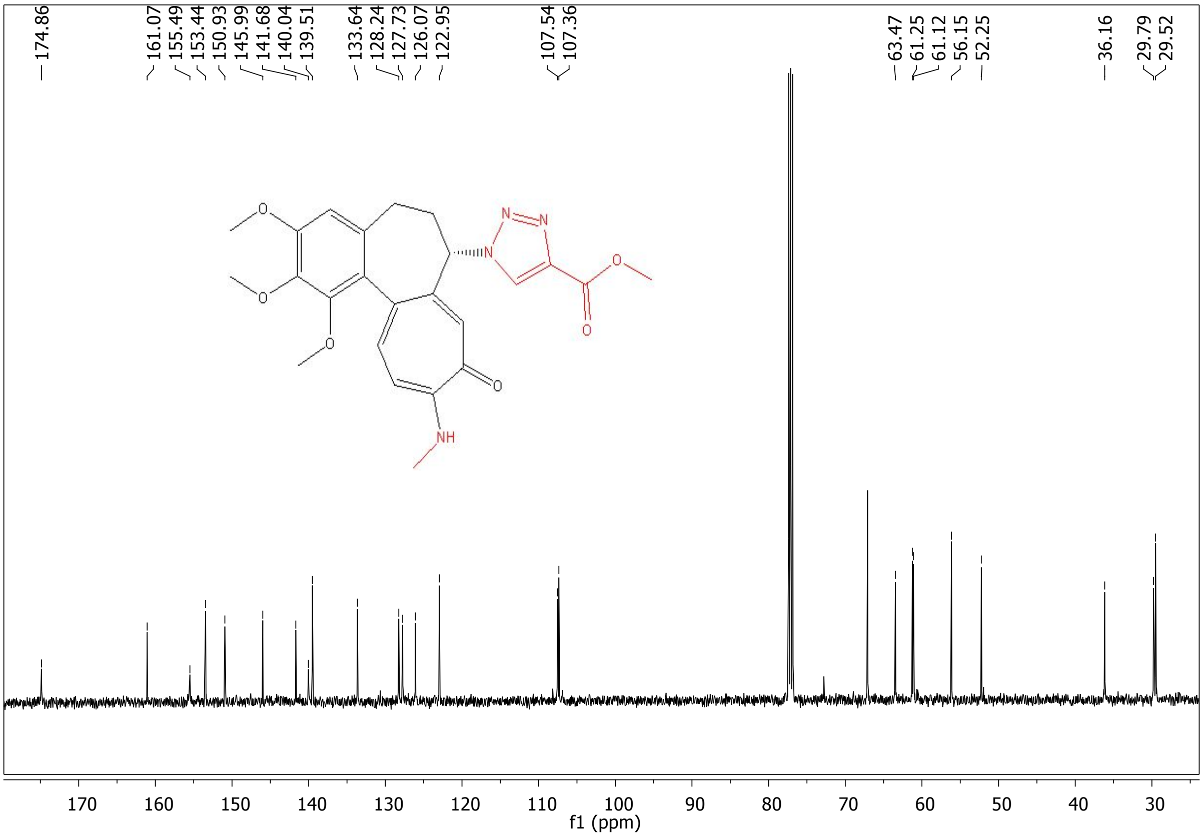

Figure S51. The ^{13}C NMR spectrum of **18** in $CDCl_3$.

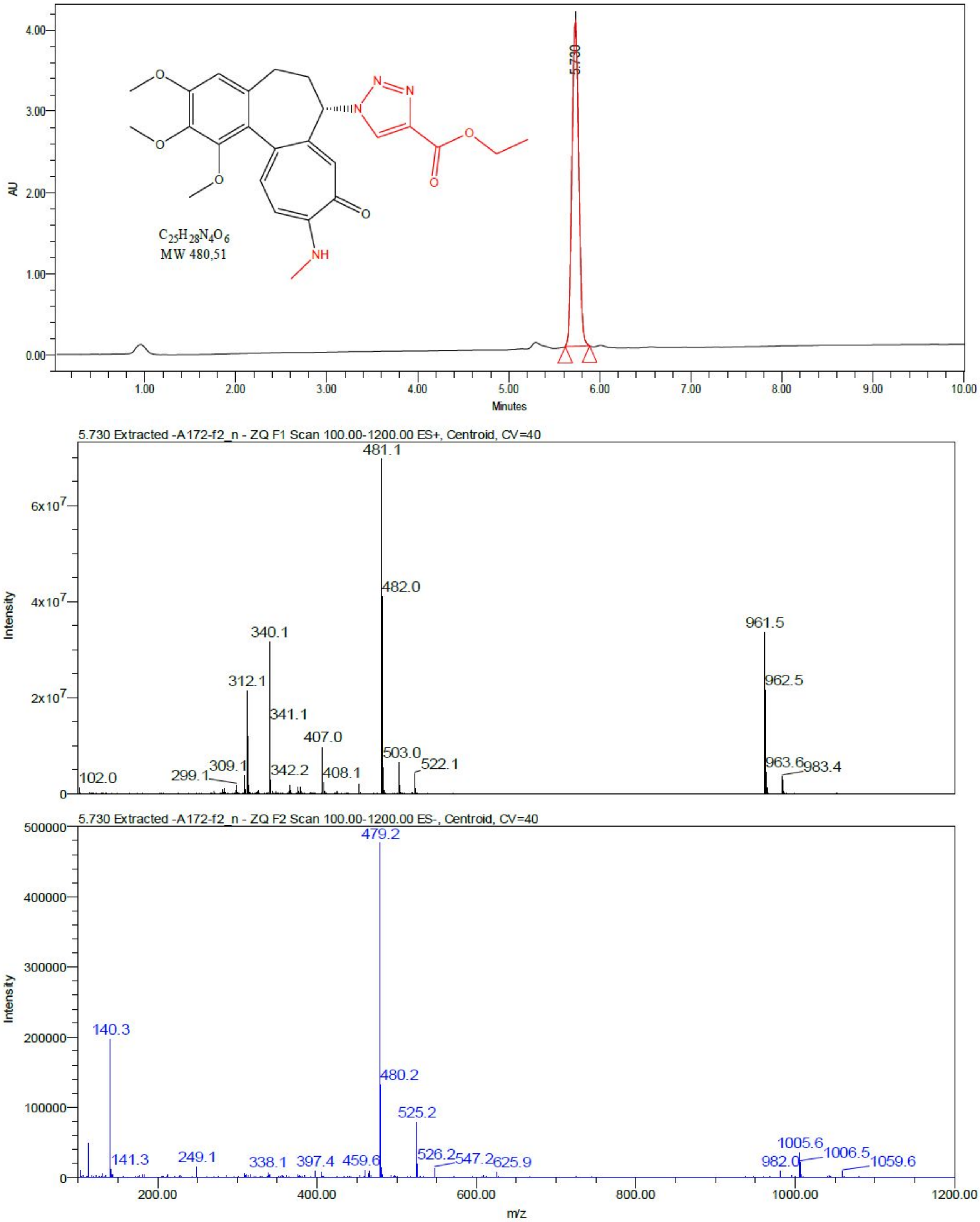

Figure S52. The LC-MS chromatogram and mass spectra of **19**.

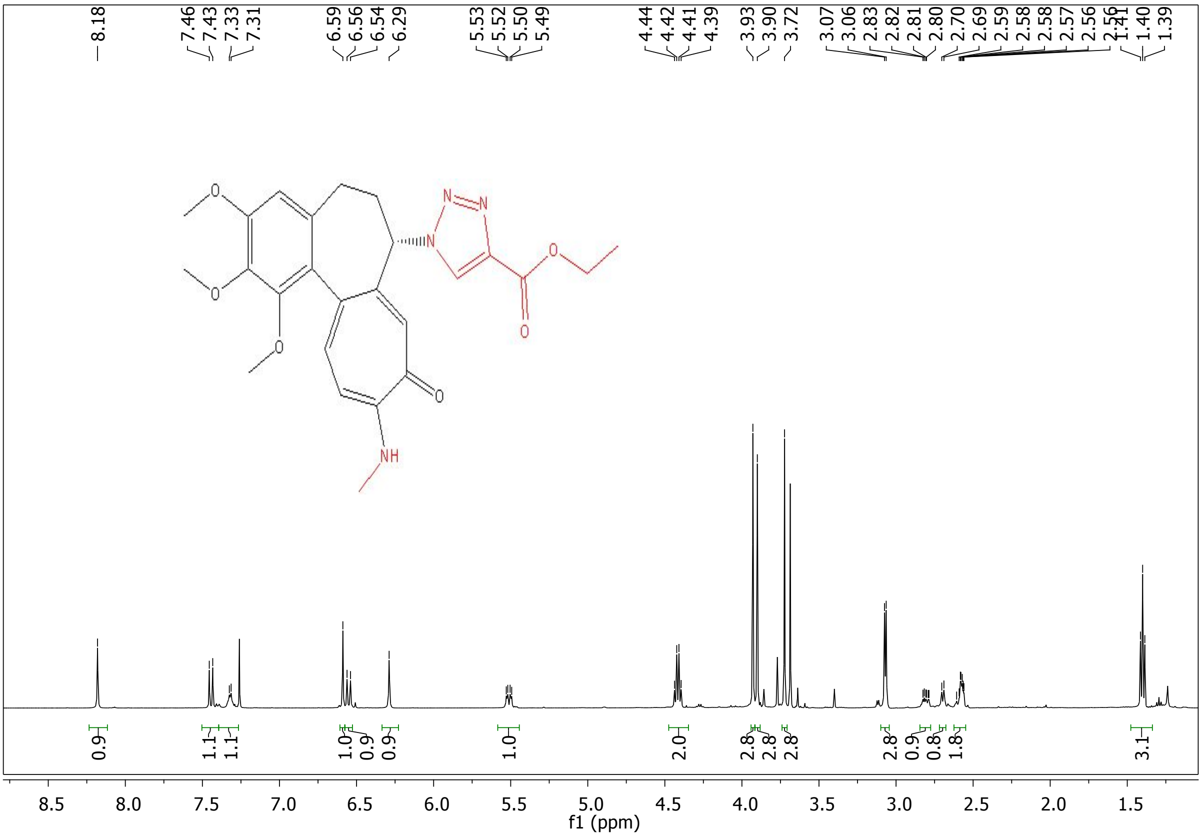

Figure S53. The ^{1}H NMR spectrum of **19** in $CDCl_3$.

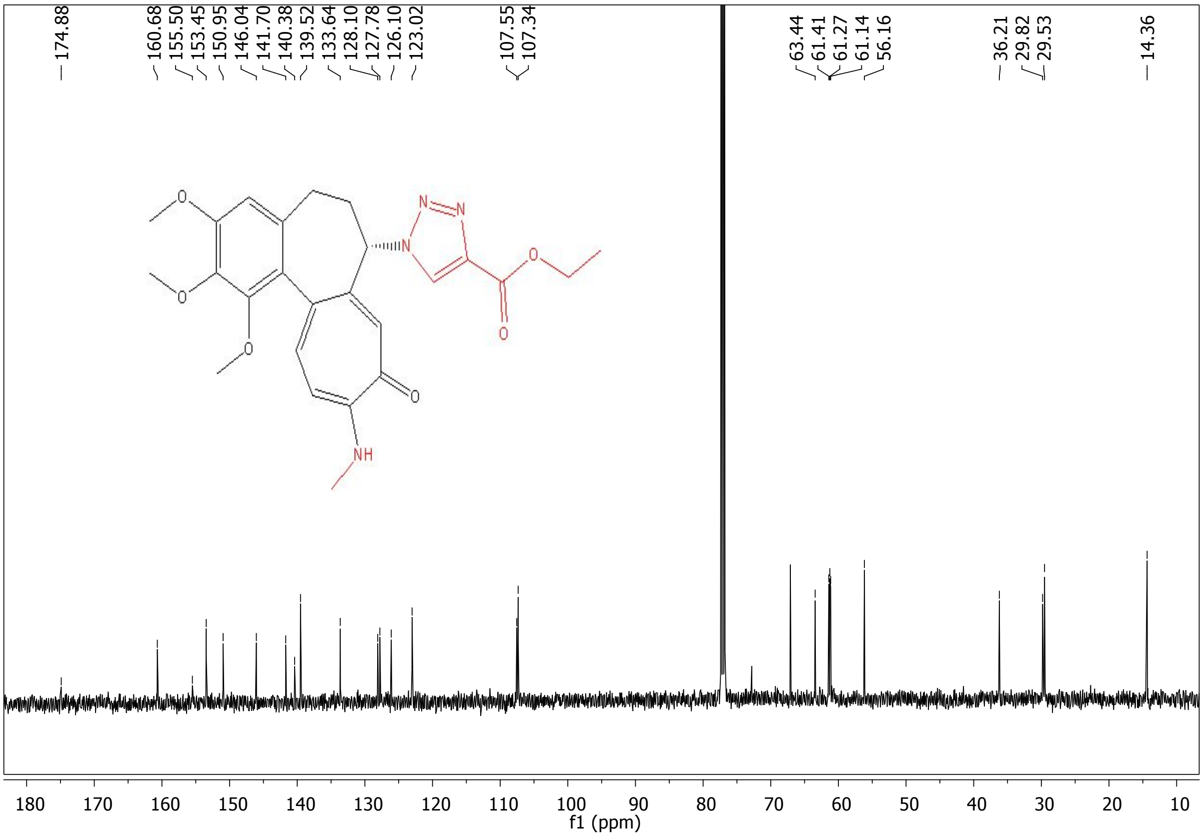

Figure S54. The ^{13}C NMR spectrum of **19** in $CDCl_3$.

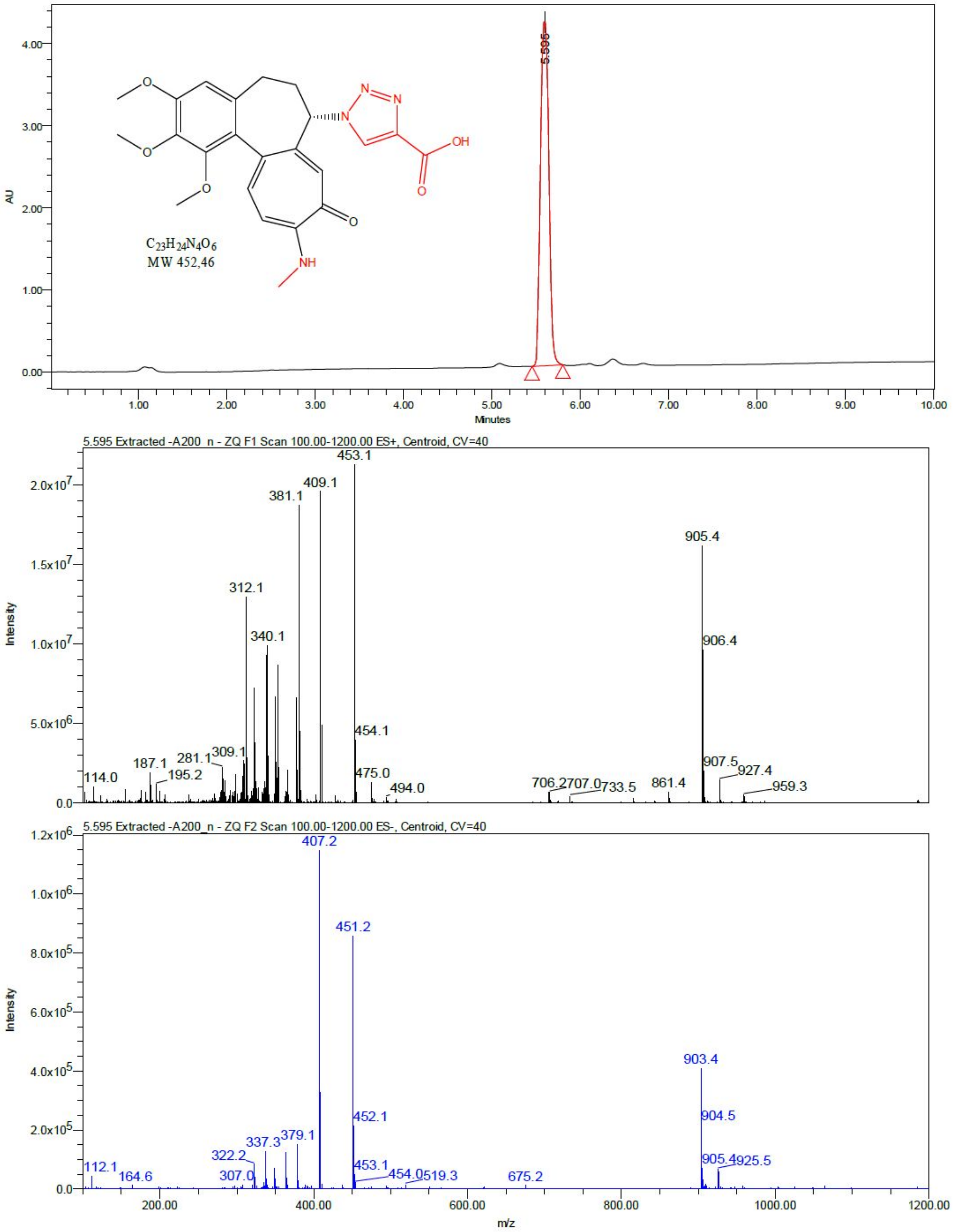

Figure S55. The LC-MS chromatogram and mass spectra of **20**.

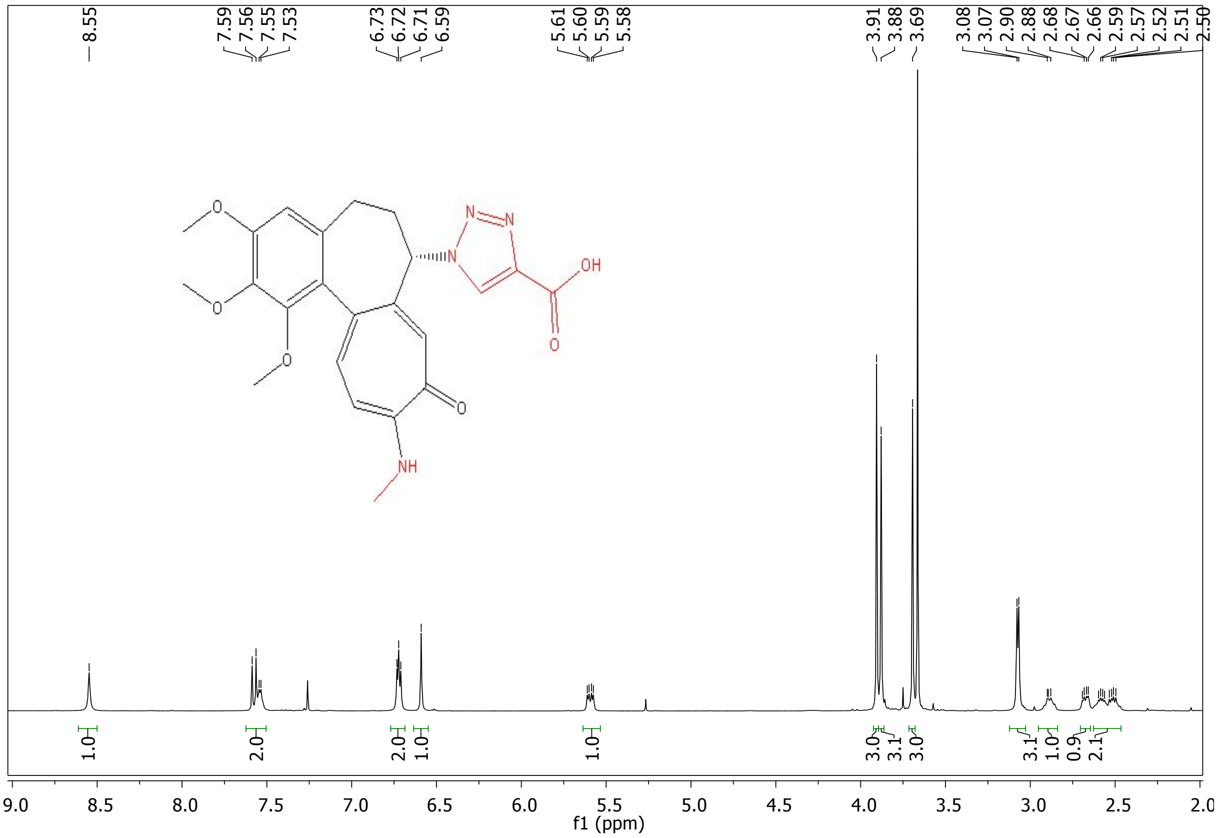

Figure S56. The ^{1}H NMR spectrum of **20** in $CDCl_3$.

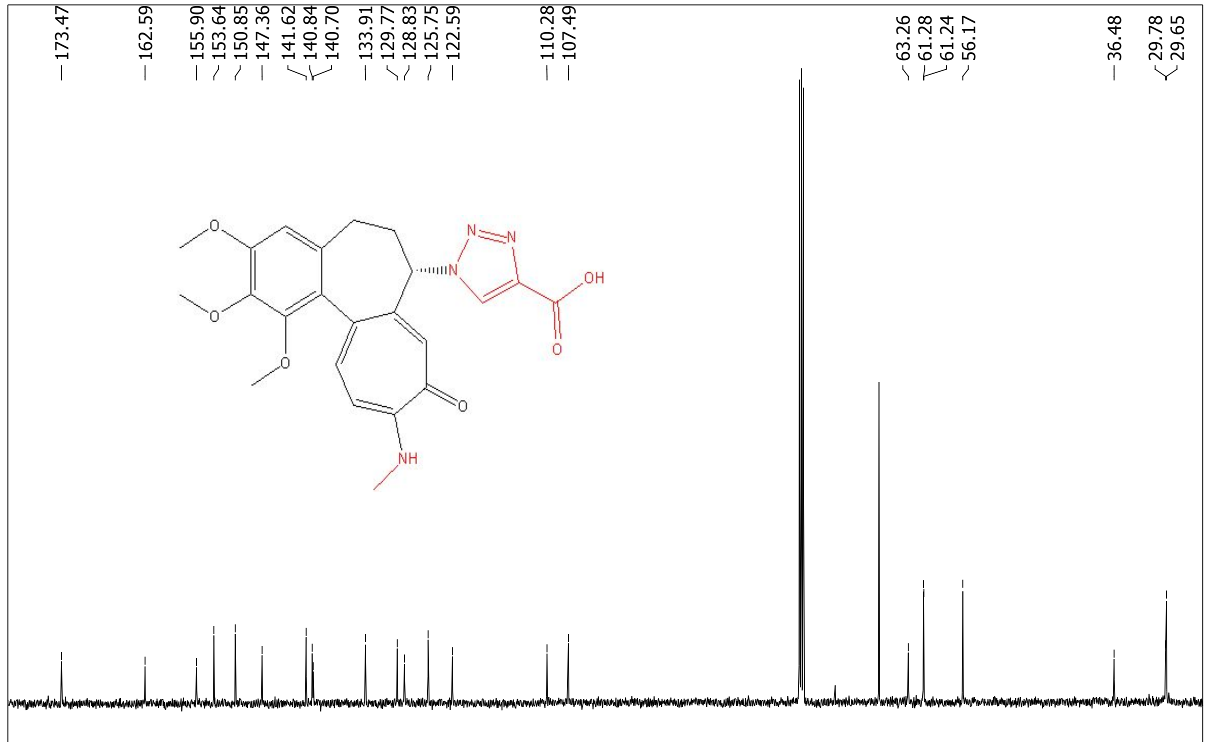

Figure S57. The ^{13}C NMR spectrum of **20** in $CDCl_3$.

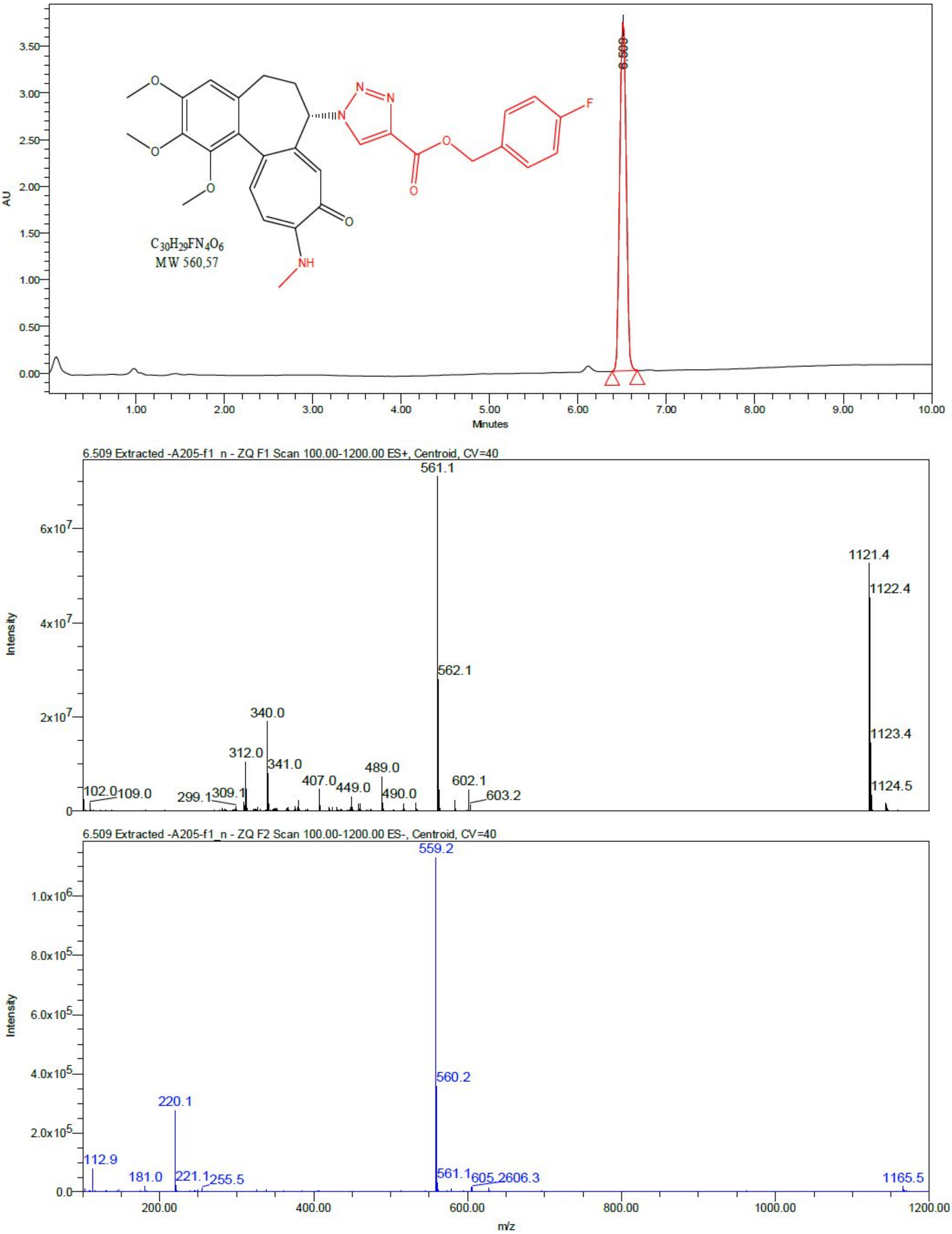

Figure S58. The LC-MS chromatogram and mass spectra of **21**.

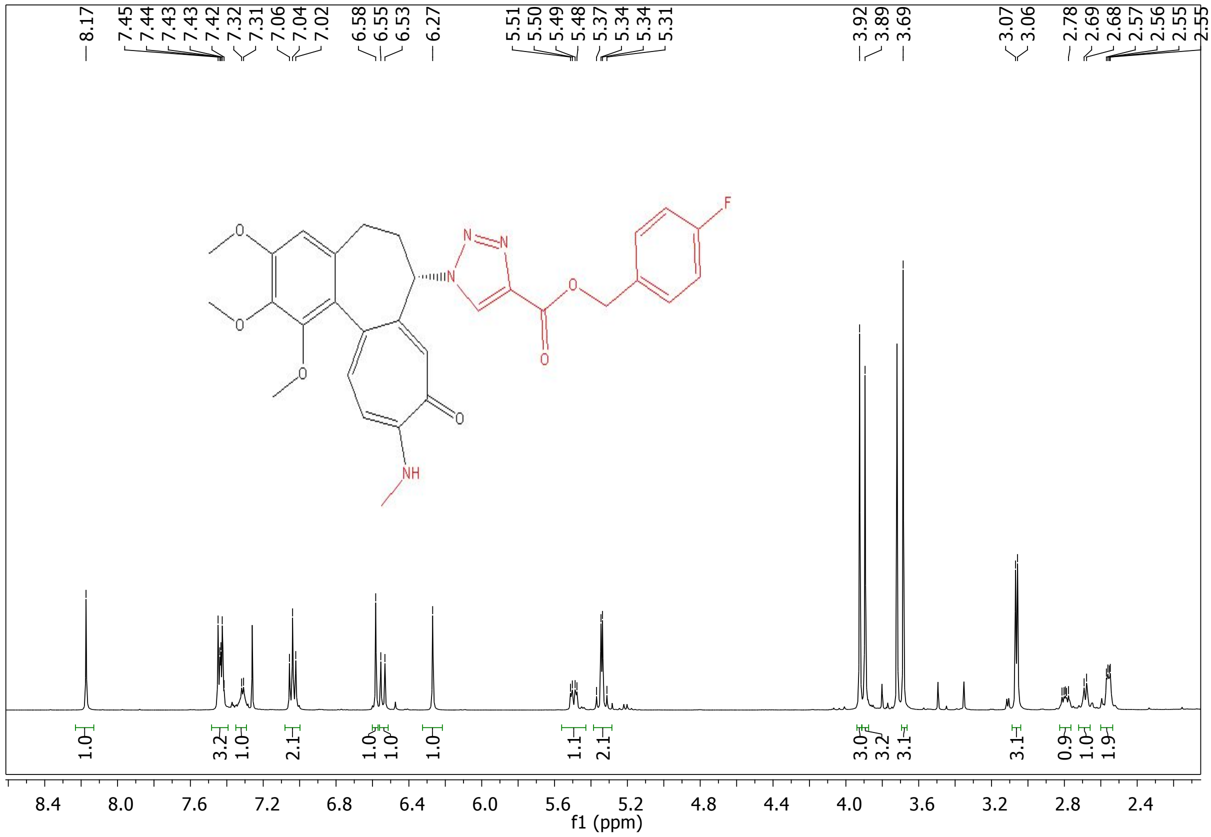

Figure S59. The ^{1}H NMR spectrum of **21** in $CDCl_3$.

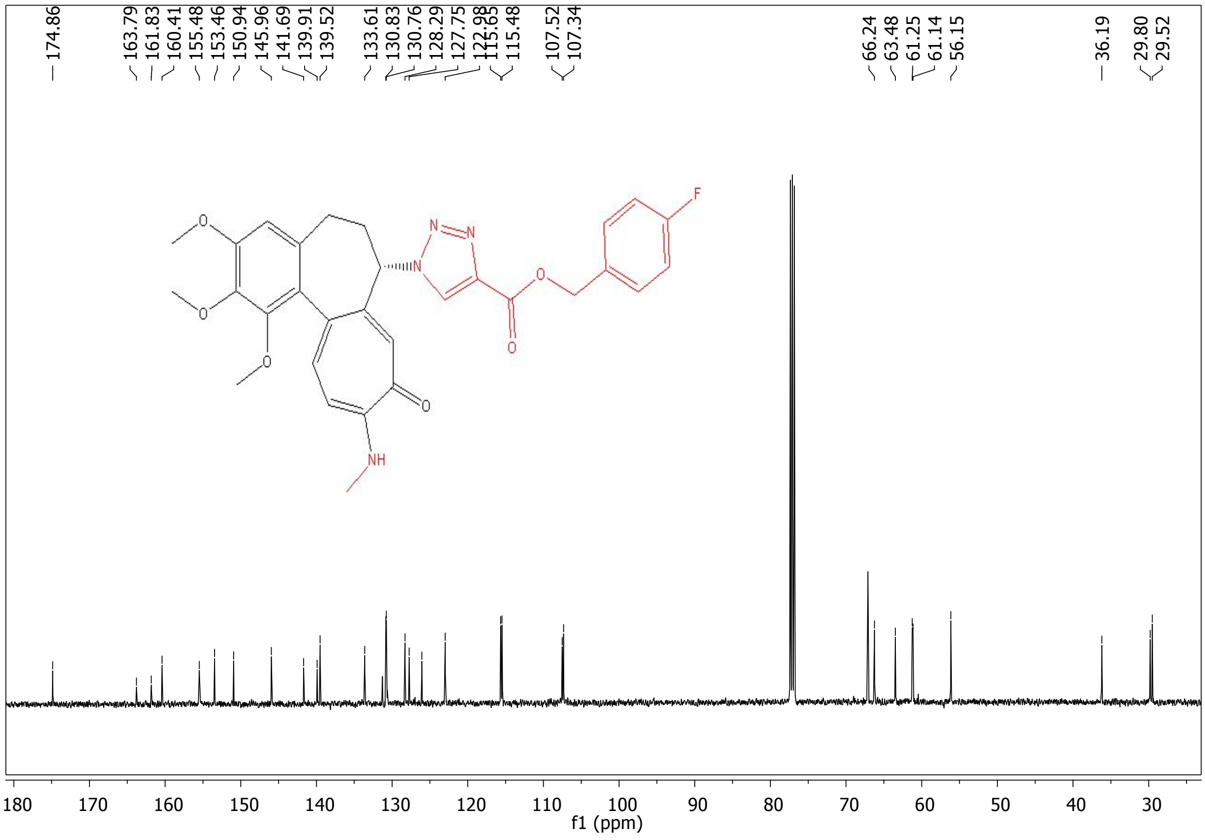

Figure S60. The ^{13}C NMR spectrum of **21** in $CDCl_3$.

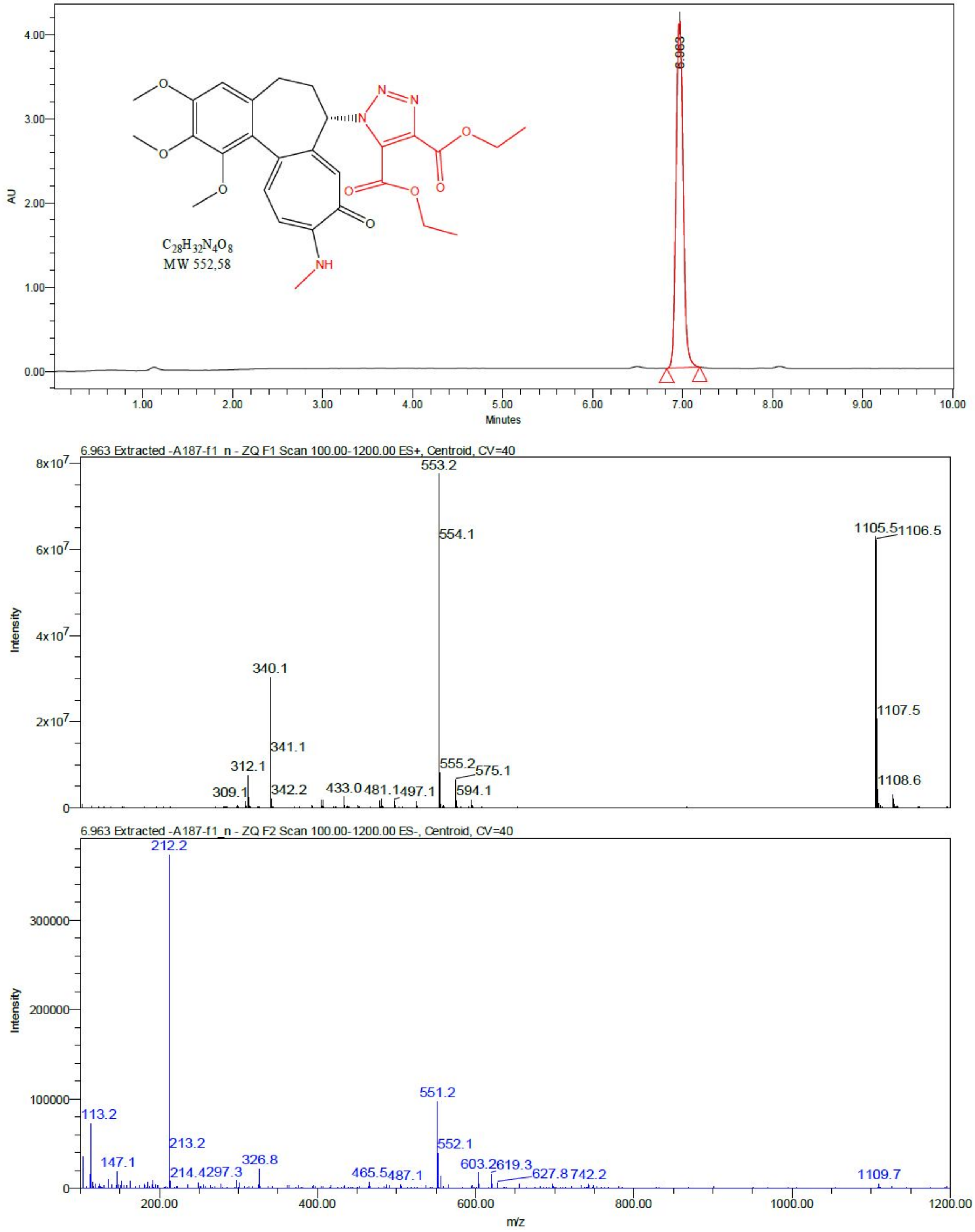

Figure S61. The LC-MS chromatogram and mass spectra of **22**.

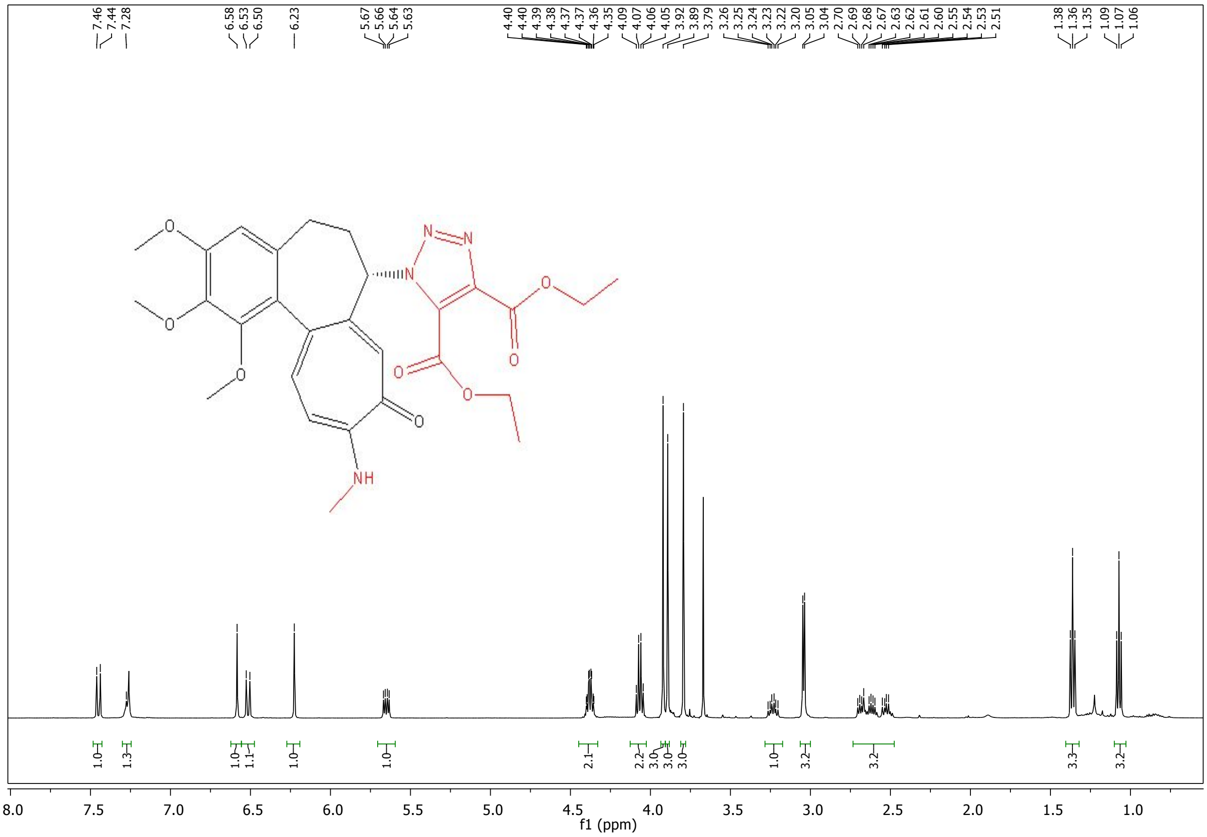

Figure S62. The ^{1}H NMR spectrum of **22** in $CDCl_3$.

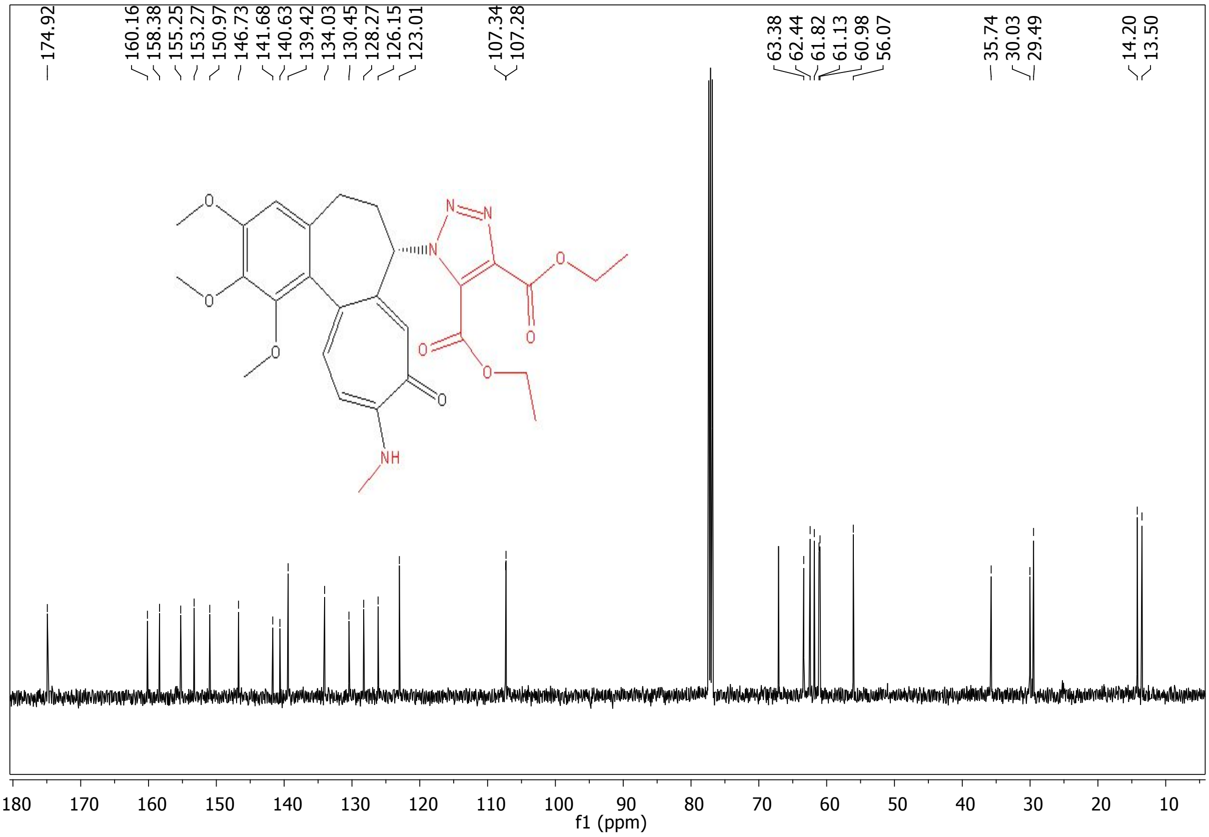

Figure S63. The ^{13}C NMR spectrum of **22** in $CDCl_3$.

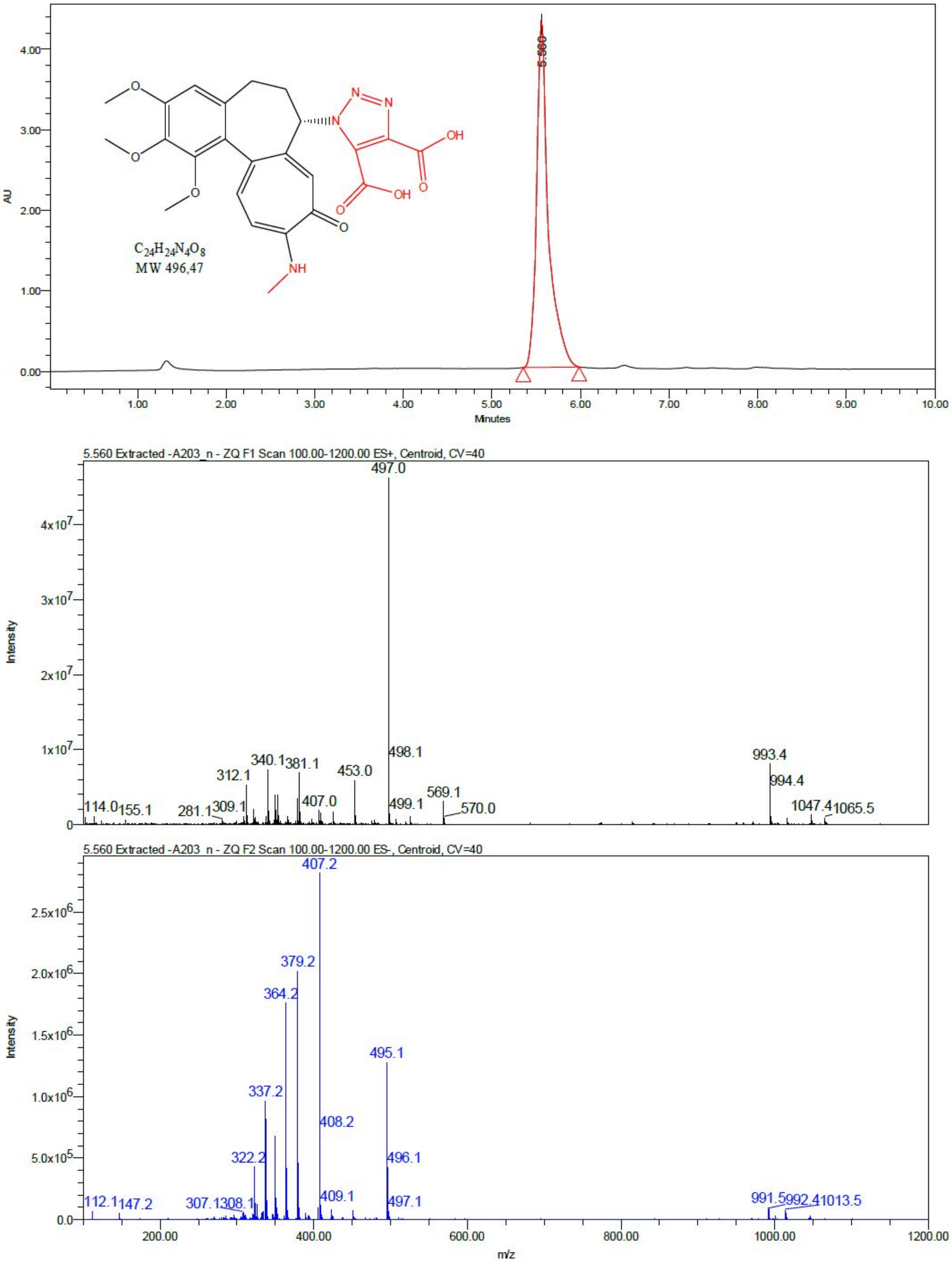

Figure S64. The LC-MS chromatogram and mass spectra of **23**.

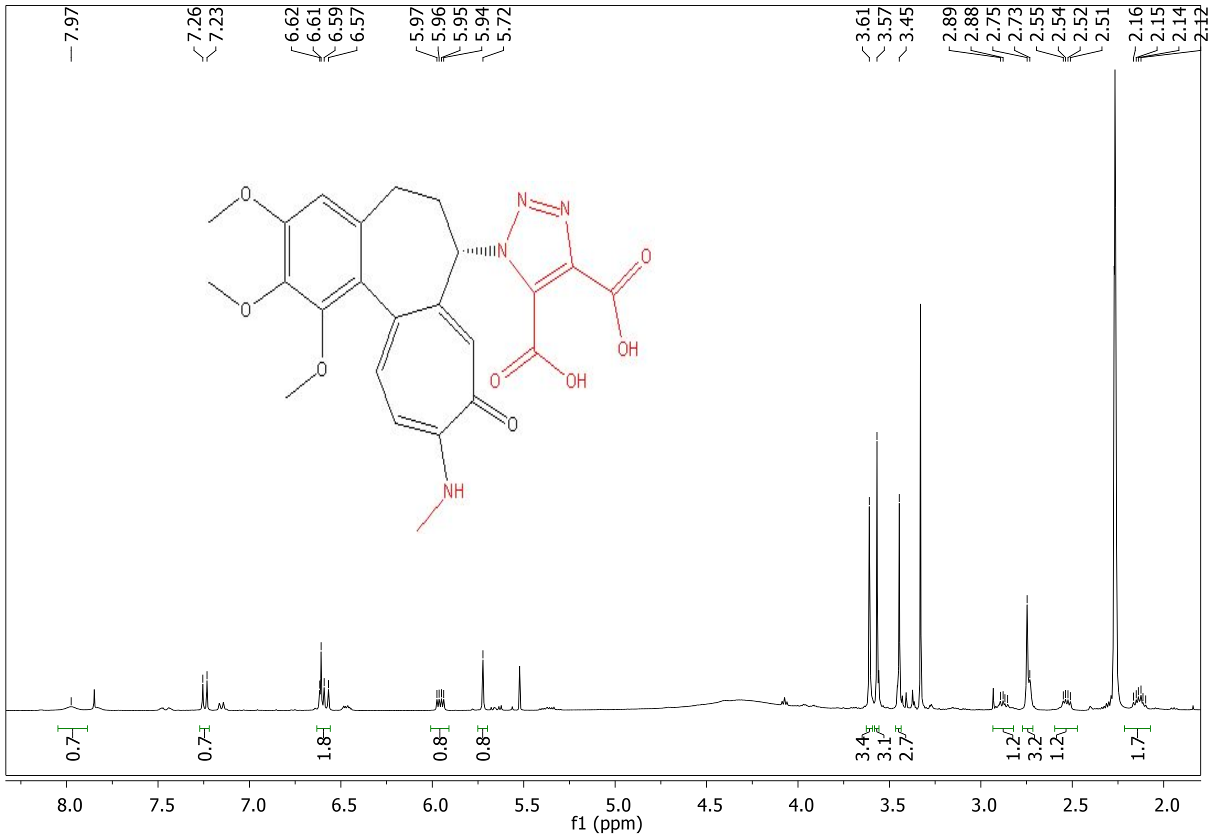

Figure S65. The ^{1}H NMR spectrum of **23** in $(CD_3)_2SO$

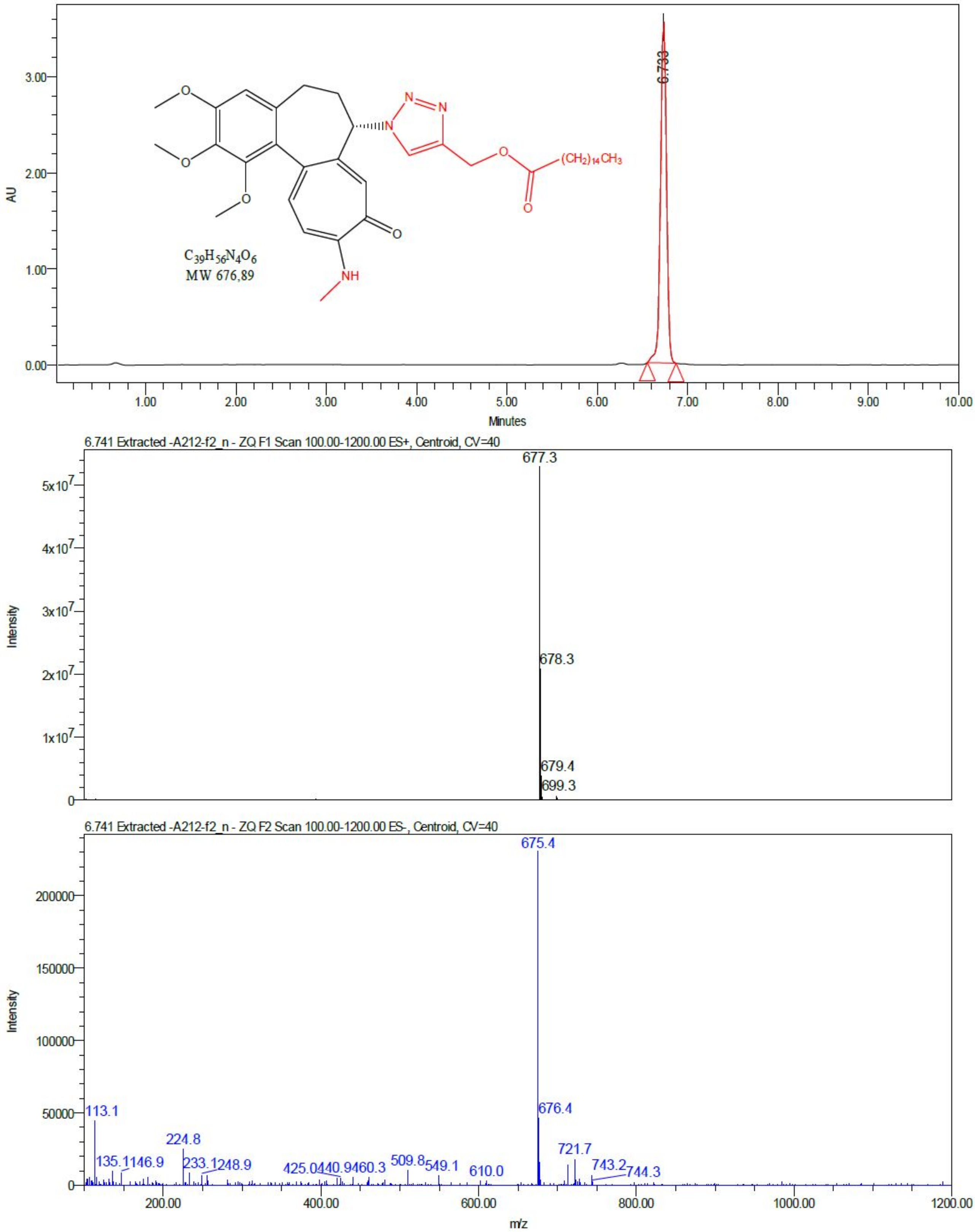

Figure S66. The LC-MS chromatogram and mass spectra of **24**.

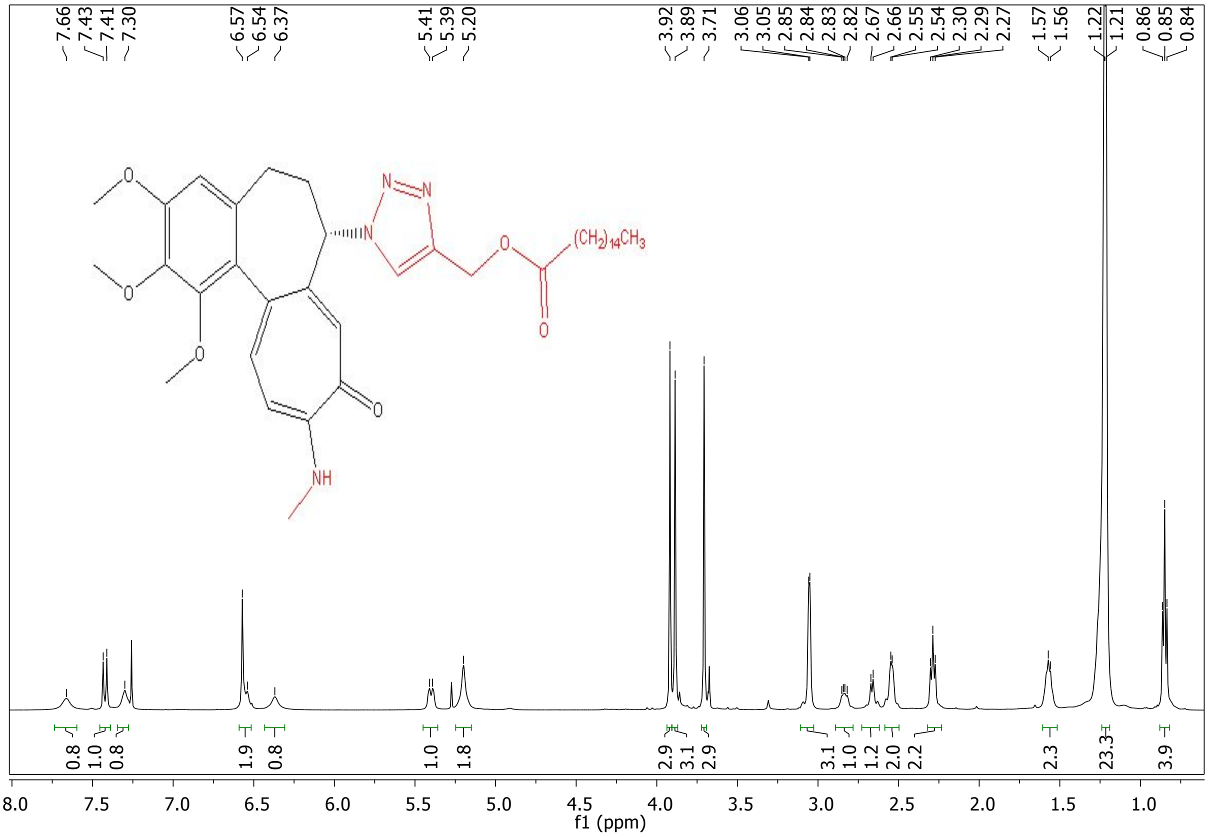

Figure S67. The ^{1}H NMR spectrum of **24** in $CDCl_3$.

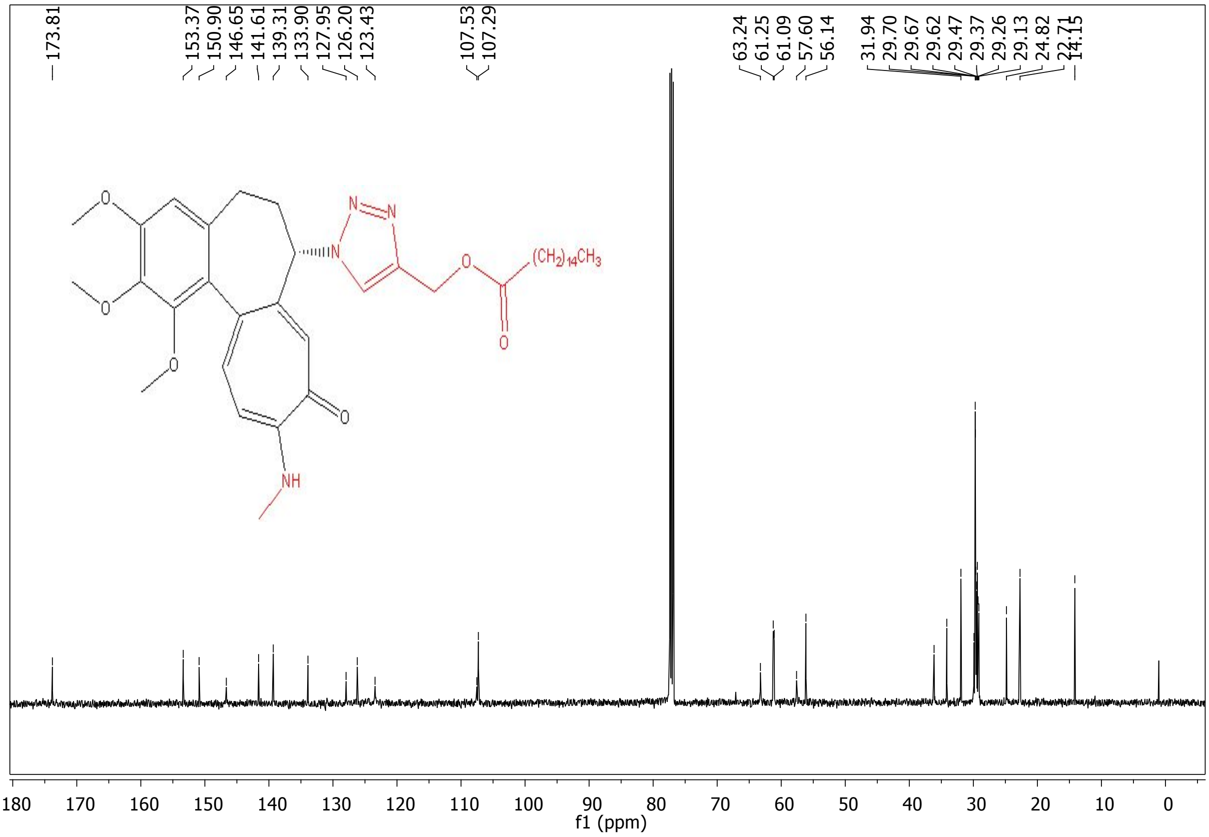

Figure S68. The ^{13}C NMR spectrum of **24** in $CDCl_3$.

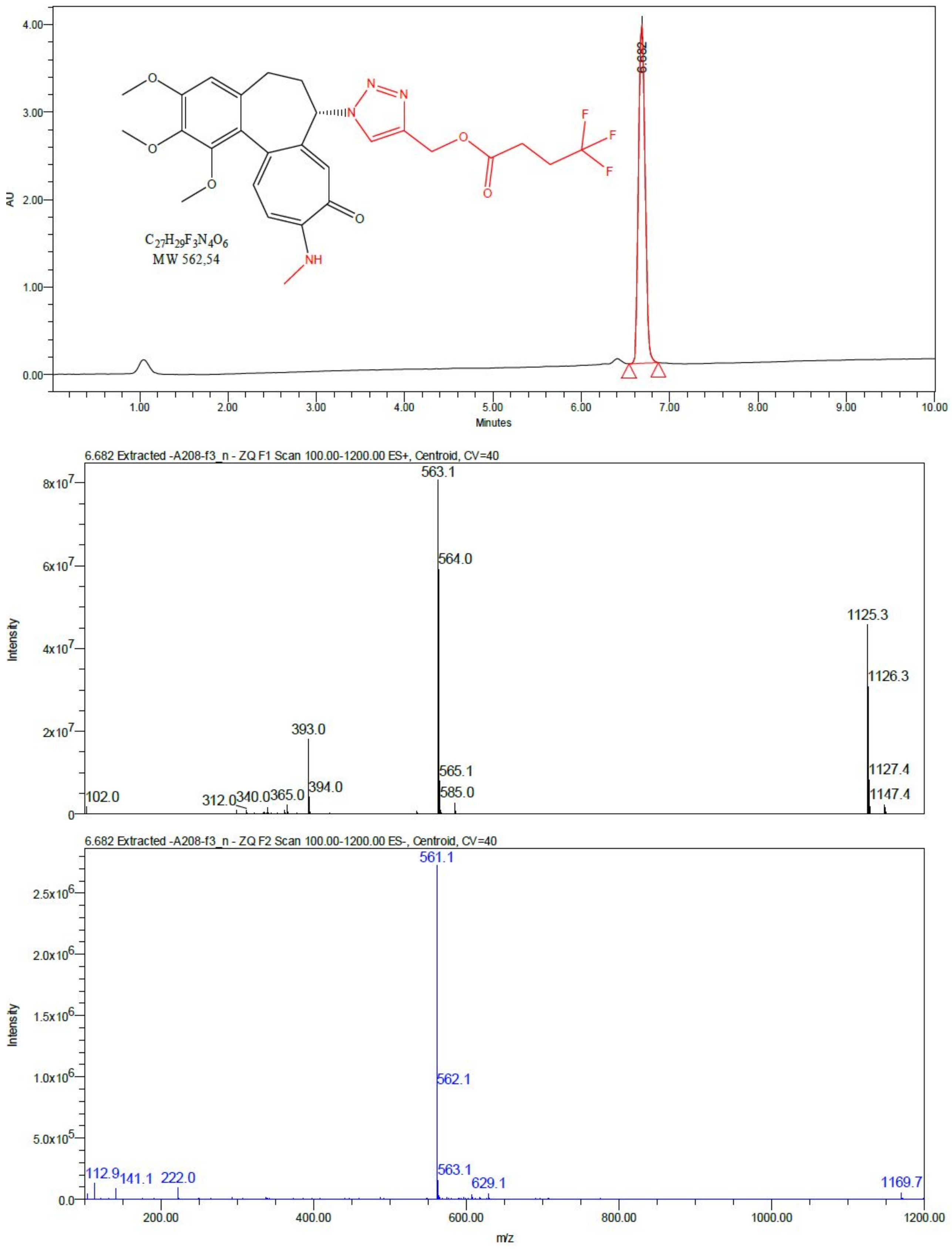

Figure S69. The LC-MS chromatogram and mass spectra of **25**.

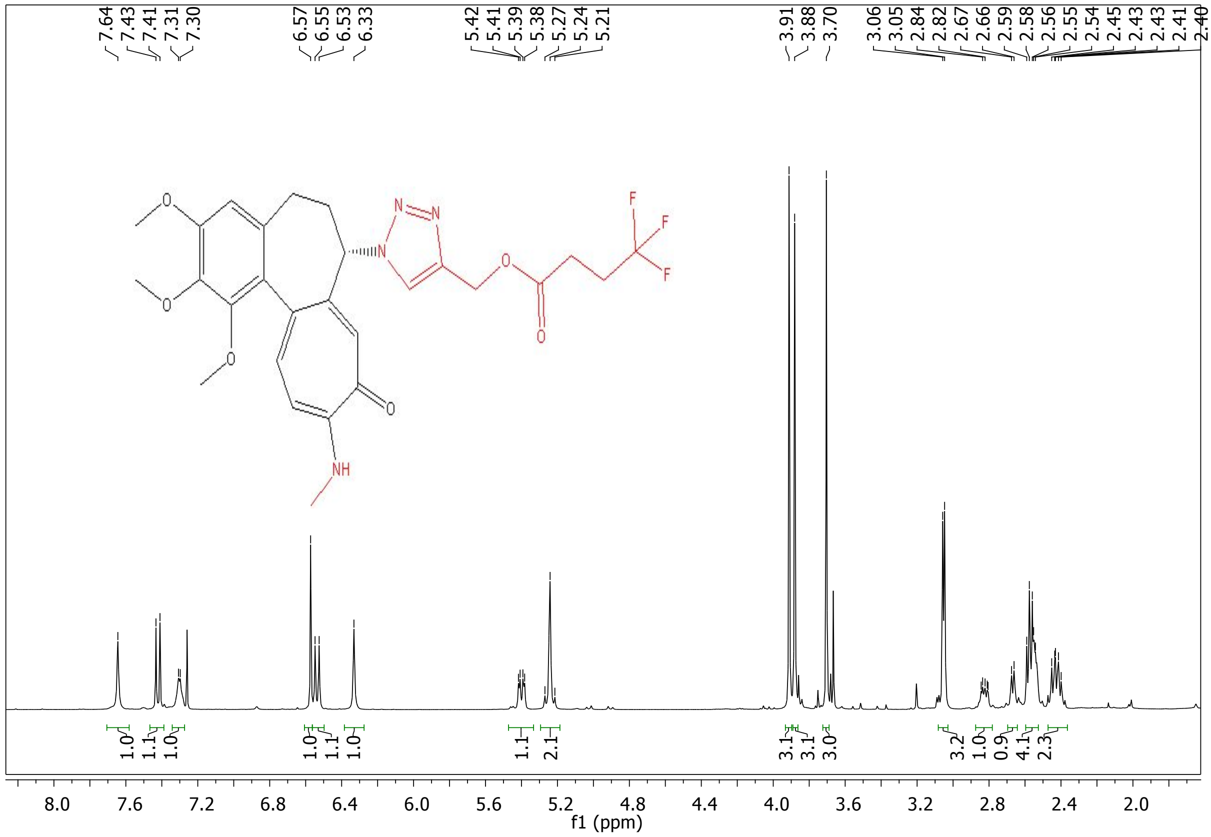

Figure S70. The ^{1}H NMR spectrum of **25** in $CDCl_3$.

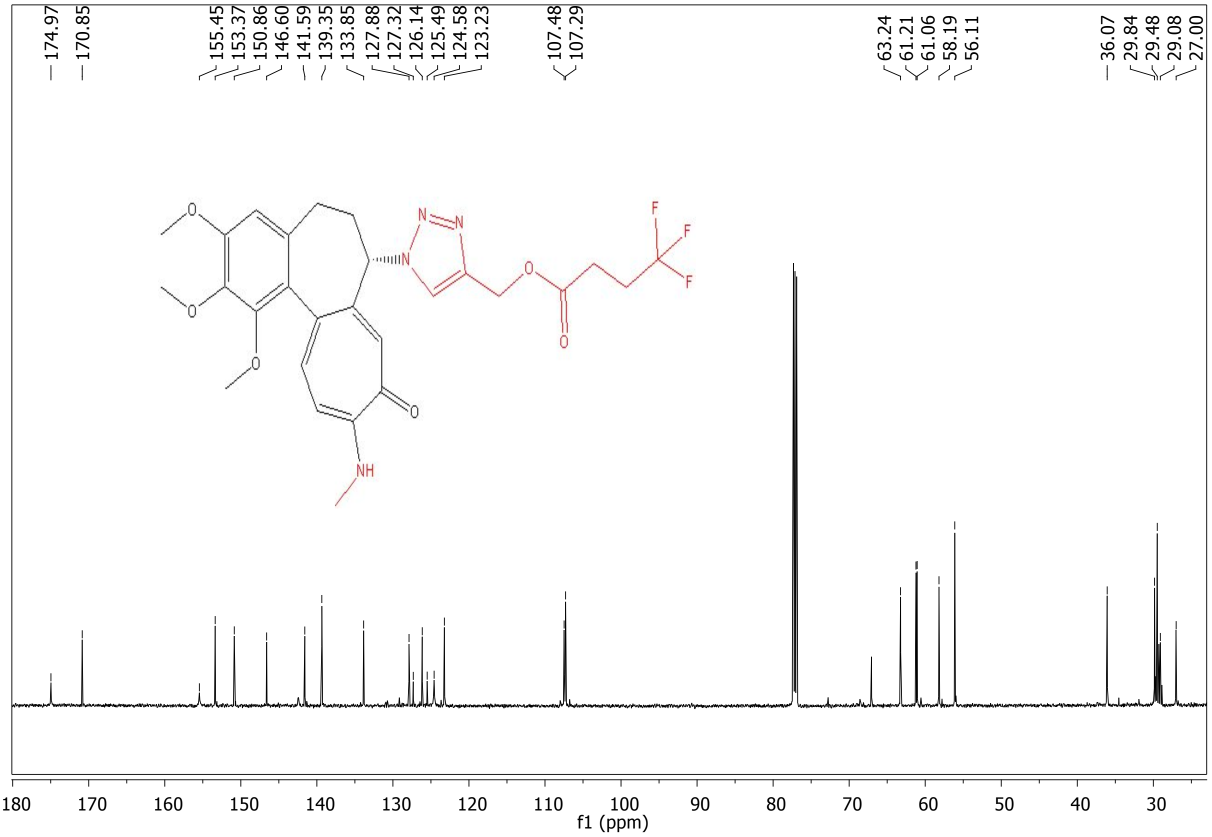

Figure S71. The ^{13}C NMR spectrum of **25** in $CDCl_3$.

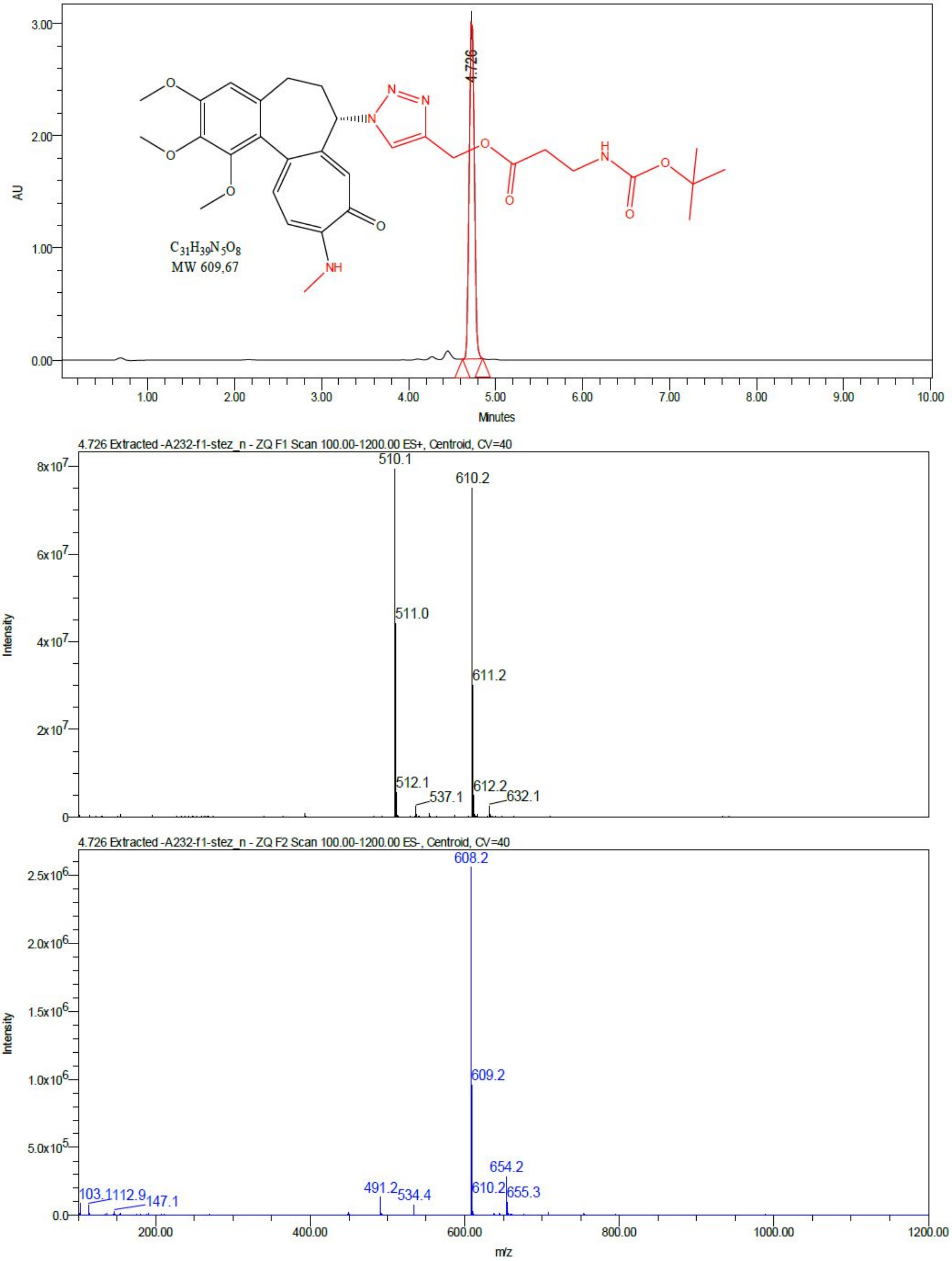

Figure S72. The LC-MS chromatogram and mass spectra of **26**.

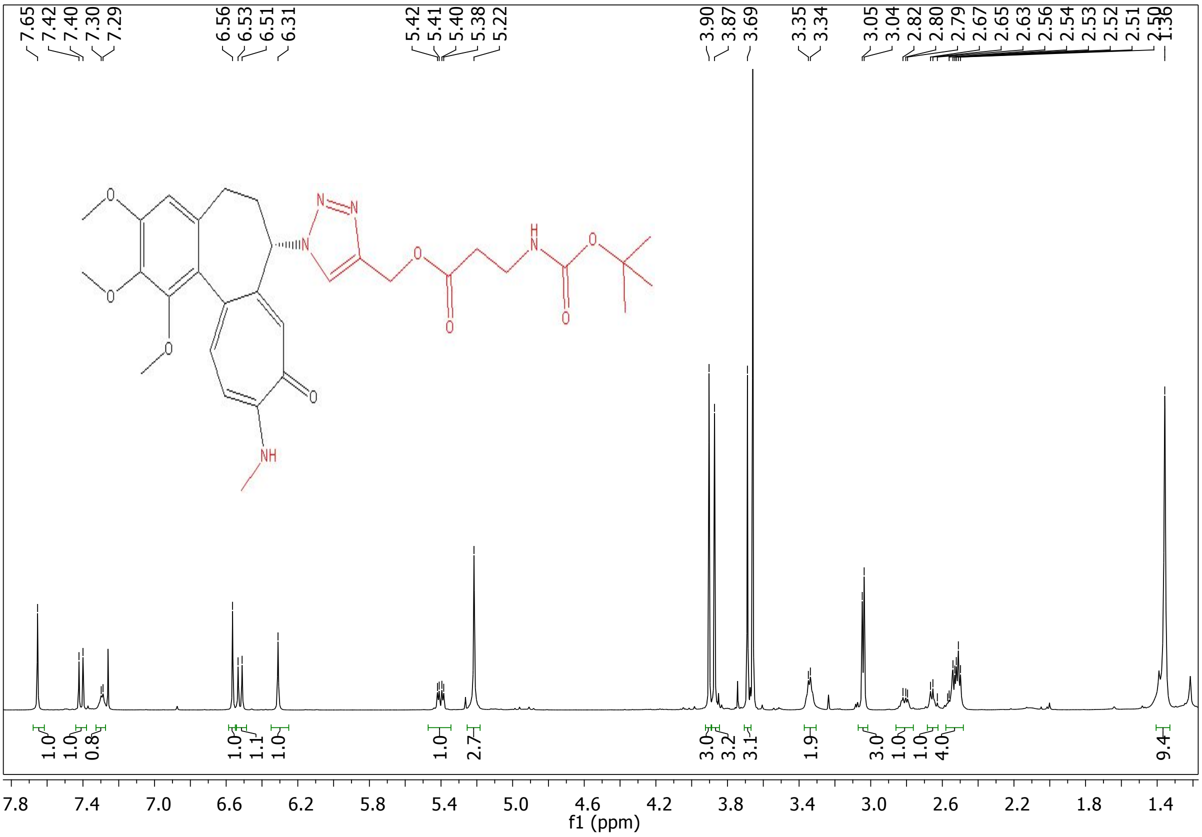

Figure S73. The ^{1}H NMR spectrum of **26** in $CDCl_3$.

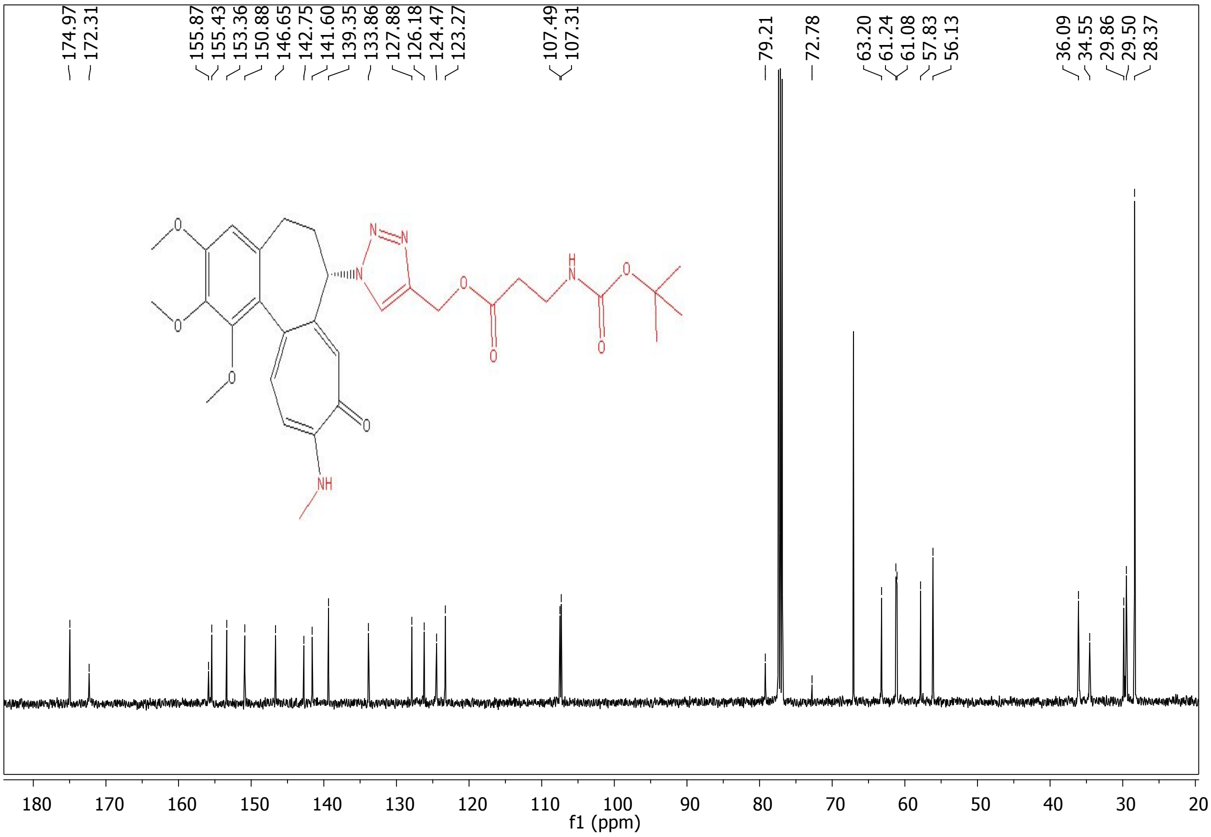

Figure S74. The ^{13}C NMR spectrum of **26** in $CDCl_3$.

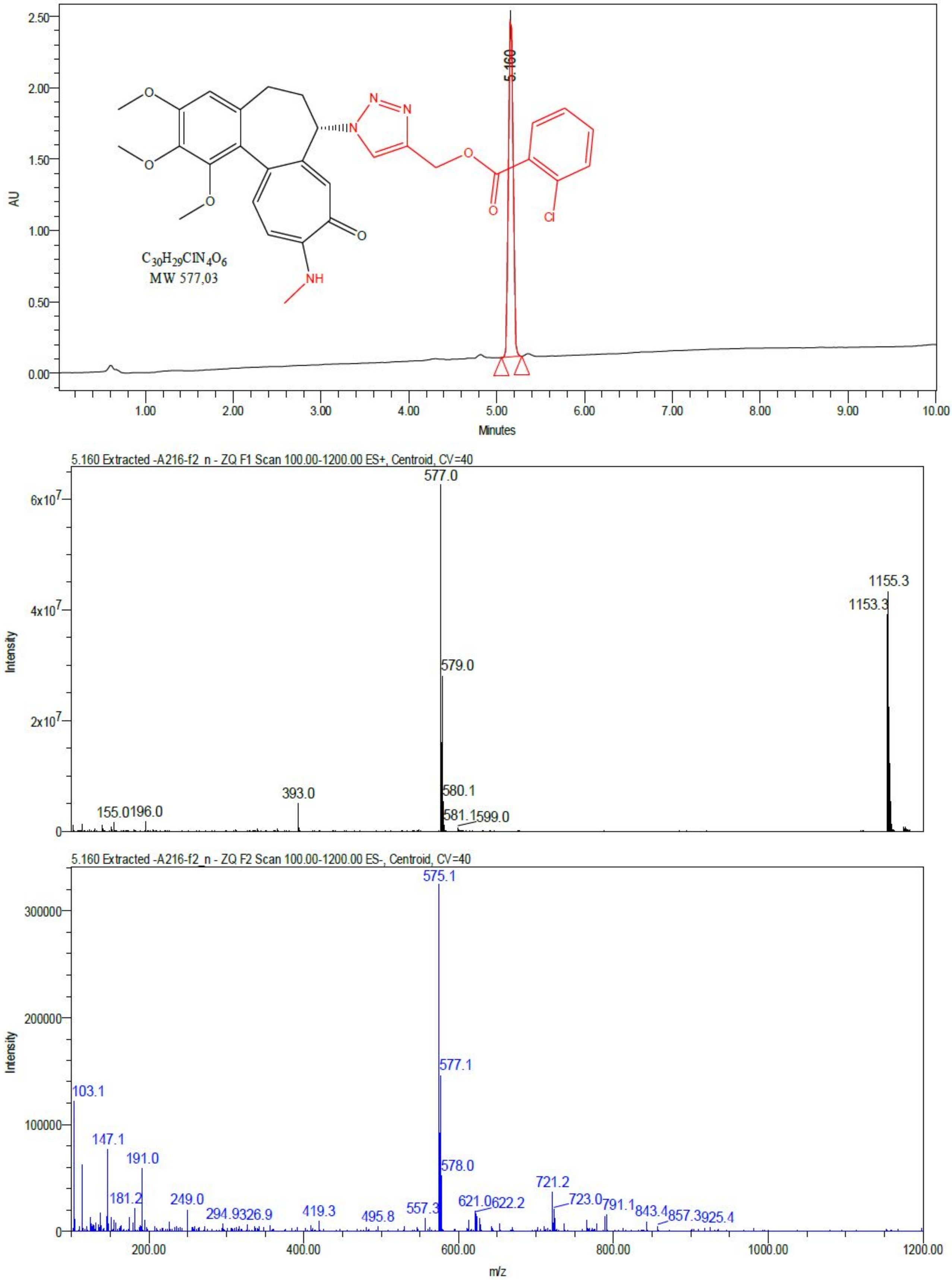

Figure S75. The LC-MS chromatogram and mass spectra of **27**.

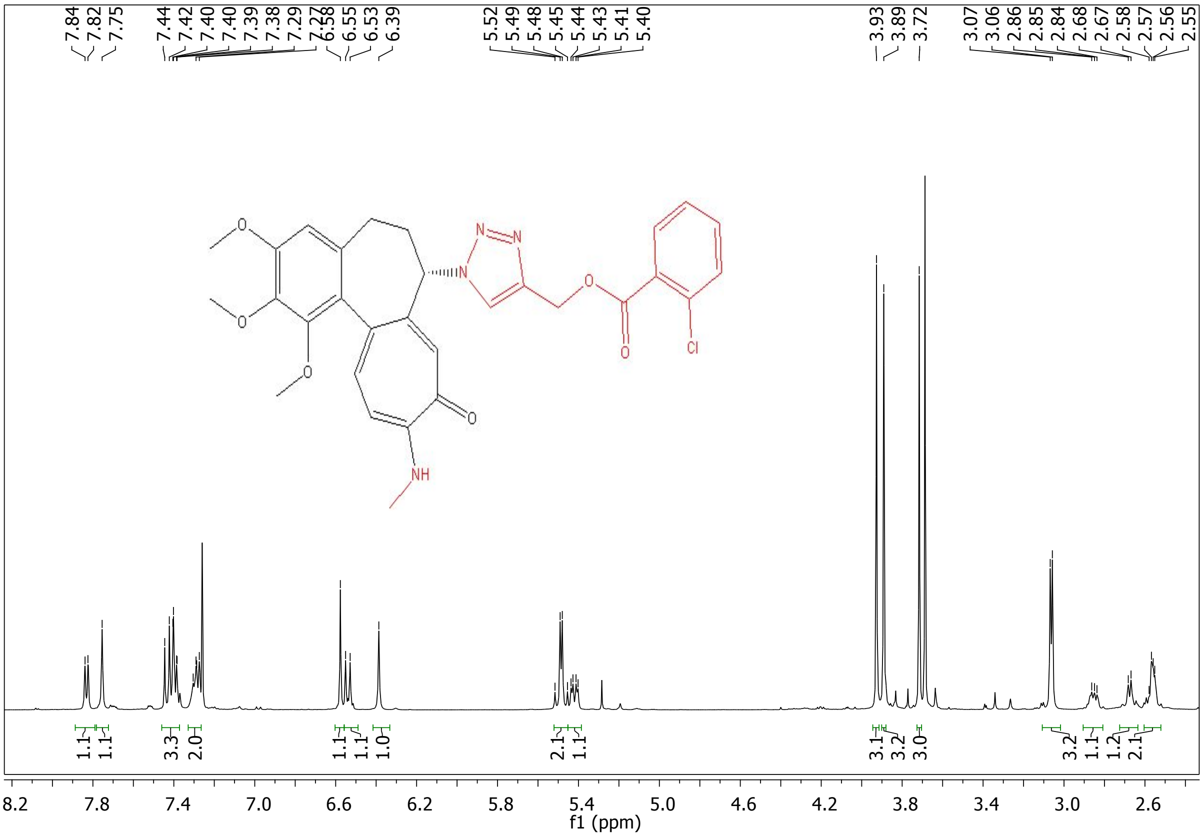

Figure S76. The ^{1}H NMR spectrum of **27** in $CDCl_3$.

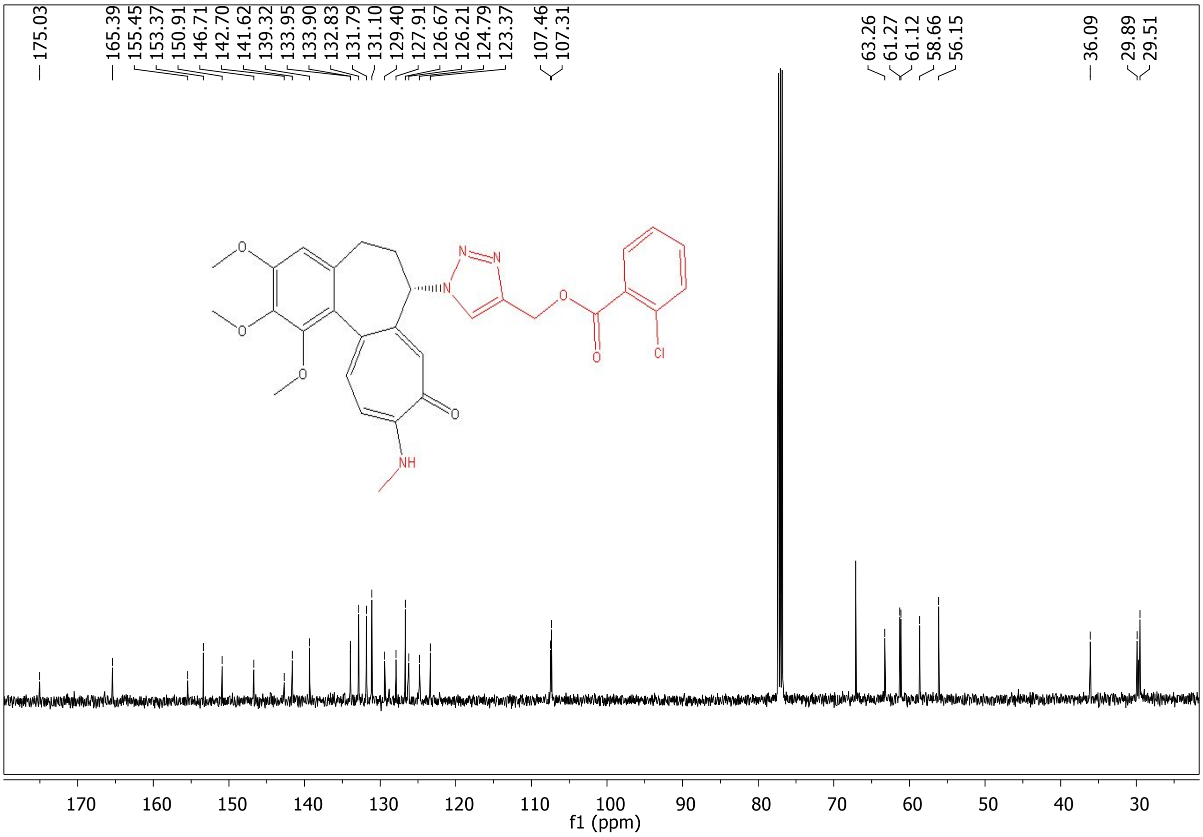

Figure S77. The ^{13}C NMR spectrum of **27** in $CDCl_3$.

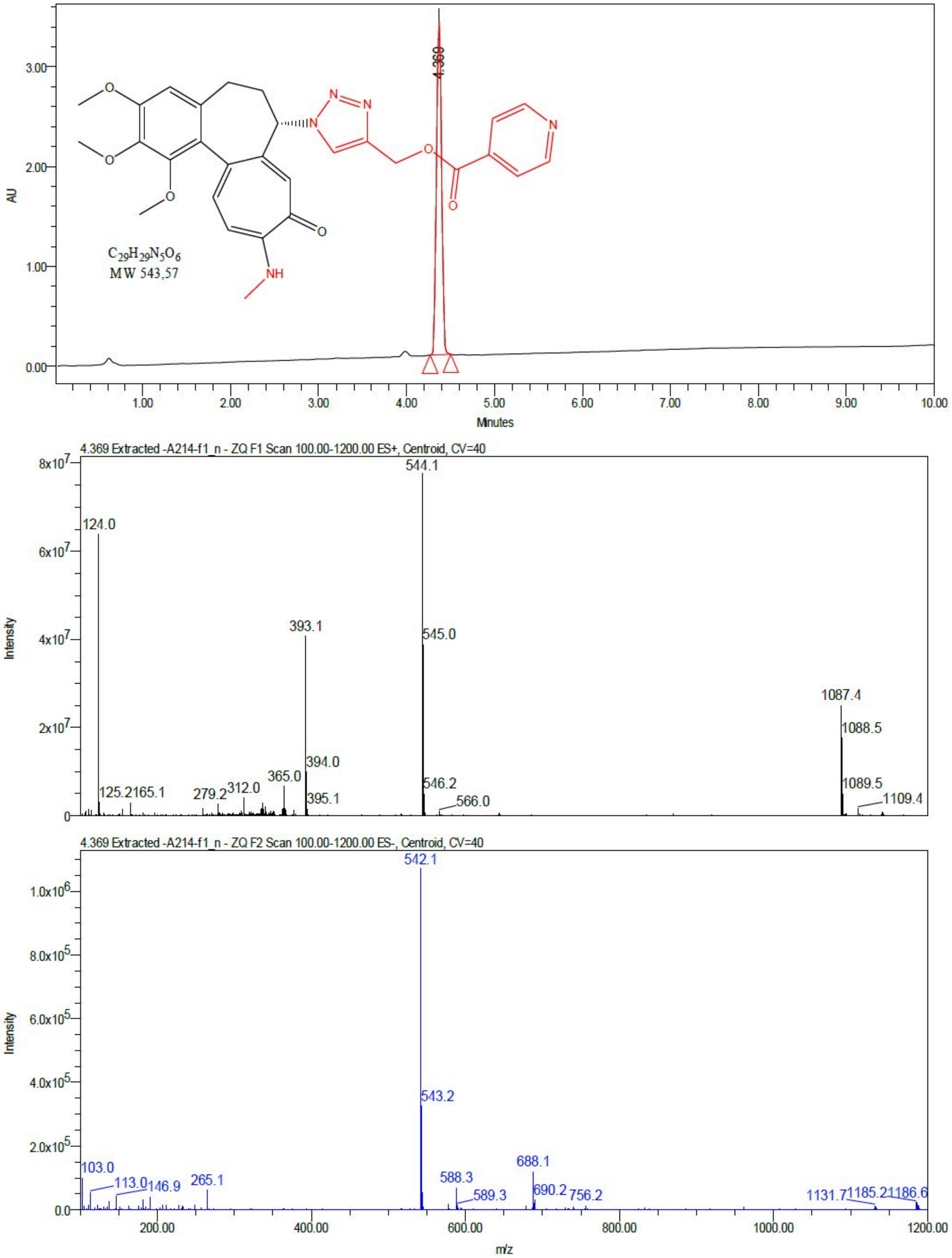

Figure S78. The LC-MS chromatogram and mass spectra of **28**.

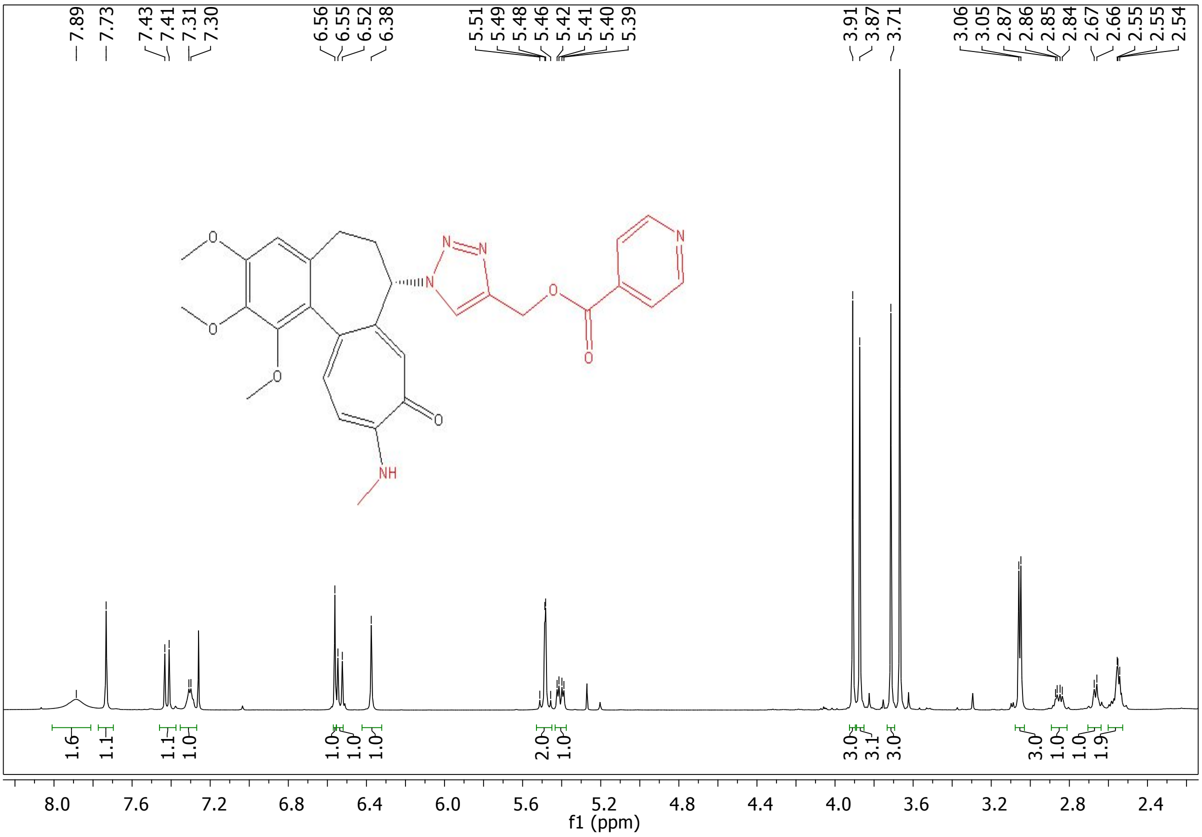

Figure S79. The ^{1}H NMR spectrum of **28** in $CDCl_3$.

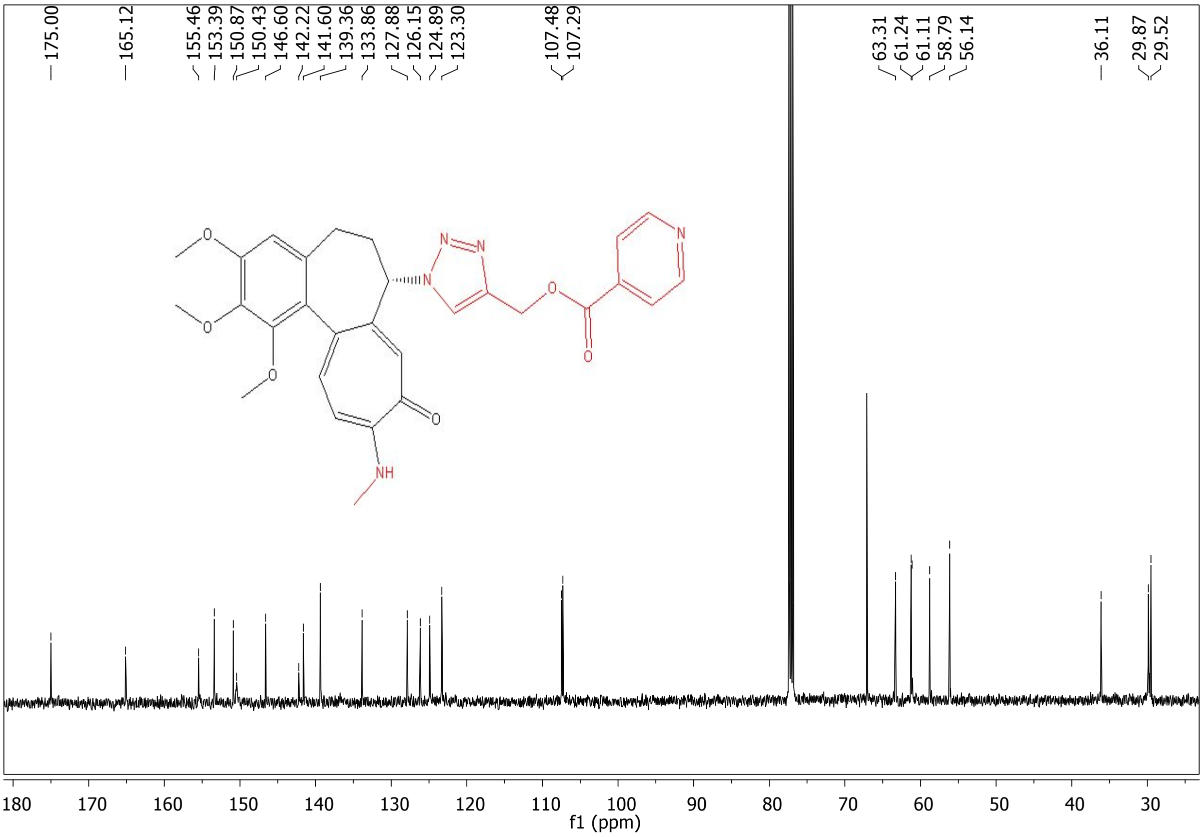

Figure S80. The ^{13}C NMR spectrum of **28** in $CDCl_3$.

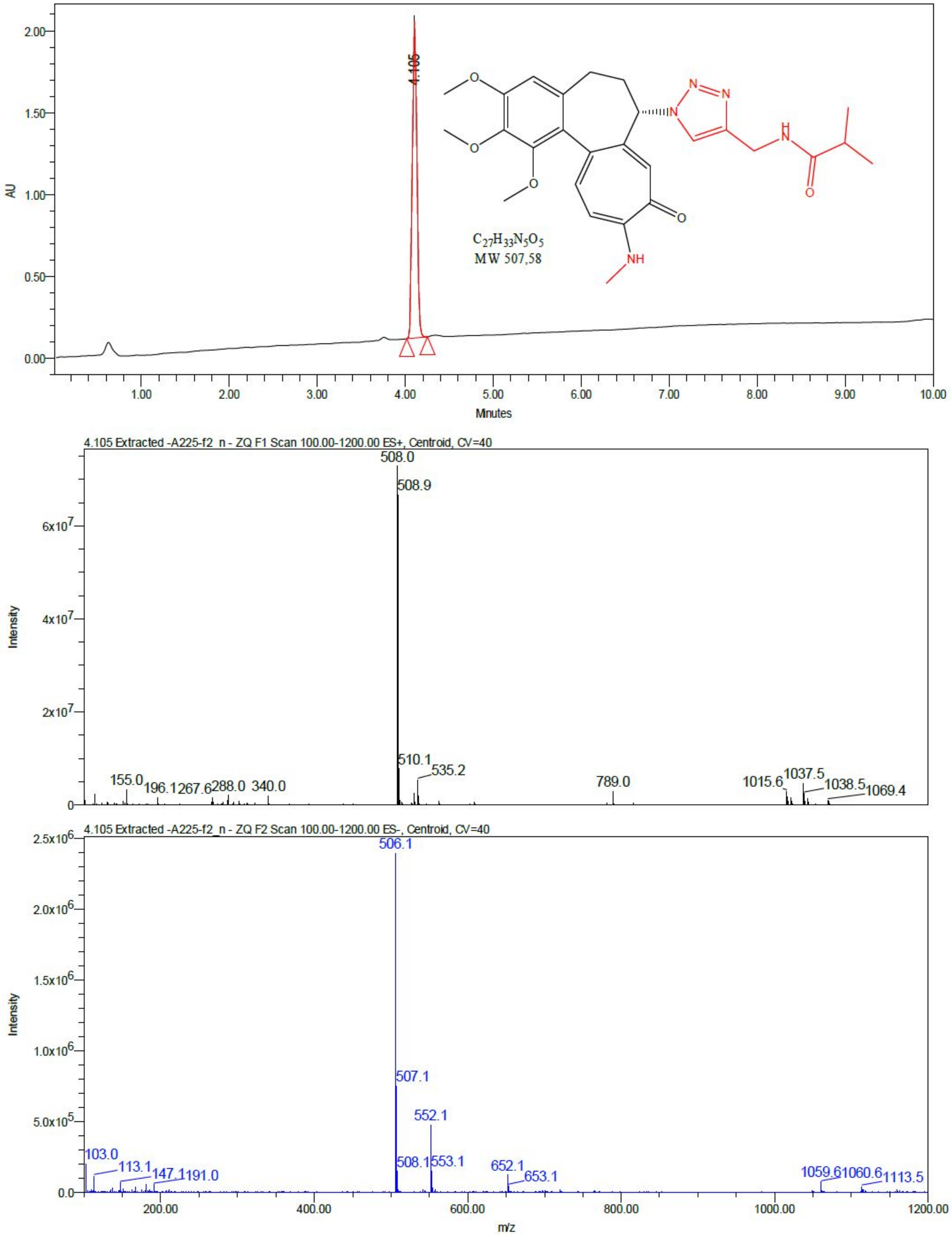

Figure S81. The LC-MS chromatogram and mass spectra of **29**.

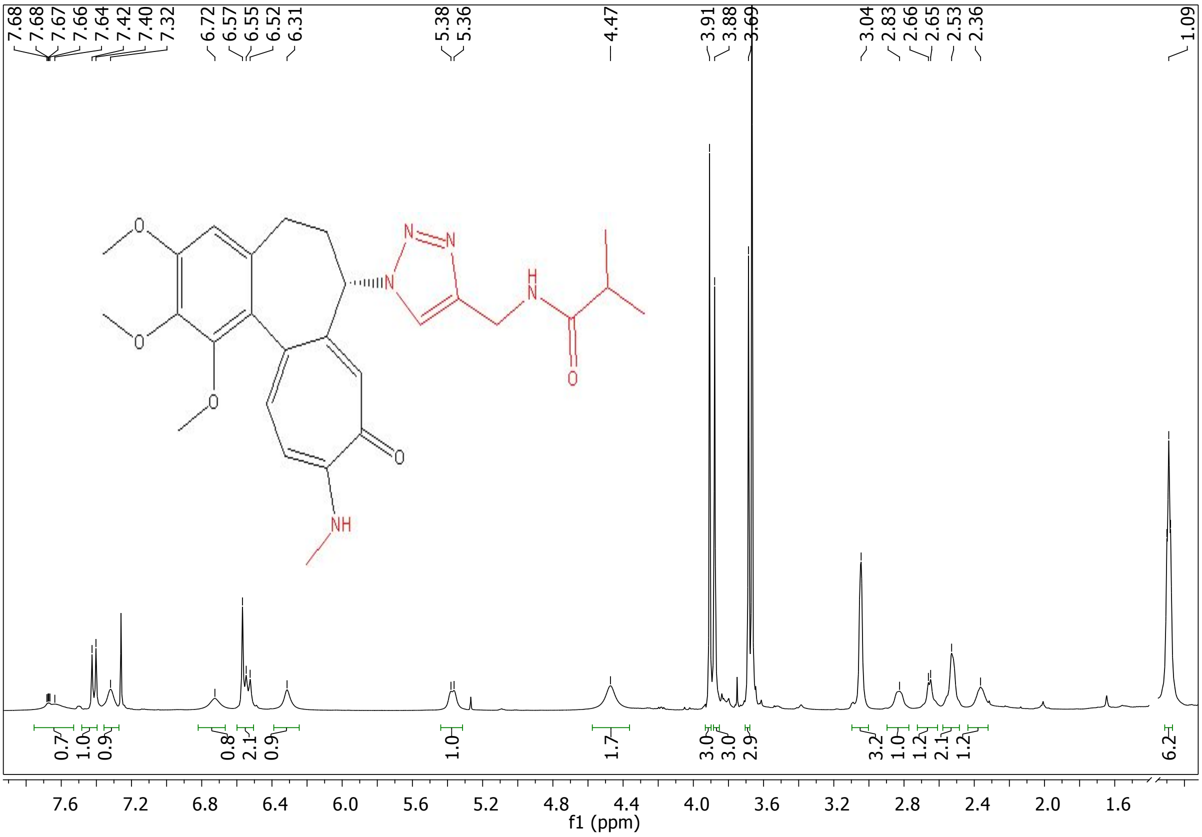

Figure S82. The ^{1}H NMR spectrum of **29** in $CDCl_3$.

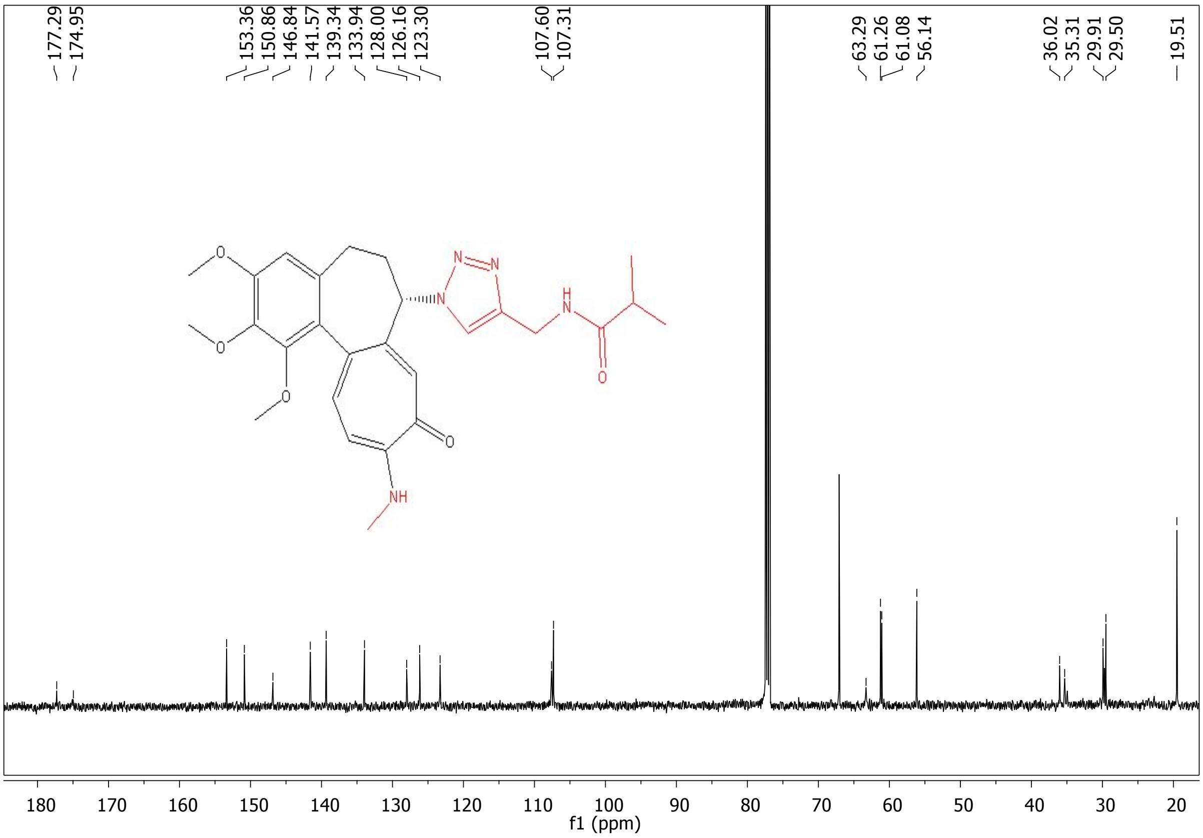

Figure S83. The ^{13}C NMR spectrum of **29** in $CDCl_3$.

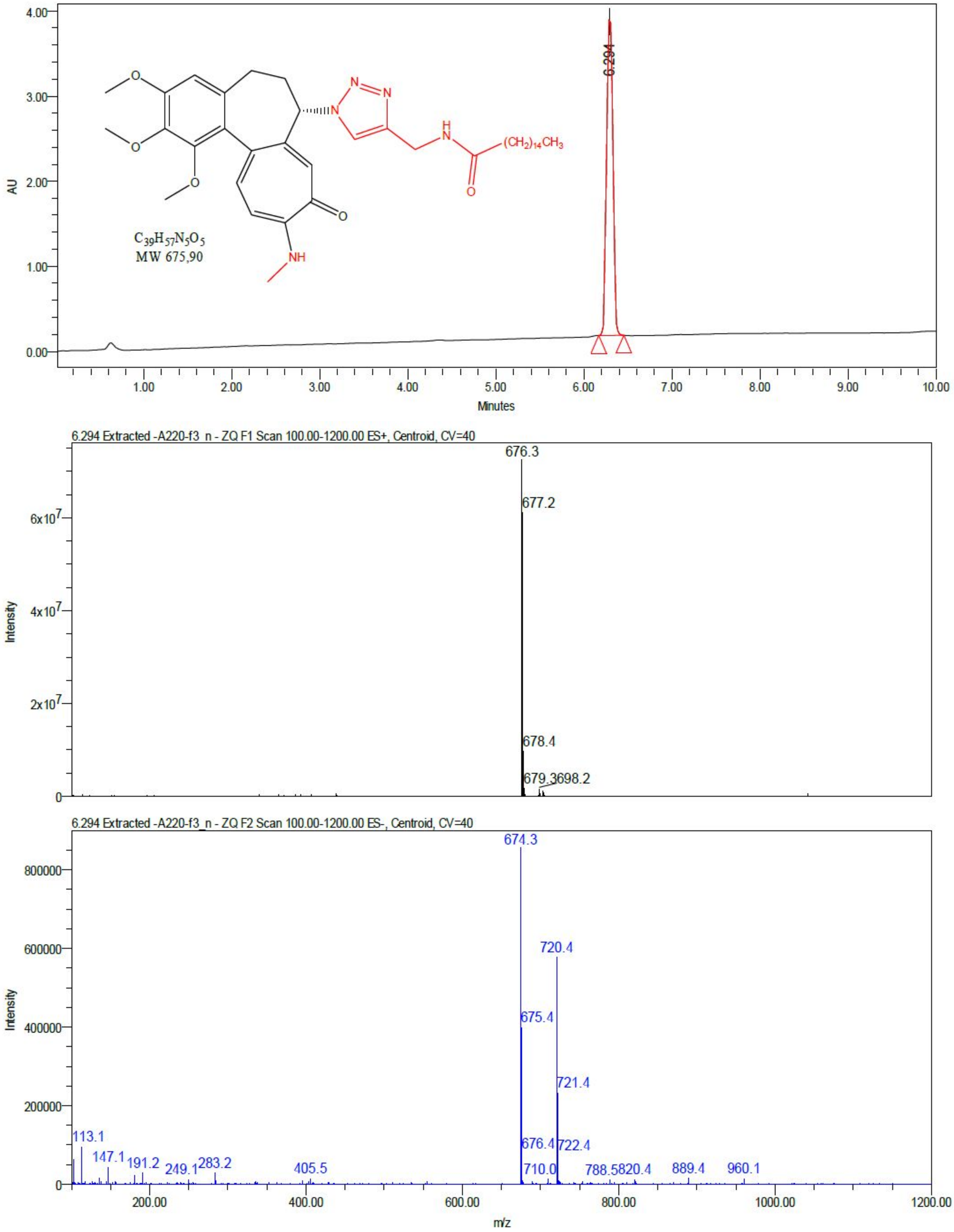

Figure S84. The LC-MS chromatogram and mass spectra of **30**.

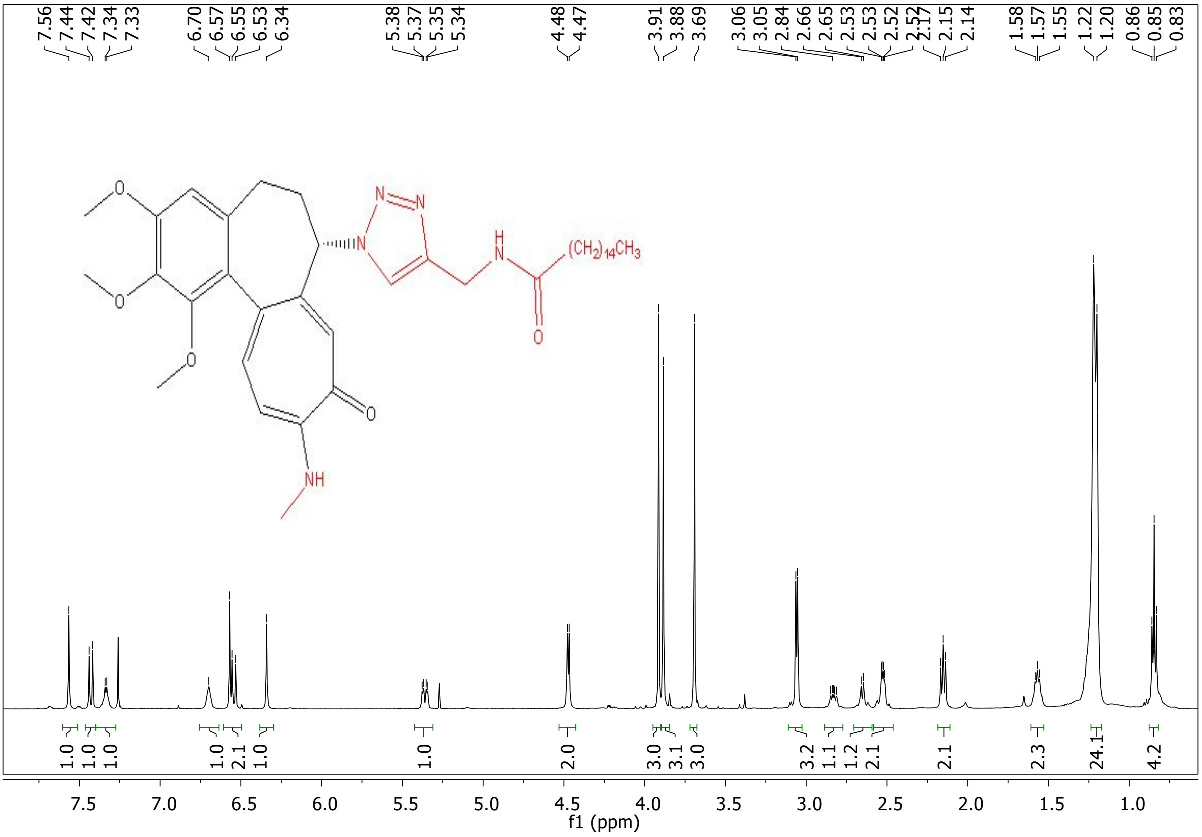

Figure S85. The ^{1}H NMR spectrum of **30** in $CDCl_3$

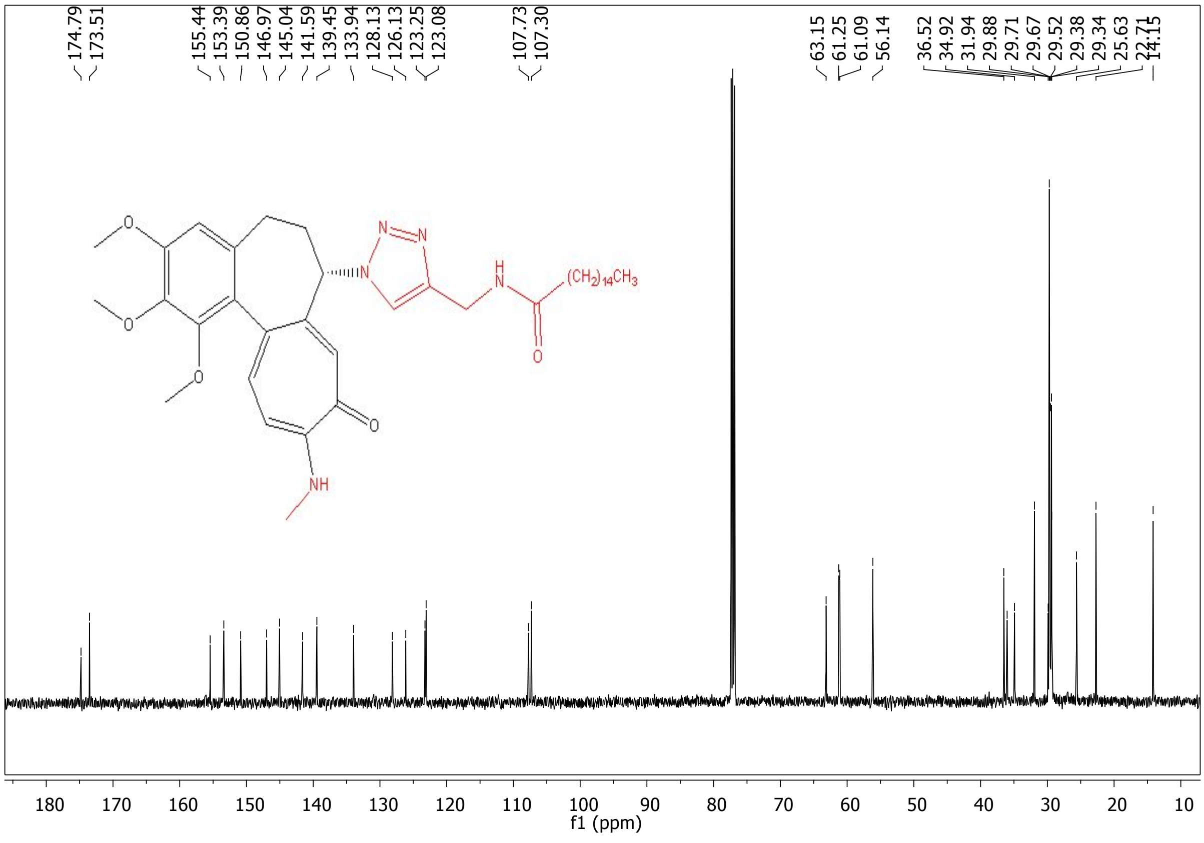

Figure S86. The ^{13}C NMR spectrum of **30** in $CDCl_3$..

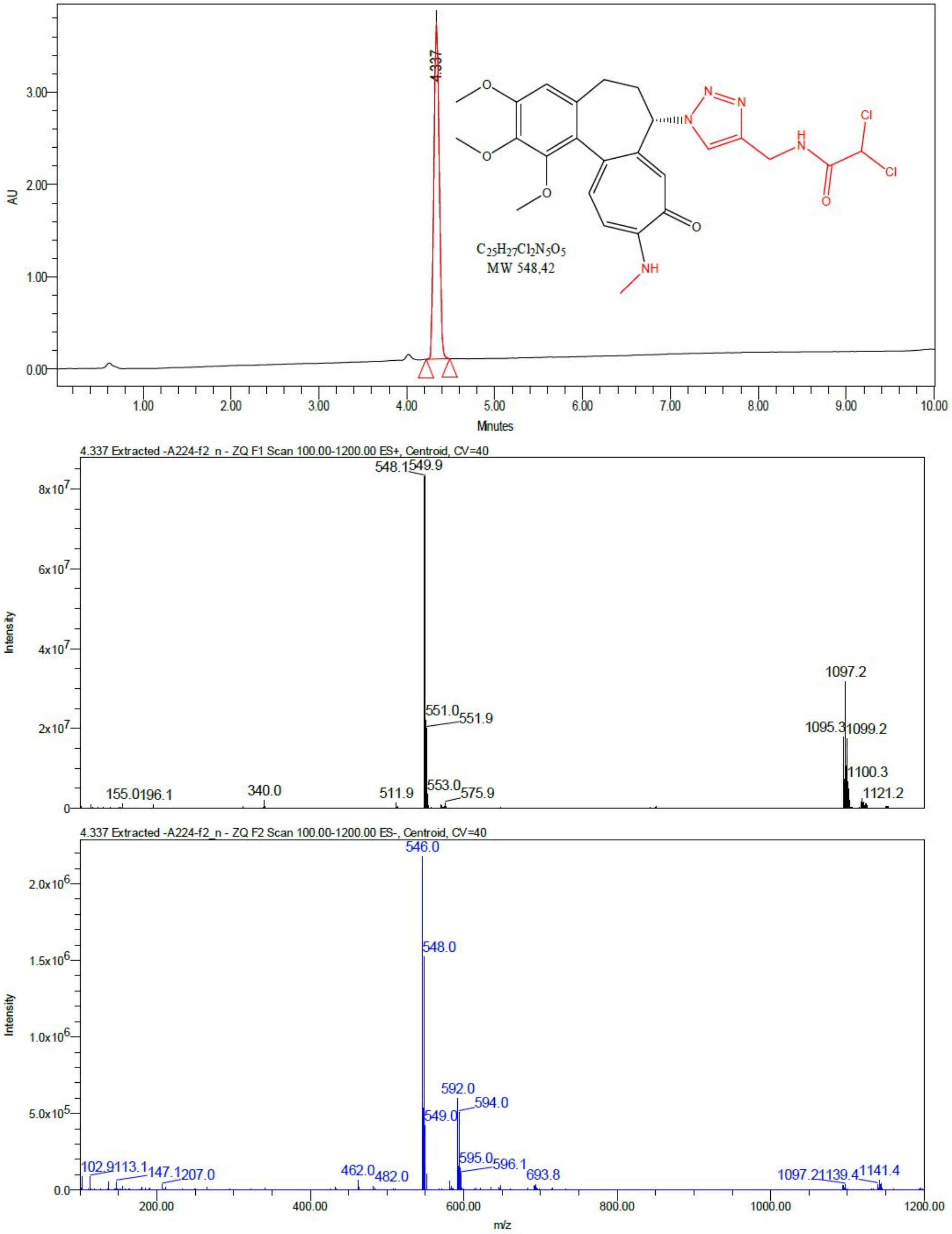

Figure S87. The LC-MS chromatogram and mass spectra of **31**.

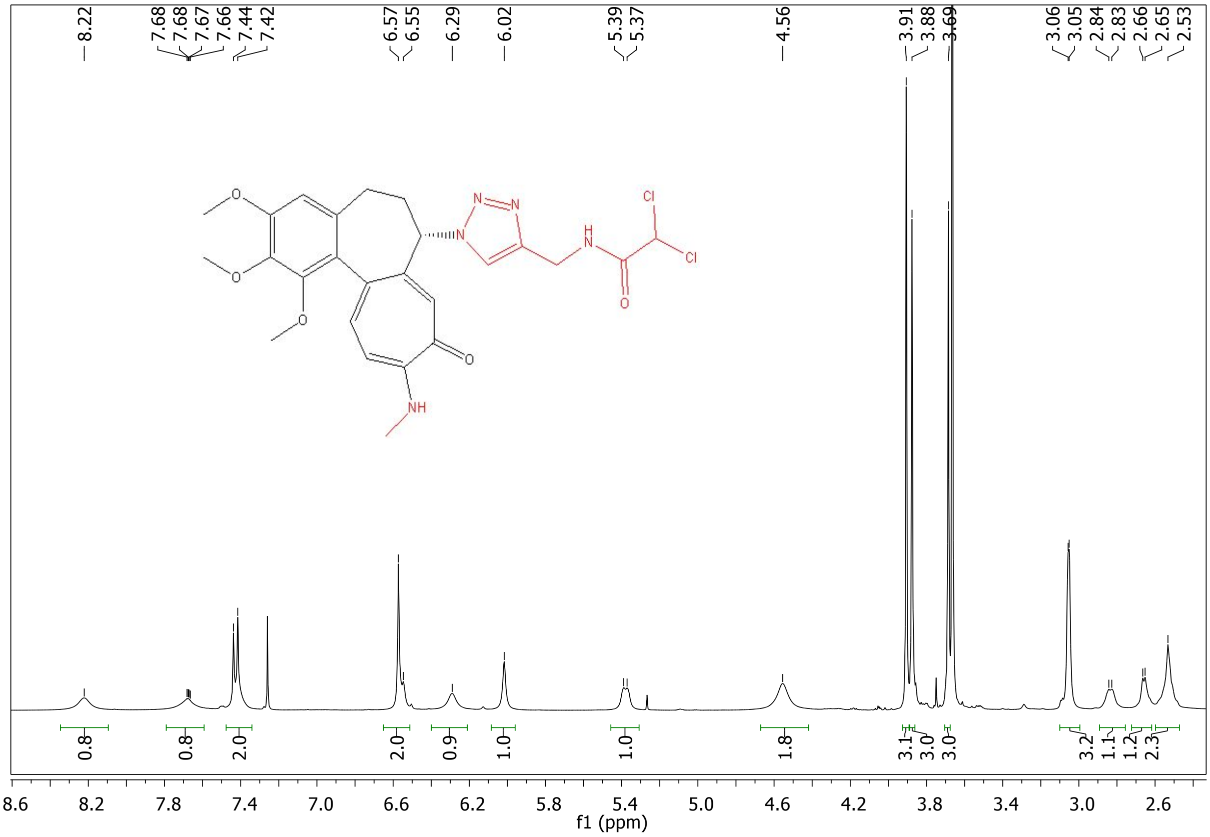

Figure S88. The ^{1}H NMR spectrum of **31** in $CDCl_3$.

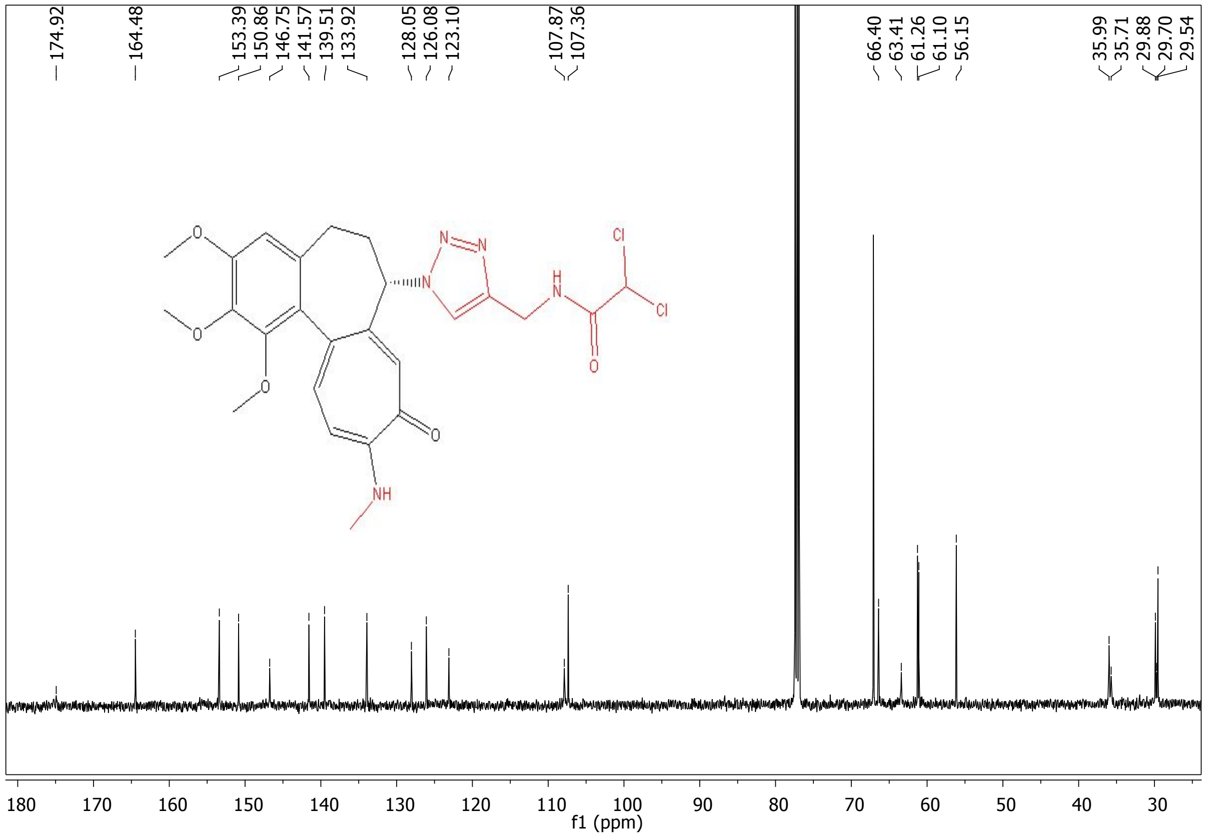

Figure S89. The ^{13}C NMR spectrum of **31** in $CDCl_3$.

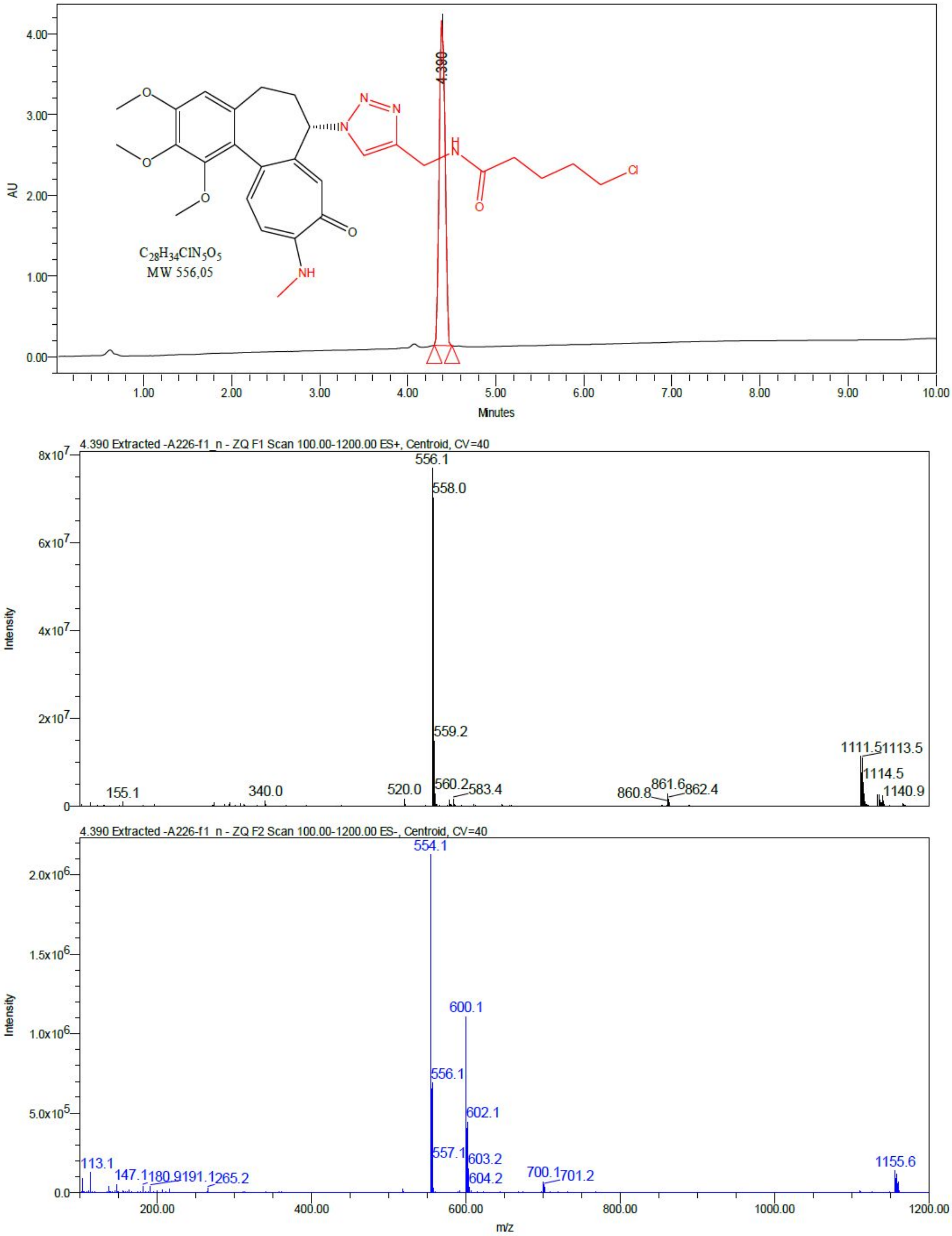

Figure S90. The LC-MS chromatogram and mass spectra of **32**.

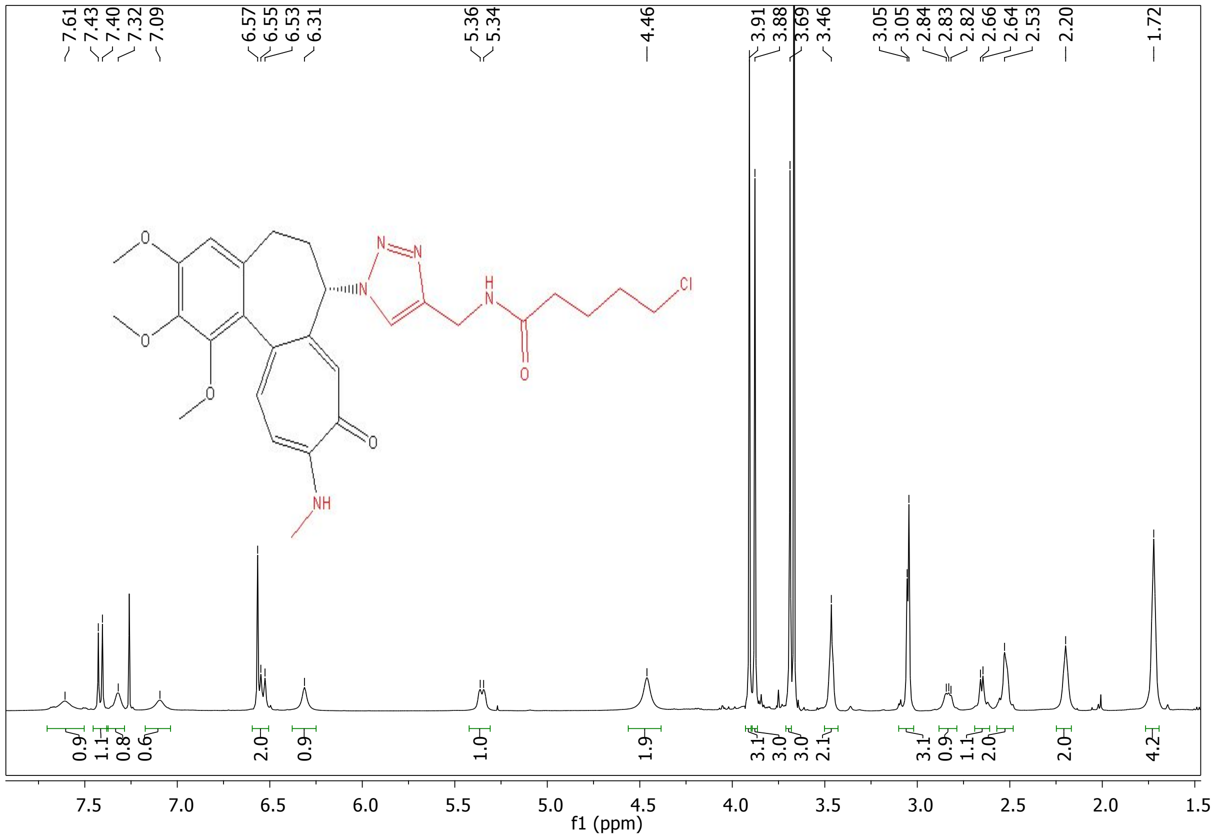

Figure S91. The ^{1}H NMR spectrum of **32** in $CDCl_3$.

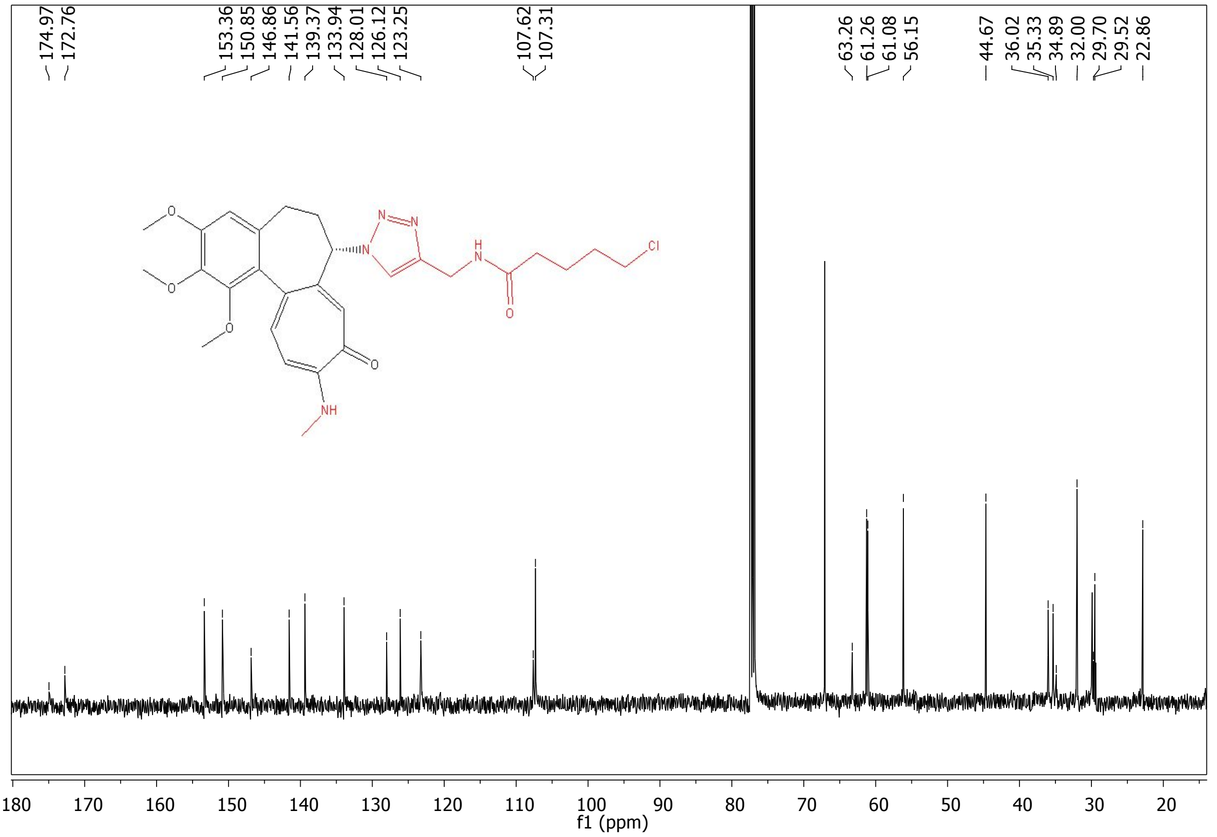

Figure S92. The ^{13}C NMR spectrum of **32** in $CDCl_3$.

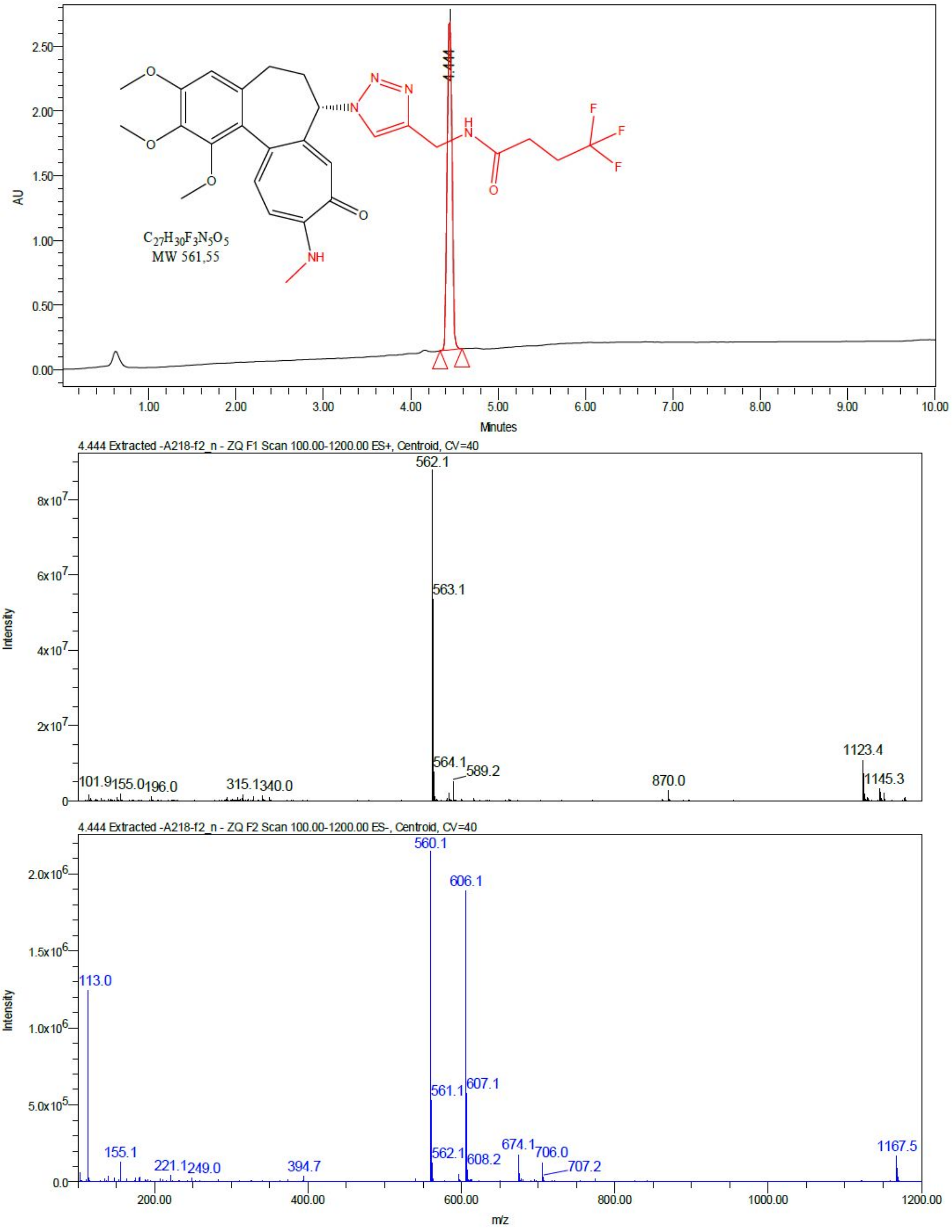

Figure S93. The LC-MS chromatogram and mass spectra of **33**.

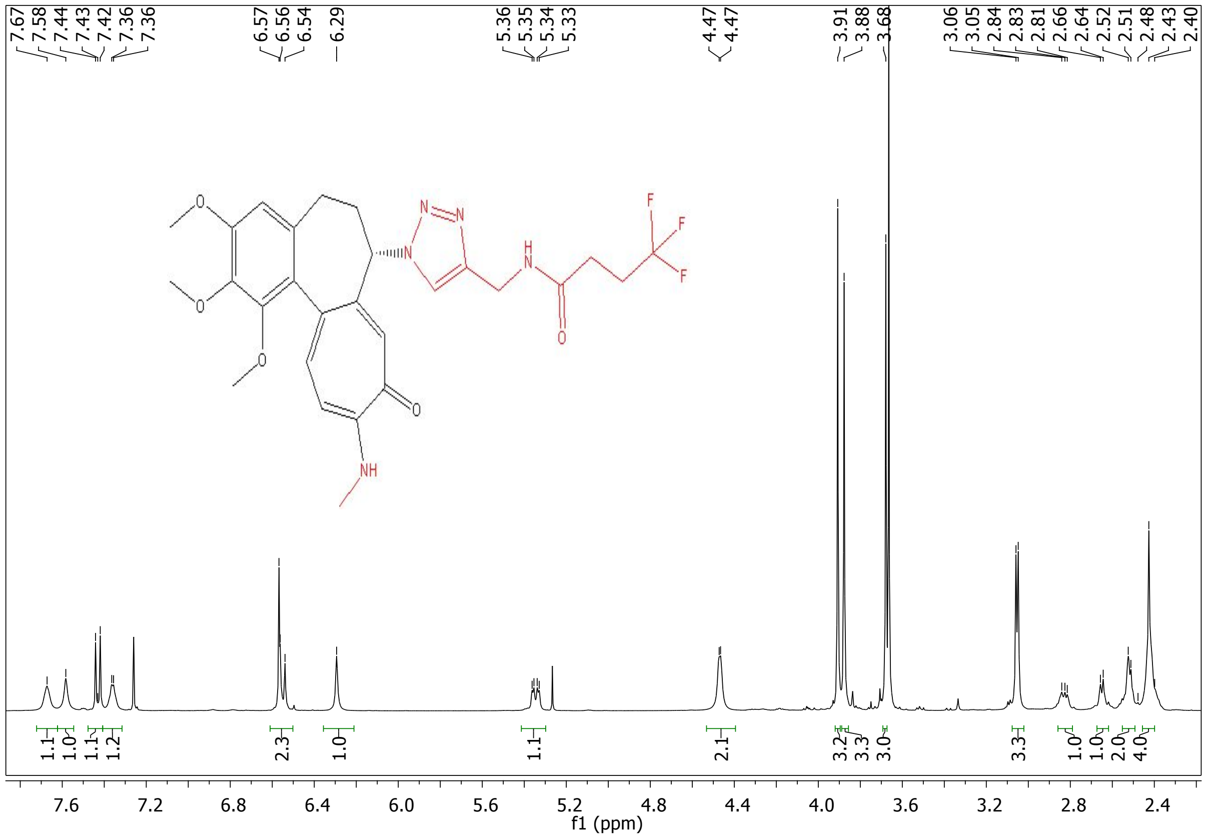

Figure S94. The ^{1}H NMR spectrum of **33** in $CDCl_3$.

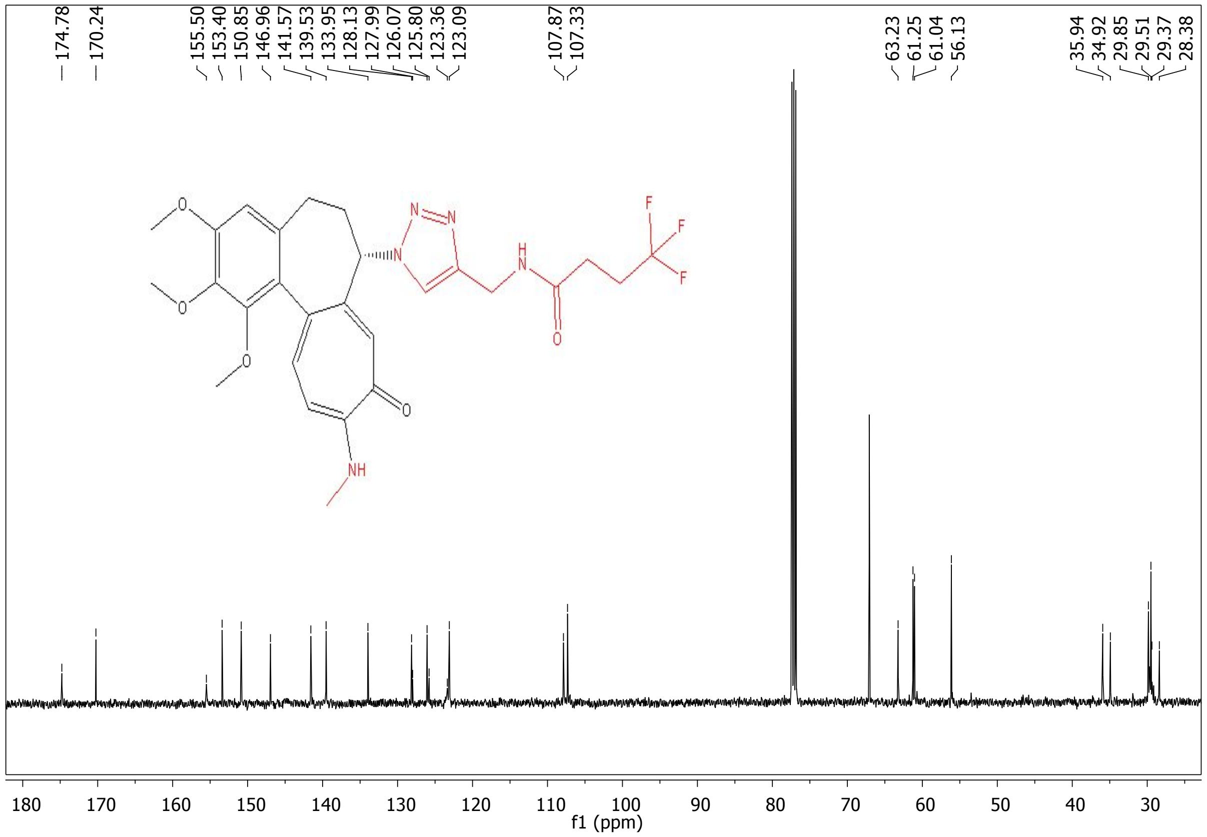

Figure S95. The ^{13}C NMR spectrum of **33** in $CDCl_3$.

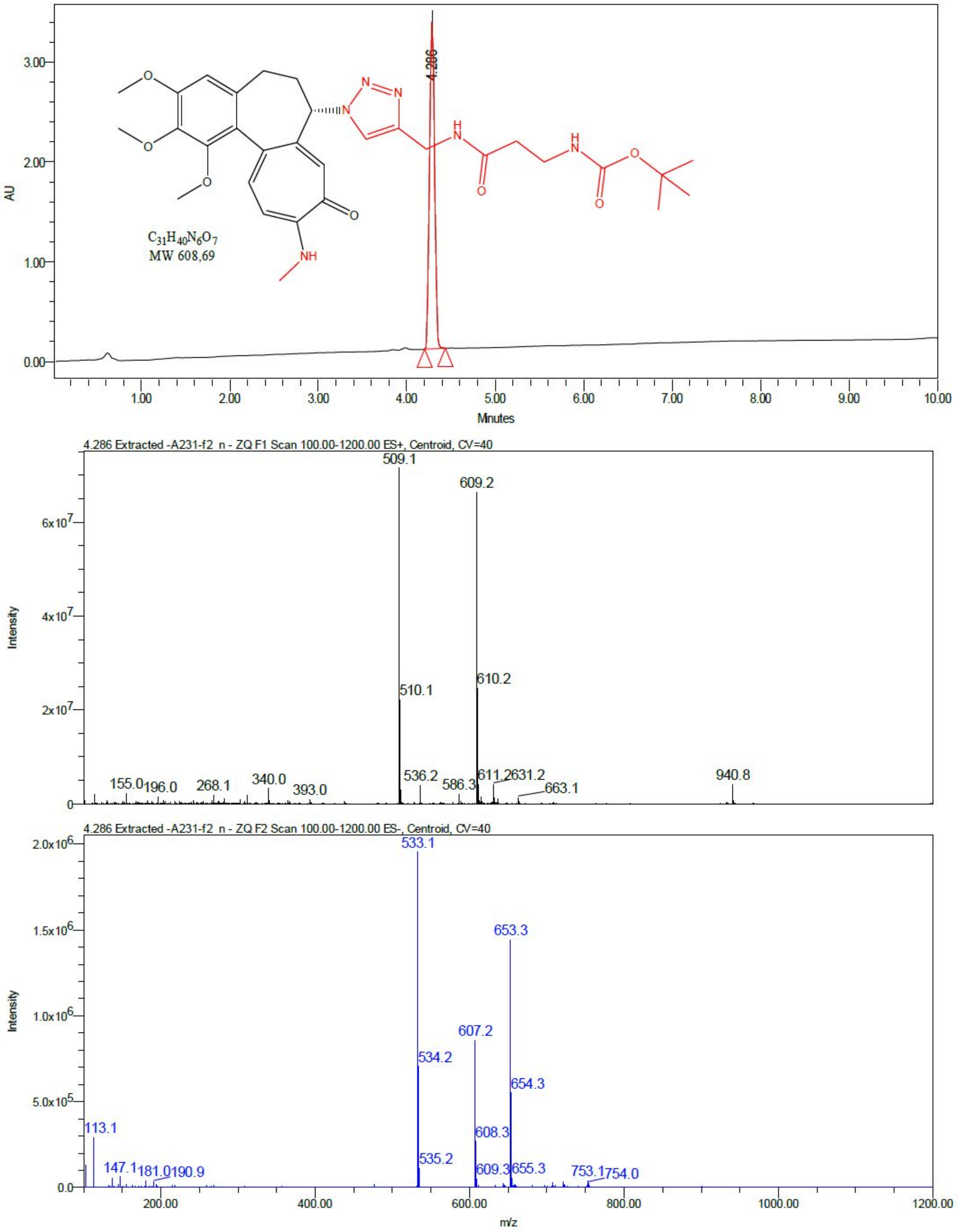

Figure S96. The LC-MS chromatogram and mass spectra of **34**.

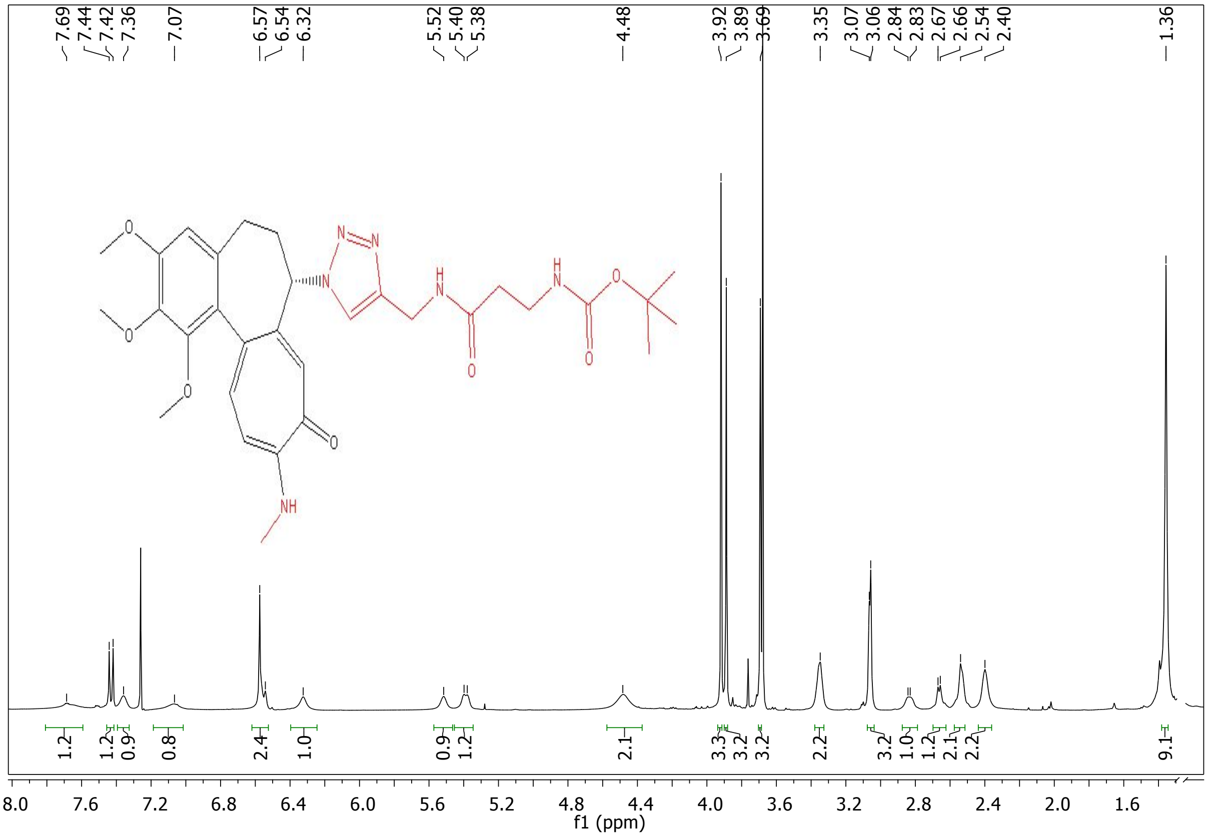

Figure S97. The ^{1}H NMR spectrum of **34** in $CDCl_3$.

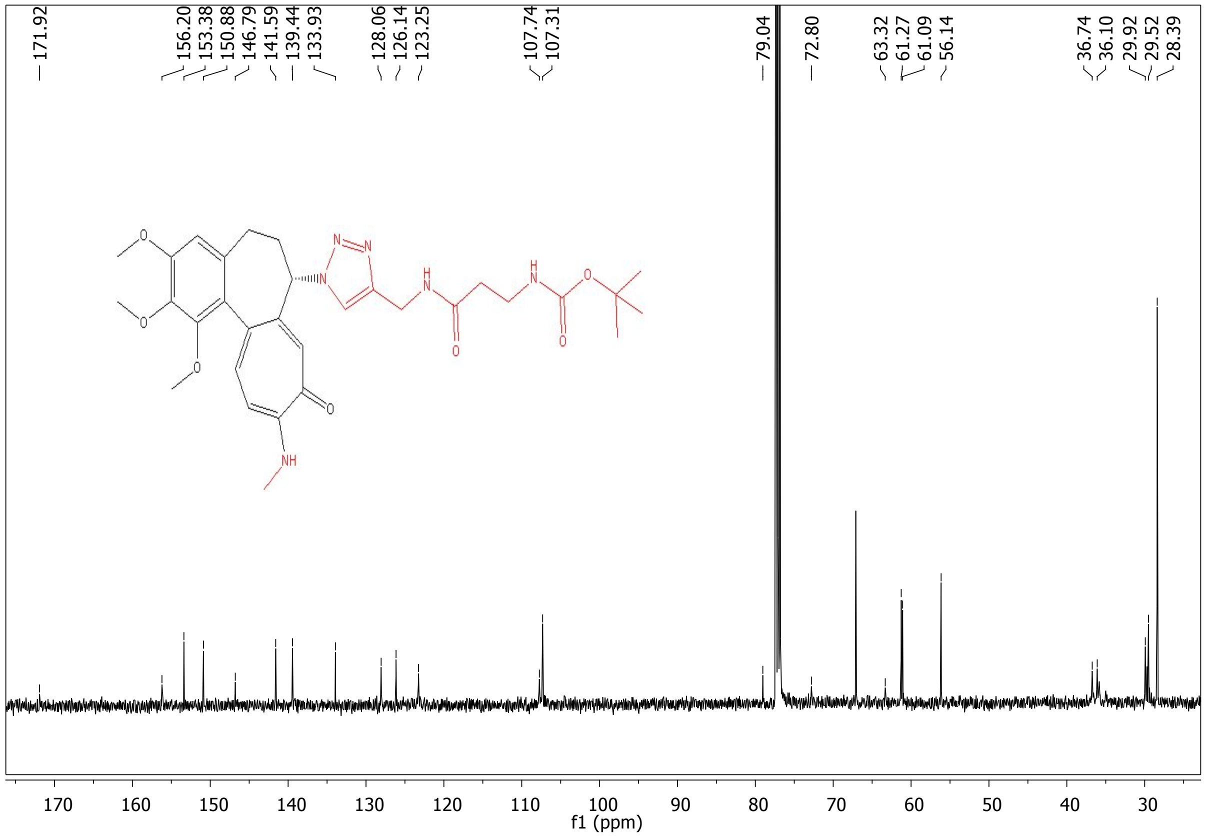

Figure S98. The ^{13}C NMR spectrum of **34** in $CDCl_3$.

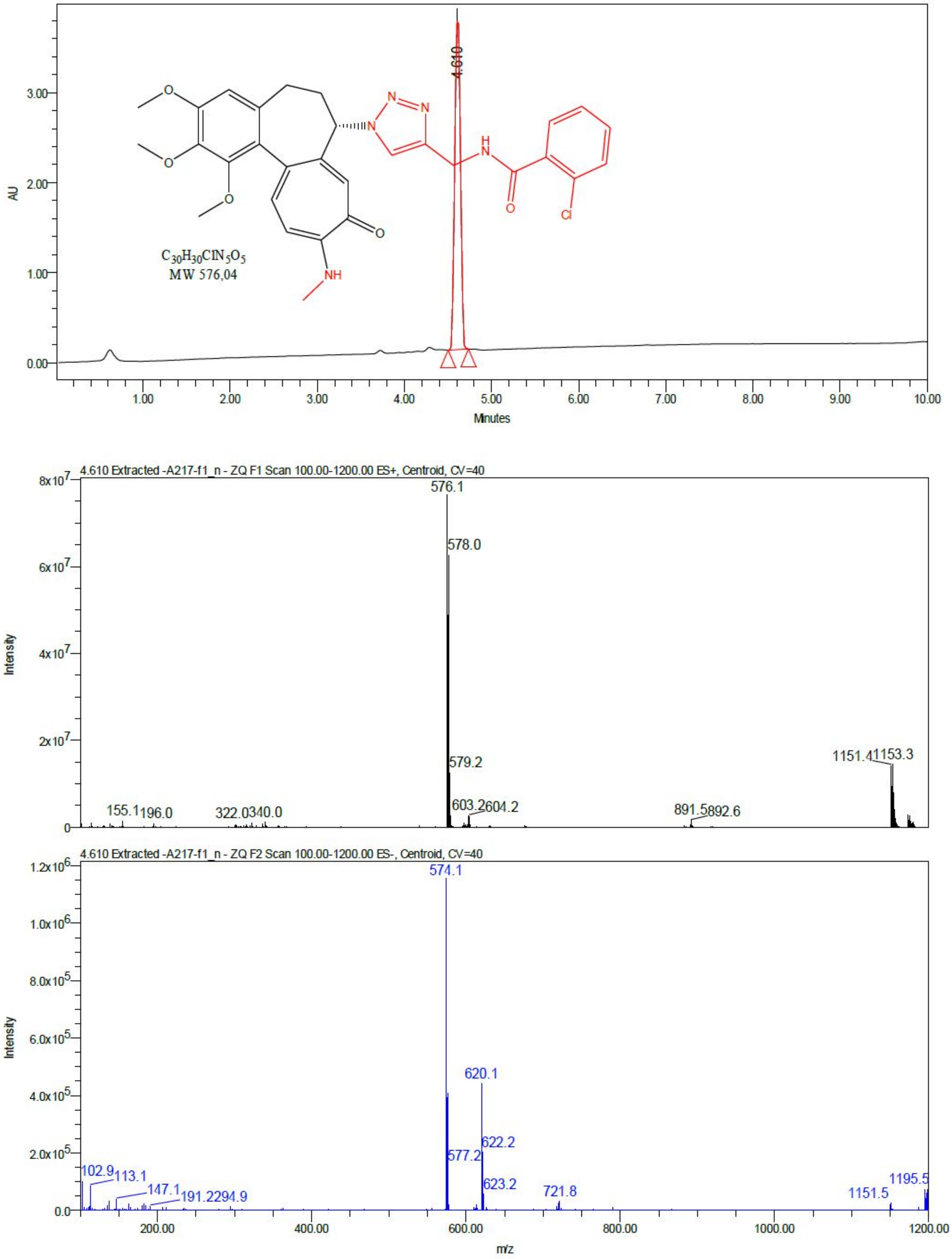

Figure S99. The LC-MS chromatogram and mass spectra of **35**.

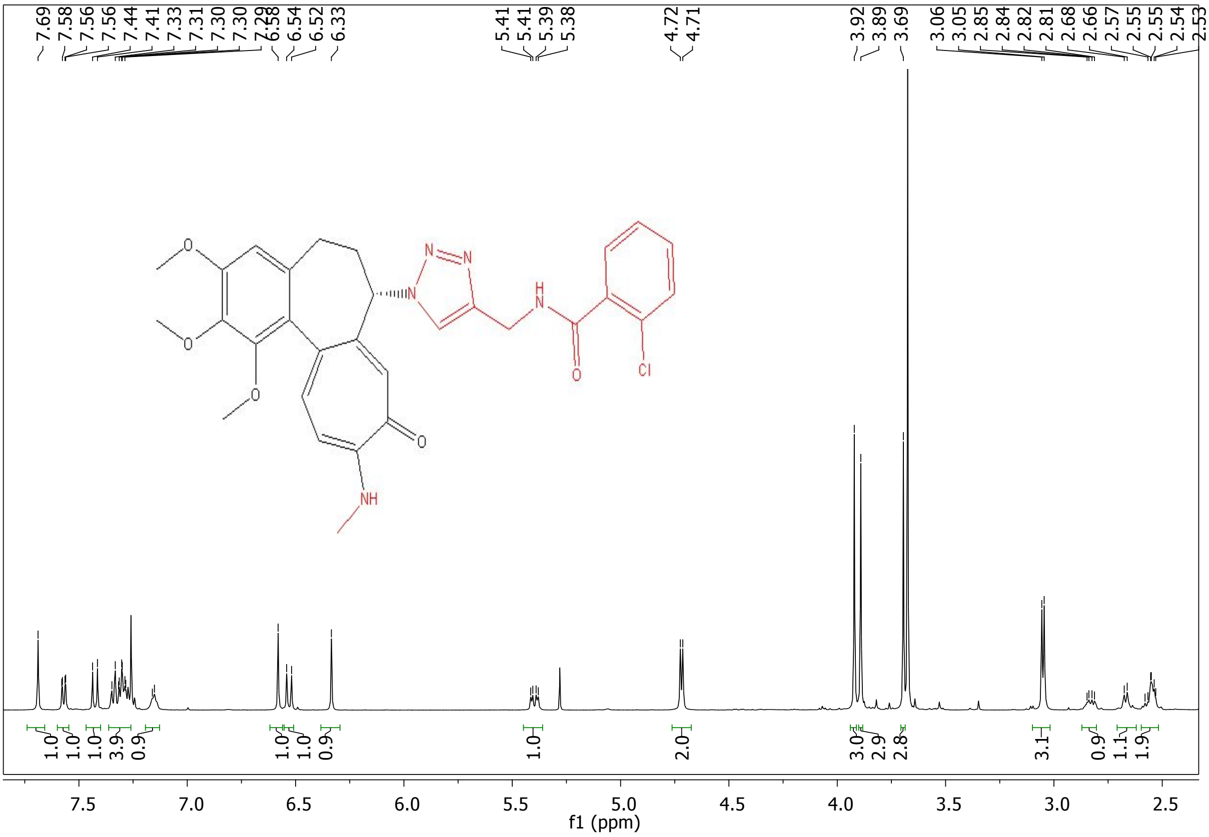

Figure S100. The ^{1}H NMR spectrum of **35** in $CDCl_3$.

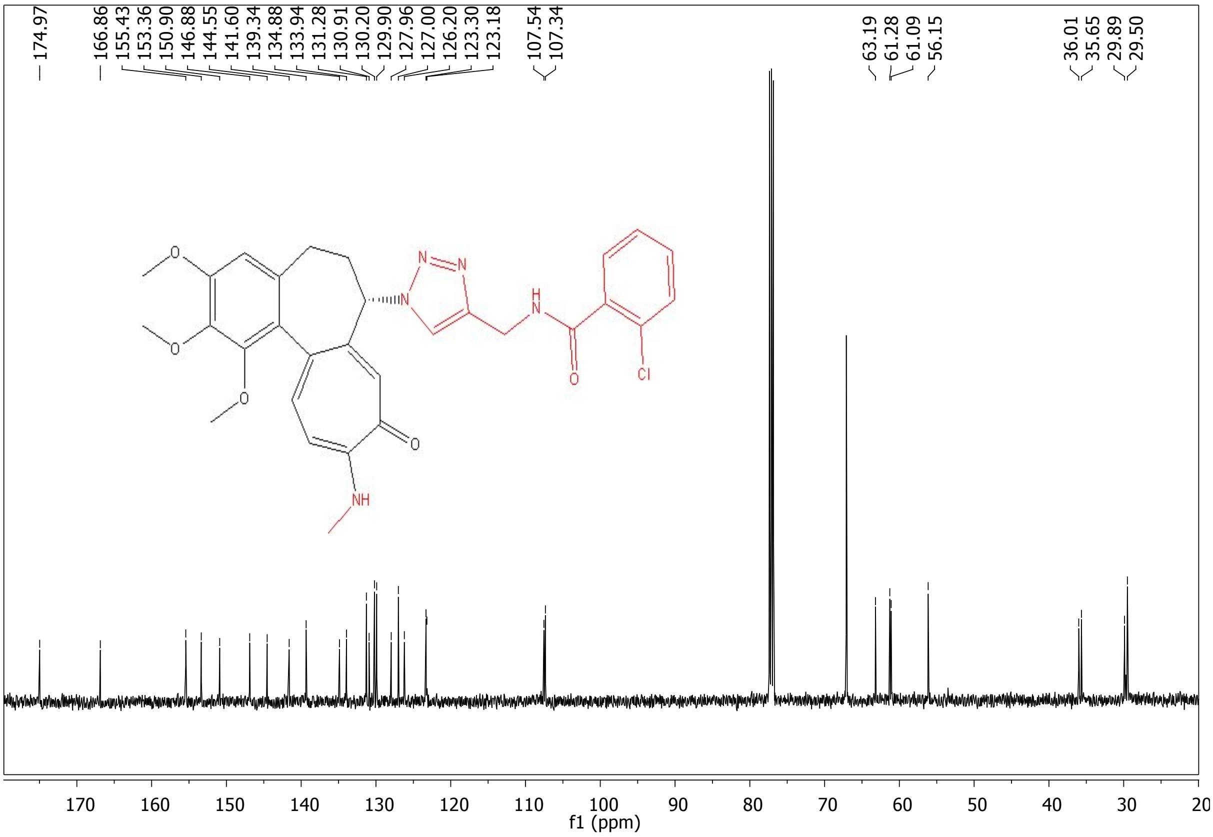

Figure S101. The ^{13}C NMR spectrum of **35** in $CDCl_3$.

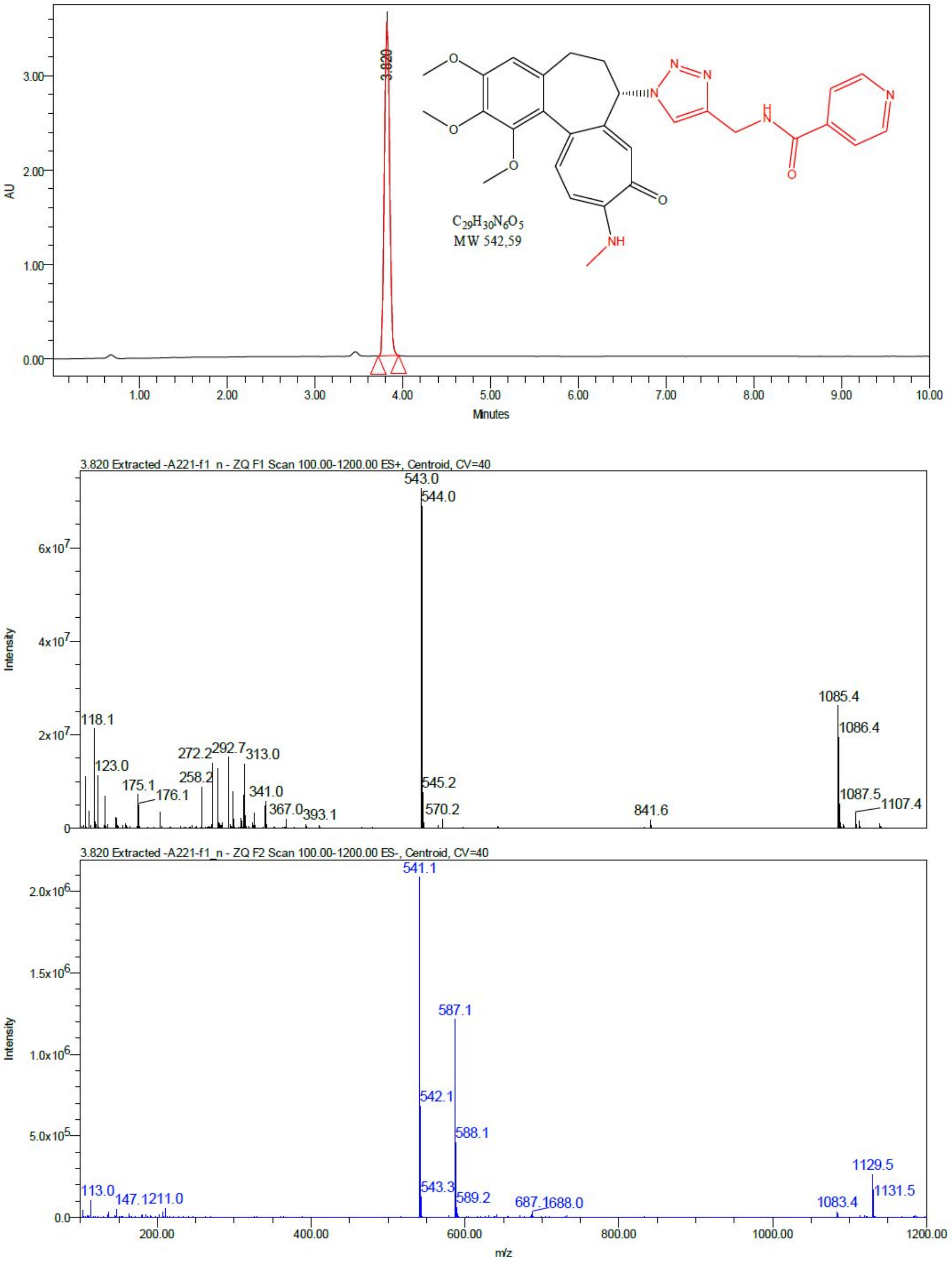

Figure S102. The LC-MS chromatogram and mass spectra of **36**.

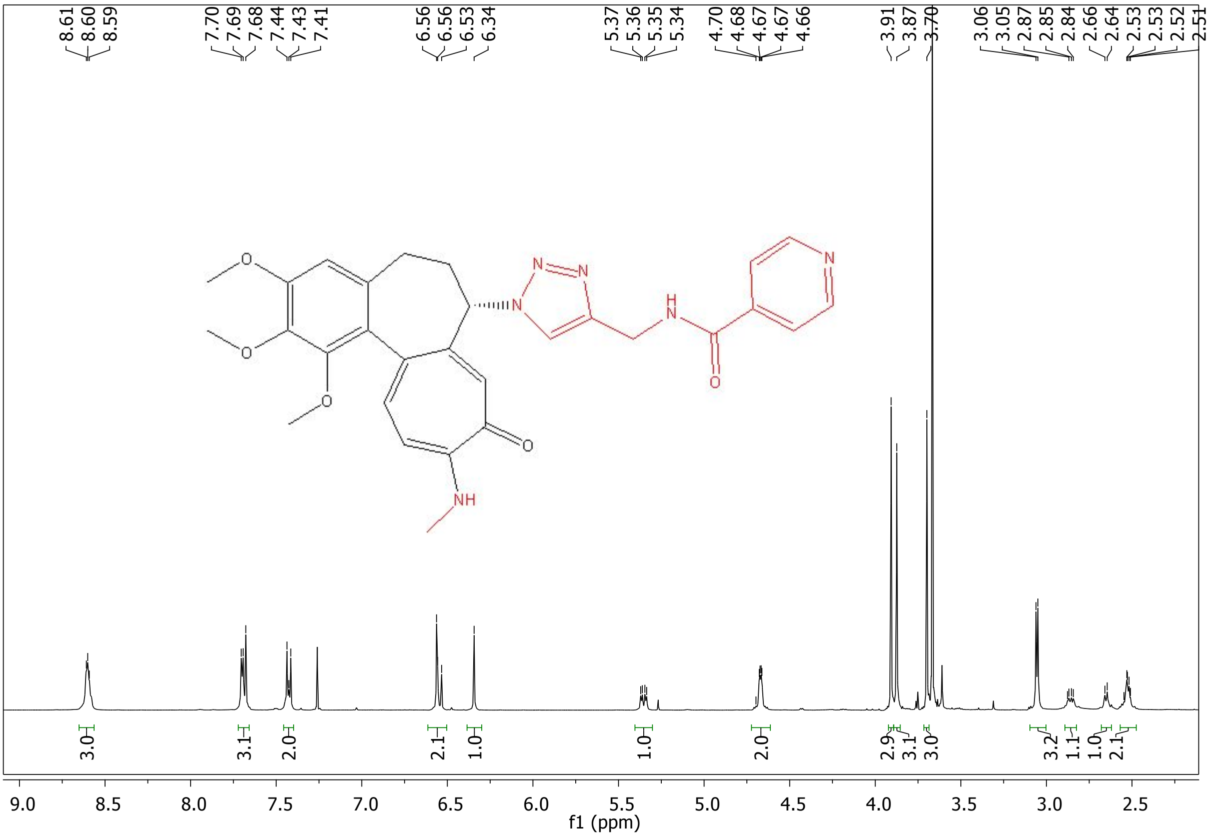

Figure S103. The ^{1}H NMR spectrum of **36** in $CDCl_3$.

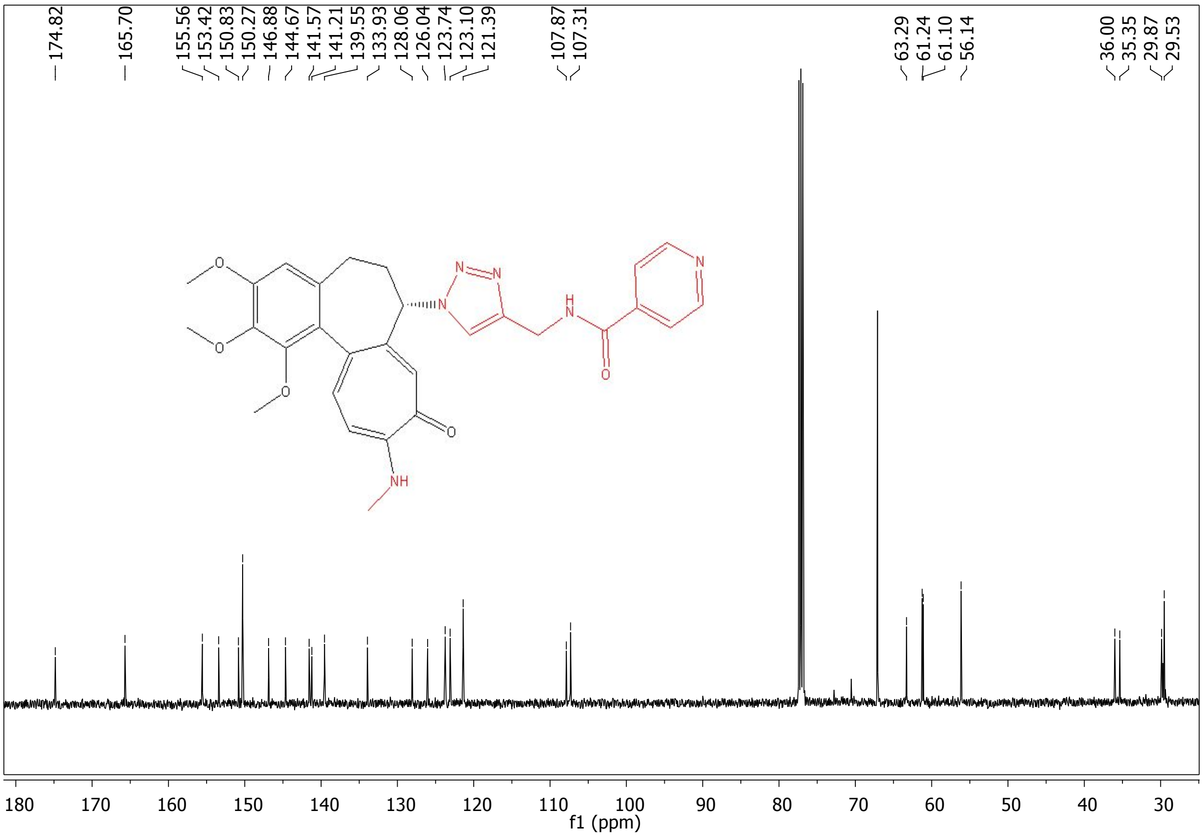

Figure S104. The ^{13}C NMR spectrum of **36** in $CDCl_3$.

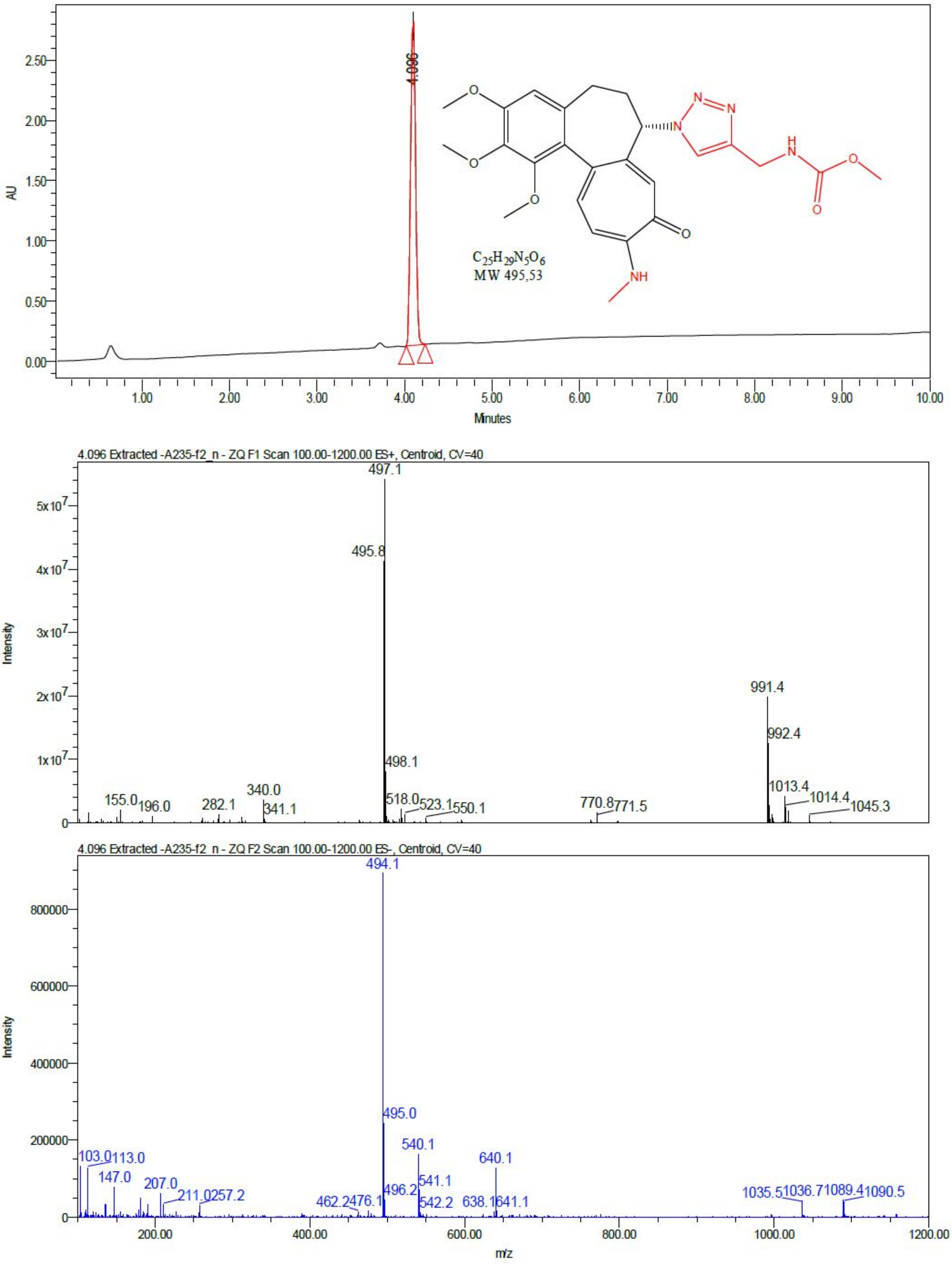

Figure S105. The LC-MS chromatogram and mass spectra of **37**.

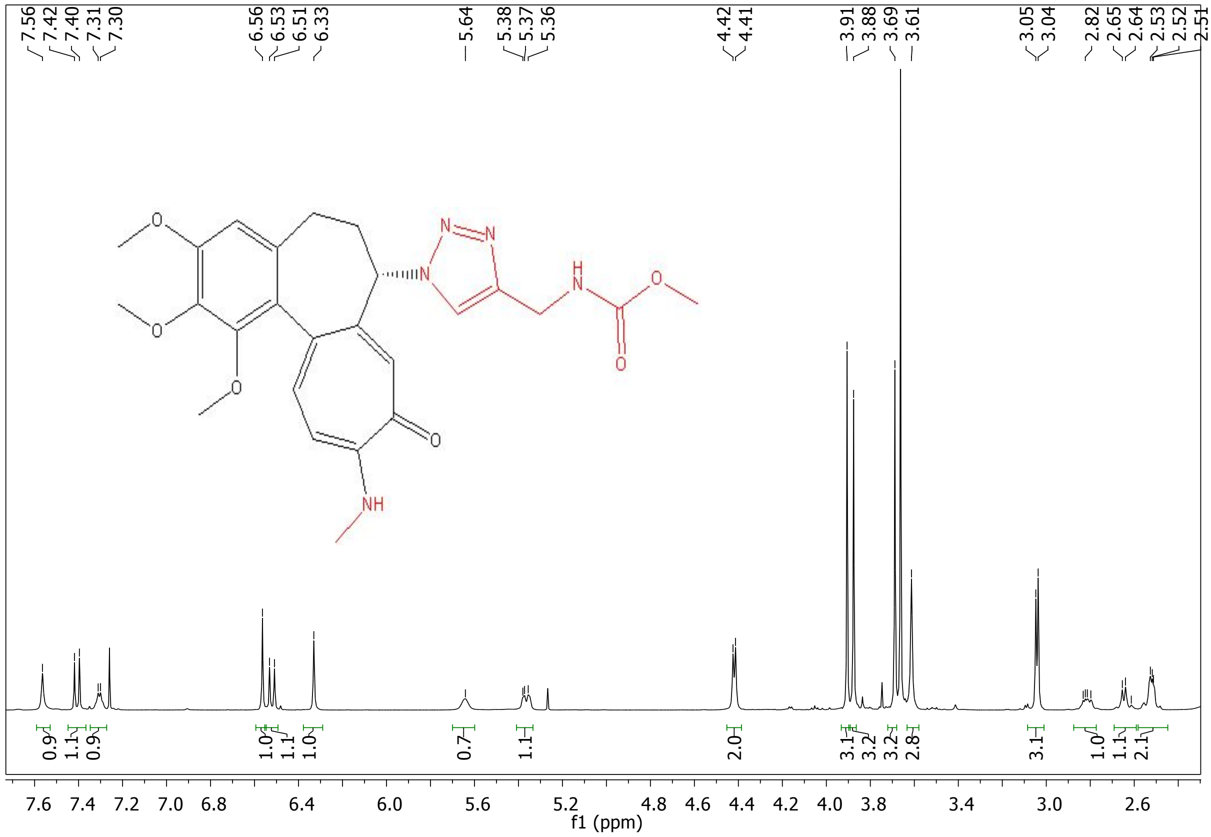

Figure S106. The ^{1}H NMR spectrum of **37** in $CDCl_3$.

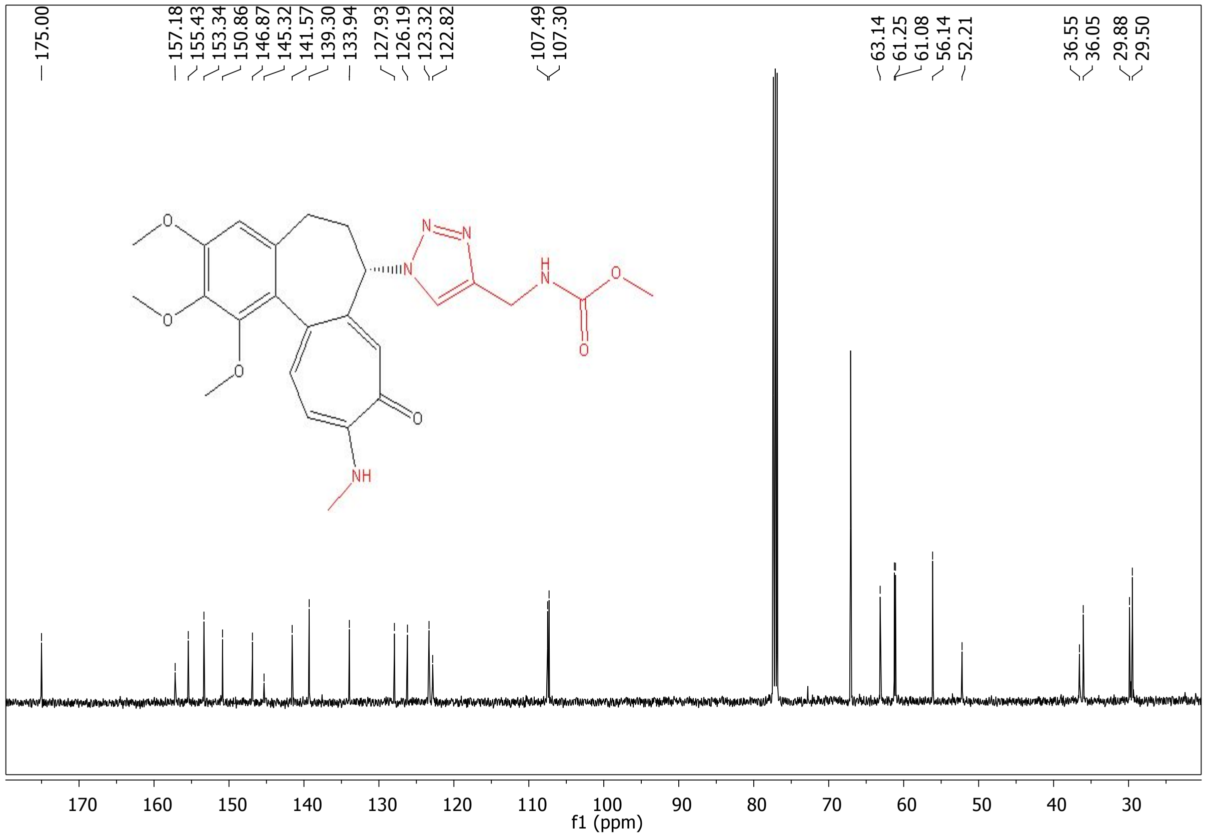

Figure S107. The ^{13}C NMR spectrum of **37** in $CDCl_3$.

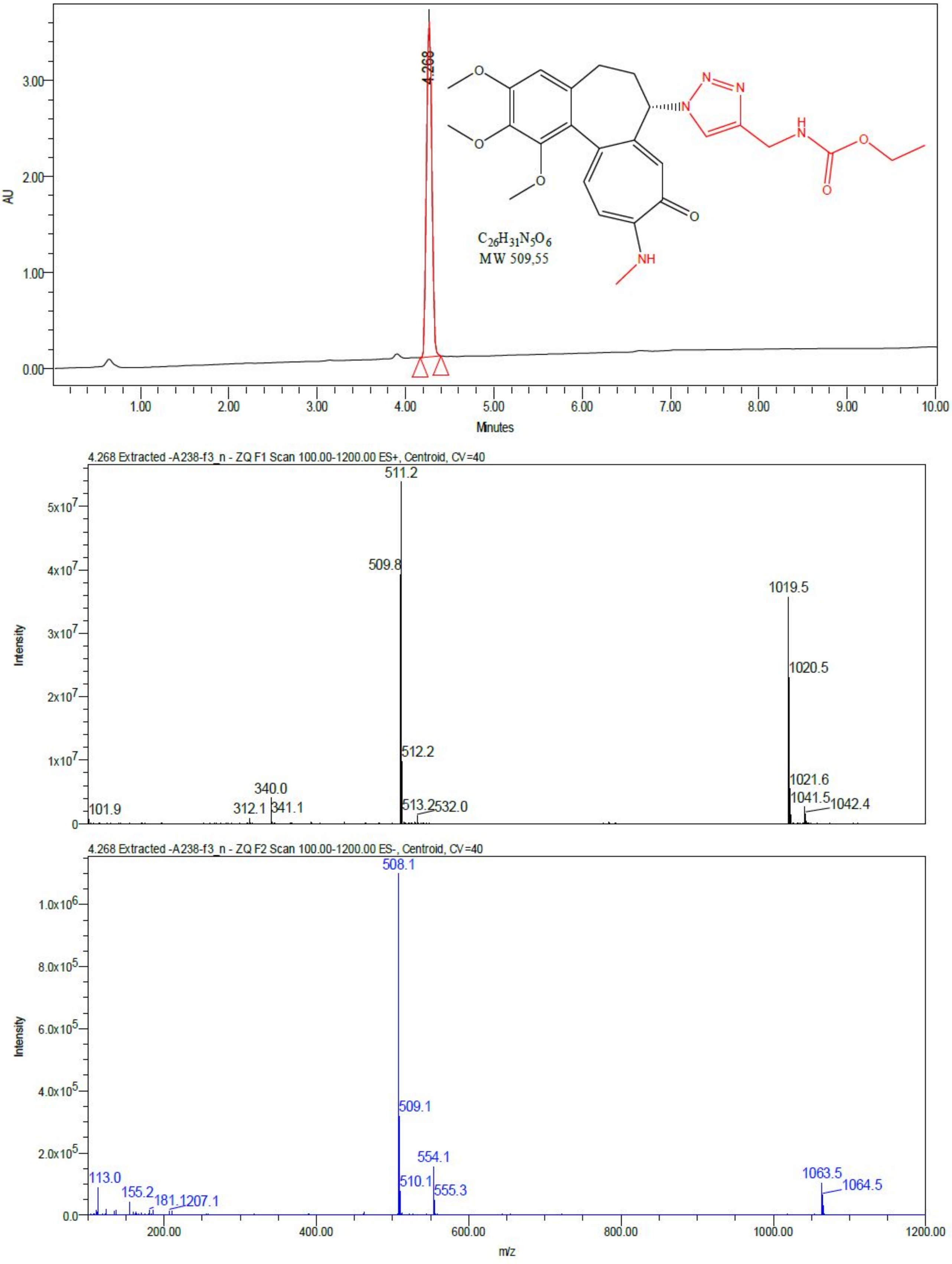

Figure S108. The LC-MS chromatogram and mass spectra of **38**.

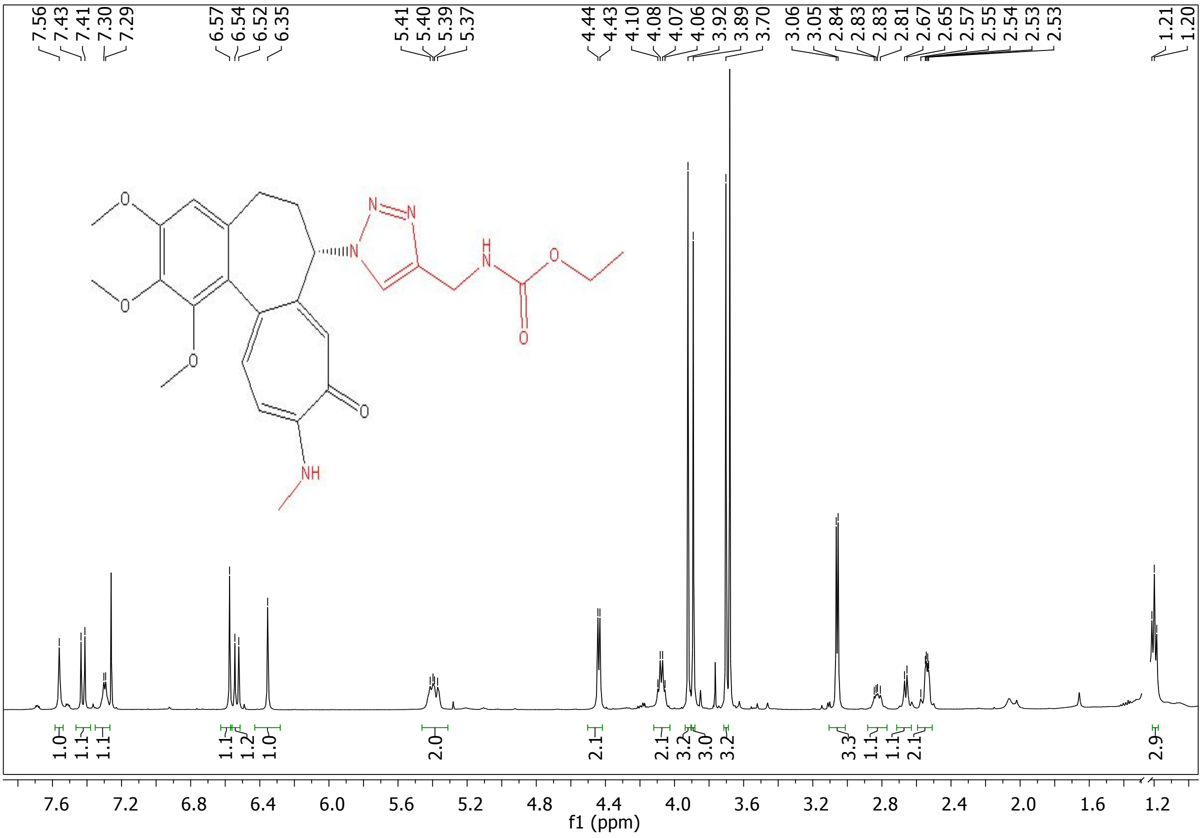

Figure S109. The ^{1}H NMR spectrum of **38** in $CDCl_3$.

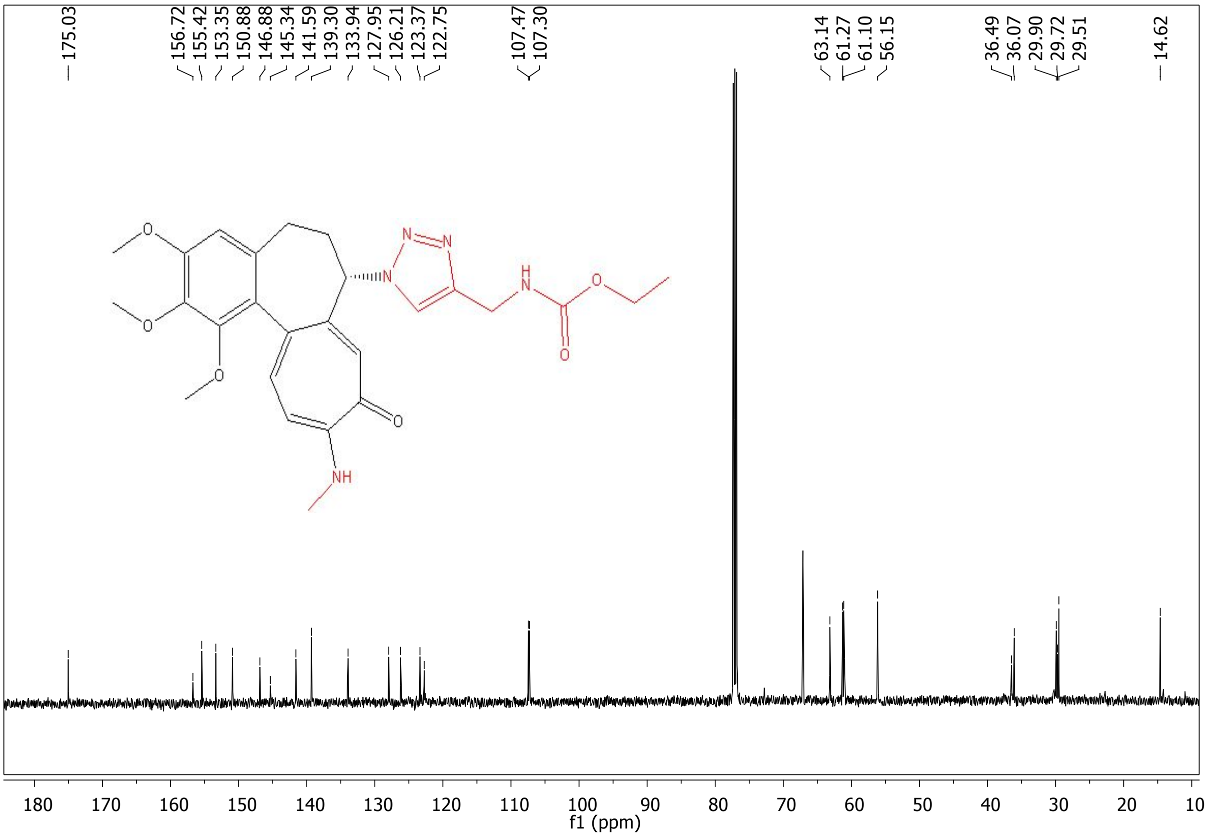

Figure S110. The ^{13}C NMR spectrum of **38** in $CDCl_3$.

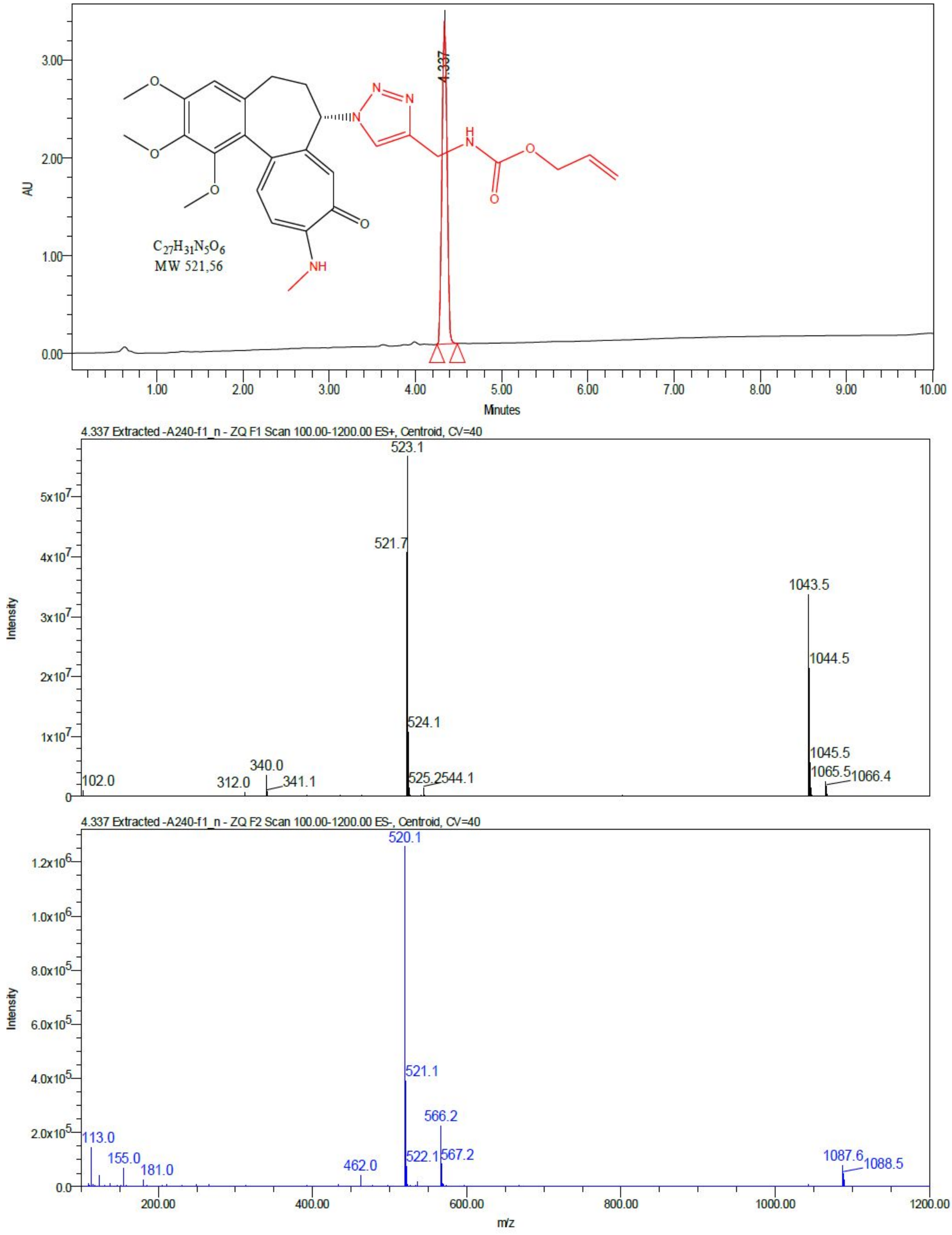

Figure S111. The LC-MS chromatogram and mass spectra of **39**.

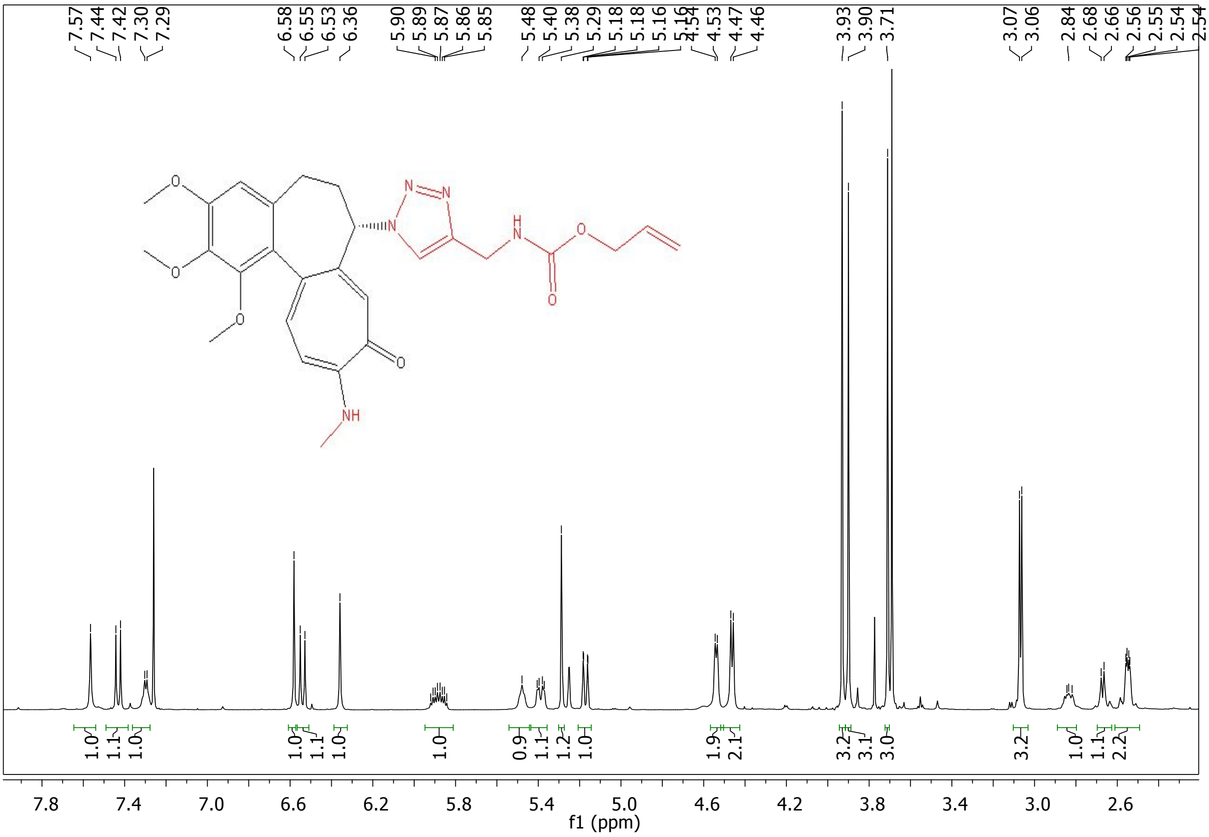

Figure S112. The ^{1}H NMR spectrum of **39** in $CDCl_3$.

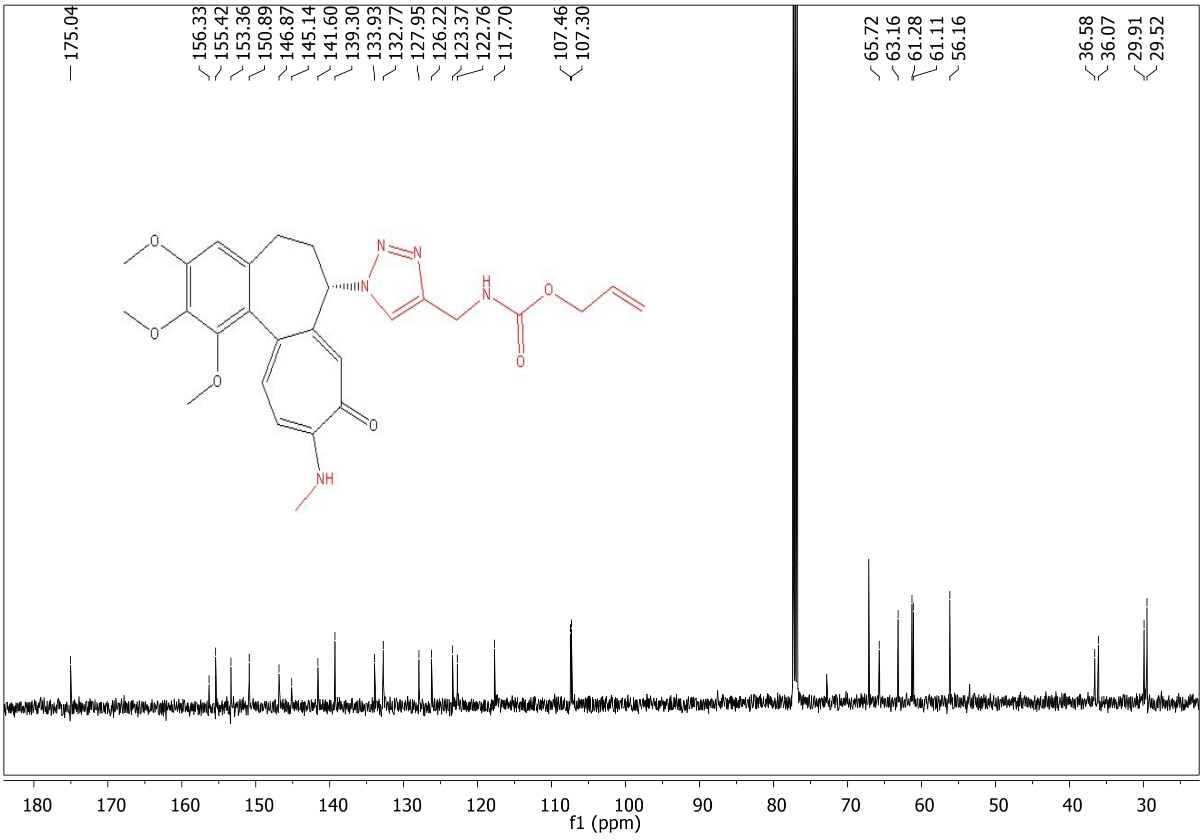

Figure S113. The ^{13}C NMR spectrum of **39** in $CDCl_3$.

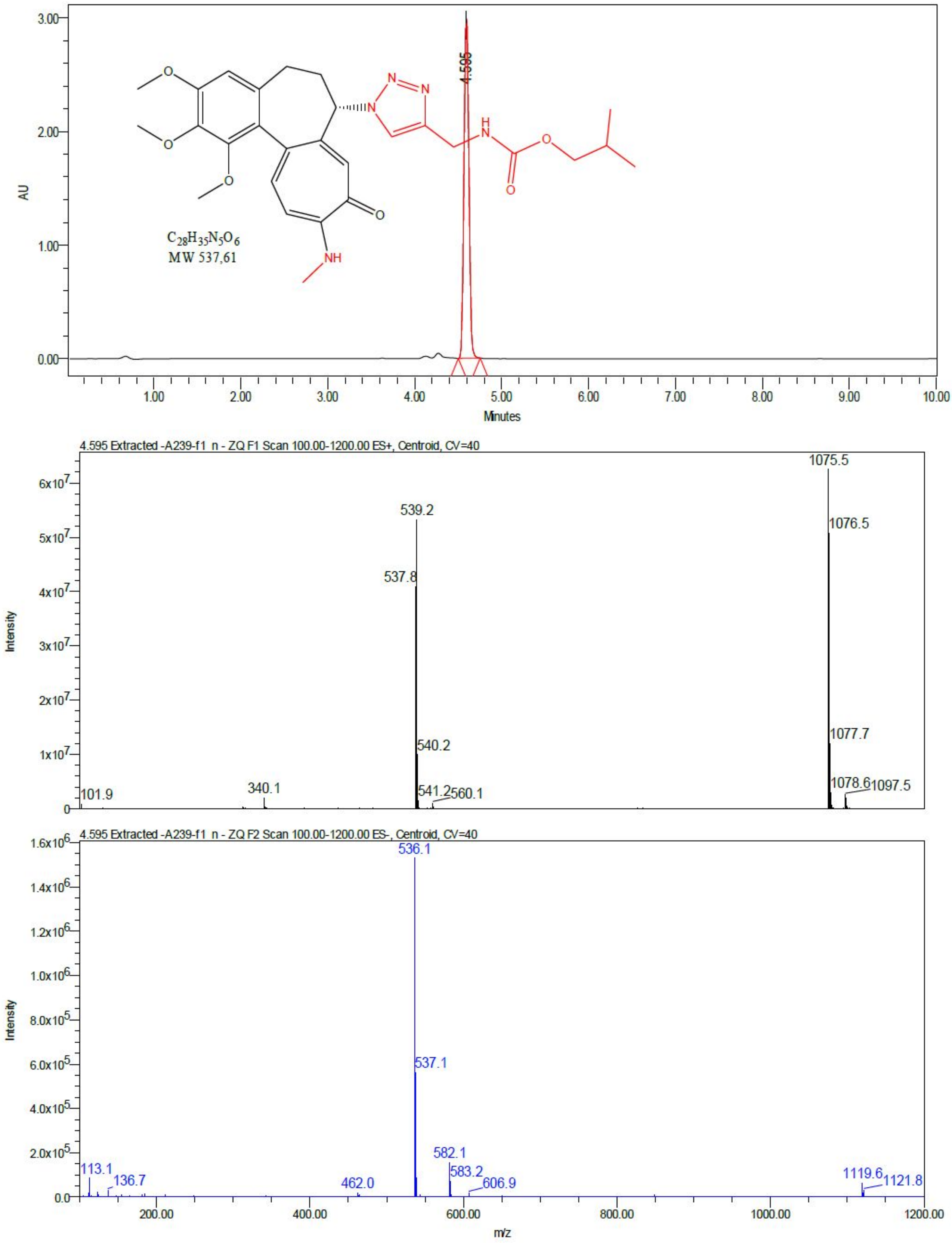

Figure S114. The LC-MS chromatogram and mass spectra of **40**.

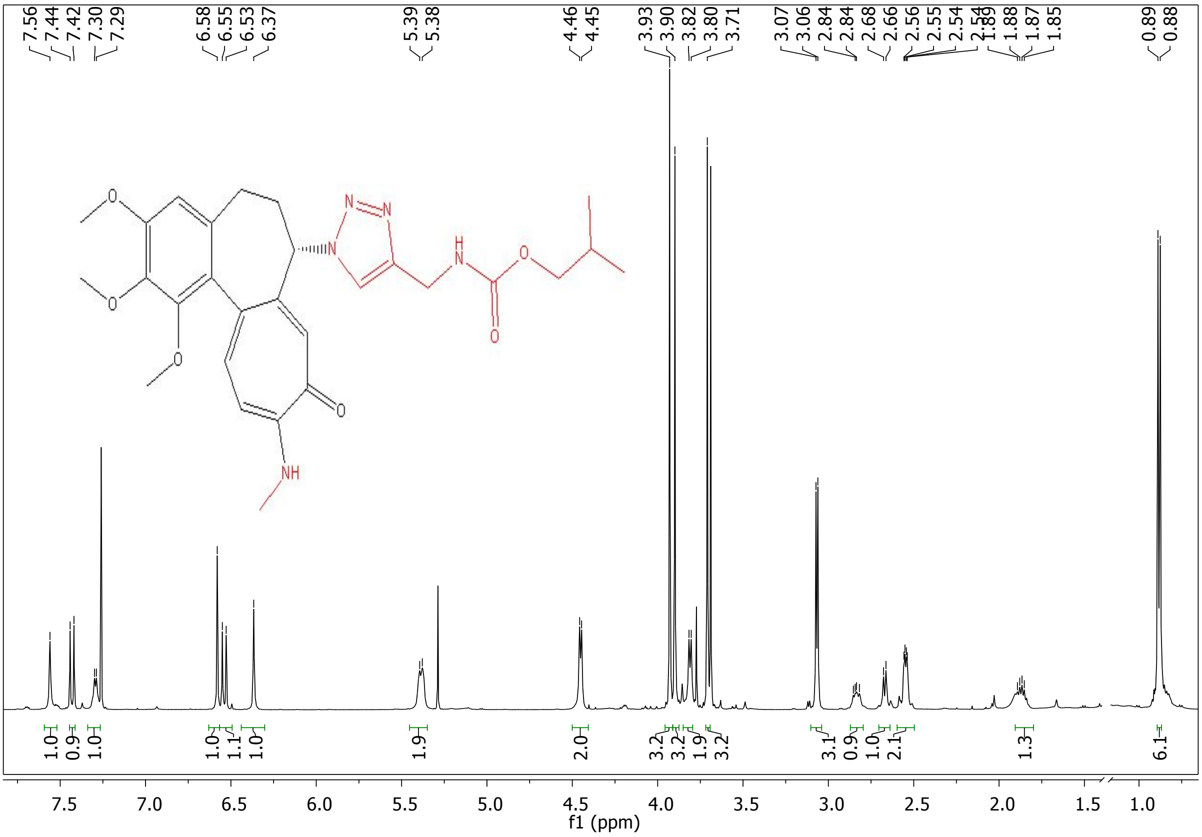

Figure S115. The ^{1}H NMR spectrum of **40** in $CDCl_3$.

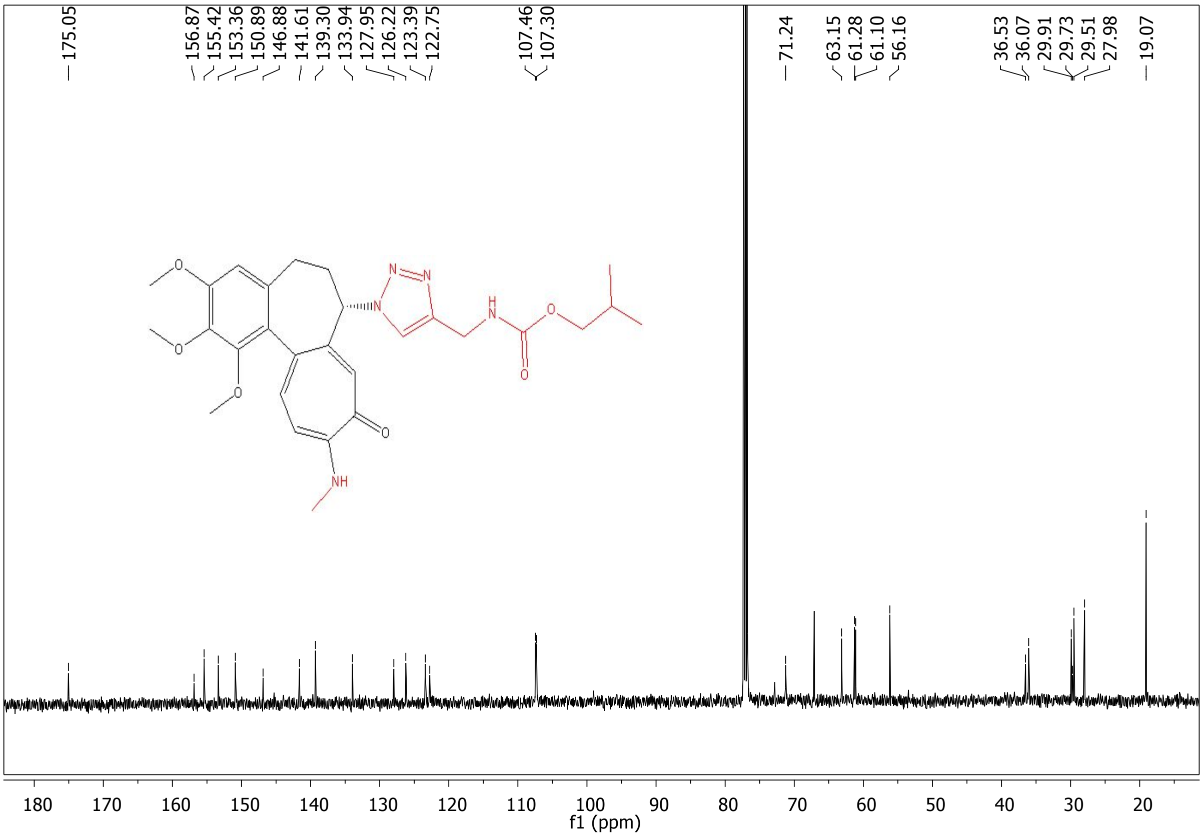

Figure S116. The ^{13}C NMR spectrum of **40** in $CDCl_3$.

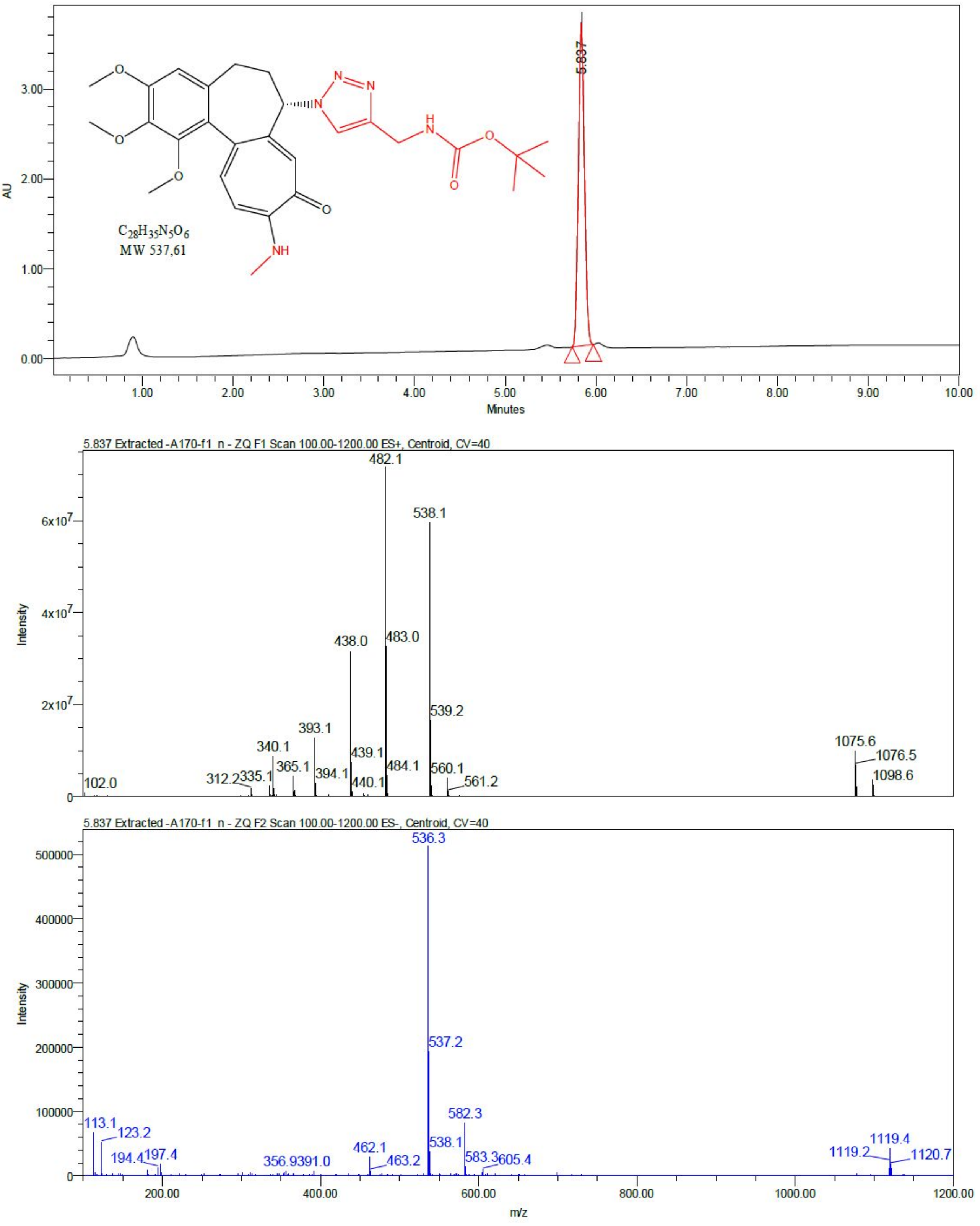

Figure S117. The LC-MS chromatogram and mass spectra of **41**.

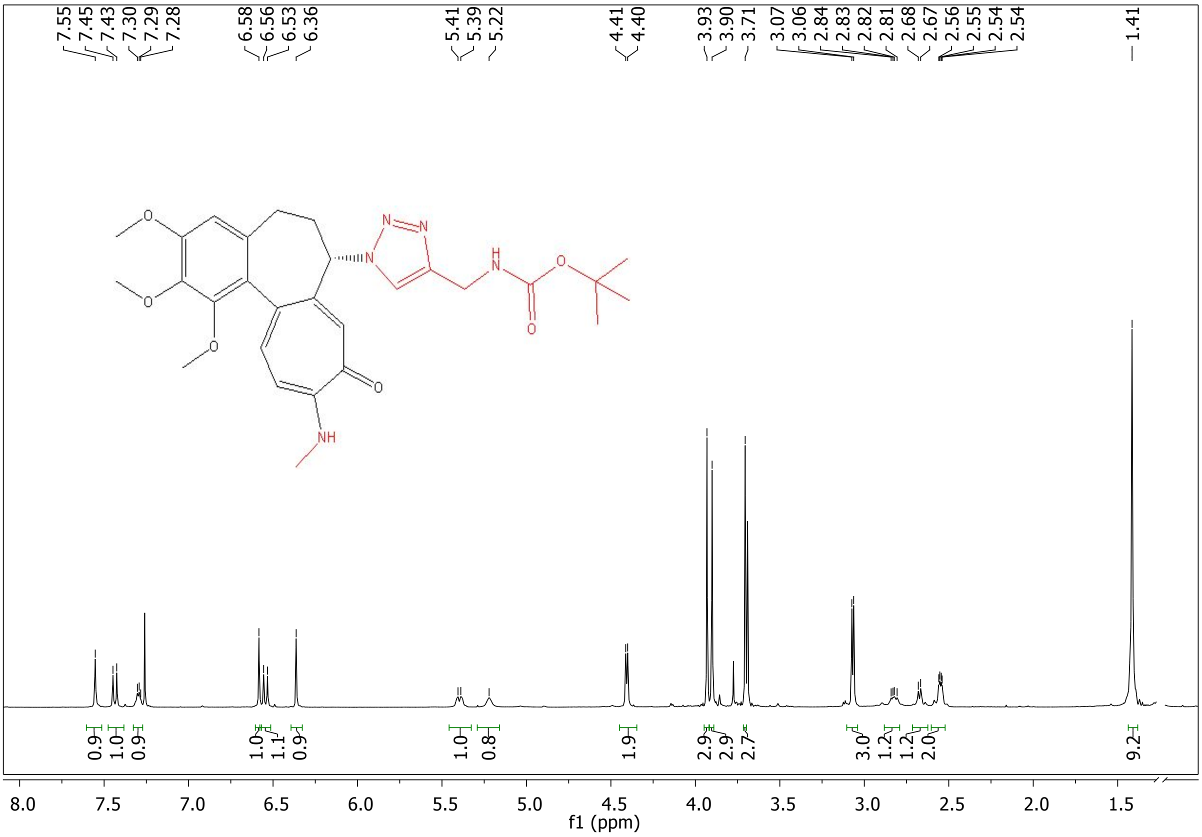

Figure S118. The ^{1}H NMR spectrum of **41** in $CDCl_3$.

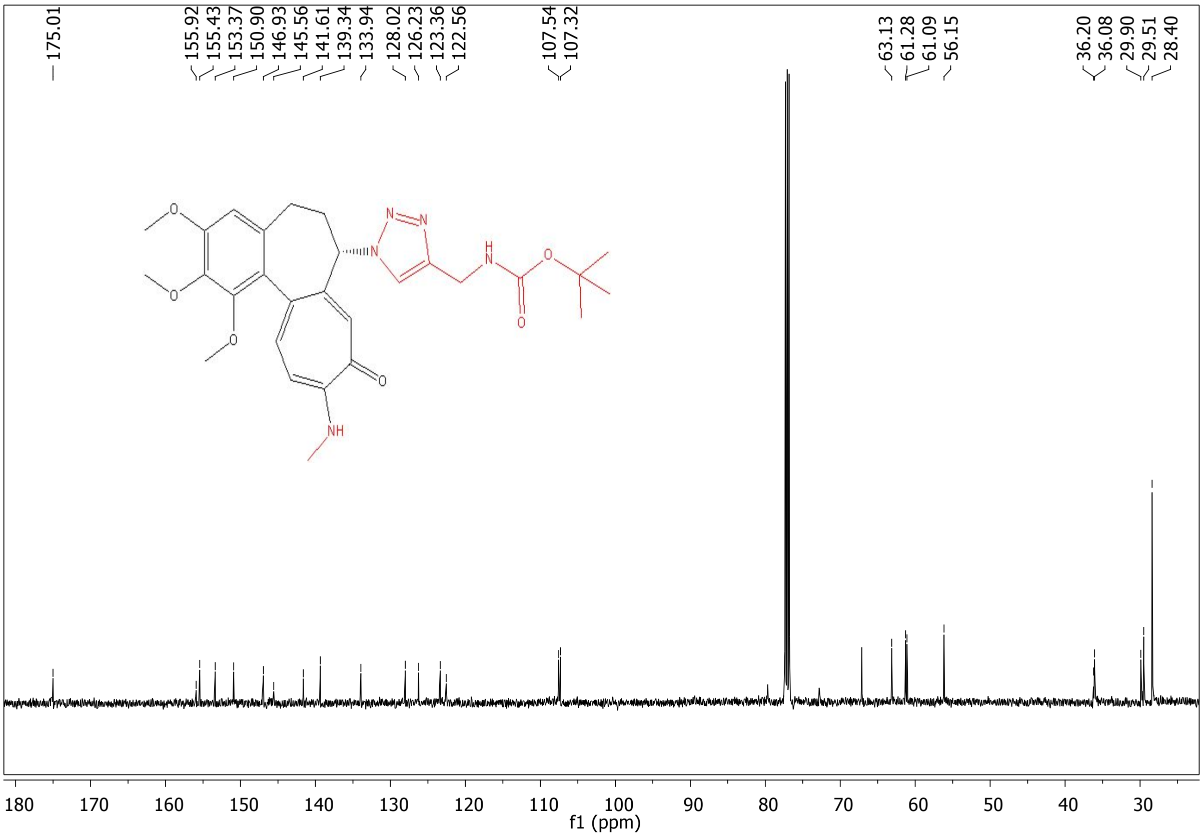

Figure S119. The ^{13}C NMR spectrum of **41** in $CDCl_3$.

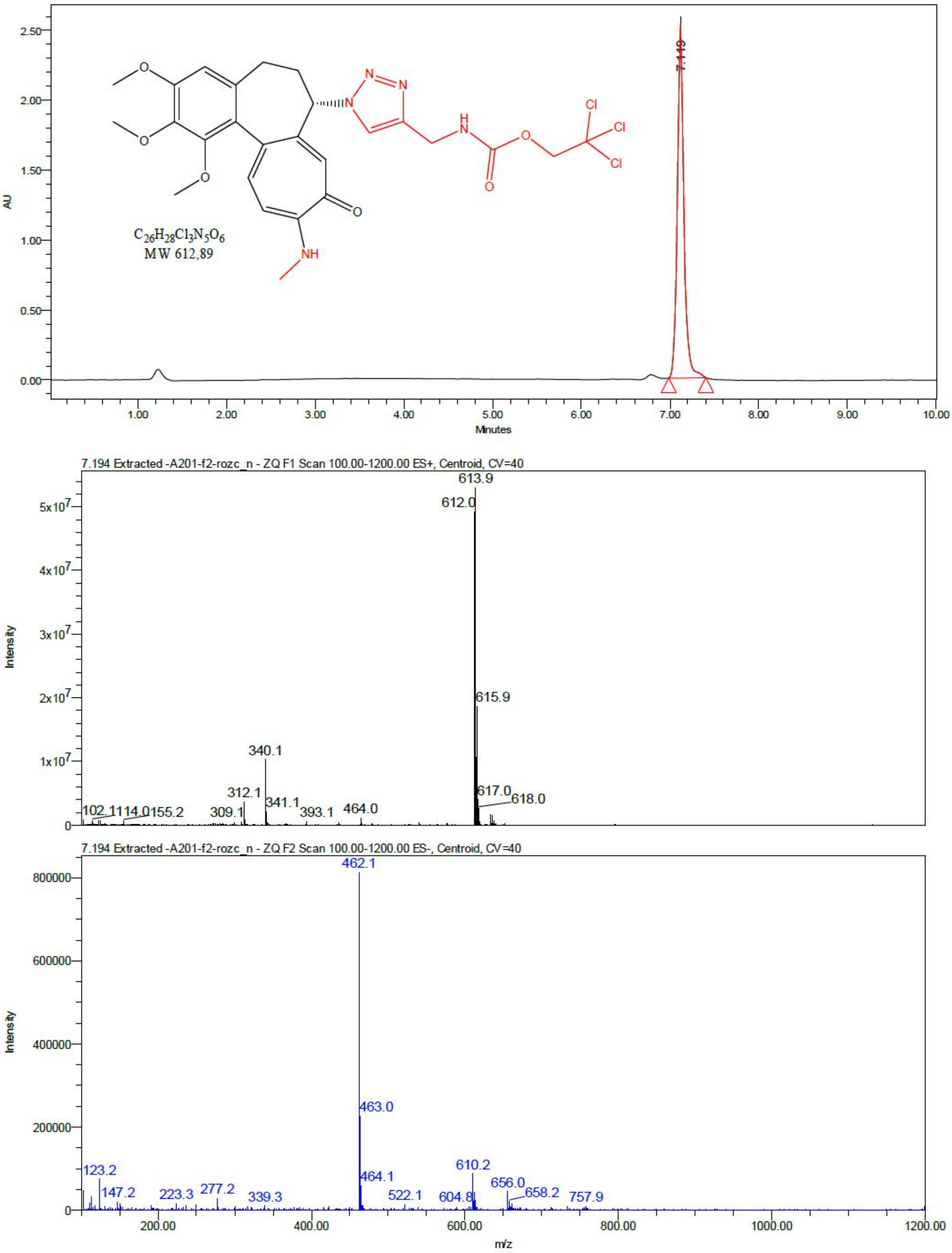

Figure S120. The LC-MS chromatogram and mass spectra of **42**.

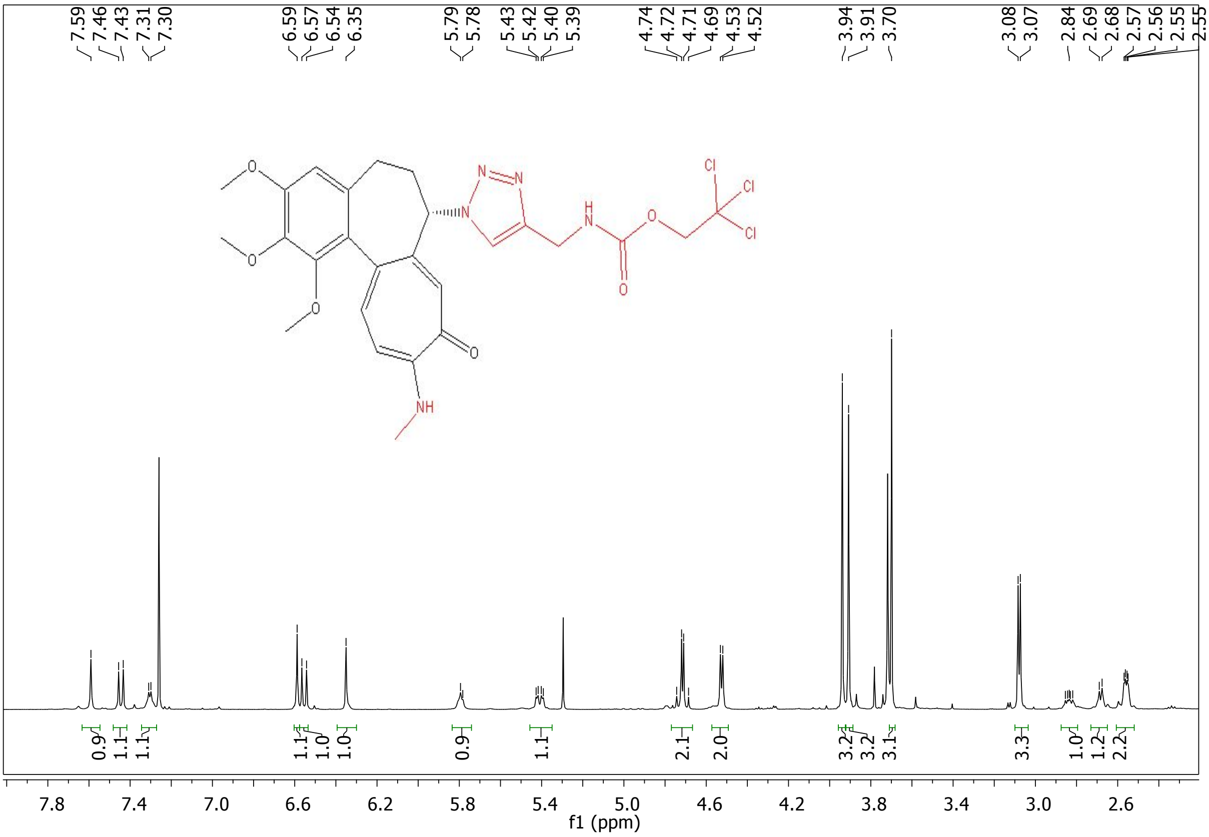

Figure S121. The ^{1}H NMR spectrum of **42** in $CDCl_3$.

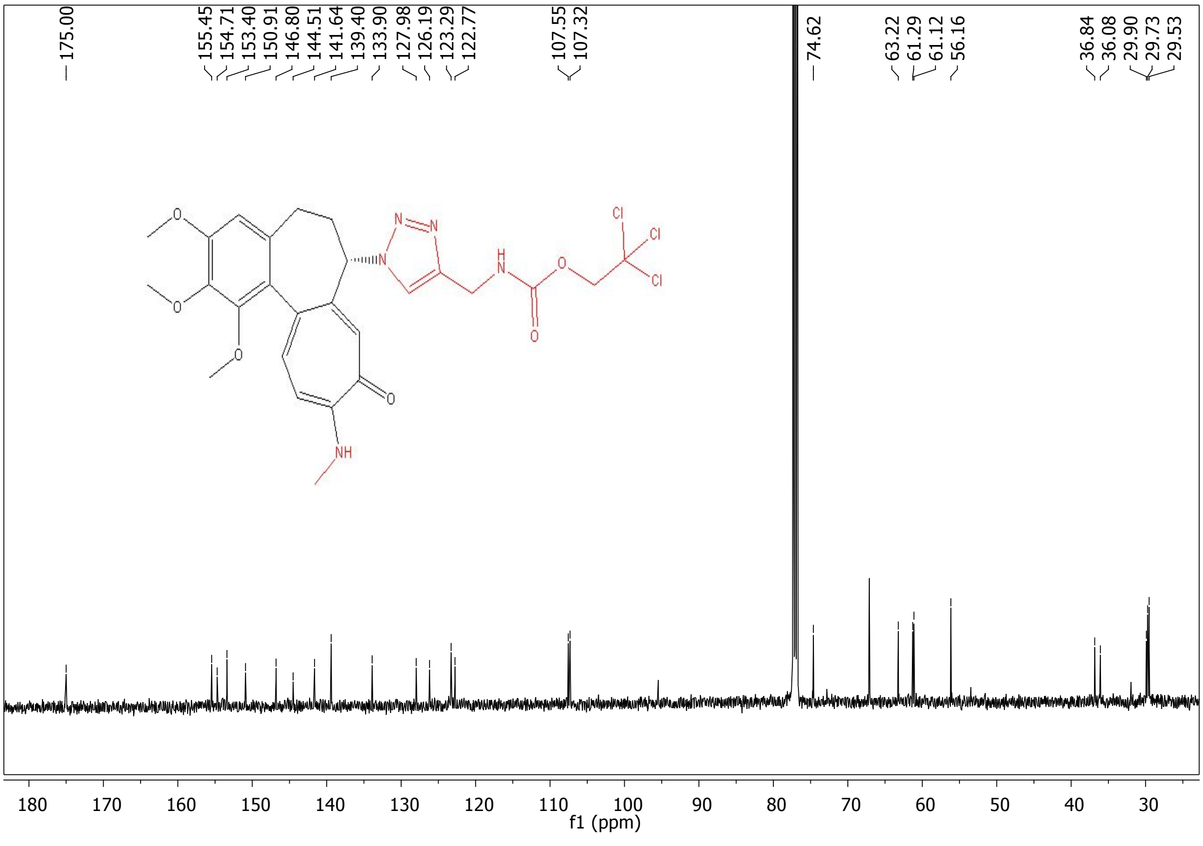

Figure S122. The ^{13}C NMR spectrum of **42** in $CDCl_3$.

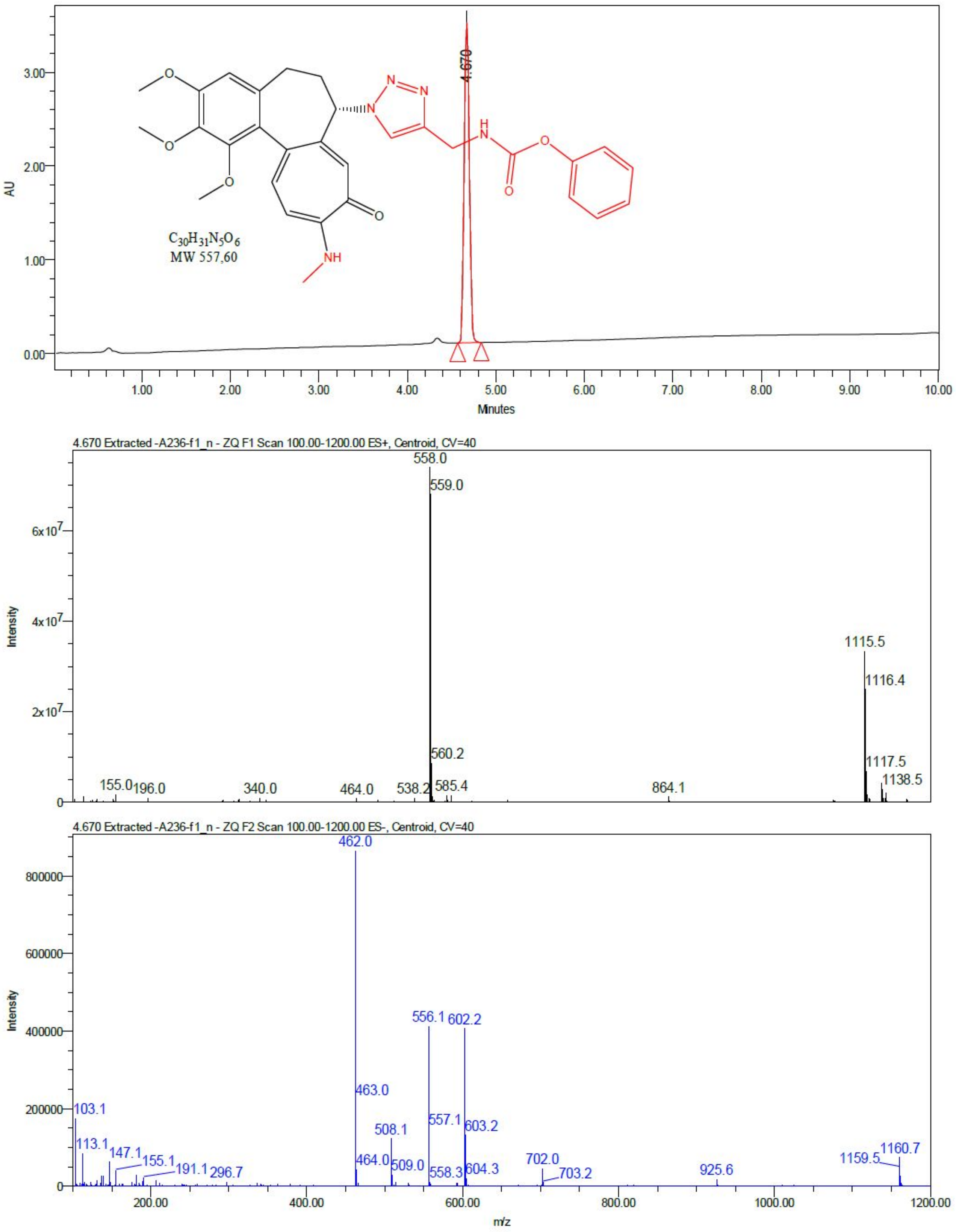

Figure S123. The LC-MS chromatogram and mass spectra of **43**.

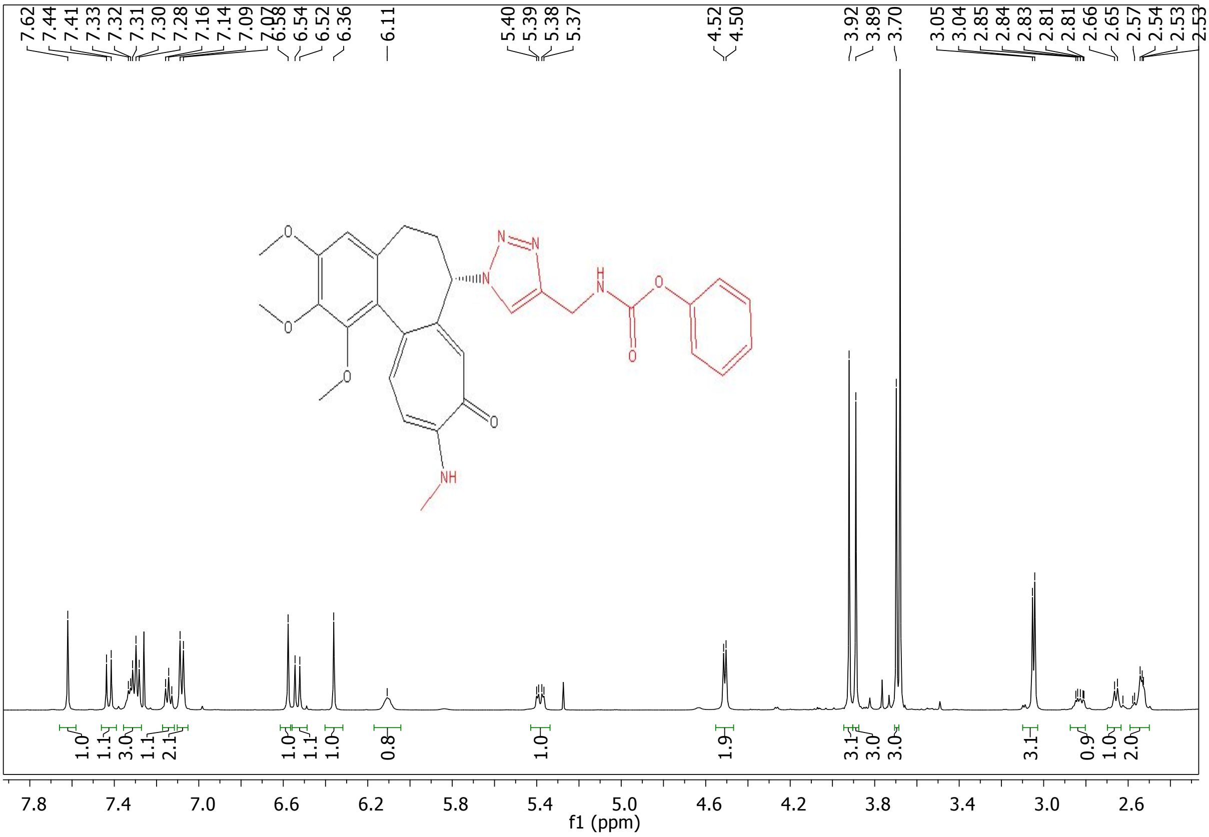

Figure S124. The ^{1}H NMR spectrum of **43** in $CDCl_3$.

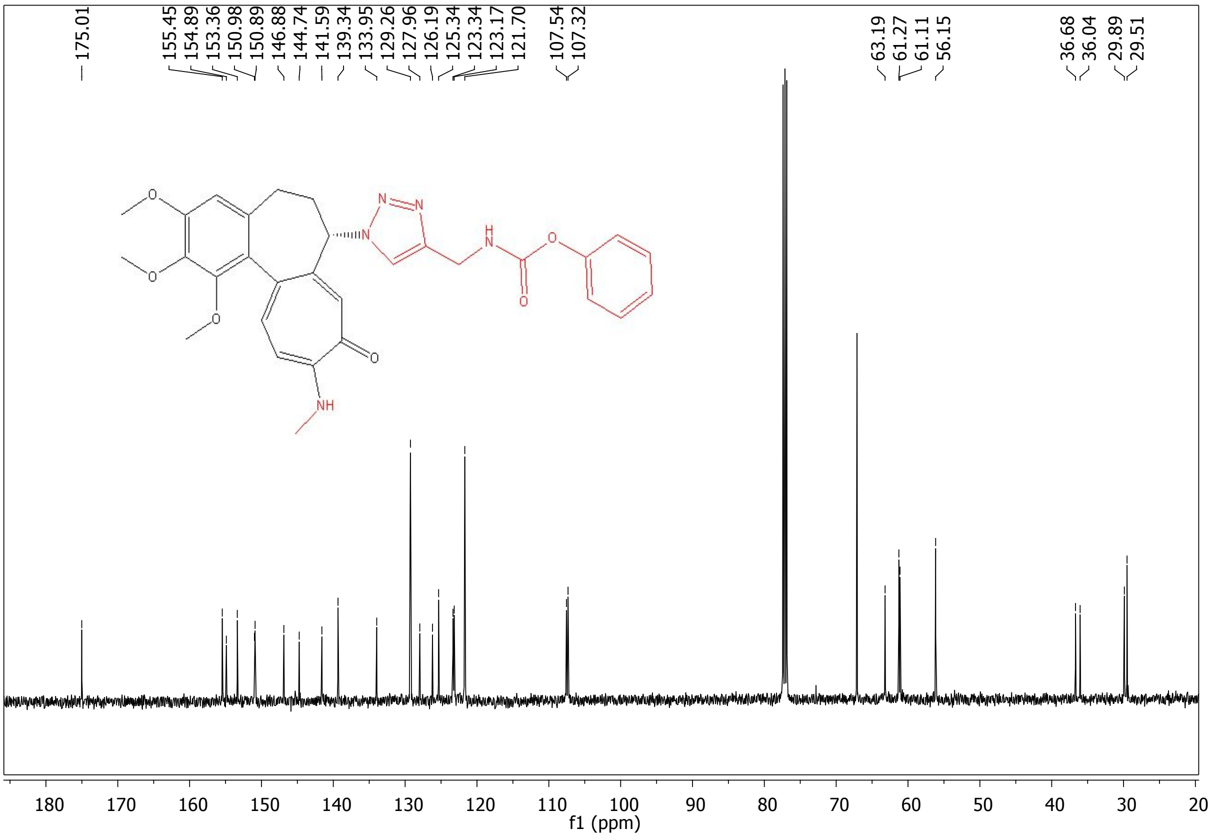

Figure S125. The ^{13}C NMR spectrum of **43** in $CDCl_3$.

FT-IR spectra of compounds 2-4, 9 and propargyl alcohol.

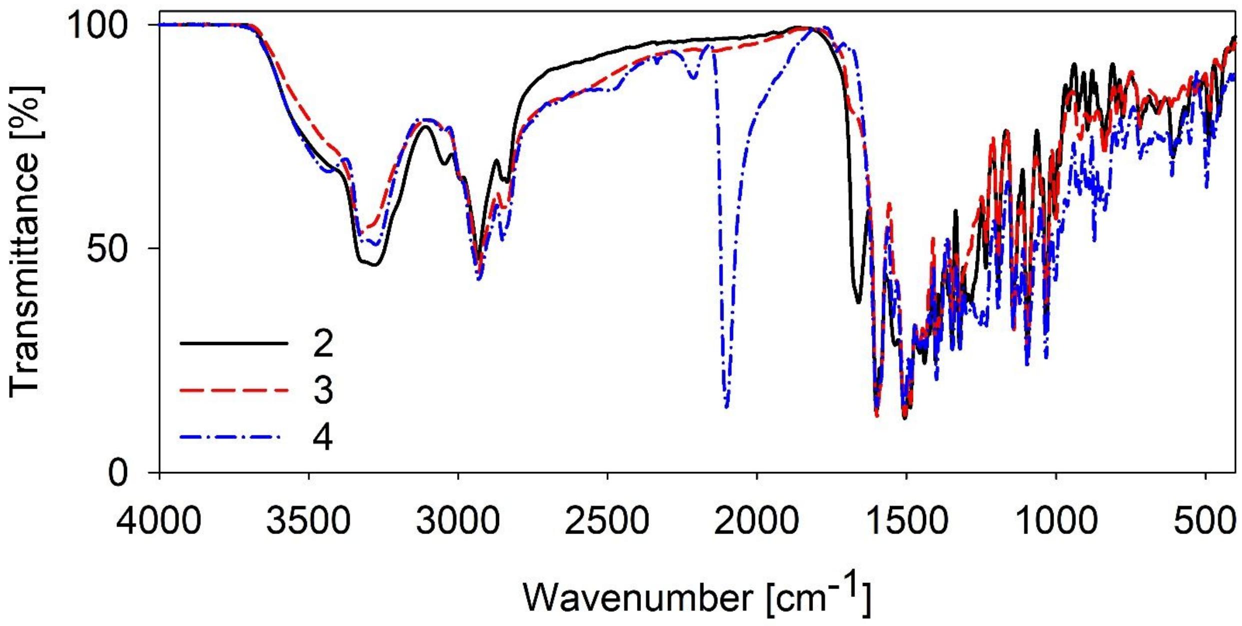

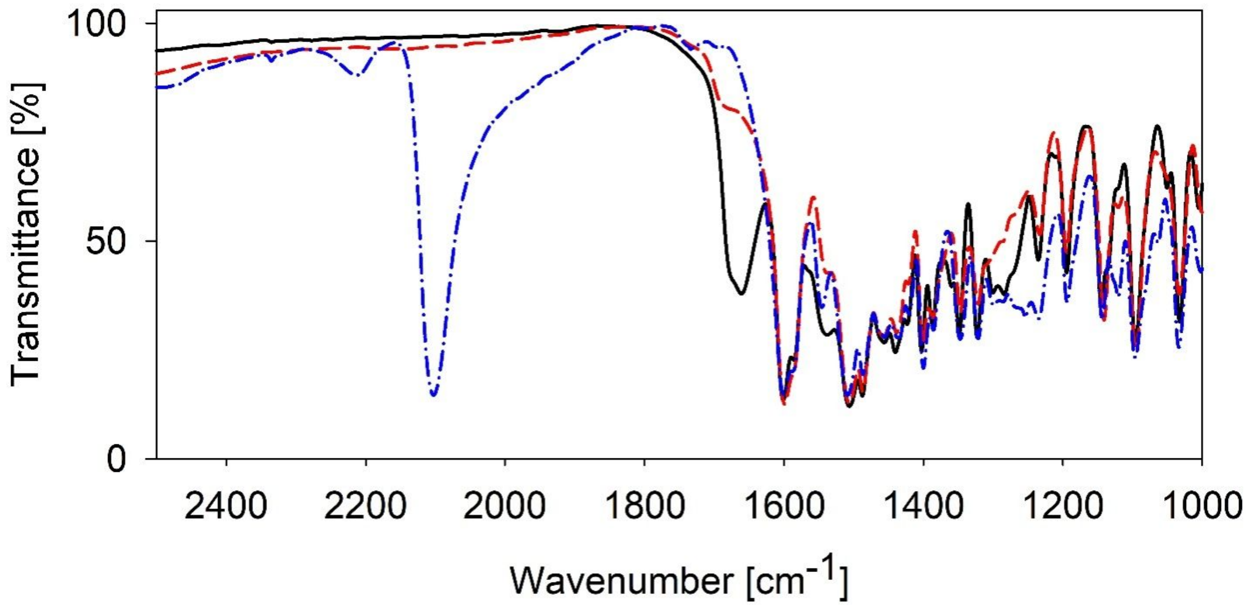

Figure S126. FT-IR spectra of (—) **2**, (- - -) **3** and (- • -) **4** in the ranges of: 4000–400 cm^{-1} and 2500-1000 cm^{-1}.

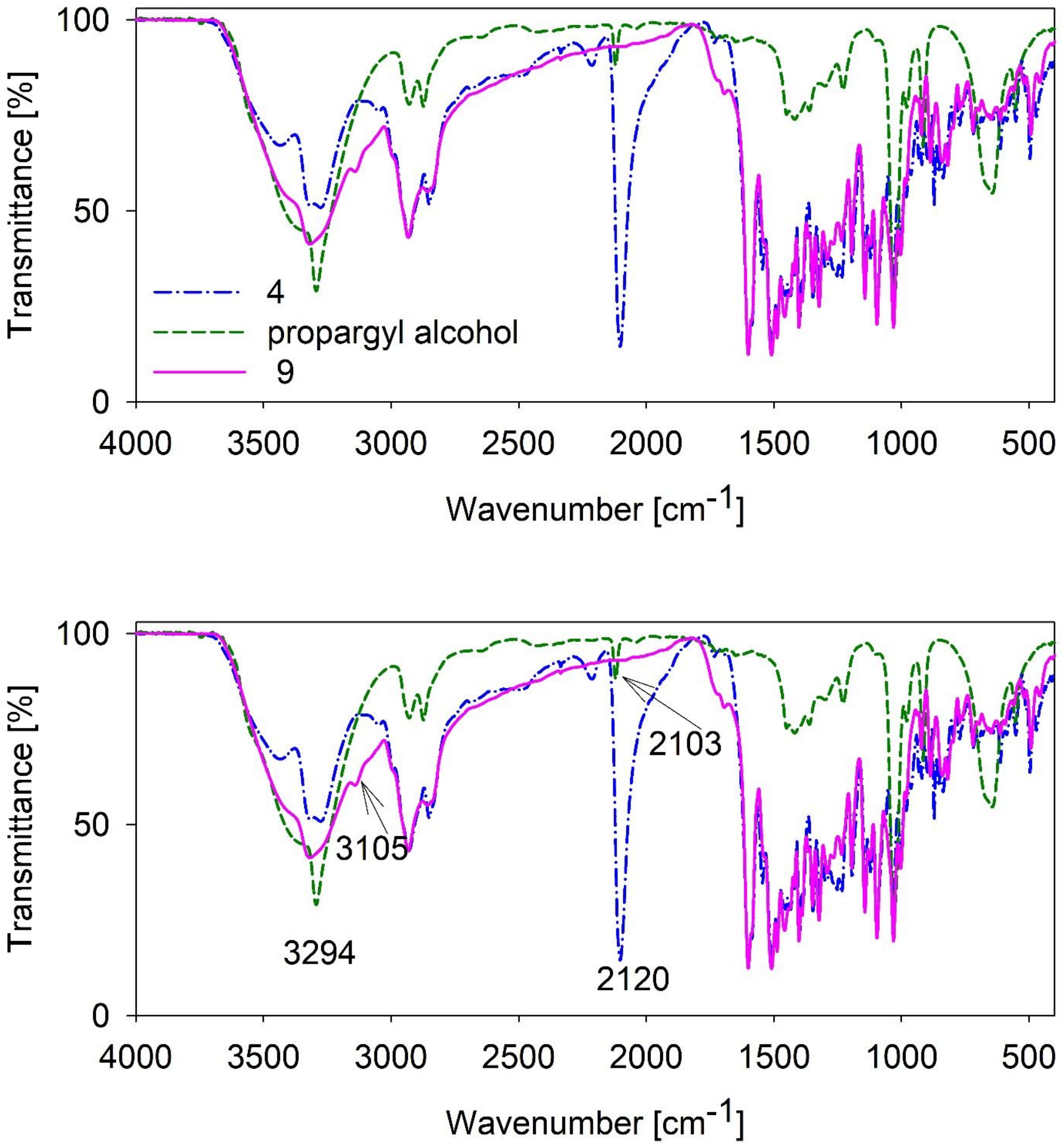

Figure S127. FT-IR spectra of (- • -) **4,** (- - -) **propargyl alcohol** (—) **9**, and in the ranges of: 4000–400 cm^{-1}.
